# Efficacy of sustained knowledge translation (KT) interventions in chronic disease management in older adults: systematic review and meta-analysis of complex interventions

**DOI:** 10.1186/s12916-023-02966-9

**Published:** 2023-07-24

**Authors:** Areti Angeliki Veroniki, Charlene Soobiah, Vera Nincic, Yonda Lai, Patricia Rios, Heather MacDonald, Paul A. Khan, Marco Ghassemi, Fatemeh Yazdi, Ross C. Brownson, David A. Chambers, Lisa R. Dolovich, Annemarie Edwards, Paul P. Glasziou, Ian D. Graham, Brenda R. Hemmelgarn, Bev J. Holmes, Wanrudee Isaranuwatchai, France Legare, Jessie McGowan, Justin Presseau, Janet E. Squires, Henry T. Stelfox, Lisa Strifler, Trudy Van der Weijden, Christine Fahim, Andrea C. Tricco, Sharon E. Straus

**Affiliations:** 1 Knowledge Translation Program, Li Ka Shing Knowledge Institute, St. Michael’s Hospital, 209 Victoria Street, East Building, Toronto, ON M5B 1T8 Canada; 2 Institute for Health Policy, Management, and Evaluation, University of Toronto, 155 College Street, Toronto, ON Canada; 3 Prevention Research Center in St. Louis, Brown School, Washington University in St. Louis, St. Louis, MO USA; 4 Department of Surgery and Alvin J. Siteman Cancer Center, Washington University School of Medicine, Washington University in St. Louis, 660 S. Euclid Avenue, St. Louis, MO USA; 5 National Cancer Institute, 9609 Medical Center Drive, Rockville, MD USA; 6 Leslie Dan Faculty of Pharmacy, University of Toronto, 144 College Street, Toronto, ON Canada; 7 Department of Family Medicine David Braley Health Sciences Centre, McMaster University, 100 Main Street West, Hamilton, ON Canada; 8 Canadian Partnership Against Cancer, 1 University Avenue, Toronto, ON Canada; 9 Faculty of Health Sciences and Medicine, Bond University, Robina, QLD 4226 Australia; 10 School of Epidemiology and Public Health, University of Ottawa, 600 Peter Morand Crescent, Ottawa, ON Canada; 11 The Ottawa Hospital Research Institute, 501 Smyth Road, Ottawa, ON Canada; 12 Department of Medicine, University of Alberta, C MacKenzie Health Sciences Centre, WalterEdmonton, AB 2J2.00 Canada; 13 The Michael Smith Foundation for Health Research (MSFHR), 200 - 1285 West Broadway, Vancouver, BC Canada; 14 Département de Médecine Familiale Et Médecine d’urgenceFaculté de Médecine, Université Laval Pavillon Ferdinand-Vandry1050, Avenue de La Médecine, Local 2431, Québec, QC Canada; 15 Axe Santé Des Populations Et Pratiques Optimales en Santé, Centre de Recherche du CHU de Québec 1050, Chemin Sainte-Foy, Local K0-03, Québec, QC Canada; 16 School of Nursing, University of Ottawa, 451 Smyth Road, Ottawa, ON K1H 8M5 Canada; 17 Departments of Critical Care Medicine, Medicine and Community Health Sciences, O’Brien Institute for Public Health, University of Calgary and Alberta Health Services, Calgary, AB Canada; 18 Department of Family Medicine, Maastricht University, CAPHRI Care and Public Health Research Institute, Debeyeplein 1, Maastricht, The Netherlands; 19 Epidemiology Division & Institute of Health Policy, Management, and Evaluation, Dalla Lana School of Public Health, University of Toronto, Toronto, ON Canada; 20 Department of Geriatric Medicine, University of Toronto, Toronto, ON Canada

**Keywords:** Sustainability, Knowledge translation, Chronic disease management, Older adults, Integrated knowledge translation, Patient and public involvement

## Abstract

**Background:**

Chronic disease management (CDM) through sustained knowledge translation (KT) interventions ensures long-term, high-quality care. We assessed implementation of KT interventions for supporting CDM and their efficacy when sustained in older adults.

**Methods:**

Design: Systematic review with meta-analysis engaging 17 knowledge users using integrated KT.

Eligibility criteria: Randomized controlled trials (RCTs) including adults (> 65 years old) with chronic disease(s), their caregivers, health and/or policy-decision makers receiving a KT intervention to carry out a CDM intervention for at least 12 months (versus other KT interventions or usual care).

Information sources: We searched MEDLINE, EMBASE, and the Cochrane Central Register of Controlled Trials from each database’s inception to March 2020.

Outcome measures: Sustainability, fidelity, adherence of KT interventions for CDM practice, quality of life (QOL) and quality of care (QOC).

Data extraction, risk of bias (ROB) assessment: We screened, abstracted and appraised articles (Effective Practice and Organisation of Care ROB tool) independently and in duplicate. Data synthesis: We performed both random-effects and fixed-effect meta-analyses and estimated mean differences (MDs) for continuous and odds ratios (ORs) for dichotomous data.

**Results:**

We included 158 RCTs (973,074 participants [961,745 patients, 5540 caregivers, 5789 providers]) and 39 companion reports comprising 329 KT interventions, involving patients (43.2%), healthcare providers (20.7%) or both (10.9%). We identified 16 studies described as assessing sustainability in 8.1% interventions, 67 studies as assessing adherence in 35.6% interventions and 20 studies as assessing fidelity in 8.7% of the interventions. Most meta-analyses suggested that KT interventions improved QOL, but imprecisely (36 item Short-Form mental [SF-36 mental]: MD 1.11, 95% confidence interval [CI] [− 1.25, 3.47], 14 RCTs, 5876 participants, *I*^2^ = 96%; European QOL-5 dimensions: MD 0.01, 95% CI [− 0.01, 0.02], 15 RCTs, 6628 participants, *I*^2^ = 25%; St George’s Respiratory Questionnaire: MD − 2.12, 95% CI [− 3.72, − 0.51] 44 12 RCTs, 2893 participants, *I*^2^ = 44%). KT interventions improved QOC (OR 1.55, 95% CI [1.29, 1.85], 12 RCTS, 5271 participants, *I*^2^ = 21%).

**Conclusions:**

KT intervention sustainability was infrequently defined and assessed. Sustained KT interventions have the potential to improve QOL and QOC in older adults with CDM. However, their overall efficacy remains uncertain and it varies by effect modifiers, including intervention type, chronic disease number, comorbidities, and participant age.

**Systematic review registration:**

PROSPERO CRD42018084810.

**Supplementary Information:**

The online version contains supplementary material available at 10.1186/s12916-023-02966-9.

## Summary box

**What is already known on this topic**

- Sustainability of knowledge translation (KT) interventions supporting implementation of chronic disease management (CDM) in older adults (> 65 years) with chronic diseases is vital to ensure long-term, high-quality patient care.

**What this study adds**

- Few RCTs assessed sustainability, fidelity, and adherence of KT interventions for CDM practice for at least 1 year.
- Sparce evidence assessing quality of life and care following sustained KT interventions present KT knowledge gaps and analytical challenges.
- More studies providing an operational standardized measure of sustained KT interventions are necessary to explore patient outcome heterogeneity and robust conclusions regarding treatments and associated results.

## Background

Evidence-based clinical interventions (i.e. early mobilisation in older adults or heart failure medications use) require tailored knowledge translation (KT or implementation) interventions (i.e. patient education or team changes) to optimise use in practice or policy. KT interventions are strategies that facilitate research uptake in practice and policy and include any action or set of actions that target factors that hinder or help someone to use a new practice or evidence-based program [1]. KT interventions are diverse and can focus on patients, caregivers, clinicians, managers and policy makers [2, 3]. Adoption of KT interventions can impact patient care and health system outcomes; however, there is a tendency to return to prior behaviours after initial interventions end [4]. Sustainability of KT interventions is defined as the continued delivery of clinical and KT intervention after its adoption is secured over a period of time (depending on the implementation context), while producing benefits for individuals and systems [5]. Failure to sustain KT interventions can lead to declining patient and health system outcomes and diminish confidence and support for future KT [6, 7].

Adults aged 65 years and older are the largest growing proportion of the global population, and many are affected by chronic diseases [8, 9]. Evidence-based clinical interventions to manage these conditions often include a combination of pharmacological and non-pharmacological interventions. However, to optimise intervention impact, their use needs to be supported at the patient, healthcare provider and health system levels via KT interventions [6]. Sustainability of KT interventions to manage chronic diseases is of paramount importance to ensure long-term, high-quality patient care and optimise health system impact consistently [10–13]. Specifically, optimal chronic disease management (CDM) in older adults requires sustained use of CDM interventions via effective KT interventions [14]. More importantly, it is expected that fostering sustainability will help reduce waste in health by facilitating their effective use. Our previous scoping review on the sustainability of KT interventions to manage CDM in adults included 62 experimental, quasi-experimental and observational studies assessing 13 different types of KT interventions [14]. Evidence showed that 56.1% of the eligible patients received a KT intervention for CDM, and even fewer maintained their use (e.g. 45.4% with diabetes mellitus, 24.7% with atrial fibrillation) over 2 years [15]. Moreover, it remains unclear which KT interventions and their individual components are most effective and sustained to optimise CDM.

The aim of this systematic review and meta-analysis was to describe sustainability of KT to implement a CDM intervention for at least 12 months by engaging 17 knowledge users, including patient partners, throughout using integrated KT. A knowledge user is defined as an individual who is likely to be able to use research results to inform their decisions about health policies, programs and practices (e.g. clinicians, managers, policy makers, patients/families and others) [16, 17]. We aimed to systematically assess the efficacy of sustainability of KT intervention for CDM end-users with comorbid conditions including older patients, their caregivers, health and policy-decision makers on healthcare outcomes (including quality of life [QOL] and quality of care [QOC]) at least 1 year after CDM intervention implementation or the termination of initial funding.

## Methods

We registered our protocol with PROSPERO (CRD42018084810) and published it in an open-access journal [18]. Our systematic review follows the PRISMA 2020 [19] and GRIPP-2 [20] reporting guidelines. Our methods are described briefly here (see also Additional file 1: Appendix 1 [3, 14, 18, 21–40]). Any deviations from the protocol are reported in Additional file 1: Appendix 2 [41, 42].

### Knowledge user engagement

We enhanced systematic review conduct by employing an integrated KT approach [4] from project onset via established partnerships with 17 knowledge users, including one patient partner (KT), one funder (DAC), one policymaker (AE), 11 international KT researchers (BRH, IDG, JES, JM, JP, LRD, LS, PPG, RCB, WI, TVdW) and four clinicians (BH, FL, HTS, SES). The knowledge users provided input throughout the research process, including formulation of the research question, study protocol, prioritization of outcome measures and interpretation of results based on context relevance [18].

### Eligibility criteria, search strategy and selection process

We included randomised controlled trials (RCTs) where the target population for the CDM intervention included patients (at least 65 years old with one or more chronic disease [22]) or their caregiver. End-users of the KT intervention to implement a CDM intervention for at least 12 months included patients aged 65 years and older with at least one chronic disease, their caregivers, clinicians (all disciplines), public health officials, health care managers and policy-makers. RCTs comparing a KT intervention versus other KT interventions or usual care were eligible.

KT interventions were classified using (1) a pre-existing taxonomy developed by the Cochrane Effective Practice and Organisation of Care (EPOC) group and (2) the behaviour change technique (BCT) taxonomy. The primary outcome was sustained implementation of a KT intervention for CDM beyond 1 year after implementation or termination of funding and which KT interventions were used (Additional file 1: Appendix 3 [14]). Secondary outcomes were health-related or disease-specific QOL and process or QOC (Additional file 1: Appendix 4).

We searched the bibliographic databases MEDLINE, EMBASE, and CENTRAL up to March 4, 2020, and developed a grey literature search strategy [21] to seek unpublished studies (Additional file 1: Appendix 5). Reviewers independently and in duplicate screened titles/abstracts in level one and similarly full-text articles in level two. Pairs of reviewers independently abstracted data from each included study. Two pairs of reviewers (ACT, CF, CS, SES) coded each KT intervention within the included studies independently using EPOC and BCT taxonomies [3, 14, 23] (Additional file 1: Appendices 5, 6 and 7 [43–59]).

### Within and across study bias assessment

Pairs of reviewers appraised included studies using the EPOC risk of bias (ROB) tool independently [29]. We visually inspected small-study effects and reporting bias using the contour-enhanced funnel plot and Egger’s test when at least ten studies were available [30].

### Synthesis

We performed a descriptive analysis for the primary outcome and sustainability of KT interventions and used frequencies and percentages for the a priori defined KT dimensions: sustainability, adherence and fidelity assessment.

We combined study-level data in a meta-analysis using the mean difference (MD) for continuous outcomes (i.e. QOL) and odds ratio (OR) for dichotomous outcomes (i.e. QOC) along with corresponding 95% confidence intervals (95% CI) when at least two studies were available. We performed both random-effects and fixed-effect meta-analysis models using the inverse-variance method. Under the random-effects model, we estimated the overall effect size and its 95% CI using the Hartung–Knapp–Sidik–Jonkman method to handle meta-analyses with few studies [39–41]. In line with recent recommendations, when the estimated heterogeneity was positive (> 0) and at least three studies were included in the meta-analysis, we prioritized the random-effects model, since we expected the studies to be methodologically and clinically different [27, 37]. When the estimated heterogeneity was zero, we prioritized the fixed-effect model since the Hartung–Knapp–Sidik–Jonkman method is considered inadequate [26, 33, 39–41, 60]. When two studies were included and the estimated heterogeneity was positive (> 0), we presented both fixed and random effects findings [37]. We calculated prediction intervals (PIs) for the overall effect under the random-effects model to capture the interval within which we expected the true intervention effect of a new study to fall. We used the restricted maximum likelihood method [29] to estimate the between-study variance *τ*^2^ and the Q-profile approach to calculate its 95% CI [32, 36]. We explored potential heterogeneity using predefined meta-regression, subgroup or sensitivity analyses.

## Results

### Study selection

**Fig. 1:**
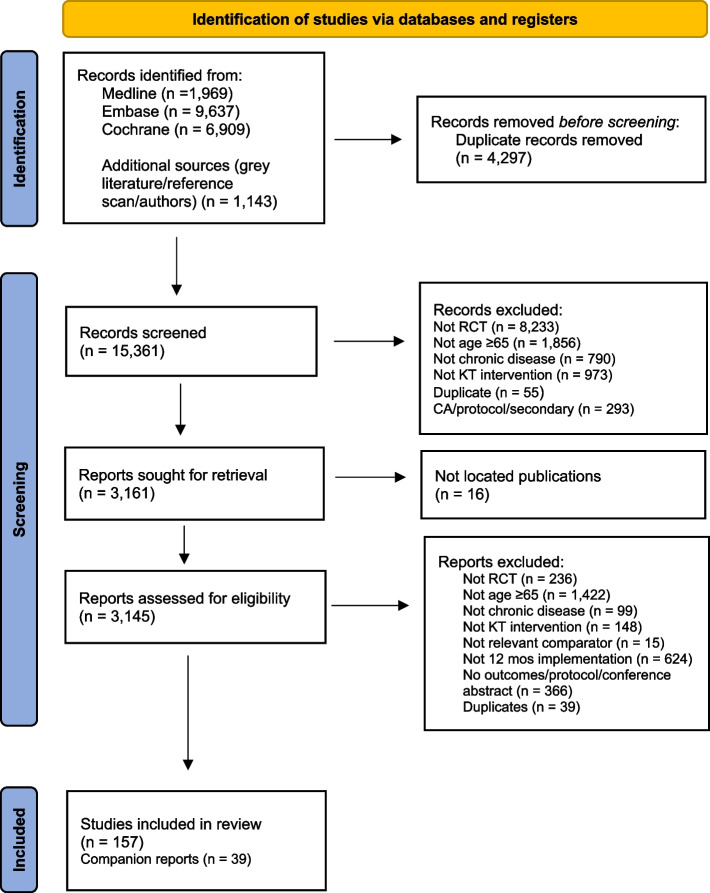
PRISMA flow diagram for identification of eligible studies. Abbreviations: CA conference abstract, KT knowledge translation, mos months, RCT randomised controlled trial. From [19]

Overall, 157 RCTs (973,074 participants overall [961,745 patients, 5540 caregivers and 5789 providers]) and 39 companion reports were included, after screening 15,361 citations and 3145 full-text articles (Fig. 1). Of the included studies, one was written in non-English language, that was in Chinese [61]. The 157 RCTs included 110 RCTs identified from literature search, 27 RCTs from reference scanning, 19 from other reference scanning in related reviews, protocols, and conference abstracts, and one study from contacted authors (Additional file 1: Appendix 8). Of the 157 RCTs, 51 were cluster-RCTs. A total of 66 of the 197 contacted authors responded to our emails, and 36 provided additional data for analysis.

### Study, patient and intervention characteristics

The included RCTs were published between 1995 and 2020, with most published between 2011 and 2015 (36.9%; Table 1). The studies were largely conducted at multiple sites (82.2%), mainly in Europe (43.9%) and North America (39.5%). The most frequent settings included clinics (38.9%) or home (37.6%), and RCT overall average duration was 36 months (range 12 to 120 months). Most studies were funded through publicly funded or government grants (53.5%), followed by mixed funding (22.9%; Additional file 1: Appendix 9 [43–47, 49, 50, 52–59, 61–200]).

**Table 1:** Summary of study characteristics

**Study characteristic**	No. (%) of RCTs (*N* = 157)
Year of publication	
1995–2000	10 (6.4)
2001–2005	25 (15.9)
2006–2010	33 (21.0)
2011–2015	58 (36.9)
2016–2020	31 (19.7)
Continent	
Europe^a^	69 (43.9)
Australia/New Zealand	15 (9.6)
North America	62 (39.5)
Asia	9 (5.7)
South America	1 (0.6)
Multi-continent	1 (0.6)
Site	
Multi-center	129 (82.2)
Single center	24 (15.3)
Not reported	4 (2.5)
Number of arms	
Two arms	145 (92.4)
Three arms	11 (7)
Four arms	1 (0.6)
Settings^b^	
Clinic	61 (38.9)
Home	59 (37.6)
Hospital	5 (3.2)
Community	5 (3.2)
Long-term care	1 (0.6)
Not reported^c^	70 (44.6)
Not applicable^d^	37 (23.6)
Sample size (no. of patients)	
40–200	50 (31.8)
201–500	49 (31.2)
501–1000	16 (10.2)
1001–2000	21 (13.4)
> 2000	19 (12.1)
Not involving patients^e^	2 (1.3)
Sample size (no. of caregivers)	
60–100	6 (3.8)
101–300	9 (5.7)
> 300	5 (3.2)
Not involving caregivers	137 (87.3)
Sample size (no. of providers)	
9–100	17 (10.8)
101–200	8 (5.1)
201–900	8 (5.1)
Not involving providers	124 (79)
Study duration with follow up	
12–19 months	14 (8.9)
20–29 months	23 (14.6)
30–39 months	43 (27.4)
40–49 months	25 (15.9)
> 50 months	13 (8.3)
Not reported	39 (24.8)
Funding	
Government	84 (53.5)
Mixed^f^	36 (22.9)
Commercial	8 (5.1)
Research organization	8 (5.1)
Voluntary organization	5 (3.2)
Charitable trust	4 (2.5)
Not reported	12 (7.6)

^a^This includes multi-country studies that were all within Europe

^b^Settings refer to the place of delivery of KT interventions. The number of RCTs exceeds 283 and percent total more than 100% because many studies involved multiple settings. A clinic covers primary or specialty care, hospital covers acute care and community-based health care covers range of primary prevention (including public health) and primary care services within the community (e.g. health promotion and disease prevention, diagnosis/treatment/management of chronic and episodic diseases, rehabilitation support)

^c^No reported setting for one or all study arms

^d^Not applicable refers to studies that have healthcare provider or caregiver KT intervention without a clinical component

^e^Two studies involving healthcare providers only

^f^Mixed refers to funding from both industry and governmental organizations/non-governmental organization/research/voluntary

The mean patient age in our review and within the included studies ranged between 65 and 85 years, with cardiovascular diseases being the most frequently reported chronic disease (40.1%; Table 2, Additional file 1: Appendix 10 [19, 43–50, 52–59, 61–170, 172–201]).

**Table 2:** Summary of patient characteristics

Patient characteristic	No. (%) of randomized clinical trials (*N* = 157)
Age, mean, years	
65.0–67.9	38 (24.2)
68.0–74.9	69 (43.9)
75.0–85.0	36 (22.9)
Age, median, years	
65.4–83.0	7 (4.5)
Age, range, years	
60.0–94.0^a^	1 (0.6)
65.0–79.0	1 (0.6)
Not reported	5 (3.2)
% Women	
0–49.9	47 (29.9)
50–100	49 (31.2)
Not reported	61 (38.9)
Chronic diseases^b^	
Cardiovascular	63 (40.1)
Respiratory	23 (14.6)
Multimorbidity	21 (13.4)
Neurological	20 (12.7)
Diabetes	15 (9.6)
Mental illness	6 (3.8)
Musculoskeletal disorders	4 (2.5)
Hypertension	3 (1.9)
Frailty	1 (0.6)
Chronic kidney disease	1 (0.6)
Co-morbidities reported^c^	
Yes	93 (59.2)
No	52 (33.1)
Unclear/not reported	12 (7.6)
Comorbidity score reported	
No	111 (70.7)
Yes	46 (29.3)

^a^Study reports on stratified age groups (60–74,$\ge$ 75 years). We included only the $\ge$ 75 age data in this paper

^b^The primary condition that is being treated/managed in the trial (e.g. hypertension)

^c^Comorbidities are additional diseases already existing or which occurred during the study that the individuals have along with a primary chronic disease[202, 203]

Overall, 327 KT interventions were identified across all study arms (Additional file 1: Appendix 11), which focused primarily on patients only (42.8%), healthcare providers only (20.8%) and both patients and healthcare providers (11%; Table 3). Most KT interventions were single interventions (42.5%) and were not tailored to end-user type (Table 3). The KT intervention delivery method was not reported in many studies (39.1%), but when reported was frequently in-person (26.3%). Across the 157 RCTs, instruction on performing a behaviour and education targeting patients/caregivers were the most frequently reported BCT and EPOC components (Table 4).

**Table 3:** Summary of KT intervention characteristics across study arms

**KT** i**ntervention** c**haracteristics**	No. (%) per study arms in included RCTs (***N*** = 327)
**Group target**	
Patients	140 (42.8)
Healthcare providers	68 (20.8)
Patients and healthcare providers	36 (11)
Caregivers	16 (4.9)
Patients and caregivers	10 (3.1)
Patients, caregivers and healthcare providers	4 (1.2)
Caregivers and healthcare providers	1 (0.3)
Not targeted population reported	52 (15.9)
**KT intervention complexity**	
Single	139 (42.5)
Multifactorial^a^	60 (18.3)
Multiple^a^	52 (15.9)
Not applicable^c^	42 (12.8)
Not reported	34 (10.4)
**KT intervention delivery**	
In-person	86 (26.3)
Indirect^b^	23 (7)
In-person and over telephone	21 (6.4)
In-person or telephone	9 (2.8)
In-person and telemonitoring	2 (0.6)
Telephone	2 (0.6)
Not applicable^c^	56 (17.1)
Not reported	128 (39.1)
**KT intervention duration**	
12–14.9 months	119 (36.4)
15–20.9 months	26 (8)
21–36 months	36 (11)
Not applicable^c^	111 (33.9)
Not reported	35 (10.7)
**Provider of KT intervention**	
Physician and/or nurse alone	110 (33.6)
Physician/nurse + clinical staff	41 (12.5)
Clinical staff	18 (5.5)
Non-clinical staff	8 (2.4)
Physician/nurse + non-clinical staff	1 (0.3)
Not applicable^d^	99 (30.2)
Not reported	50 (15.3)
**Tailoring of KT intervention**	
Not tailored intervention	190 (58.1)
Tailored intervention	60 (18.3)
Not applicable^e^	78 (23.9)

^a^‘Multiple’ refers to multi-component interventions, where every patient received the same, fixed set of intervention components, whereas and ‘multifactorial’ refers to different sets of intervention components that the patients received, which were tailored to their clinical profile

^b^Indirect delivery refers to interventions not delivered face-to-face, such as home exercise, medication and self-management

^c^Not applicable refers to arms that are control group, not receiving a KT intervention

^d^Not applicable refers to providers delivering KT interventions, which may include treatment arms targeting patients without a clinical component (e.g. receiving educational material, self-management), arms targeting healthcare workers, or arms targeting caregivers. Some interventions without a provider are tailored (e.g. self-management, medication) to while others are not tailored (e.g. web-based education) to a patient’s needs

^e^Not applicable refers to all arms with healthcare and caregiver population, or all arms targeting patients but without a clinical component (i.e. only have a KT intervention, such as education material)

**Table 4:** Summary of KT intervention behaviour change characteristics across studies

KT intervention characteristics	No. (%) of randomized clinical trials (*N* = 157)
**BCT**^a^** component (all not tailored) as part of KT intervention (*intervention target*)**	
Instruction on how to perform a behaviour (*patient*)	80 (50.9)
Restructuring the social environment (*patient*)	67 (42.7)
Instruction on how to perform a behaviour (*healthcare provider*)	51 (32.5)
Goal setting (outcomes) (*patient*)	31 (19.7)
Adding objects to the environment (*patient*)	24 (15.3)
Prompts/cues (*healthcare provider*)	22 (14)
Self-monitoring of outcome(s) of behaviour (*patient*)	22 (14)
Problem solving (*patient*)	22 (14)
Umbrella term—Patient education (*patient*)	20 (12.7)
Restructuring the social environment (*healthcare provider*)	20 (12.7)
Goal setting (behaviour) (*patient*)	20 (12.7)
Prompts/cues (*patient*)	19 (12.1)
Credible source (*healthcare provider*)	16 (10.2)
**EPOC**^b^** component as part of KT intervention (*intervention target*)**	
Patient education^**c**^ *(patients/caregivers)*	110 (70.1)
Promotion of self-management *(patients/caregivers)*	94 (59.9)
Case management *(patients/caregivers)*	88 (56.1)
Staff education *(healthcare providers)*^c^	74 (47.1)
Team changes *(healthcare providers)*	51 (32.5)
Facilitated relay of information *(healthcare providers)*	37 (23.6)
Patient reminders *(patients/caregivers)*	22 (14.0)
Audit and feedback *(healthcare providers)*	20 (12.7)
Electronic patient registry *(healthcare providers)*	20 (12.7)
Motivational interview *(patients/caregivers)*	20 (12.7)

^a^Behaviour change techniques (BCT) taxonomy-based coding

^b^Effective Practice and Organization of Care (EPOC) taxonomy-based coding

^c^Staff education and patient education was used as part of control/usual care arms in 24 and 23 studies, respectively

### Within-study risk of bias and across-study reporting bias

Within-study bias appraisal suggested that low ROB was present for 105 (67%) RCTs for random sequence generation, 63 (40%) RCTs for allocation concealment, 121 (77%) RCTs with incomplete outcome data and 119 (76%) RCTs with ‘other’ bias. Participant and personnel blinding and outcome assessment were judged at high ROB in 121 (77%) and 68 (43%) RCTs, respectively. Selective reporting was of unclear ROB in 74 (47%) RCTs (Additional file 1: Appendix 12 [43–50, 52–59, 61–65, 67–108, 110–201]).

Reporting bias assessment across studies using Egger’s test for each outcome and measurement scale separately suggested no evidence of publication bias or small-study effects (Additional file 1: Appendix 13).

### Results of syntheses

#### Sustainability, fidelity, adherence of KT interventions for CDM practice

**Fig. 2:**
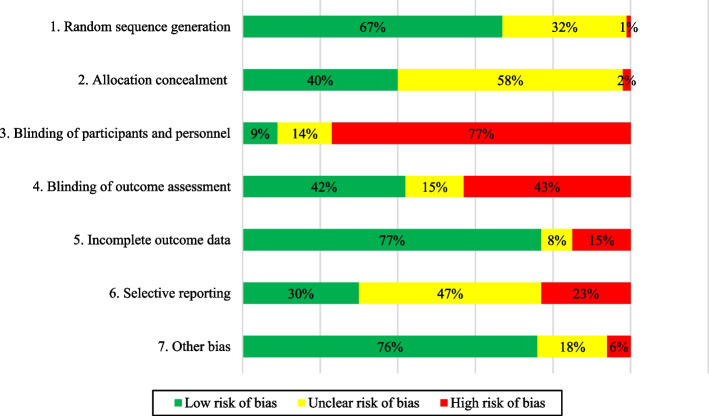
Cochrane Effective Practice and Organisation of Care (EPOC) risk of bias summary results (*n* = 157 RCTs)

**Fig. 3:**
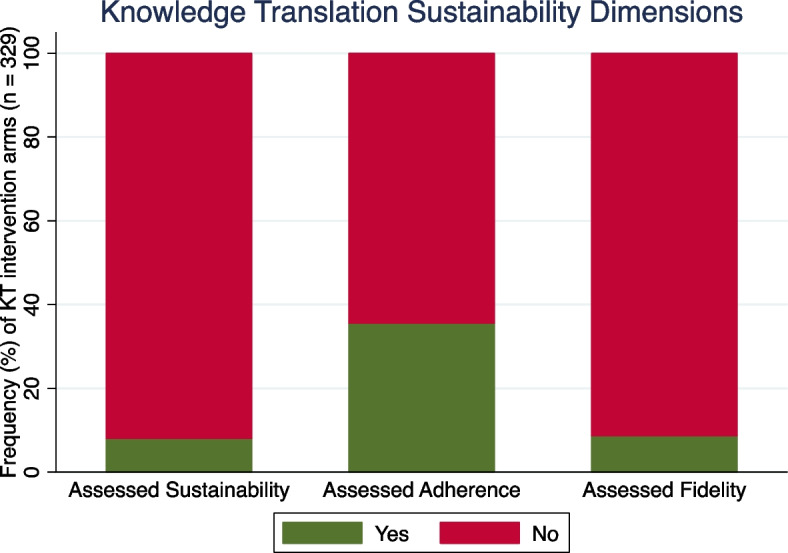
Stacked bar plot of knowledge translation (KT) sustainability dimensions

**Fig. 4:**
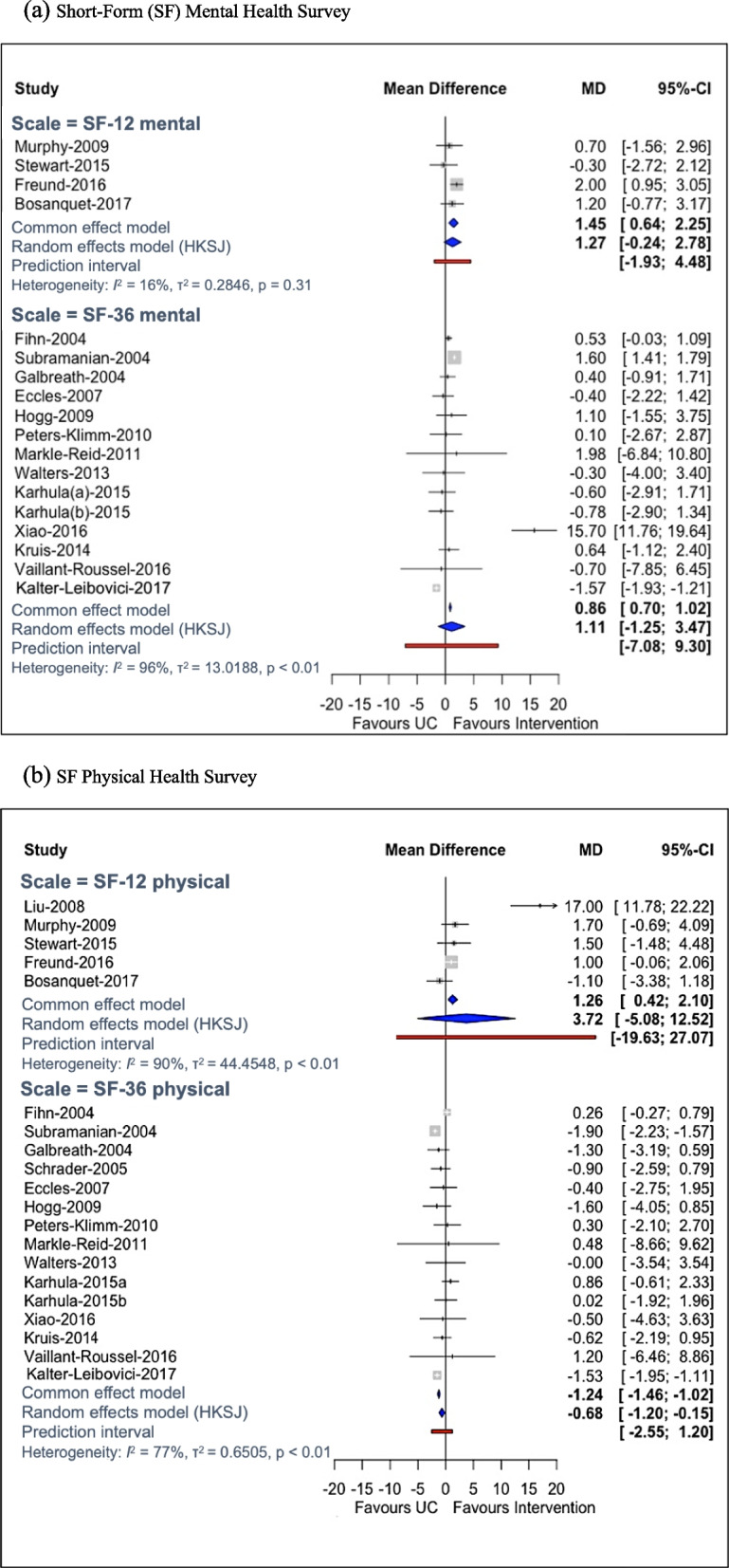

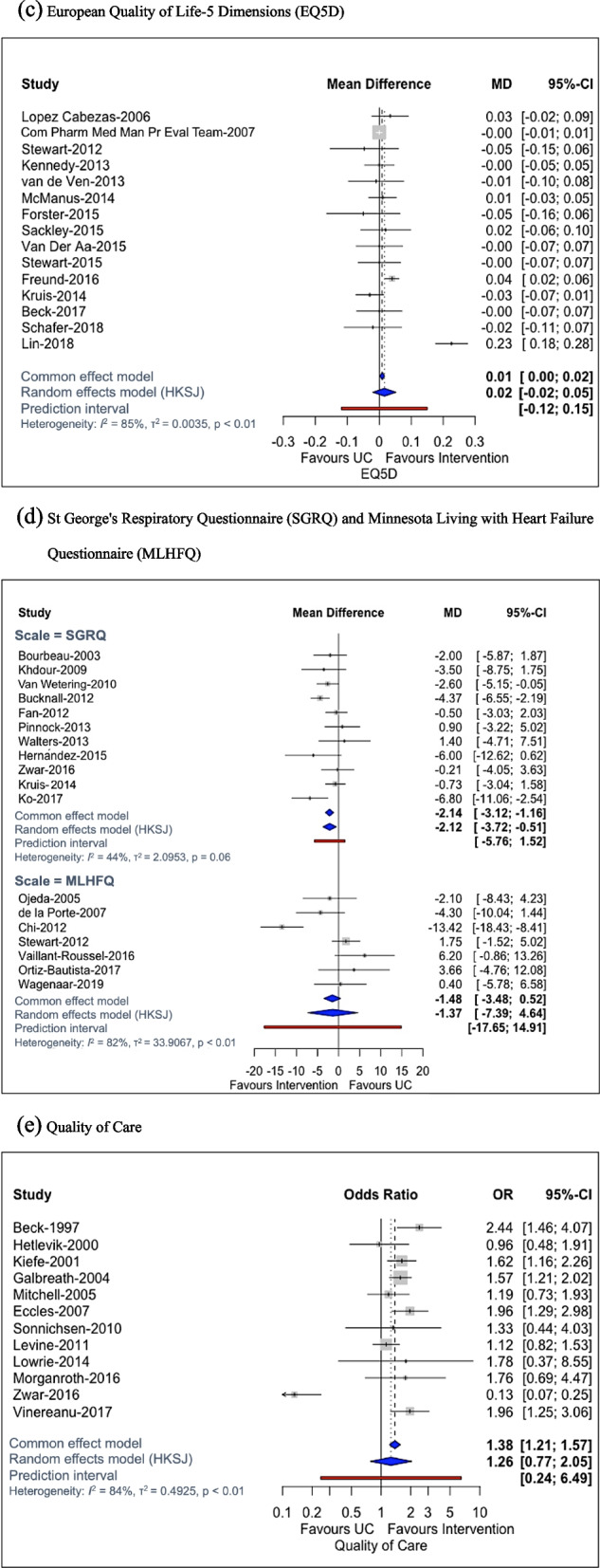
Forest plots for quality of life (**a**, **b**, **c** and **d**) and care outcomes (**e**). **a** Short-Form (SF) Mental Health Survey. **b** SF Physical Health Survey. **c** European Quality of Life-5 Dimensions (EQ-5D). **d** St George’s Respiratory Questionnaire (SGRQ) and Minnesota Living with Heart Failure Questionnaire (MLHFQ). **e** Quality of care. CI confidence interval, EQ5D European Quality of Life-5 Dimensions, HK Hartung–Knapp–Sidik–Jonkman method, MD mean difference, MLHFQ Minnesota Living with Heart Failure Questionnaire, OR odds ratio, SF Short-Form, SGRQ St George’s Respiratory Questionnaire, UC usual care

Overall, 157 RCTs reported on the primary outcome of sustained implementation of KT intervention for CDM practice. Of these, studies used different terms for sustainability dimensions: 14 studies were described by the authors as assessing sustainability in 25 (8.1%) interventions, 67 studies were described as assessing adherence in 115 (35.6%) interventions and 19 studies were described as assessing fidelity in 27 (8.7%) of the total 327 interventions (Figs. 2, 3 and 4, Additional file 1: Appendix 11). Of the 14 studies, five studies described adherence. Of 67 studies, five were also described by authors as assessing sustainability and 12 described as assessing fidelity. No study reported on all three dimensions. The 36.4% of the 327 KT interventions, representing most of the identified KT interventions, had a duration up to 15 months (Table 3).

### Healthcare outcomes with meta-analysis—quality of life (QOL)

QOL was described in 50 studies reporting seven different measurement scales and 49 different interventions, including usual care. Below we present the results of each scale informed by at least 10 studies separately, whereas in Additional file 1: Appendix 14 [103, 137] we show the results with < 10 studies. The individual study results are reported in Additional file 1: Appendix 15 [44, 46, 49, 50, 62, 63, 65, 66, 72, 73, 77, 78, 83, 88, 94, 97, 100, 102–104, 111–113, 123, 124, 126, 128, 129, 134, 136–139, 143, 147, 151, 153, 155–157, 159, 161–163, 170, 176, 177, 180–183, 187–192, 194, 198–200].

#### Short-form (SF) mental health survey

The 36 item Short-Form mental (SF-36 mental) showed that KT interventions improved QOL compared to usual care, but with imprecise effect estimate and high between-study heterogeneity (MD 1.11, 95% CI [− 1.25, 3.47]; 14 RCTs; 16 interventions plus usual care, 5876 participants; *I*^2^ = 96%, *τ* = 3.60; range of longer follow-up across studies 12–24 months; Additional file 1: Appendix 16). Excluding an outlier from SF-36 mental (MD 0.12, 95% CI [− 0.52, 0.75]; *I*^2^ = 95%, *τ* = 0.98), no differences were observed between groups [199].

For concomitant CDM therapies, results suggested that KT interventions did not improve QOL, but were imprecise (MD − 1.08, 95% CI [− 2.29, 0.12], 4 RCTs, *I*^2^ = 8%, *τ* = 0.59; Additional file 1: Appendices 16–17 [202, 203]). Results were also imprecise in sensitivity analyses restricting to studies with low ROB due to attrition and selective reporting and history of prescription use and to studies with up to 80% male participants (Additional file 1: Appendix 18 [46, 88, 136, 137, 155, 181, 199, 200]). Results showed no differences when a different number of chronic diseases or comorbidities were included or for different duration of KT sustainability (12 months vs > 12 months). A home setting was associated with the highest KT intervention effect among all groups, yet with wide uncertainty and heterogeneity (MD 3.44, 95% CI [− 5.26, 12.13], 5 RCTs, *I*^2^ = 95%, *τ* = 6.78). Meta-regression suggested that publication year and mean participant age after excluding an outlier had no important impact on the KT intervention effect (Additional file 1: Appendix 19). Baseline low QOL was an important factor for higher KT intervention efficacy, but results were informed by 10 RCTs and driven by an outlier [199].

#### Short-Form (SF) physical health survey

The SF-36 physical scale meta-analysis suggested that KT interventions were not associated with improvement in QOL, yet the 95% PI suggested that new evidence may change results (15 RCTs, 5678 participants, 14 interventions plus usual care; MD − 0.68, 95% CI [− 1.20, − 0.15], 95% PI [− 2.55, 1.98]; *I*^2^ = 77%, *τ* = 0.81; range of longer follow-up across studies 12–24 months). Results were in agreement when excluding studies with imputed SDs.

Inconclusive results were provided with sensitivity analyses restricting to studies with concomitant CDM therapies; these results aligned with the overall meta-analysis and according to PIs, restricting to studies with up to 80% male, low ROB due to attrition and selective reporting. History of patient prescription use studies suggested that KT interventions do not improve QOL (MD − 1.41, 95% CI [− 1.81, − 1.02], 8 RCTs, *I*^2^ = 0%, *τ* = 0.29). No differences in results were observed when a different number of comorbidities were included and when the follow-up time changed from 12 months to longer. For studies in the home setting, usual care was better (MD − 1.51, 95% CI [− 1.91, − 1.10], five RCTs, *I*^2^ = 0%, *τ* = 0.00). Similarly, 13 RCTs favoured usual care when a single chronic disease was present (MD − 0.81, 95% CI [− 1.38, − 0.24], *I*^2^ = 50%, *τ* = 0.76). Meta-regression suggested that publication year, QOL baseline, and mean participant age had no significant impact on the magnitude of KT intervention effect (Additional file 1: Appendix 19).

#### European quality of life-5 dimensions (EQ-5D)

The EQ-5D scale was assessed in 15 studies of 15 interventions plus usual care (6628 participants). KT interventions only marginally improved QOL, but the result was imprecise (MD 0.02, 95% CI [− 0.02, 0.05]; *I*^2^ = 85%, *τ* = 0.06; range of longer follow-up across studies 12–24 months; all studies included male participant proportions < 80%). Results were insignificant (MD 0.01, 95% CI [− 0.01, 0.02]; *I*^2^ = 25%, *τ* = 0.02) with an excluded outlier [61].

Restricting to studies with low ROB due to attrition and selective reporting, history of prescription use and concomitant CDM therapy, results suggested no clear differences between KT interventions and usual care. Similarly, no differences were observed among the different subgroups of time in KT intervention sustainability, study settings, number of chronic diseases and comorbidities. Publication year, QOL baseline and mean participant age did not impact KT intervention effect on QOL (Additional file 1: Appendix 19).

#### St George's Respiratory Questionnaire (SGRQ)

The SGRQ scale was reported in 12 studies of nine interventions plus usual care. Meta-analysis of 11 studies (2893 participants) comparing any intervention vs usual care showed that KT interventions improved QOL (MD − 2.12, 95% CI [− 3.72, − 0.51]; *I*^2^ = 44%, *τ* = 1.45; range of longer follow-up across studies 12–24 months, single chronic disease across all studies).

Results were in agreement with primary meta-analysis when restricting to studies with up to 80% male participant proportions, a history of prescription use and concomitant CDM therapy. Similar results were observed for sensitivity analysis restricting to studies with low ROB due to attrition and selective reporting, yet KT intervention effects were imprecise (Additional file 1: Appendix 18 [46, 88, 136, 137, 155, 181, 199, 200]). No major differences were observed across subgroups of a different number of comorbidities, time in sustainability of KT interventions and settings. Publication year did not impact the KT intervention effect. The effect increased with mean participant age, suggesting that KT interventions improve QOL more effectively in older people (regression coefficient: MD =  − 0.60, 95% CI [− 1.15, − 0.06]; *I*^2^ = 14%, *τ* = 0.74; Additional file 1: Appendix 19).

### Quality of care (QOC)

QOC was reported in 14 RCTs comparing 16 interventions plus usual care. Meta-analysis of 12 RCTs (5271 participants) comparing any intervention vs usual care showed that KT interventions improved QOC; nonetheless, this result was surrounded with high uncertainty and between-study heterogeneity (OR 1.26, 95%CI [0.77, 2.05]; *I*^2^ = 84%, *τ* = 0.70; range of longer follow-up across studies 12–18 months). Excluding an outlier [200], results suggested KT interventions improved statistically significantly QOC compared to usual care (OR 1.55, 95% CI [1.29, 1.85]; *I*^2^ = 21%, *τ* = 0.15). The combination of team, case management and patient education interventions was associated with the largest effect compared with usual care, but evidence derived from a single study (OR 2.44, 95% CI [1.46, 4.07]) [65].

Results agreed with primary meta-analysis in studies with low ROB due to selective reporting but were imprecise when restricting to studies with up to 80% male participants, low ROB due to attrition and history of prescription use. No major differences were observed across subgroups of study settings and time in sustainability of KT interventions (Additional file 1: Appendices 16–17 [202, 203]). The number of chronic diseases statistically significantly modified the QOC effect of KT interventions vs usual care (*p* = 0.04), but a single study [65] informed the group of at least one chronic disease vs the 11 RCTs in the one chronic disease group (one or more chronic diseases: OR 2.44, 95% CI [1.46, 4.07], one RCT; one chronic disease: OR 1.18, 95% CI [0.70, 1.98], 11 RCTs, *I*^2^ = 85%, *τ* = 0.71). QOC improved with KT interventions when four or more comorbidities were included (four comorbidities: OR 1.62, 95% CI (1.16, 2.26), one RCT; five or more comorbidities: OR 1.63, 95% CI (1.12, 2.37), 5 RCTs, *I*^2^ = 52%, *τ* = 0.23). KT interventions improved QOC with concomitant CDM therapies, but results may change when a new study becomes available (OR 1.94, 95% CI (1.45, 2.59), 95% PI (0.29, 12.77), 3 RCTs, *I*^2^ = 0%, *τ* = 0.00). Publication year and QOC baseline did not impact QOC (Additional file 1: Appendix 19).

## Discussion

Sustainability of KT interventions is critical to ensuring long-term, high QOL and consistent care for patients. This is very important in older adults with many chronic diseases. Overall, few trials evaluated the dimensions of sustainability of any KT intervention with data points on assessing adherence being the most frequently reported dimension. For trials that reported on QOL, sustained KT interventions were on average helpful. But, evidence was imprecise at improving CDM intervention outcomes. Our results should be interpreted with caution, since individual study effects were small, imprecise and heterogeneous. In most studies, there was a high risk of bias detected for participant and personnel blinding and outcome assessment. Also, KT interventions improved on average QOC, but uncertainty around the point estimate was high. PIs suggested that results were robust with low heterogeneity when an outlier was excluded. Varied KT intervention effect may be explained by the number of chronic diseases and comorbidities that are present. Efficacy tends to improve as the number of chronic diseases and comorbidities accumulate, with concomitant CDM therapies, history of prescription use and in older adults. Also, we expect that people with a greater number and severity of complex conditions may require different doses and combinations of interventions. However, there was insufficient evidence to make definite conclusions or explore the heterogeneity in varying population and intervention characteristics that could modify the intervention effect (e.g. dose or duration).

Our findings showed that long-term, maintained implementation of KT interventions was rarely defined and infrequently assessed, suggesting fundamental gaps in knowledge. This finding aligns with the conceptual analysis done by Proctor and colleagues [6], which described sustainability as one of the most significant KT research gaps. Similar findings were also observed by others given the evolving nature of healthcare. Specifically, there is overlap across sustainability, adaptation and fidelity concepts [204], and sustainability is a dynamic concept with anticipated adaptation of KT interventions [10].

Our 2016 scoping review on the sustainability of KT interventions in CDM provided an overview of all available studies, irrespective of their study design, and described their results narratively. In the scoping review, we identified 62 studies assessing sustainability of 13 KT interventions; most studies focused on patient-level interventions [14]. In the present systematic review and in contrast to the scoping review, we assessed a more focused research question. We examined the impact of sustainable KT interventions on health outcomes, included RCTs, and performed a meta-analysis of the RCT findings. In this systematic review, we found substantial publication growth, and while most interventions were similarly intended for patients, they were not tailored for patient use. Stirman and colleagues identified 125 studies in their systematic review of public health and clinical intervention sustainability; half were quantitative studies and few reported rigorous evaluation methods [7]. The authors noted a limitation that there is insufficient intervention or outcome details to inform what interventions are effective in which contexts [7].

Two frequent KT challenges in the majority of studies included in this review are a lack of a clear definition of sustainability and the scarcity of evidence assessing QOL and QOC in KT interventions. We defined KT sustainability in this study as clinical and KT interventions continuing to be delivered beyond a certain period of time. Ideally, sustainability studies should specify whether the relevant outcomes are sustained, which is difficult to report given the short duration of grant funding. Researchers and implementers should consider other sustainability aspects, including capacity to sustain implementation. Our findings can be used by knowledge users (e.g. patients, clinicians, policy-makers) regarding the sustainability of KT interventions for CDM. Initial implementation strategies may need to be modified over-time to facilitate the intervention’s sustainability, as inducing behavioural changes in patients for extended periods of time may be difficult.

Prolonged implementation of effective clinical CDM interventions through sustainable KT interventions has the potential to optimise QOL and QOC in older adults with chronic diseases. More studies are necessary to assess the efficacy of individual KT interventions and their separate components in a network meta-analysis [18]. Future work could build on our study by addressing this research gap and relevant KT intervention costs. We anticipate that these results will help to explore sustainable KT interventions development for CDM in older adults and outline how to tailor interventions. In particular, our unique review provides a more granular look at KT intervention components and behaviour change strategies.

Strengths of our study include that we followed the Cochrane Handbook methods for systematic reviews [26]. Reviewers worked in pairs and independently for screening, data abstraction and risk of bias appraisal. We reported the results using the PRISMA 2020 statement [19]. To our knowledge, this is the first study assessing the KT intervention efficacy in a systematic review with meta-analysis of RCTs. We used novel approaches to engage knowledge users and integrate their views and values in this research [4]. We used different taxonomies (EPOC and BCT) to code KT interventions, allowing researchers to use our results to build their interventions to optimise future studies [23].

Our study has some limitations to be considered. First, due to the small number of studies, we were unable to compare the efficacy of different KT interventions. High heterogeneity might be due to varied KT interventions combined in a single group. Initially, we aimed to perform a network meta-analysis to compare multiple KT interventions and produce a ranked order of their KT sustainability efficacy; however, the available evidence did not permit this. Based on the network meta-analysis results, we planned to perform an economic analysis of the interventions identified as effective. Moving forward, we plan to update our systematic review and conduct a network meta-analysis to examine the impact of different sustained KT interventions in older adults with comorbid conditions and determine which approaches are most successful and cost-effective. We will explore how different KT intervention types are linked to CDM practice. Second, the scarcity of available data is a limitation in that many KT interventions were informed by only a few studies and patients. This could affect our ability to detect differences in effects due to reduced statistical power. Also, demographic variables that may explain heterogeneity, such as age categories, living with or without a partner, were not available in the original studies. Third, our literature search is about 3 years old and new relevant studies may be available [205]. However, institutional COVID-19 lockdowns, remote work and logistical difficulties in coordinating a geographically dispersed team have resulted in extended time taken to gather, analyze, organize and present this data—excessive financial cost and lost personnel make updating this review non-feasible at present.

## Conclusions

Detailed assessment of KT intervention sustainability and understanding which are the most effective intervention components remain important research gaps. The overall efficacy of KT interventions regarding supporting a better QOL and QOC remains uncertain. Our results should be interpreted with caution due to small, imprecise and heterogeneous observed study effects with high risk of bias in participant and personnel blinding and outcome assessment. Also, KT intervention efficacy may vary depending on the intervention type, number of chronic diseases, comorbidities and participant age, among other effect modifiers. For example, the number of chronic diseases and patient comorbidities may account for varying KT intervention effect, with a tendency to observe improved KT intervention efficacy as health issues accumulated. However, it is important to note that the relationship between these factors and KT intervention efficacy is complex and requires careful interpretation. Addressing specific outcome effect modifiers can be exploited by tailoring KT interventions in future studies.

### Supplementary Information

**Additional file 1: Appendix 1.** Systematic Review Methods. **Appendix 2.** Protocol Deviations Summary Sheet. **Appendix 3.** Delphi Results. **Appendix 4.** KT sustainability outcome definitions. **Appendix 5.** Search Strategy for MEDLINE. **Appendix 6.** Coding Guide for KT Intervention Components Using EPOC Taxonomy Coding. **Appendix 7.** Coding Guides for Clinical Intervention Components using BCT coding. **Appendix 8.** List of Included Studies. **Appendix 9.** Individual Study Characteristics. **Appendix 10.** Individual Patient Characteristics. **Appendix 11.** Sustainability of KT Interventions Summarized Results. **Appendix 12.** Cochrane Effective Practice and Organisation of Care (EPOC) Risk of Bias Results. **Appendix 13.** Contour-Enhanced Funnel Plots. **Appendix 14.** Additional Analysis Results. **Appendix 15.** Individual Study Results. **Appendix 16.** Meta-analysis Results of All Interventions vs Usual Care. **Appendix 17.** Subgroup Analyses of All KT Interventions vs Usual Care. **Appendix 18.** Sensitivity Analyses of All KT Interventions vs Usual Care. **Appendix 19.** Meta-regression for Each Outcome/Scale Comparing Any KT Intervention vs Usual Care.

## Data Availability

The datasets supporting the conclusions of this article are included within the article and its additional file. The full list of excluded studies from this review at level 1 (titles/abstracts) or level 2 (full-texts) will be made available upon request.

## Abbreviations

BCT: Behaviour change technique
CDM: Chronic disease management
CI: Confidence interval
CIHR: Canadian Institutes of Health Research
EPOC: Cochrane Effective Practice and Organisation of Care
EQ-5D: European Quality of Life-5 Dimensions
GP: General practitioner
KT: Knowledge translation
MD: Mean difference
MLHFQ: Minnesota Living with Heart Failure Questionnaire
OR: Odds ratio
PI: Prediction intervals
PRESS: Peer Review of Electronic Search Strategies
QOC: Quality of care
QOL: Quality of life
RCTs: Randomized controlled trials
ROB: Risk of bias
SD: Standard deviation
SF: Short-form
SGRQ: St. George's Respiratory Questionnaire
