# Supplementary material for: Efficacy of sustained knowledge translation (KT) interventions in chronic disease management in older adults: systematic review and meta-analysis of complex interventions
Source: BMC Med. 2023 Jul 24;21:269. doi: 10.1186/s12916-023-02966-9 (PMC10367354; doi:10.1186/s12916-023-02966-9)
Supplement: Supplementary file 1 — Additional file 1: Appendix 1. Systematic Review Methods. Appendix 2. Protocol Deviations Summary Sheet. Appendix 3. Delphi Results. Appendix 4. KT sustainability outcome definitions. Appendix 5. Search Strategy for MEDLINE. Appendix 6. Coding Guide for KT Intervention Components Using EPOC Taxonomy Coding. Appendix 7. Coding Guides for Clinical Intervention Components using BCT coding. Appendix 8. List of Included Studies. Appendix 9. Individual Study Characteristics. Appendix 10. Individual Patient Characteristics. Appendix 11. Sustainability of KT Interventions Summarized Results. Appendix 12. Cochrane Effective Practice and Organisation of Care (EPOC) Risk of Bias Results. Appendix 13. Contour-Enhanced Funnel Plots. Appendix 14. Additional Analysis Results. Appendix 15. Individual Study Results. Appendix 16. Meta-analysis Results of All Interventions vs Usual Care. Appendix 17. Subgroup Analyses of All KT Interventions vs Usual Care. Appendix 18. Sensitivity Analyses of All KT Interventions vs Usual Care. Appendix 19. Meta-regression for Each Outcome/Scale Comparing Any KT Intervention vs Usual Care. [file 12916_2023_2966_MOESM1_ESM.pdf]

Efficacy of sustained knowledge translation (KT) interventions in chronic disease management in older adults: systematic review and meta-analysis of complex interventions

Supplementary Online Content

Appendix 1: Systematic Review Methods 2
Appendix 2: Protocol Deviations Summary Sheet 7
Appendix 3: Delphi Results 8
Appendix 4: KT sustainability outcome definitions 12
Appendix 5: Search Strategy for MEDLINE 13
Appendix 6. Coding Guide for KT Intervention Components Using EPOC Taxonomy Coding 21
Appendix 7. Coding Guides for Clinical Intervention Components using BCT coding 26
Appendix 8: List of Included Studies 36
Appendix 9. Individual Study Characteristics 54
Appendix 10: Individual Patient Characteristics 69
Appendix 11: Sustainability of KT Interventions Summarized Results 75
Appendix 12: Cochrane Effective Practice and Organisation of Care (EPOC) Risk of Bias Results 84
Appendix 13: Contour-Enhanced Funnel Plots 91
Appendix 14. Additional Analysis Results 92
Appendix 15: Individual Study Results 94
Appendix 16: Meta-analysis Results of All Interventions vs Usual Care 107
Appendix 17: Subgroup Analyses of All KT Interventions vs Usual Care 108
Appendix 18: Sensitivity Analyses of All KT Interventions vs Usual Care 120
Appendix 19: Meta-regression for Each Outcome/Scale Comparing Any KT Intervention vs Usual Care 126
References 129

Appendix 1: Systematic Review Methods

Eligibility criteria

We included randomised controlled trials (RCTs) where the target population for the CDM intervention included patients (aged 65 years and older with one or more chronic disease including non-communicable diseases, such as diabetes or dementia [1]) or their caregiver. CDM interventions were continued for 12 months or more to be eligible for inclusion. End-users of the KT intervention included patients aged 65 years and older with one or more chronic diseases, their caregivers, clinicians (all disciplines), public health officials (including medical officers of health, department chairs, programme managers), health care managers and policy-makers (including regional, state/provincial, federal).

The CDM intervention was defined as the clinical intervention (may include pharmacologic and non-pharmacologic interventions such as exercise in a patient with type 2 diabetes); CDM interventions could target any chronic condition and may target the patient, caregiver, clinician, or health system, aiming to optimise health and patient well-being. The KT intervention was defined as the strategy used to support implementation of the CDM intervention, such as a reminder system for patients to exercise or motivational interviewing for clinicians to promote patient exercise. KT interventions were classified using (1) a pre-existing taxonomy developed by the Cochrane Effective Practice and Organisation of Care (EPOC) group, and (2) the behaviour change technique (BCT) taxonomy. We included CDM interventions for any chronic condition combined with at least one KT intervention (e.g., audit and feedback, reminders, opinion leaders) targeting patients, caregivers, healthcare providers, or health systems. The comparators were other KT interventions or usual care.

We selected core outcomes through a modified Delphi agreement-building approach undertaken by 11 knowledge users, as described in our protocol (Appendix 1) [2]. The primary outcome was sustained implementation of a KT intervention for CDM beyond one year after implementation or termination of funding, and which KT interventions were used. This outcome implies that the CDM intervention study would have a duration of 12 months or longer and we determined if, and what, KT interventions were sustained during this period. Secondary outcomes were health-related or disease specific QOL and process or QOC (Appendix 2). Eligible were both published and unpublished RCTs in any language and healthcare setting (e.g., primary care, specialist care, acute or long-term care).

Search strategy

We searched the bibliographic databases MEDLINE (1946 – March 4, 2020), EMBASE (1974 – March 4, 2020), and the Cochrane Central Register of Controlled Trials databases (CENTRAL, 2020, Issue 2). A librarian (JM) developed the search strategies with input from the study team, and peer-reviewed by a second librarian using the Peer Review of Electronic Search Strategies (PRESS) checklist (Appendix 3). Reference lists of all included studies and relevant reviews were scanned. We developed a grey literature search strategy [3] to seek unpublished studies which included reviewing websites of key funding agencies and healthcare provider organisations from Australia, Canada, United Kingdom, United States, and by contacting experts in the field. These included the Agency for Healthcare Research and Quality(https://www.ahrq.gov/index.html), Evidence & Policy journal, Australia KT, Implementation Science journal, Health Evidence and KT Plus McMaster, KT Strategies database, Canadian Knowledge Transfer and Exchange Community of Practice, Canadian Stroke best practices, Ontario Institute for Cancer Research, Canadian Centre for Applied Research in Cancer Control, Canadian Kidney KT and Generation Network, Evaluation Support Scotland, and the online resource created for Behaviour Change theories for researchers, policymakers and practitioners (http://www.behaviourchangetheories.com/) through the relevant websites. In all websites, we used the search strategy: ("chronic disease" AND aged AND "randomized controlled trial") AND ("knowledge translation" AND sustain).

Selection process

After, duplicate scanning within the Endnote software, the team first conducted two calibration screening exercises of study eligibility criteria on 50 records each, among seven reviewers (48% disagreement on first pilot exercise and 20% disagreement on second pilot exercise). Next, pairs of reviewers independently screened titles and abstracts in duplicate. Disagreements (14%) were resolved through discussion. Subsequently, one pilot test of 20 full-text articles was conducted among all reviewers (24% disagreement) and the team refined the data extraction template accordingly. Thereafter, two reviewers screened remaining full-text articles independently and in duplicate. The full screening process was performed through Synthesi.SR (https://breakthroughkt.ca/login.php).

Data collection process and data items

We developed a standardized data abstraction form, and pilot tested it using a random sample of five studies. We extracted participant, intervention, and outcome level data from each study, as described in our protocol [2]. Pairs of reviewers independently abstracted data from each included study, and third review resolved discrepancies. We emailed authors for missing data or data clarifications.

As per our protocol, two reviewers (ACT, CF) with expertise in KT and research methods coded each KT intervention within the included studies independently using EPOC and BCT taxonomies to identify active components in each intervention [4-6]. KT intervention coding conflicts using EPOC (arose in 38% of the coded interventions) and BCT (arose in 57% of the coded interventions) were resolved through discussion and by a third reviewer (SES, JP, CS, PR). Based on the intervention target population, each KT intervention components were classified at: the healthcare system, healthcare provider or patient level using EPOC taxonomy (Appendix 4); and healthcare system, healthcare provider, caregiver, and patient levels using BCT taxonomy (Appendix 5)[6]. Moreover, each KT intervention component was stratified as tailored or non-tailored (using BCT taxonomy) to the audience. We used the coding structure to create intervention nodes for meta-analysis by classifying interventions according to their taxonomy categories. We captured the following binary variables (yes/no): whether sustainability of KT interventions was assessed beyond 1 year, fidelity of KT interventions was Assessed ("the consistency and quality of targeted organizational members' use of the specific innovation" [7]), and adherence (continuing all components of the intervention) of KT interventions for at least one year.

Study risk of bias and reporting bias assessment

We used the EPOC risk of bias (ROB) tool to appraise included studies [8]. Following a calibration exercise of three studies, pairs of reviewers, who worked independently, appraised each included study. Third reviewer (PR) resolved conflicts.

We used visual inspection of the contour-enhanced funnel plot and Egger's test to assess for small-study effects and reporting bias when more than ten studies were available per meta-analysis and for all interventions as a single group against usual care [9].

Effect measures and synthesis methods

We performed a descriptive analysis for the primary outcome, sustainability of KT interventions, and used frequencies and percentages for the discrete variables: sustainability, adherence, and fidelity assessment for each KT intervention.

We combined study-level data in a meta-analysis using the mean difference (MD) for continuous outcomes (i.e., QOL) and odds ratio (OR) for dichotomous outcomes (i.e., QOC) along with corresponding 95% confidence intervals (95% CI) when at least two studies were available. We combined study-level data in a meta-analysis using the mean

difference (MD) for continuous outcomes (i.e., QOL) and odds ratio (OR) for dichotomous outcomes (i.e., QOC) along with corresponding 95% confidence intervals (95% CI) when at least two studies were available. We decided to use MD for each different scale since included studies reported multiple results eligible for inclusion, i.e., effect estimates for multiple scales measuring the same outcome (e.g., Short-Form mental and Short-Form physical for QOL), to capture any differences in the results. We also preferred using the OR due to its good mathematical properties [10]. We summarized results using both EPOC and BCT coding. We combined all interventions as a single group and compared it with usual care for each outcome separately, and irrespective of EPOC and BCT coding. In case a study compared multiple KT intervention groups against usual care, we combined KT interventions in a single group as suggested in the Cochrane Handbook [11]. We analysed different measurement scales separately to allow for MD inclusion provided by the original publications, allowing combination of change scores and final values, thereby simplifying findings interpretation. For missing standard deviations (SDs) that could not be retrieved from publications or calculated from other statistics, we borrowed SDs from similar studies with the same intervention comparison. For cluster-RCTs, we used the cluster-adjusted effect estimate, or if not reported, we used the unadjusted data accounting for the design effect [11]. We used the average intraclass correlation coefficient across when not reported in a study to calculate the design effect [11].

Where study-level data were available, we performed a random-effects meta-analysis model using the inverse-variance method, since we expected the studies to be methodologically and clinically different [12]. We estimated the overall effect size and its 95% CI using the Hartung-Knapp-Sidik-Jonkman method to handle meta-analyses with few studies [13-15]. We calculated prediction intervals (PIs) for the overall effect, to capture the interval within which we expected the true intervention effect of a new study to fall. We assessed between-study heterogeneity by visual inspection of each meta-analysis forest plot, using the χ^2 test (with $p<0.10$ indicating a larger variation across studies rather than between subjects within a study), the I^2 statistic (quantifying the degree of heterogeneity), and τ^2 (estimating the between-study variance) along with its associated 95% CI [16-18]. We used the restricted maximum likelihood method to estimate τ^2 and the Q-profile approach to calculate a 95% CI for τ^2 [19, 20].

When excessive heterogeneity was detected, we checked the data for potential data abstraction errors. If no errors were identified, we performed meta-regression, subgroup, or sensitivity analyses. We explored possible heterogeneity sources using predefined sensitivity analyses: excluding studies with imputed SDs, excluding outliers as visually detected in the funnel plot, excluding studies with more than 80% male participants, restricting to studies at low ROB given the

components of incomplete outcome data and selective outcome reporting, restricting to studies reporting a history of prescription usage, and restricting to studies with concomitant CDM therapies. We combined findings using both random-effects and fixed-effect models and prioritized a random-effects model in all analyses except for those with estimated heterogeneity equal to zero and fewer than three studies. When heterogeneity was estimated to be zero, we reported the fixed-effect model estimate because in these cases the Hartung-Knapp-Sidik-Jonkman method is considered inadequate [13, 21]. When two studies were included and the estimated heterogeneity was (>0), we presented both fixed and random effects findings."[22]

We explored the influence of patient age, publication year, and QOL baseline level or care using meta-regression when meta-analysis included more than 10 studies. We performed a series of subgroup analyses to further explore sources of heterogeneity and differences in summary estimates, specifically duration of KT sustainability at 12 months against longer than 12 months, number of chronic diseases, number of comorbidities, and KT setting (e.g., home, primary care clinic, outpatient clinic, general practitioner (GP) clinic, community). All subsequent analyses were performed for comparison including all interventions vs usual care due to the dearth of studies within each individual intervention comparison. All analyses were conducted in R using the *meta* package [23].

Appendix 2: Protocol Deviations Summary Sheet

Category of deviation	Reasons for deviation	Action undertaken
Data Analysis	We were unable to estimate the individual component effects and their combinations due to insufficient power. Our taxonomy identified 15 EPOC and 78 BCT individual components and multiple combinations of these (104 for EPOC and 190 for BCT). Examples include patient/provider education, reminders, and financial incentives in EPOC and problem solving, action planning, and social support in BCT.<br>The quality-of-life outcome sustained to at least 12 months was described in 50 studies reporting seven different measurement scales and 49 different interventions (combinations of components), including usual care. This sparce evidence did not allow us to derive granular insights to fully inform the priorities of KUs.	We restricted our analysis to a meta-analysis instead of a network meta-analysis to infer on the identified individual measurement scales separately. Our review provided high-level evidence of the KT sustainability evidence base, but we plan to perform a rapid update of this systematic review and perform a network meta-analysis as originally planned.
Data Analysis	We decided to perform a frequentist approach and follow the most recent and rigorous Cochrane guidelines on conducting a pairwise meta-analysis [11, 13, 24].<br>We expect no important differences in the results between Bayesian and frequentist approaches, and we preferred to focus on model features rather than choosing between statistical frameworks [25].	We performed a frequentist analysis instead of a Bayesian approach
Economic analysis of the interventions identified to be effective	We were unable to perform an economic evaluation to compare the cost and outcome among the most effective and sustainable KT interventions, since results from a network meta-analysis were not provided.	This will be performed in a separate paper, once a rapid update of our systematic review is performed along with a network meta-analysis as planned in our protocol.<br>As per our initial plan, we will build a decision analytic model comparing effective interventions to estimate an incremental cost-effectiveness ratio.

Appendix 3: Delphi Results

Process: We followed a modified e-Delphi, where two rounds of online voting were conducted to rank outcomes using the outcomes from our previous scoping review[4]. Participants included researchers, clinicians, policymakers, caregivers, and patients, and were asked to rate importance of each outcome on a 7-point scale (ranging from 1 = least important to 7 = most important)

Results: Round 1 (10 respondents): 28 outcomes with median rank score 6 or higher shortlisted for second round of voting; Round 2 (11 respondents): The 3 top ranked outcomes selected were: Clinical outcomes (patient); Quality of life (patient); Process/quality of care (health system). Results from both rounds are presented below.

Table 1. Delphi results in round 1

Item*	Not Important (1) [N (%)]	2 [N (%)]	3 [N (%)]	Neutral (4) [N (%)]	5 [N (%)]	6 [N (%)]	Extremely Important (7) [N (%)]	Median (IQR)
Patient-level Outcomes								
**Adaptation**	0 (0%)	0 (0%)	3 (30%)	1 (10%)	**3 (30%)**	2 (20%)	0 (0%)	5 (3, 5)
**Acceptability**	0 (0%)	0 (0%)	0 (0%)	3 (30%)	0 (0%)	**4 (40%)**	2 (20%)	6 (4, 6)
**Adherence To Treatment**	0 (0%)	1 (10%)	0 (0%)	0 (0%)	1 (10%)	**3 (30%)**	4 (40%)	6 (6, 7)
**Adverse Events/Complications**	0 (0%)	0 (0%)	0 (0%)	2 (20%)	2 (20%)	**2 (20%)**	3 (30%)	6 (5, 7)
**Anxiety**	0 (0%)	0 (0%)	1 (10%)	1 (10%)	**4 (40%)**	3 (30%)	0 (0%)	5 (5, 6)
**Attitude To Treatment**	0 (0%)	2 (20%)	0 (0%)	1 (10%)	**2 (20%)**	4 (40%)	0 (0%)	5 (4, 6)
**Behavioural Disturbance**	0 (0%)	0 (0%)	0 (0%)	3 (30%)	1 (10%)	**4 (40%)**	1 (10%)	6 (4, 6)
**Clinical Outcomes**	0 (0%)	0 (0%)	0 (0%)	2 (20%)	0 (0%)	2 (20%)	**5 (50%)**	7 (6, 7)
**Disease Specific Quality Of Life**	0 (0%)	0 (0%)	0 (0%)	1 (10%)	1 (10%)	1 (10%)	**6 (60%)**	7 (6, 7)
**Functional Status**	0 (0%)	0 (0%)	0 (0%)	1 (10%)	0 (0%)	3 (30%)	**5 (50%)**	7 (6, 7)
**Health Behaviours**	0 (0%)	0 (0%)	0 (0%)	1 (10%)	3 (30%)	**1 (10%)**	4 (40%)	6 (5, 7)
**Health Related Distress**	0 (0%)	0 (0%)	1 (10%)	3 (30%)	**2 (20%)**	3 (30%)	0 (0%)	5 (4, 6)
**Health Status -Self Reported**	0 (0%)	0 (0%)	0 (0%)	2 (20%)	2 (20%)	**3 (30%)**	2 (20%)	6 (5, 6)
**Health Related Quality Of Life**	0 (0%)	0 (0%)	0 (0%)	0 (0%)	1 (10%)	3 (30%)	**5 (50%)**	7 (6, 7)
**Health Literacy/Knowledge**	0 (0%)	0 (0%)	1 (10%)	1 (10%)	**4 (40%)**	3 (30%)	0 (0%)	5 (5, 6)
**Mortality**	0 (0%)	0 (0%)	1 (10%)	1 (10%)	**3 (30%)**	0 (0%)	4 (40%)	5 (5, 7)
**Perceived Quality Of Care**	0 (0%)	0 (0%)	0 (0%)	1 (10%)	3 (30%)	**2 (20%)**	3 (30%)	6 (5, 7)
**Physical Function**	0 (0%)	0 (0%)	0 (0%)	1 (10%)	**3 (30%)**	2 (20%)	2 (20%)	5.5 (5, 6.25)

**Psychological Well-Being**	0 (0%)	0 (0%)	0 (0%)	0 (0%)	3 (30%)	**4 (40%)**	2 (20%)	6 (5, 6)
**Residence Status**	0 (0%)	1 (10%)	1 (10%)	2 (20%)	**2 (20%)**	3 (30%)	0 (0%)	5 (4, 6)
**Risk Scores**	0 (0%)	2 (20%)	0 (0%)	2 (20%)	**2 (20%)**	2 (20%)	1 (10%)	5 (4, 6)
**Patient Satisfaction**	0 (0%)	0 (0%)	0 (0%)	2 (20%)	2 (20%)	**3 (30%)**	2 (20%)	6 (5, 6)
**Self-Care Behaviour**	0 (0%)	0 (0%)	0 (0%)	2 (20%)	**4 (40%)**	2 (20%)	1 (10%)	5 (5, 6)
**Self-Efficacy**	0 (0%)	0 (0%)	0 (0%)	2 (20%)	**5 (50%)**	2 (20%)	0 (0%)	5 (5, 5)
**Social Support/Activity**	0 (0%)	0 (0%)	0 (0%)	2 (20%)	**4 (40%)**	3 (30%)	0 (0%)	5 (5, 6)
**Survival**	0 (0%)	1 (10%)	0 (0%)	1 (10%)	1 (10%)	**3 (30%)**	3 (30%)	6 (5, 7)
Caregiver-level Outcomes								
**Anxiety**	0 (0%)	0 (0%)	1 (10%)	3 (30%)	**3 (30%)**	3 (30%)	0 (0%)	5 (4, 5.75)
**Caregiver Burden**	0 (0%)	0 (0%)	0 (0%)	0 (0%)	3 (30%)	**2 (20%)**	5 (50%)	6.5 (5.25, 7)
**Caregiver Competence/Skill**	0 (0%)	1 (10%)	0 (0%)	2 (20%)	**4 (40%)**	2 (20%)	1 (10%)	5 (4.25, 5.75)
**Caregiver Stress/Distress**	0 (0%)	0 (0%)	0 (0%)	2 (20%)	2 (20%)	**3 (30%)**	3 (30%)	6 (5, 6.75)
**Caregiver Personality (Neuroticism)**	1 (10%)	0 (0%)	3 (30%)	**4 (40%)**	1 (10%)	1 (10%)	0 (0%)	4 (3, 4)
**Caregiver Preparedness**	0 (0%)	1 (10%)	1 (10%)	1 (10%)	**3 (30%)**	2 (20%)	1 (10%)	5 (4, 6)
**Caregiver Unmet Needs**	0 (0%)	1 (10%)	2 (20%)	0 (0%)	**6 (60%)**	1 (10%)	0 (0%)	5 (3.5, 5)
**Caregiver Satisfaction**	0 (0%)	1 (10%)	0 (0%)	1 (10%)	**4 (40%)**	3 (30%)	1 (10%)	5 (5, 6)
**Caregiver Social Activity**	0 (0%)	1 (10%)	1 (10%)	2 (20%)	**5 (50%)**	1 (10%)	0 (0%)	5 (4, 5)
**Caregiver Well-Being**	0 (0%)	0 (0%)	0 (0%)	0 (0%)	4 (40%)	**4 (40%)**	2 (20%)	6 (5, 6)
**Caregiver Depression/Depressive Symptoms**	0 (0%)	1 (10%)	0 (0%)	2 (20%)	**2 (20%)**	3 (30%)	2 (20%)	5.5 (4.25, 6)
**Caregiver Knowledge**	1 (10%)	0 (0%)	1 (10%)	1 (10%)	**2 (20%)**	4 (40%)	1 (10%)	5.5 (4.25, 6)
**Caregiver's Physical Health**	0 (0%)	1 (10%)	1 (10%)	2 (20%)	**3 (30%)**	2 (20%)	1 (10%)	5 (4, 5.75)
**Caregiver's Productivity Loss**	0 (0%)	1 (10%)	0 (0%)	1 (10%)	**4 (40%)**	4 (40%)	0 (0%)	5 (5, 6)
**Caregiving Quality**	0 (0%)	0 (0%)	2 (20%)	1 (10%)	**4 (40%)**	1 (10%)	2 (20%)	5 (4.25, 5.75)
**Coping Strategies**	0 (0%)	0 (0%)	0 (0%)	0 (0%)	**5 (50%)**	4 (40%)	0 (0%)	5 (5, 6)
**Decision Making Confidence**	0 (0%)	1 (10%)	0 (0%)	1 (10%)	**5 (50%)**	3 (30%)	0 (0%)	5 (5, 5.75)
**Decision-Making Skill**	0 (0%)	0 (0%)	0 (0%)	1 (10%)	3 (30%)	**5 (50%)**	0 (0%)	6 (5, 6)
**Desire To Institutionalize**	0 (0%)	1 (10%)	1 (10%)	**3 (30%)**	2 (20%)	3 (30%)	0 (0%)	4.5 (4, 5.75)

**Family Conflict**	1 (10%)	1 (10%)	0 (0%)	**3 (30%)**	2 (20%)	3 (30%)	0 (0%)	4.5 (4, 5.75)
**Health Related Quality Of Life**	0 (0%)	0 (0%)	0 (0%)	2 (20%)	**3 (30%)**	2 (20%)	3 (30%)	5.5 (5, 6.75)
**Self-Efficacy**	0 (0%)	0 (0%)	0 (0%)	1 (10%)	**6 (60%)**	2 (20%)	1 (10%)	5 (5, 5.75)
**Sense Of Mastery**	0 (0%)	1 (10%)	0 (0%)	2 (20%)	**4 (40%)**	3 (30%)	0 (0%)	5 (4.25, 5.75)
**Social Problem Solving Abilities**	1 (10%)	0 (0%)	1 (10%)	1 (10%)	**5 (50%)**	2 (20%)	0 (0%)	5 (4.25, 5)
**Social Support**	0 (0%)	0 (0%)	0 (0%)	0 (0%)	**5 (50%)**	4 (40%)	1 (10%)	5.5 (5, 6)
**Use Of Support Services**	0 (0%)	0 (0%)	2 (20%)	1 (10%)	**2 (20%)**	4 (40%)	1 (10%)	5.5 (4.25, 6)
Healthcare provider-level outcomes								
**Adherence To Guidelines**	0 (0%)	1 (10%)	0 (0%)	0 (0%)	0 (0%)	**5 (50%)**	4 (40%)	6 (6, 7)
**Process/Quality Of Care Outcomes**	0 (0%)	0 (0%)	0 (0%)	1 (10%)	1 (10%)	**3 (30%)**	5 (50%)	6.5 (6, 7)
**Prescribing Behaviour**	0 (0%)	1 (10%)	0 (0%)	1 (10%)	1 (10%)	**3 (30%)**	4 (40%)	6 (5.25, 7)
**Physician Knowledge**	0 (0%)	1 (10%)	1 (10%)	0 (0%)	1 (10%)	**5 (50%)**	2 (20%)	6 (5.25, 6)
**Implementation**	0 (0%)	0 (0%)	1 (10%)	0 (0%)	0 (0%)	**6 (60%)**	3 (30%)	6 (6, 6.75)
**Perceived Job Demands/Satisfaction/Quality**	0 (0%)	1 (10%)	0 (0%)	2 (20%)	**4 (40%)**	3 (30%)	0 (0%)	5 (4.25, 5.75)
Health system-level outcomes								
**Healthcare/Health Services Use**	0 (0%)	0 (0%)	0 (0%)	2 (20%)	2 (20%)	**3 (30%)**	3 (30%)	6 (5, 6.75)
**Hospitalization/Readmission**	0 (0%)	0 (0%)	0 (0%)	0 (0%)	1 (10%)	**6 (60%)**	3 (30%)	6 (6, 6.75)
**Institutionalization**	0 (0%)	0 (0%)	0 (0%)	1 (10%)	3 (30%)	**4 (40%)**	2 (20%)	6 (5, 6)
**Process/Implementation Outcomes**	0 (0%)	0 (0%)	0 (0%)	1 (10%)	3 (30%)	**3 (30%)**	3 (30%)	6 (5, 6.75)
**Clinical Targets**	0 (0%)	0 (0%)	1 (10%)	2 (20%)	1 (10%)	**3 (30%)**	3 (30%)	6 (4.25, 6.75)

***Item names in bold text received a median ranking >5 and will be included the round 2 survey**

Table 2. Delphi results in round 2

Outcome	Not Important (1)[N (%)]	2 [N(%)]	3 [N (%)]	Neutral (4)[N (%)]	5 [N (%)]	6 [N (%)]	Extremely Important (7)[N (%)]	Median (IQR)
**Clinical Outcomes**	0 (0%)	0 (0%)	0 (0%)	0 (0%)	0 (0%)	3 (30%)	7 (70%)	7 (6.25, 7)
**Disease Specific Quality Of Life**	0 (0%)	0 (0%)	0 (0%)	0 (0%)	0 (0%)	5 (50%)	5 (50%)	6.5 (6, 7)
**Functional Status**	0 (0%)	0 (0%)	0 (0%)	1 (10%)	0 (0%)	5 (50%)	4 (40%)	6 (6, 7)
**Health Related Quality Of Life**	0 (0%)	0 (0%)	0 (0%)	0 (0%)	1 (10%)	5 (50%)	4 (40%)	6 (6, 7)
**Process/Quality Of Care Outcomes**	0 (0%)	0 (0%)	0 (0%)	0 (0%)	1 (10%)	5 (50%)	4 (40%)	6 (6, 7)
**Implementation**	0 (0%)	0 (0%)	0 (0%)	1 (10%)	0 (0%)	6 (60%)	3 (30%)	6 (6, 6.75)
**Adherence**	0 (0%)	0 (0%)	0 (0%)	0 (0%)	2 (20%)	6 (60%)	2 (20%)	6 (6, 6)
**Adverse Events**	0 (0%)	0 (0%)	0 (0%)	0 (0%)	2 (20%)	6 (60%)	2 (20%)	6 (6, 6)
**Health Behaviours**	0 (0%)	0 (0%)	0 (0%)	0 (0%)	2 (20%)	6 (60%)	2 (20%)	6 (6, 6)
**Psychological Well-Being**	0 (0%)	0 (0%)	0 (0%)	0 (0%)	1 (10%)	7 (70%)	0 (0%)	6 (6, 6)
**Caregiver Burden**	0 (0%)	0 (0%)	0 (0%)	0 (0%)	1 (10%)	7 (70%)	2 (20%)	6 (6, 6)
**Adherence To Guidelines**	0 (0%)	0 (0%)	0 (0%)	0 (0%)	2 (20%)	7 (70%)	1 (10%)	6 (6, 6)
**Healthcare/Health Services Use**	0 (0%)	0 (0%)	0 (0%)	1 (10%)	1 (10%)	6 (60%)	2 (20%)	6 (6, 6)
**Hospitalization/Readmission**	0 (0%)	0 (0%)	0 (0%)	0 (0%)	0 (0%)	9 (90%)	1 (10%)	6 (6, 6)
**Process/Implementation Outcomes**	0 (0%)	0 (0%)	0 (0%)	0 (0%)	1 (10%)	7 (70%)	2 (20%)	6 (6, 6)
**Clinical Targets**	0 (0%)	0 (0%)	0 (0%)	1 (10%)	1 (10%)	8 (80%)	0 (0%)	6 (6, 6)
**Survival**	0 (0%)	0 (0%)	1 (10%)	0 (0%)	2 (20%)	4 (40%)	3 (30%)	6 (5.25, 6.75)
**Health Status (Self-Rated)**	0 (0%)	0 (0%)	0 (0%)	1 (10%)	2 (20%)	7 (70%)	0 (0%)	6 (5.25, 6)
**Caregiver Well-Being**	0 (0%)	0 (0%)	0 (0%)	1 (10%)	2 (20%)	7 (70%)	0 (0%)	6 (5.25, 6)
**Prescribing Behaviour**	0 (0%)	0 (0%)	1 (10%)	1 (10%)	1 (10%)	6 (60%)	1 (10%)	6 (5.25, 6)
**Physician Knowledge**	0 (0%)	0 (0%)	0 (0%)	1 (10%)	2 (20%)	6 (60%)	1 (10%)	6 (5.25, 6)
**Institutionalization**	0 (0%)	0 (0%)	0 (0%)	1 (10%)	2 (20%)	7 (70%)	0 (0%)	6 (5.25, 6)
**Behavioural Disturbance**	0 (0%)	0 (0%)	0 (0%)	2 (20%)	2 (20%)	6 (60%)	0 (0%)	6 (5, 6)
**Patient Satisfaction**	0 (0%)	0 (0%)	0 (0%)	0 (0%)	4 (40%)	4 (40%)	2 (20%)	6 (5, 6)
**Perceived Quality Of Care**	0 (0%)	0 (0%)	0 (0%)	2 (20%)	3 (30%)	5 (50%)	0 (0%)	5.5 (5, 6)
**Caregiver Stress/Distress**	0 (0%)	0 (0%)	0 (0%)	1 (10%)	4 (40%)	4 (40%)	1 (10%)	5.5 (5, 6)
**Decision-Making Skill**	0 (0%)	0 (0%)	0 (0%)	1 (10%)	4 (40%)	5 (50%)	0 (0%)	5.5 (5, 6)
**Acceptability**	0 (0%)	0 (0%)	0 (0%)	2 (20%)	4 (40%)	3 (30%)	1 (10%)	5 (5, 6)

Appendix 4: KT sustainability outcome definitions

Outcome	Definition/Measure/Cut-off value
Sustainability of the delivery of the Knowledge Translation (KT) intervention	Sustainability of the delivery of the KT intervention >1 year after implementation or termination of study funding
Quality of Life/Disease or Symptom severity measures	St. George's Respiratory Questionnaire<br>• Medical Outcomes Survey<br>o 36-item Veterans general health scale (SF-36V)<br>o 12-item Veterans general health scale (SF-12v)<br>o 36-item Short Form Health Survey (SF-36)<br>o 20-item Short Form Health Survey (SF-20)<br>o 12-item Short Form Health Survey (SF-12)<br>• Euroqol Quality of Life (EQ-5D)<br>o Visual Analogue Scale (EQ-VAS)<br>o EuroQol-5D-5L<br>o EuroQol-5D-3L<br>• Minnesota Living with Heart Failure Questionnaire
Process/Quality of care measures	• Proportion of patients receiving necessary/appropriate screening tests<br>• Proportion of patients receiving appropriate diagnoses/assessments<br>• Level/quality of communication with patient<br>• Treatment efficacy<br>• Adherence to treatment/practice guidelines

Appendix 5: Search Strategy for MEDLINE

Note: The following search was adjusted and translated for other databases as necessary.

Database: Ovid MEDLINE: Epub Ahead of Print, In-Process & Other Non-Indexed Citations, Ovid MEDLINE® Daily and Ovid MEDLINE® <1946-Present>

Search Strategy:

----

1 exp Chronic Disease/ (246037)

2 ((chronic or longterm or long-term) adj (disease*or illness* or condition*)).tw,kw. (15265)

3 chronic* ill*.tw,kw. (18361)

4 exp *Angina Pectoris/ (27035)

5 (stenocardia* or angina).ti. (18554)

6 exp *Cardiovascular Diseases/ (1835963)

7 ((cardiovascular or cardio-vascular) adj (disease* or abnormalit* or infection*)).ti. (30520)

8 (aneurysm* or aortic rupture* or endoleak* or microaneurysm* or hypertension or high blood pressure* or heart disease* or heart failure or cardiac failure or cardiac edema* or cardia oedema* or cardiac arrest*).ti. (345556)

9 (arrhythmia* or bradycardia* or tachycardia* or cardiac dysrhythmia* or heart block* or ventricular fibrillation* or atrial fibrillation*).ti. (82590)

10 (cardiomegaly or hypertrophy or enlarged heart or heart enlarge*).ti. (22118)

11 (cardiomyopath* or cardio-myopath* or myocarditis or myocardial disease* or endomyocardial fibrosis).ti. (40633)

12 (thrombosis or thrombus or thromboses or thromboembolism* or embolism* or thrombophlebitis* or lemierre syndrome).ti. (93101)

13 (peripheral vascular disease* or peripheral angiopath* or peripheral arterial disease* or phlebitis or raynaud disease*).ti. (7180)

14 (arteriosclerosis or atherosclerosis or atheromata* or atherogenesis).ti. (41783)

15 (coronary disease* or myocardial infarct* or heart attack*).ti. (87994)

16 exp *Cerebrovascular Disorders/ (266818)

17 ((cerebrovascular or cerebro-vascular or vascular) adj (disorder* or disease* or occlusion*)).ti. (16450)

18 (ischemia or stroke or strokes or carotid artery disease* or arterial disease* or vascular dementia or arteriovenous malformation*).ti. (159210)

19 (intracranial h?emorrhage* or cerebral h?emorrhage* or cerebrovascular trauma* or vascular headach* or central nervous system vasculitis or Intracranial vasospasm* or brain infarc*).ti. (5482)

20 exp *Ischemia/ (35183)

21 exp *Arthritis/ (192902)

22 (arthritis or osteoarthritis or osteo-arthritis or gout or caplan syndrome or sjogren* syndrome or rheumatoid nodule or rheumatoid vasculitis or still* disease* or periarthritis or rheumatic fever or spondylarthritis).ti. (134111)

23 exp *Osteoporosis/ (36260)

24 Osteoporosis.ti. (22685)

25 exp *Asthma/ (97349)

26 (asthma or asthmas).ti. (74639)

27 exp *Neoplasms/ (2617204)

28 (carcinoma* or neoplasm* or tumor* or tumour* or cancer* or adenocarcinoma* or malignanc* or ependymoma* or leukemia* or lymphoma* or sarcoma* or rhabdomyosarcoma* or osteosarcoma* or adenoma* or adenosarcoma* or carcinosarcoma* or blastoma* or nephroma* or thymoma* or melanoma* or retinoblastoma*).ti. (1999444)

29 exp *Pulmonary Disease, Chronic Obstructive/ (38293)

30 (COPD or chronic obstructive pulmonary disease* or chronic bronchitis or pulmonary emphysema*).ti. (37059)

31 exp *Inflammatory Bowel Diseases/ (60361)

32 *Irritable Bowel Syndrome/ (4994)

33 (ulcerative colitis or crohn* disease* or inflammatory bowel disease* or irritable bowel syndrome* or mucous colitis).ti. (60779)

34 exp *Diabetes Mellitus/ (300755)

35 (diabetes or diabetic or IDDM or wolfram syndrome* or T1DM or T2DM or T1 DM or T2 DM or NIDDM).ti. (291463)

36 exp *Mental Disorders/ (918626)

37 exp *Sleep Wake Disorders/ (61709)

38 ((drug or substance or alcohol or opioid or fentanyl or marijuana or cocaine or tobacco or amphetamine) adj (addiction* or abus* or dependenc* or disorder*)).ti. (26830)

39 (depression* or depressive disorder* or anxiet* or dementia).ti. (168517)

40 (sleep* adj2 disorder*).ti. (6970)

41 exp *HIV Infections/ (221809)

42 (acquired immunodeficiency syndrome* or AIDS arteritis or AIDS virus or AIDS related or AIDS dementia or HIV infection* or human immunodeficiency virus* or HIV seropositivit* or HIV wasting syndrome*).ti. (59111)

43 or/1-42 (6883217)

44 Caregivers/ (29045)

45 (care giver* or caregiver* or care next giver* or carer* or companion* or client care attendant* or home-care assistant* or home-care worker* or home care worker* or home-care aide* or home care aide* or home health care worker* or home healthcare worker* home health care assistant* or home healthcare assistant* or home health care aide* or home healthcare aide* or homemaker* or home-maker* or housekeeper* or house-keeper* or home support worker* or home health aid* or home visitor* or in-home assistant* or personal aide* or personal assistant* or personal care aide* or personal care attendant* or personal care provider* or personal support aide* or personal support worker* or respite worker* or personal attendant*).tw,kw. (76491)

46 44 or 45 (84834)

47 43 and 46 (33680)

48 aged.sh. or age*.tw. (5142994)

49 43 and 48 (2202098)

50 limit 43 to "all aged (65 and over)" (1495597)

51 47 or 49 or 50 (2232508)

52 (randomized controlled trial or controlled clinical trial or pragmatic clinical trial).pt. (540449)

53 (randomized or placebo or randomly).tw. or trial.ti. (828494)

54 clinical trials as topic/ (182450)

55 or/52-54 (1139198)

56 51 and 55 (222097)

57 animals/ not humans/ (4389953)

58 56 not 57 (219701)

59 Waiting Lists/ (10402)

60 (wait* list* or queu*).tw,kw. (12975)

61 exp *"Continuity of Patient Care"/ (121453)

62 (care continuity or "coordination of care" or shared care).tw,kw. (2925)

63 ((continui* or continuum*) adj2 (care or healthcare)).tw. (10868)

64 Triage/ (9887)

65 (triage* or triaging).tw,kw. (15459)

66 *Quality Assurance, Health Care/ (30805)

67 exp *Quality Improvement/ (9023)

68 *total Quality Management/ (7201)

69 (quality assurance or total quality management or TQM or continuous quality improvement or CQI or QI).tw,kw. (31595)

70 (quality adj (improvement$ or initiativ$ or intervention$ or program$ or plan$ or audit$)).tw,tw. (29386)

71 outreach.tw,kw. (11034)

72 exp Self Care/ (48833)

73 *Self Efficacy/ (6652)

74 exp Self Help Devices/ (10153)

75 (self car* or self-car* or self help or self-help or self manag$ or self-manag$ or self-monitor* or self monitor* or goal setting or self efficacy or self-efficacy).tw,kw. (60547)

76 exp *social support/ (22867)

77 (social network* or social support* or social systems*).tw,kw. (44860)

78 ((length or time) adj3 (consult* or consultation*)).tw,kw. (2499)

79 exp *"Referral and Consultation"/ (28117)

80 models, nursing/ (11701)

81 "Personnel Staffing and Scheduling"/og [Organization & Administration] (3311)

82 (staff* adj (model or models)).tw,kw. (684)

83 exit interview*.tw,kw. (783)

84 Personnel Turnover/ and (Interviews as Topic/ or interview*.tw,kw.) (322)

85 Personnel Selection/ and (exp Rural Health Services/ or Medically Underserved Area/) (501)

86 ((recruit* or hire or hiring or retention or retain* or train* or retrain* or re-train* or personnel select* or staff select*) adj2 (underserv* or rural or physician shortage* or doctor shortage* or health worker* or health manager* or health system manager*)).tw,kw. (2440)

87 district health manager*.tw,kw. (59)

88 or/59-87 (455087)

89 58 and 88 (12440)

90 exp Patient Care Planning/ (59223)

91 exp disease management/ (56137)

92 (advance care planning or care pathway* or case management or case coordination or case co-ordination or disease management or patient care management or patient care planning or patient handoff or patient hand-off or patient discharge or discharge plan* or care pathway*).tw,kw. (31419)

93 Geriatric Assessment/ (23231)

94 (geriatric assessment or psychogeriatric* or psycho-geriatri* or gerontolog* assessment*).tw,kw. (4701)

95 exp Interprofessional Relations/ (63960)

96 (interdisciplinary communication* or interprofessional communication* or interdisciplinary relation* or interprofessional relation* or inter-disciplinary communication* or inter-professional communication* or inter-disciplinary relation* or inter-professional relation*).tw,kw. (1648)

97 Communication/ and exp Health Personnel/ (13310)

98 (communication adj2 (provider* or manager* or professional* or health personnel or health care personnel or physician* or doctor* or nurse* or pharmacist* or medical staff* or health care worker* or health worker* or specialist*)).tw,kw. (9734)

99 ("package of care" or (package* adj care)).tw,kw. (172)

100 delivery of health care/mt (3815)

101 exp "Appointments and Schedules"/ and Patient Participation/ (116)

102 ((Patient initiated or patient-initiated) adj2 (follow up or follow-up or appointment* or schedul*)).tw,kw. (18)

103 Patient Participation/ and (exp "Appointments and Schedules"/ or follow up.tw,kw. or follow-up.tw,kw. or appointment*.tw,kw. or schedule*.tw,kw.) (1202)

104 "Referral and Consultation"/og (2815)

105 (referral system* or referral software*).tw,kw. (1238)

106 (shared adj2 care).tw,kw. (1532)

107 Decision Making/ (81893)

108 (shared decision making or shared decision-making).tw,kw. (5240)

109 patient transfer/ (7156)

110 ("transfer of care" or patient transfer*).tw,kw. (1637)

111 (guided care nurs* or transition coach* or nurse case manage* or advanced practice nurs* or care coordinator* or transition liaison nure* or nurse discharge advocate* or care transition nurs* or case manager* or transitional care nurs* or Home care nurs* or Liaison nurs* or Community care nurs* or System navigator* or Hospital home care coordinator* or Bed utilization coordinator* or Clinical nurse specialist* or Discharge coordinator* or Discharge planner* or Outreach coordinator* or Care coordinato* or Community case manager* or Patient flow coordinator* or Outreach nurs* or Outreach case manag* or Mental health case manag* or Community care coordinator* or Access homecare coordinato*r or Long term care transition* or elderly care transition*).tw,kw. (10214)

112 or/90-111 (340260)

113 58 and 112 (5062)

114 *Information Systems/ (12280)

115 Health Information Systems/ (897)

116 Integrated Advanced Information Management Systems/ (288)

117 exp Management Information Systems/ (44400)

118 "information and communication technolog*".kw,tw. (2002)

119 (health adj3 information system*).kw,tw. (3791)

120 (smart home* or smart environment*).kw,tw. (398)

121 exp telemetry/ (11504)

122 exp Telemedicine/ (21905)

123 (telemedicine or telepathology or telerehabilitation or teleradiology or telenurs* or mobile health or ehealth or Tele-medicine or tele-pathology or tele-rehabilitation or tele-radiology or tele-nurs*).kw,tw. (14975)

124 Absenteeism/ and exp policy/ (112)

125 (absenteeism* and polic*).tw,kw. (338)

126 exp *Accreditation/ or accreditation/ or accreditation.tw,kw. (21782)

127 Patient Rights/ (6569)

128 (patient* adj right*).tw,kw. (4311)

129 or/114-128 (128217)

130 58 and 129 (1258)

131 Organizational Culture/ or organizational culture*.tw,kw. or organisational culture*.tw,kw. or corporate culture*.tw,kw. (16639)

132 Organizational Innovation/ (22928)

133 "audit and feedback".tw,kw. (938)

134 ((audit or audits or auditing) adj2 feedback).tw,kw. (1029)

135 (Medical Errors/ or Risk Management/ or incident report.tw,kw.) and (system*.tw,kw. or exp information systems/) (7075)

136 ("communities of practice" or "community of practice" or "practice community" or "practice communities").tw,kw. (3526)

137 ((education* adj2 gam*) or (education* adj2 play)).tw,kw. (597)

138 exp Education/ and exp "Play and Playthings"/ (1933)

139 exp Teaching Materials/ (112194)

140 exp Inservice Training/ (27060)

141 (education* adj2 (printed or material* or meeting* or outreach or visit* or conference* or workshop* or train* or inservice or in-service or cours*)).tw,kw. (26318)

142 exp Education, Continuing/ (58644)

143 ((continuing or physician* or provider* or professional* or clinician* or doctor* or nurs* or pharmac*) adj2 (CME or educat* or train* or retrain* or re-train* or workshop* or professional development*)).tw,kw. (88447)

144 academic detail*.tw,kw. (457)

145 practice guidelines as topic/ (102126)

146 guideline adherence/ (27563)

147 ((guideline* or CPG or CPGs) adj3 (disseminat$ or adherence)).tw,kw. (6046)

148 (consensus adj (expert or local or develop* or conference* or process* or workshop*)).tw,kw. (7745)

149 (group adj (nominal or technique* or process* or consensus)).tw,kw. (3013)

150 opinion leader*.tw,kw. (1184)

151 ((opinion or education* or influential) adj leader*).tw,kw. (1608)

152 manag* supervis*.tw,kw. or *Nursing, Supervisory/ (5887)

153 patient mediat*.tw,kw. (62)

154 (Patient Participation/ or Consumer Participation/) and (Professional Practice/ or exp Professional Competence/ or performance.tw,kw.) (1359)

155 (performance data adj5 public).tw,kw. (67)

156 Reminder Systems/ (2991)

157 (recall adj2 system*).tw. (481)

158 (reminder* adj2 (prompt or system* or process* or manual or computer* intervention*)).tw,kw. (796)

159 patient reported outcome measure*.tw,kw. (2734)

160 patient reported outcome measures/ (1196)

161 (tailor* adj3 intervention*).tw,kw. (5956)

162 or/131-161 (464962)

163 58 and 162 (4916)

164 89 or 113 or 130 or 163 (20367)

165 Translational Medical Research/ (8083)

166 exp *Evidence-Based Practice/ (29723)

167 *Program Evaluation/ (9329)

168 *Information Dissemination/ (7295)

169 Knowledge Management/ (295)

170 *"Outcome and Process Assessment (Health Care)"/ (9050)

171 "process assessment (health care)"/ (4022)

172 "Outcome Assessment (Health Care)"/ (62270)

173 exp "diffusion of innovation"/ (18025)

174 *Program Development/ (7077)

175 (knowledge adj2 (translation or disseminat* or diffus* or exchange or transfer*)).tw,kw. (5931)

176 sustain*.tw,kw. (285655)

177 ((adherence or maintenance or adoption or adaptation or effect* or report or reporting or improv* or implement*) adj3 (innovation* or intervention* or strateg* or outcome* or process*)).tw,kw. (375574)

178 (diffusion adj3 innovation*).tw,kw. (911)

179 (continued adj2 effect*).tw,kw. (1164)

180 or/165-179 (781353)

181 164 and 180 (5529)

182 (editorial or comment).pt. (1021117)

183 181 not 182 (5518)

184 "Costs and Cost Analysis"/ (45658)

185 (cost* or cost benefit analys* or cost effective*).tw. (498399)

186 Cost-Benefit Analysis/ (71461)

187 Health Care Costs/ (34182)

188 or/184-187 (549206)

189 88 or 112 or 129 or 162 (1233580)

190 51 and 189 (124651)

191 188 and 190 (8426)

192 180 and 191 (2288)

193 192 not 183 (1458)

Appendix 6. Coding Guide for KT Intervention Components Using EPOC Taxonomy Coding

Component	CODE	Description	Examples from the included studies
QI strategies targeting health systems			
Electronic patient registry	hs EPR	General electronic medical record system or electronic tracking system for patients with chronic diseases. We do not include websites unless patients were tracked over time. To qualify, the system has to have been part of the clinical trial as an intervention (i.e., not pre-existing infrastructure unless used more actively).	*"...A key intervention element was health care organization- and community agency-based dementia care managers (primarily social workers) who received formal training and used an Internet-based care management software system for care planning and coordination... Assessment responses were entered into the software system, generating a preliminary problem list and guides to care-plan actions...The software system had a feature to enable efficient tracking of multiple cases and tasks. Referrals to a particular community agency were guided by flagged problem areas..." [26]*
Financial incentives	hs finan	Interventions with positive or negative financial incentives directed at system-wide changes in reimbursement (e.g., capitation, prospective payment, or a shift from fee-for-service to salary pay structure).	*“Specifically, we organized the distribution of printed educational brochures, pedometers and home blood glucose material (HBGM), which are not typically reimbursed in Belgium for patients on oral antidiabetic drugs or followed up solely by primary care.” [27]*
QI strategies targeting health-care providers			
Team changes	team	Changes to the structure or organisation of the primary health-care team (adding team member, multidisciplinary teams, expansion, or revision of professional roles)	*"Each site had two full-time clinical pharmacist equivalents, three pharmacists at KP (2 were half-time) and two in VA who worked exclusively with patients on intervention teams" [28]*
Electronic patient registry	EPR	General electronic medical record system or electronic tracking system for patients with chronic diseases. We do not include websites unless patients were tracked over time. To qualify, it had to be a part of the clinical trial as an intervention (i.e., not pre-existing infrastructure unless used more actively)	*"Electronic clinical information systems allow for “real time” access to individual patient information, as well as the tracking of individual contacts and interventions. The tracking systems provide e-mail alerts notifying the NCM about specific patient contact within the Carle system, including emergency department visits, hospital admissions and discharges, outpatient*

Component	CODE	Description	Examples from the included studies
			procedures, and appointments with other clinicians" [29]
Facilitated relay of info to clinicians	Relay	Clinical information collected from patients and transmitted to clinicians by means other than the existing medical record (excluding conventional means of correspondence between clinicians.)	*"Participants randomized to the intervention group received a home telemedicine unit... consisting of a Web-enabled computer with modem connection to an existing telephone line. The HTU provided four major functions: (i) video-conferencing over plain old telephone service (POTS) connections at eight to 15 frames per second allowing patients to interact with nurse case managers... (ii) remote monitoring of glucose... (iii) dial-up Internet service provider access to a Web portal providing access to patients' own clinical data and secure Web-based messaging with nurse case mangers; and (iv) access to an educational Web site created for the project" [30]*
Continuous QI	cQI	Interventions explicitly identified as involving the techniques of continuous QI, total quality management, or plan-do-study-act, or any iterative process for assessing quality problems, developing solutions to those problems, testing their effects, and then reassessing the need for further action.	"*All ACQIP physicians participated in an intensive quality improvement program in which they were informed of their individual performance on the ACQIP indicators as well as of the mean performance of their peers... Each physician received this information in mailings approximately 3 to 6 weeks apart during 1996, according to a schedule developed by HCFA and AQAF. With assistance from AQAF, physician offices developed quality improvement plans (QIPs), currently on file at AQAF. The extensive and multimodal QIPs included formalized group meetings, root cause analysis, and changes of care at the office level, such as posting of patient educational material, use of chart interventions in the practice environment, reminders, clinical flow sheets, and standing orders for appropriate administration of influenza vaccination." [31]*
Audit & feedback	AF	Summary of clinical performance of health care delivered by an individual clinician or clinic over a specified period, which was then transmitted back to the clinician. This	*"Because data about their performance have been shown to contribute to and reinforce practice changes, many NHs receive periodic reports*

Component	CODE	Description	Examples from the included studies
		strategy was strictly based on clinical data and excluded clinical skills.	*about residents prescribed antipsychotics from their pharmacies. Enhanced audit and feedback reports were developed that presented aggregated facility-level atypical antipsychotic prescribing data for the most recent quarter and quarterly for the prior 12 months and benchmark comparisons with state and national prescribing levels that are not typically provided to facilities. Important information about the efficacy and safety of atypical antipsychotics from the CERSG was also provided." [32]*
Staff education	staff	Interventions for staff designed to promote increased understanding of principles guiding clinical care or awareness of specific recommendations for a target disorder or population of patients.	*"The general practitioners were introduced to possible solutions to therapeutic problems through clinical guidelines supported by an annual half-day seminar [33]*
Clinician reminders	clin_reminder	Paper-based or electronic systems intended to prompt a health professional to recall patient-specific information (e.g., most recent HbA1c value) or to do a specific task (e.g., foot examination).	*"This study aimed to evaluate, within a pragmatic, cluster-randomised controlled trial design, the effectiveness and efficiency of an area-wide, 'extended' computerised diabetes register incorporating a full-structured recall and management system, actively involving patients, and including individualised patient-management prompts to primary care clinicians based on locally-adapted, evidence-based guidelines." [34]*
Financial incentives	finan	Interventions with positive or negative financial incentives directed at providers (e.g., linked to adherence to some process of care or achievement of some target outcome).	*"The nurse also organized appointments for participants to discuss the CDM plan with their GP. GPs were remunerated for using the CDM plan, providing an incentive for them to participate in the trial as well as a pathway for implementation of trial outcomes" [35]*
QI strategies targeting patients			
Case management	case-M	Any system for coordinating diagnosis, treatment, or routine management of patients (e.g., arrangement for referrals, follow-up of test results) by a person or multidisciplinary team in collaboration with, or supplementary to, the primary-care clinician. If the study called the intervention 'case management', we classified it as such.	*"We designated one nurse from each of the eight clusters (primary care clinics) as the clinic's depression care manager... We gave nurse care managers an additional 3 h of group training in their care management role and responsibilities; they then participated in monthly supervision and continuing education sessions led by the study*

Component	CODE	Description	Examples from the included studies
			psychiatrist for the duration of the study. Care manager responsibilities included education of patients and families about their illnesses, assistance with communication between patients and providers, and support of patients' adherence to treatment. During the 16 week acute phase of treatment, primary care nurses telephoned patients every 2 weeks to assess their antidepressant use and side-effects, administered the PHQ-9 to assess treatment response, and encouraged patients to keep their appointments. In alternate weeks to the telephone call, patients attended visits to their primary care clinic and the care managers administered the PHQ-9. In this manner, depression scores on the PHQ-9 were taken every week" [36]
Promotion of self-management	Self-M	Provision of equipment or access to resources to promote self-management. If the study called the intervention promotion of self-management, personalised goal-setting, or action-planning, we included it here. In general, we perceive this as a more active strategy than education of patients.	*"Assessment of patient need was linked to appropriate support, including self help guidebooks based on published development methods, access to relevant community groups and programs via a web-based directory of local self management resources..." [37]*
Patient Reminders	px reminder	Any effort to remind patients about upcoming appointments or important aspects of self-care. If the intervention included case management, reminders to patients needed to be explicit and to represent an extra task as compared to normal case management.	*"Participants randomized to the wait-list control group were instructed to wear the pedometer every day, reminded monthly to log in to the website to upload step-count data, and asked to report all adverse events" [38]*
Patient education	px_educ	Interventions designed to promote greater understanding of a target disorder or to teach specific prevention or treatment strategies, or specific in-person education (e.g., individual or group sessions with diabetes nurse educator, distribution of printed or electronic educational materials). Interventions with education of patients are included only if they also include at least one other strategy related to clinician or organizational change. We do not include occasions of optional education.	*"During hospital admission all patients received standard evidence-based treatment with the patients of education group participated in 1-hour one-on-one teaching session before hospital discharge" [39]*
Motivational interview	mot_interview	'a directive and client-centred counselling style that relies upon identifying and mobilising the client's intrinsic values and goals to stimulate behaviour change [40], thus	*Motivational Interviewing (MINT) is a patient-centered, directive method for enhancing intrinsic motivation to change by exploring and resolving*

Component	CODE	Description	Examples from the included studies
		encouraging client and family involvement in all aspects of care.’	*the ambivalence subjects feel with respect of health choices... In the study, nurses delivered a MINT call every 3 months (four calls in a period of 12 months) to elicit individual values, preferences, arguments for change, reasons for past failures, and to empower patients to resolve ambivalence and develop a behavior modification plan" [41]*
Financial incentives	px_finan	Interventions with positive or negative financial incentives directed at patients/caregivers (e.g., linked to adherence to some process of care or achievement of some target outcome).	*"All patients were compensated $25.00 per visit" [42]*

Abbreviations: QI, Quality Improvement

Appendix 7. Coding Guides for Clinical Intervention Components using BCT coding

Component	CODE	Description	Example
QI strategies targeting healthcare system			
1.1 Goal setting (behaviour) – not tailored	NT_HS_1.1	The individual is encouraged to make a behavioural resolution. This is directed towards encouraging people to decide to change or maintain change.	*Medical assistants developed specific action plans to achieve these goals together with patients and caregivers.* [48]
1.3 Goal setting (outcomes) – not tailored	NT_HS_1.3	The individual is encouraged to set a general goal that can be achieved by behavioural means but is not defined in terms of behaviour (e.g., lose/maintain weight). The goal may be an expected consequence of one or more behaviours, but is not a behaviour per se. Use content of reported outcomes to decide whether to code Goal setting (outcome) and/or Goal setting (behaviour). If unclear if one or other, code both (see 1.1)	*Personalized quality improvement reports pushed directly to the site by email each quarter, as well as tailored teleconferences, webinars, and specialized tool kits for healthcare providers.*[34]
2.1 Monitoring of behaviour by others - not tailored	NT_HS_2.1	Must be active and in service of changing behaviour, not simply routine data collection that is already in place.	*Hospitals continued to receive access to the usual on-demand reports, 'Get with it the Guidelines – Heart Failure' quality improvement tools, and publicly available GWTG-HF webinars. These reports continued to be available on request but were not actively pushed to the sites on a routine basis…*[34]
2.2 Feedback on behaviour – not tailored	NT_HS_2.2	Coded only when there was clear feedback about behaviour. This code implies a monitoring process providing feedback.	*Intervention added to the current baseline reports with personalized quality improvement reports pushed directly to the site by email each quarter…The personalized reports were designed to describe the site's heart failure patient population compared with other "Get with the guidelines – Heart Failure hospitals, highlight performance on both GWTG-HF achievement measures and 9 GWTG-HF quality metrics…*[34]
2.7 Feedback on outcome(s) of behaviour – not tailored	NT_HS_2.7	Coded only when there is clear feedback about outcomes. This code implies a monitoring process providing the feedback data.	*Processing that data to make it informative and providing comparative feedback combined with academic detailing. A key feature is presenting comparative feedback comparing practices at twice yearly meetings held within a locality/primary care organization.*[31]
4.1 Instruction on how to perform a behaviour – not tailored	NT_HS_4.1	Coding in relation to skills acquisition/development.<br><br>Code algorithms and decision trees (also consider coding as Adding objects to environment if material objects or software).	*Training (developed and piloted with two non-trial practices) was delivered in each practice over two sessions, which we estimated through informed feedback was the maximum feasible in UK primary care using current educational structures. Session 1 practices worked on ways to embed self management tools in their systems; in session 2, clinicians practised ways to use core*

Component	CODE	Description	Example
			self management skills in consultations and ensure patients received, or were directed to, appropriate resources. [71]
12.5 Adding objects to the environment – not tailored	NT_HS_12.5	Code for the addition of new physical or virtual (software) aimed at facilitating behaviour change. If something existing is changed, consider coding to Restructuring physical environment.	*In addition to personalized reporting…individuals also received access to webinars, teleconferences and specialized tool kits* [34]
QI strategies targeting health-care providers			
1.1 Goal setting (behaviour) – tailored/not tailored	T_HP_1.1/ NT_HP_1.1	The individual is encouraged to make a behavioural resolution. This is directed towards encouraging people to decide to change or maintain change.	*The initial assessment involved the entire team and took approximately 2 hours. After the assessments were complete, the team developed goals, interventions, treatment, and individualized follow-up for each patient.*[18]
1.2 Problem solving – not tailored	NT_HP_1.2	The person is prompted to think about potential barriers and identify the ways of overcoming them. Barriers may include competing goals in specified situations. This may be described as 'problem solving'. If it is problem solving in relation to the performance of a behaviour, then it counts as an instance of this technique.	*Multimodal QIPs included formalized group meetings, root cause analysis, and changes of care at the office level, such posting of patient educational material, use of chart interventions in the practice environment…*[74]
1.3 Goal setting (outcomes) – tailored/not tailored	T_HP_1.3/ NT_HP_1.3	The individual is encouraged to set a general goal that can be achieved by behavioural means but is not defined in terms of behaviour (e.g., lose/maintain weight). The goal may be an expected consequence of one or more behaviours, but is not a behaviour per se. Use content of reported outcomes to decide whether to code Goal setting (outcome) and/or Goal setting (behaviour). If unclear if one or other, code both (see 1.1)	*The medical coordinator discussed and agreed guidelines for secondary prevention with the practice doctors and gave ongoing support in setting up a register and recall system for regular review of patients with coronary heart disease by their general practitioner.*[101]
1.4 Action planning	NT_HP_1.4	Detailed planning of performance of the behavior (must include at least one of context, frequency, duration and intensity). Context ca be considered environmental (physical or social) or internal (physical, emotional or cognitive)	*An action plan for each practice was agreed with the practice and regularly reviewed by the study research nurse and practice.*[105]
1.7 Review outcome goal(s) – tailored	T_HP_1.7	Involves a review or analysis of the extent to which previously set outcome goals (e.g., to reduce blood pressure or lose/maintain weight) were achieved. In most cases, this will follow previous goal setting (see technique 6, goal	*The general practitioner was also requested to define, together with the patient, the best possible goals for blood glucose concentration, glycated haemoglobin, diastolic blood pressure, and lipids within three predefined categories…. At follow up, the general practitioner was asked to compare the achievements with*

Component	CODE	Description	Example
		setting-outcome') and an attempt to act on those goals, followed by a revision of goals, and/or means to attain them.	*the goal and consider changing either goal or treatment accordingly.*[29]
2.1 Monitoring of behaviour by others - not tailored	NT_HP_2.1	Must be active and in service of changing behaviour, not simply routine data collection that is already in place.	*First, the care manager had weekly meetings with a support team…. who reviewed the care of new and active patients and monitored adherence to the standard protocols. Web-based longitudinal tracking system that managed the schedule for patient contacts, tracked the patient's progress and current treatments, and provided an instrument for communicating the patient's and caregiver's current clinical status to the entire care team*[20]
2.2 Feedback on behaviour – tailored/not tailored	T_HP_2.2/ NT_HP_2.2	Coded only when there was clear feedback about behaviour. This code implies a monitoring process providing feedback.	*Physicians in the quality improvement program were given performance feedback based on several quality measures…an achievable benchmark for each indicator in the final report was mailed to physicians…*[74]
2.4 Self-monitoring of outcome(s) of behaviour – not tailored	NT_HP_2.4	The person is asked to keep a record of specified measures expected to be influenced by the behaviour change, e.g., blood pressure, blood glucose, weight loss, physical fitness.	*Medical assistants monitored goal achievement and symptom deterioration either face-to-face with patients in the clinic or by telephone using paper-based checklists. Monitoring intervals were tailored to the patient's health status but were scheduled at least once every 6 weeks. Primary care physicians met with medical assistants weekly to review patient progress.*[48]
2.5 Monitoring outcome(s) of behavior by others without feedback	NT_HP_2.5	Observe or record outcomes of behavior with the person's knowledge as part of a behavior change strategy.	*Alerts were automatically transmitted via text message to the responsible physician but were 'silent' (inaudible) to the patient.*[11]
2.7 Feedback on outcome(s) of behaviour – tailored/not tailored	T_HP_2.7/ NT_HP_2.7	This involves providing the participant with data about their own recorded behaviour or commenting on a person's behavioural performance (e.g., identifying a discrepancy with between behavioural performance and a set goal	*Practices were given summary audit results at a practice meeting (one practice requested written material only)… Anonymized data from other practices in the study were given for comparison.*[101]
3.1 Social support (unspecified) – tailored	T_HP_3.1	Advise on, arrange or provide social support (e.g., from friends, relatives, colleagues,' buddies' or staff) or non-contingent praise or reward for performance of the behavior.	*Counseling and individual coaching were provided after class...Two monthly support calls for 6 consecutive months after the end of the systematic classroom course.*[71]
3.2 Social support (practical) – not tailored	NT_HP_3.2	Advise on, arrange, or provide practical help (e.g., from friends, relatives, colleagues, 'buddies' or staff) for performance of the behavior	*All providers on the intervention teams agreed that AIM pharmacists assigned to their teams could proactively reach out to eligible patients…. pharmacists were authorized to make medication changes; at all sites the clinical pharmacists copied the participating patient's assigned provider on all of that*

Component	CODE	Description	Example
			patient's clinical notes and alerted the PCP when one of that PCP's patients declined participation in the program, entered the program, or was discharged….[56]
4.1 Instruction on how to perform a behaviour – not tailored	NT_HP_4.1	Involves telling the person how to perform behaviour or preparatory behaviours, either verbally or in written form.	*In addition to providing patients with information, heart failure nurses were trained to increase patient self-efficacy…*[67]
6.1 Demonstration of the behavior	NT_HP_6.1	Provide an observable sample of the performance of the behaviour, directly in person or indirectly e.g., via film, pictures, for the person to aspire to or imitate or model	*Occupational therapists received a training which included 5-7 coaching-on-the-job sessions led by a study expert* [35]
6.2 Social comparison – not tailored	NT_HP_6.2	Draw attention to others' performance to allow comparison with the person's own performance Note: being in a group setting does not necessarily mean that social comparison is actually taking place	*Practices were given summary audit results at a practice meeting (one practice requested written material only)… Anonymized data from other practices in the study were given for comparison.*[101]
7.1 Prompts/cues – tailored/not tailored	T_HP_7.1/ NT_HP_7.1	Introduce or define environmental or social stimulus with the purpose of prompting or cueing the behavior. The prompt or cue would normally occur at the time or place of performance	*Automatic alerts were generated and presented to the nurses if the signals were out of the patient-specific present range*[69]
8.1 Behavioral practice/ rehearsal	NT_HP_8.1	Prompt practice or rehearsal of the performance of the behavior one or more times in a context or at a time when the performance may not be necessary, in order to increase habit and skill	*Ten staff members, two from each intervention care home, attended the basic and advanced training given by research group and became certified dementia-care mappers. Advanced users are able to observe, report, provide feedback to the staff, and instruct and support them in drawing up action plans.* [145]
9.1 Credible source – tailored/not tailored	T_HP_9.1/ NT_HP_9.1	Present verbal or visual communication from a credible source in favour of or against the behavior. Consider national organisations, national guidelines and respected peers as credible sources.	*Providers in control clinics were sent a link to an existing VA Web site that contained links to a wide range of clinical guidelines for various medical conditions*[82]
10.1 Material incentive (behaviour) – not tailored	NT_HP_10.1	Inform that money, vouchers or other valued objects will be delivered if and only if there has been effort and/or progress in performing the behavior (includes 'Positive reinforcement')	*Practices were remunerated so that there was no cost to the patient for COPD-related GP consultations*[117]
12.1 Restructuring the physical environment – tailored/not tailored	T_HP_12.1/ NT_HP_12.1	Change, or advise to change the physical environment in order to facilitate performance of the wanted behavior or create barriers to the unwanted behavior (other than prompts/cues, rewards and punishments).	*Care planning templates were provided in an electronic form for use by the nurse and GP in computerized clinical record systems. The templates were designed for smokers and non-smokers respectively and had prompts for assessment, goals and actions based on the COPD guidelines.*[158]

Component	CODE	Description	Example
		Code only if it is a change to an existing physical or virtual (i.e., software/web) structure	
12.2 Restructuring the social environment – tailored/not tailored	T_HP_12.2/ NT_HP_12.2	Change, or advise to change the social environment in order to facilitate performance of the wanted behavior or create barriers to the unwanted behavior (other than prompts/cues, rewards and punishments)	*Accessibility to the specialised nurse at the hospital was ensured for primary care professionals during the follow-up period through an ICT platform including a web-based call centre.*[58]
12.5 Adding objects to the environment – not tailored	NT_HP_12.5	Code for the addition of new physical or virtual (software) aimed at facilitating behaviour change. If something existing is changed, consider coding to Restructuring physical environment.	*Medical coordinator discussed and agreed guidelines for secondary prevention with the practice doctors and gave ongoing support in setting up a register and recall system for regular review of patients with coronary heart disease by their general practitioner.*[101]
13.2 Umbrella term - Clinician/staff education – tailored/not tailored	T_HP_13.2/ NT_HP_13.2	Unclear description	*Staff from each organization notified the study data manager of eligible patients and who were then randomized to either study arm -Enhanced Usual Care (EUC) which included routine depression screening and staff training in depression care*[40]
QI strategies targeting caregivers			
1.2 Problem solving	NT_C_1.2	Analyse, or prompt the person to analyse, factors influencing the behavior and generate or select strategies that include overcoming barriers and/or increasing facilitators	*In the office setting, a master's-prepared health educator–interventionist met with the caregiver to discuss his or her problems and the appropriate pamphlets or strategies for addressing them, while a research specialist sat with the care recipient*[19]
1.3 Goal setting (outcome)	NT_C_1.3	Set or agree on a goal defined in terms of a positive outcome of wanted behavior	*The intervention protocol called for the care consultant to have monthly contact for 12 months with each family caregiver. Responsibilities at each contact were to determine which aspects of dementia symptoms and care responsibilities caused caregiver concerns, discuss action steps to address caregiver concerns, and compose a written care plan* [47]
1.4 Action planning	NT_C_1.4	Prompt detailed planning of performance of the behavior (must include at least one of context, frequency, duration and intensity).	*The care manager collaborated with the caregiver to prioritize problem areas; teach problem-solving skills; initiate care plan actions; and send an assessment summary, a problem list, and selected recommendations to the patient's primary care physician and other designated providers. A menu of potential care plan actions (for example, referral for respite care services) was documented in a comprehensive care management manual.*[148]
2.4 Self-monitoring of outcome(s) of	NT_C_2.4	The person is asked to keep a record of specified measures expected to be influenced by the	*At each contact with the care manager, caregivers completed the Memory and Behavior Problems Checklist to assess current*

Component	CODE	Description	Example
behaviour – not tailored		behaviour change, e.g., blood pressure, blood glucose, weight loss, physical fitness.	*symptoms and stressors. Based on the caregiver's responses, individualized recommendations were made regarding how to manage a patient's behavioral symptoms.*[20]
3.1 Social support (unspecified) – tailored	T_C_3.1	Advise on, arrange or provide social support (e.g., from friends, relatives, colleagues,' buddies' or staff) or non-contingent praise or reward for performance of the behavior.	*The minimum care plan for all family caregivers included the action steps that family caregivers should take to learn more about or use; key information about the clinical course of the disease process; legal and financial planning issues; family support groups; dementia educational programs offered by the chapter and other organizations; adult day care services; and respite care services. The care consultant's initial and final meetings with family caregivers occurred in the home of the family caregiver and/or patient.* [47]
4.1 Instruction on how to perform a behaviour – not tailored	NT_C_4.1	Involves telling the person how to perform behaviour or preparatory behaviours, either verbally or in written form.	*Participants were mailed instructions on the IVF monitoring and provided 12 months of self-management support phone calls.*[114]
7.1 Prompts/cues	NT_C_7.1	Introduce or define environmental or social stimulus with the purpose of prompting or cueing the behavior. The prompt or cue would normally occur at the time or place of performance	*Patients received weekly IVR monitoring and self-management support calls for 12 months. IVR calls included recorded information and questions that patients answered using their touchtone keypad. Calls lasted roughly 10 minutes and followed a tree-structured algorithm to ask about overall health, HF symptoms, and self-management behaviors.*[114]
11.2 Reduce negative emotions – not tailored/not tailored	NT_C_11.2	Advise on ways of reducing negative emotions to facilitate performance of the behavior (includes 'Stress Management')	*Relevant issues were identified (e.g., management of patient behavior problems, coping with feelings of guilt) and the counselor motivated the family to form ideas to help the caregiver and to delegate tasks. The follow up meetings reviewed the previous session, previous commitments and the progress of tasks. Ad hoc telephone counseling from the same counselor was available to caregivers and their families beyond the scheduled sessions.*[68]
12.2 Restructuring the social environment	NT_C_12.2	Change, or advise to change the social environment in order to facilitate performance of the wanted behavior or create barriers to the unwanted behavior (other than prompts/cues, rewards and punishments)	*All dyads received mailed monthly personalized letters with information on signs and symptoms of stroke, stroke prevention, stress reduction strategies, diet and exercise guidelines, links to support groups and advocacy organizations, and tips for leisure activity adaptations.* [111]
13.1 Identification of self as role model	NT_C_13.1	Inform that one's own behavior may be an example to others	*Care partners received guidelines about how to communicate in a positive motivating way, avoid conflict by respecting boundaries, include in-home caregivers, and respect confidentiality. Patients*

Component	CODE	Description	Example
			received a notebook including reminders and tips for their weekly patient-care partner calls. [114]
13.3 Umbrella term - Caregiver training/support – not tailored	NT_C_13.3	Unclear description	*During these sessions, caregivers were taken to a support session led by a social psychologist that focused on caregiver stress.*[20]
QI strategies targeting patients			
1.1 Goal setting (behaviour) – tailored/not tailored	T_P_1.1/ NT_P_1.1	The individual is encouraged to make a behavioural resolution. This is directed towards encouraging people to decide to change or maintain change.	*The intervention aimed to empower patients to manage their COPD independently by improving their understanding of disease and monitoring of symptoms by developing their confidence to carry out appropriate actions, such as altering treatment early in the evolution of an exacerbation…*[17]
1.2 Problem solving	NT_P_1.2	Analyse, or prompt the person to analyse, factors influencing the behavior and generate or select strategies that include overcoming barriers and/or increasing facilitators	*A patient self management group session (45 minutes), led by a team nurse or social worker, that emphasized self-management skills and group problem-solving for chronic health problems (individual groups were encouraged to select the topics, some of which included physical activity, nutrition, and advanced care planning)* [26]
1.3 Goal setting (outcomes) – tailored/not tailored	T_P_1.3/ NT_P_1.3	The individual is encouraged to set a general goal that can be achieved by behavioural means but is not defined in terms of behaviour (e.g., lose/maintain weight). The goal may be an expected consequence of one or more behaviours, but is not a behaviour per se. Use content of reported outcomes to decide whether to code Goal setting (outcome) and/or Goal setting (behaviour).	*Patients were provided with a welcome packet…which included mailed with an introduction letter from the pharmacist and educational materials, including instructions for home monitoring and documents to record BPs and action plans…*[56]
1.4 Action planning – tailored/not tailored	T_P_1.4/ NT_P_1.4	Prompt detailed planning of performance of the behavior (must include at least one of context, frequency, duration and intensity). Context may be environmental (physical or social) or internal (physical, emotional or cognitive) (includes 'Implementation Intentions')	*An individualized exercise plan was developed with the participants...Participants were encouraged to complete endurance exercises at least four times per week for 30 minutes per session and arm strengthening exercises at least three*[106]
1.5 Review behavior goal(s)	NT_P_1.5	Review behavior goal(s) jointly with the person and consider modifying goal(s) or behavior change strategy in light of achievement. This may lead to re-setting the same goal, a small change in that goal or setting a new goal instead of (or in addition to) the first, or no change	*OTs used behavioral activation techniques (ie, defining a goal and taking steps to achieve it) to reinforce action plans to increase medication adherence. For example, morning medications were placed by a coffee maker and embedded within the morning routine (e.g., "I turn on the coffee maker take my pills and mark the check sheet on the refrigerator."). If pills*

Component	CODE	Description	Example
			needed to be transferred to a pill organizer, the OTs supervised the transfer based on the medication prescription. [119]
1.7 Review outcome goal(s) – tailored/not tailored	T_P_1.7/ NT_P_1.7	Involves a review or analysis of the extent to which previously set outcome goals (e.g., to reduce blood pressure or lose/maintain weight) were achieved. In most cases, this will follow previous goal setting (see technique 6, goal setting-outcome') and an attempt to act on those goals, followed by a revision of goals, and/or means to attain them.	*Intervention patients visited the practice nurse monthly to review their progress and visited the GP 3 monthly and at other times if worsening symptoms demanded more visits*[117]
2.1 Monitoring of behaviour by others – tailored/not tailored	T_P_2.1/ NT_P_2.1	Must be active and in service of changing behaviour, not simply routine data collection that is already in place.	*During training, a qualified exercise specialist closely supervised patients in a ratio of 4 to 5 participants for 1 trainer.*[91]
2.2 Feedback on behaviour – tailored/not tailored	T_P_2.2/ NT_P_2.2	Coded only when there was clear feedback about behaviour. This code implies a monitoring process providing feedback.	*The nurses used this information to provide individualized feedback and reinforcement to participants regarding their use of dyspnea management strategies and exercise progress via email or telephone weekly for the first month and then biweekly for 11 months.*[106]
2.3 Self-monitoring of behaviour – tailored/not tailored	T_P_2.3/ NT_P_2.3	Establish a method for the person to monitor and record their behavior(s) as part of a behavior change strategy	*Participants completed paper diaries [tracking symptoms (dyspnea, cough) and exercise (mode, duration, and worst dyspnea), and reasons for not exercising;] and mailed them back weekly to the study office.*[106]
2.4 Self-monitoring of outcome(s) of behaviour – not tailored	NT_P_2.4	The person is asked to keep a record of specified measures expected to be influenced by the behaviour change, e.g., blood pressure, blood glucose, weight loss, physical fitness.	*Using the touch screen telemonitoring equipment, the participant recorded and transmitted a daily questionnaire about symptoms and use of treatment, and monitored oxygen saturation using linked validated instruments*[115]
2.5 Monitoring of outcome(s) of behaviour without feedback – not tailored	NT_P_2.5	Observe or record outcomes of behavior with the person's knowledge as part of a behavior change strategy	*A written list of recommendations, a weight chart, a contact number available 6 h/day, and an educational booklet were provided only to these patients. They were encouraged to present their discharge/visit summary and weight chart at all visits*[33]
2.7 Feedback on outcome(s) of behaviour – not tailored	NT_P_2.7	Coded only when there is clear feedback about outcomes. This code implies a monitoring process providing the feedback data.	*Patients were provided with feedback on blood pressure and blood levels*[56]

Component	CODE	Description	Example
3.1 Social support (unspecified) – tailored/not tailored	T_P_3.1/ NT_P_3.1	Advise on, arrange or provide social support (e.g., from friends, relatives, colleagues,' buddies' or staff) or non-contingent praise or reward for performance of the behavior.	*During the 20-month active maintenance phase, patients visited the physiotherapist once a month to monitor exercise capacity and adherence to the training and to provide encouragement to continue the exercise training at home.*[147]
3.2 Social support (practical) – tailored/not tailored	T_P_3.2/ NT_P_3.2	Advise on, arrange, or provide practical help (e.g., from friends, relatives, colleagues, 'buddies' or staff) for performance of the behavior	*Home visits (0 to 4) and telephone calls (at least every 3 months) were carried out according to patient need and patient risk level, which was assessed by the study nurse during the first home visit based on compliance, the social network, and the comorbidities.*[98]
4.1 Instruction on how to perform a behaviour – tailored/not tailored	T_P_4.1/ NT_P_4.1	Involves telling the person how to perform behaviour or preparatory behaviours, either verbally or in written form.	*Patients in both groups received an educational packet describing the causes of HF, the basic principles of treatment, their role in routine care and monitoring of their condition, and appropriate strategies for managing a HF exacerbation*[37]
4.2 Information about antecedents - not tailored	NT_P_4.2	Provide information about antecedents (e.g., social and environmental situations and events, emotions, cognitions) that reliably predict performance of the behaviour	*Primary care providers met with patients and discussed how behaviours impacted their current systolic blood pressure results*[56]
5.1 Information about health consequences – tailored/not tailored	T_P_5.1/ NT_P_5.1	Provide information (e.g., written, verbal, visual) about health consequences of performing the behavior	*Subjects in both arms began to receive mailings regarding osteoporosis. During the course of the study, all subjects received 7 informational mailings covering topics such as exercise, fall prevention, and recommended calcium intake*[133]
6.1 Demonstration of the behaviour – not tailored	NT_P_6.1	Provide an observable sample of the performance of the behaviour, directly in person or indirectly e.g., via film, pictures, for the person to aspire to or imitate (includes 'Modelling')	*The class included varied methods such as specialized lectures, small group discussions, physical demonstrations, live presentations, and games with prizes.*[83]
7.1 Prompts/cues – not tailored	NT_P_7.1	Introduce or define environmental or social stimulus with the purpose of prompting or cueing the behavior. The prompt or cue would normally occur at the time or place of performance	…the exercise trainer made weekly telephone calls to reinforce the importance of the exercises and to detect problems...The case manager contacted patients of both groups every 2 months to reinforce mastery of the intended behavior (home exercises 3 times per week).[91]
9.1 Credible source – not tailored	NT_P_9.1	Present verbal or visual communication from a credible source in favour of or against the behavior. Consider national organisations, national guidelines and respected peers as credible sources.	*Input was provided from a pharmacist, cardiac rehabilitation specialist nurse, dietician, welfare benefits advisor and Recreation Services*[28]
10.1 Material incentive (behavior)	NT_P_10.1	Inform that money, vouchers or other valued objects will be delivered if and only if there has been effort and/or progress in performing the behavior (includes 'Positive reinforcement')	*Healthcare logs and diaries were returned monthly in stamped, self-addressed envelopes to the appropriate project director and all patients were compensated $25.00 per visit*[36]
11.1 Pharmacological	T_P_11.1/ NT_P_11.1	Provide, or encourage the use of or adherence to, drugs to facilitate behavior change	*The usual care group received pharmacotherapy according to accepted guidelines*[147]

Component	CODE	Description	Example
support – tailored/not tailored			
11.2 Reduce negative emotions	NT_P_11.2	Advise on ways of reducing negative emotions to facilitate performance of the behavior	*Patients received cognitive coping skills training to identify and challenge negative COPD-related cognitions that impede self management* [152]
12.1 Restructuring the physical environment – not tailored	NT_P_12.1	Change, or advise to change the physical environment in order to facilitate performance of the wanted behavior or create barriers to the unwanted behavior (other than prompts/cues, rewards and punishments)	*Patients received a home assessment which focused on lighting and safety... and modified when necessary*[4]
12.2 Restructuring the social environment – tailored/not tailored	T_P_12.2/ NT_P_12.2	Change, or advise to change the social environment in order to facilitate performance of the wanted behavior or create barriers to the unwanted behavior (other than prompts/cues, rewards and punishments)	*A case manager was added to the team to provide additional support for patients*[41]
12.5 Adding objects to the environment – tailored/not tailored	T_P_12.5/ NT_P_12.5	Code for the addition of new physical or virtual (software) aimed at facilitating behaviour change. If something existing is changed, consider coding to Restructuring physical environment.	*Blood pressure monitors were given to patients to take home.*[56]
12.6 Body changes –tailored/not tailored	T_P_12.6/ NT_P_12.6	Alter body structure, functioning or support directly to facilitate behavior change	*Participants were enrolled in an exercise program to lose weight*[4]
13.1 Umbrella term - Patient education – not tailored	NT_P_13.1	Unclear description	*All participants received a general information booklet for patients with COPD and had access to an existing VA 24-hour help line for any medical questions about their COPD*[41]
14.1 Insufficient description – not tailored	NT_P_14.1	Unclear description	The case management was not well reported[84]

NOTE: Only codes encountered and used more than twice in the data set are reported in this table.

Abbreviations: QI, Quality Improvement

Appendix 8: List of Included Studies

1. Ansari M, Shlipak MG, Heidenreich PA, Van Ostaeyen D, Pohl EC, Browner WS, Massie BM. Improving guideline adherence: a randomized trial evaluating strategies to increase β-blocker use in heart failure. Circulation. 2003 Jun 10;107(22):2799-804.

2. Baker R, Fraser RC, Stone M, Lambert P, Stevenson K, Shiels C. Randomised controlled trial of the impact of guidelines, prioritized review criteria and feedback on implementation of recommendations for angina and asthma. British Journal of General Practice. 2003 Apr 1;53(489):284-91.

3. Batchelor FA, Hill KD, Mackintosh SF, Said CM, Whitehead CH. Effects of a multifactorial falls prevention program for people with stroke returning home after rehabilitation: a randomized controlled trial. Archives of physical medicine and rehabilitation. 2012 Sep 1;93(9):1648-55.

4. Beck A, Scott J, Williams P, Robertson B, Jackson D, Gade G, Cowan P. A randomized trial of group outpatient visits for chronically ill older HMO members: the Cooperative Health Care Clinic. Journal of the American Geriatrics Society. 1997 May;45(5):543-9.

5. Beck CA, Beran DB, Biglan KM, Boyd CM, Dorsey ER, Schmidt PN, Simone R, Willis AW, Galifianakis NB, Katz M, Tanner CM. National randomized controlled trial of virtual house calls for Parkinson disease. Neurology. 2017 Sep 12;89(11):1152-61.

6. Bekelman DB, Plomondon ME, Carey EP, Sullivan MD, Nelson KM, Hattler B, McBryde CF, Lehmann KG, Gianola K, Heidenreich PA, Rumsfeld JS. Primary results of the patient-centered disease management (PCDM) for heart failure study: a randomized clinical trial. JAMA internal medicine. 2015 May 1;175(5):725-32.

7. Benzo R, Vickers K, Novotny PJ, Tucker S, Hoult J, Neuenfeldt P, Connett J, Lorig K, McEvoy C. Health coaching and chronic obstructive pulmonary disease rehospitalization. A randomized study. American journal of respiratory and critical care medicine. 2016 Sep 15;194(6):672-80.

8. Blue L, Lang E, McMurray JJ, Davie AP, McDonagh TA, Murdoch DR, Petrie MC, Connolly E, Norrie J, Round CE, Ford I. Randomised controlled trial of specialist nurse intervention in heart failure. BMJ. 2001 Sep 29;323(7315):715-8.

9. Bohingamu Mudiyanselage S, Stevens J, Watts JJ, Toscano J, Kotowicz MA, Steinfort CL, Bell J, Byrnes J, Bruce S, Carter S, Hunter C. Personalised telehealth intervention for chronic disease management: a pilot randomised controlled trial. Journal of telemedicine and telecare. 2019 Jul;25(6):343-52.

10. Böhm M, Drexler H, Oswald H, Rybak K, Bosch R, Butter C, Klein G, Gerritse B, Monteiro J, Israel C, Bimmel D. Fluid status telemedicine alerts for heart failure: a randomized controlled trial. European Heart Journal. 2016 Nov 1;37(41):3154-63.

11. Bosanquet K, Adamson J, Atherton K, Bailey D, Baxter C, Beresford-Dent J, Birtwistle J, Chew-Graham C, Clare E, Delgadillo J, Ekers D. Collaborative care for screen-positive elders with major depression

(CASPER plus): a multicentred randomised controlled trial of clinical effectiveness and cost-effectiveness. Health Technology Assessment (Winchester, England). 2017 Nov;21(67):1-251.

12. Bourbeau J, Julien M, Maltais F, Rouleau M, Beaupré A, Bégin R, Renzi P, Nault D, Borycki E, Schwartzman K, Singh R. Reduction of hospital utilization in patients with chronic obstructive pulmonary disease: a disease-specific self-management intervention. Archives of internal medicine. 2003 Mar 10;163(5):585-91.

13. Boyne JJ, Vrijhoef HJ, Crijns HJ, De Weerd G, Kragten J, Gorgels AP, TEHAF investigators. Tailored telemonitoring in patients with heart failure: results of a multicentre randomized controlled trial. European journal of heart failure. 2012 Jul;14(7):791-801.

14. Bruce ML, Ten Have TR, Reynolds III CF, Katz II, Schulberg HC, Mulsant BH, Brown GK, McAvay GJ, Pearson JL, Alexopoulos GS. Reducing suicidal ideation and depressive symptoms in depressed older primary care patients: a randomized controlled trial. JAMA. 2004 Mar 3;291(9):1081-91.

15. Bruce ML, Raue PJ, Reilly CF, Greenberg RL, Meyers BS, Banerjee S, Pickett YR, Sheeran TF, Ghesquiere A, Zukowski DM, Rosas VH. Clinical effectiveness of integrating depression care management into medicare home health: the Depression CAREPATH Randomized trial. JAMA Internal Medicine. 2015 Jan 1;175(1):55-64.

16. Bucknall CE, Miller G, Lloyd SM, Cleland J, McCluskey S, Cotton M, Stevenson RD, Cotton P, McConnachie A. Glasgow supported self-management trial (GSuST) for patients with moderate to severe COPD: randomised controlled trial. BMJ. 2012 Mar 6;344.

17. Burns R, Nichols LO, Graney MJ, Cloar FT. Impact of continued geriatric outpatient management on health outcomes of older veterans. Archives of internal medicine. 1995 Jun 26;155(12):1313-8.

18. Burns R, Nichols LO, Martindale-Adams J, Graney MJ, Lummus A. Primary care interventions for dementia caregivers: 2-year outcomes from the REACH study. The Gerontologist. 2003 Aug 1;43(4):547-55.

19. Callahan CM, Boustani MA, Unverzagt FW, Austrom MG, Damush TM, Perkins AJ, Fultz BA, Hui SL, Counsell SR, Hendrie HC. Effectiveness of collaborative care for older adults with Alzheimer disease in primary care: a randomized controlled trial. JAMA. 2006 May 10;295(18):2148-57.

20. Campbell NC, Ritchie LD, Thain J, Deans HG, Rawles JM, Squair JL. Secondary prevention in coronary heart disease: a randomised trial of nurse led clinics in primary care. Heart. 1998 Nov 1;80(5):447-52.

21. Chen S, Conwell Y, He J, Lu N, Wu J. Depression care management for adults older than 60 years in primary care clinics in urban China: a cluster-randomised trial. The Lancet Psychiatry. 2015 Apr 1;2(4):332-9.

22. Chi C, Chen H. Daily-based self-management for non-hospitalised heart failure patients improve prognosis. Heart. 2012 Oct 1;98(Suppl 2):E231-2.

23. Ciaschini PM, Straus SE, Dolovich LR, Goeree RA, Leung KM, Woods CR, Zimmerman GM, Majumdar SR, Spadafora S, Fera LA, Lee HN. Community based intervention to optimize osteoporosis management: randomized controlled trial. BMC geriatrics. 2010 Dec;10(1):1-7.

24. Cleveringa FG, Gorter KJ, Van Den Donk M, Rutten GE. Combined task delegation, computerized decision support, and feedback improve cardiovascular risk for type 2 diabetic patients: a cluster randomized trial in primary care. Diabetes care. 2008 Dec 1;31(12):2273-5.

25. Coleman EA, Grothaus LC, Sandhu N, Wagner EH. Chronic care clinics: a randomized controlled trial of a new model of primary care for frail older adults. Journal of the American Geriatrics Society. 1999 Jul;47(7):775-83.

26. Community Pharmacy Medicines Management Project Evaluation Team. The MEDMAN study: a randomized controlled trial of community pharmacy-led medicines management for patients with coronary heart disease. Family Practice. 2007 Apr 1;24(2):189-200.

27. Coull AJ, Taylor VH, Elton R, Murdoch PS, Hargreaves AD. A randomised controlled trial of senior Lay Health Mentoring in older people with ischaemic heart disease: The Braveheart Project. Age and ageing. 2004 Jul 1;33(4):348-54.

28. de Fine Olivarius N, Beck-Nielsen H, Andreasen AH, Hørder M, Pedersen PA. Randomised controlled trial of structured personal care of type 2 diabetes mellitus. BMJ. 2001 Oct 27;323(7319):970-5.

29. de la Porte PW, Lok DJ, van Veldhuisen DJ, van Wijngaarden J, Cornel JH, Zuithoff NP, Badings E, Hoes AW. Added value of a physician-and-nurse-directed heart failure clinic: results from the Deventer–Alkmaar heart failure study. Heart. 2007 Jul 1;93(7):819-25.

30. De Lusignana S, Gallagher H, Jones S, Chan T, Van Vlymen J, Tahir A, Thomas N, Jain N, Dmitrieva O, Rafi I, McGovern A. Audit-based education lowers systolic blood pressure in chronic kidney disease: the Quality Improvement in CKD (QICKD) trial results. Kidney international. 2013 Sep 1;84(3):609-20.

31. DeBusk RF, Miller NH, Parker KM, Bandura A, Kraemer HC, Cher DJ, West JA, Fowler MB, Greenwald G. Care management for low-risk patients with heart failure: a randomized, controlled trial. Annals of Internal Medicine. 2004 Oct 19;141(8):606-13.

32. Del Sindaco D, Pulignano G, Minardi G, Apostoli A, Guerrieri L, Rotoloni M, Petri G, Fabrizi L, Caroselli A, Venusti R, Chiantera A. Two-year outcome of a prospective, controlled study of a disease management programme for elderly patients with heart failure. Journal of Cardiovascular Medicine. 2007 May 1;8(5):324-9.

33. DeVore AD, Cox M, Heidenreich PA, Fonarow GC, Yancy CW, Eapen ZJ, Peterson ED, Hernandez AF. Cluster-randomized trial of personalized site performance feedback in get with the guidelines-heart failure. Circulation: Cardiovascular Quality and Outcomes. 2015 Jul;8(4):421-7.

34. Döpp CM, Graff MJ, Teerenstra S, Olde Rikkert MG, Nijhuis–van der Sanden MW, Vernooij-Dassen MJ. Effectiveness of a training package for implementing a community-based occupational therapy

program in dementia: a cluster randomized controlled trial. Clinical Rehabilitation. 2015 Oct;29(10):974-86.

35. Dracup K, Moser DK, Pelter MM, Nesbitt TS, Southard J, Paul SM, Robinson S, Cooper LS. Randomized, controlled trial to improve self-care in patients with heart failure living in rural areas. Circulation. 2014 Jul 15;130(3):256-64.

36. Dunagan WC, Littenberg B, Ewald GA, Jones CA, Emery VB, Waterman BM, Silverman DC, Rogers JG. Randomized trial of a nurse-administered, telephone-based disease management program for patients with heart failure. Journal of cardiac failure. 2005 Jun 1;11(5):358-65.

37. Eccles MP, Whitty PM, Speed C, Steen IN, Vanoli A, Hawthorne GC, Grimshaw JM, Wood LJ, McDowell D. A pragmatic cluster randomised controlled trial of a Diabetes REcall And Management system: the DREAM trial. Implementation Science. 2007 Dec;2(1):1-2.

38. Eckert K, Schrader G, Wilkinson D, Askew D, Dick M, Wade T, Marwick T, Scuffham P, Jackson C, Schluter P, Stewart S. Detection and management of depression in patients with chronic heart disease: the TAKE heart in primary care cluster randomised controlled trial. Heart, Lung and Circulation. 2010 Jan 1;19:S240-1.

39. Ell K, Unützer J, Aranda M, Gibbs NE, Lee PJ, Xie B. Managing depression in home health care: a randomized clinical trial. Home health care services quarterly. 2007 Jul 12;26(3):81-104.

40. Fan VS, Gaziano JM, Lew R, Bourbeau J, Adams SG, Leatherman S, Thwin SS, Huang GD, Robbins R, Sriram PS, Sharafkhaneh A. A comprehensive care management program to prevent chronic obstructive pulmonary disease hospitalizations: a randomized, controlled trial. Annals of internal medicine. 2012 May 15;156(10):673-83.

41. Federman AD, O’Conor R, Mindlis I, Hoy-Rosas J, Hauser D, Lurio J, Shroff N, Lopez R, Erblich J, Wolf MS, Wisnivesky JP. Effect of a self-management support intervention on asthma outcomes in older adults: the SAMBA study randomized clinical trial. JAMA internal medicine. 2019 Aug 1;179(8):1113-21.

42. Fihn SD, McDonell MB, Diehr P, Anderson SM, Bradley KA, Au DH, Spertus JA, Burman M, Reiber GE, Kiefe CI, Cody M. Effects of sustained audit/feedback on self-reported health status of primary care patients. The American journal of medicine. 2004 Feb 15;116(4):241-8.

43. Fihn SD, Bucher JB, McDonell M, Diehr P, Rumsfeld JS, Doak M, Dougherty C, Gerrity M, Heidenreich P, Larsen G, Lee PI. Collaborative care intervention for stable ischemic heart disease. Archives of internal medicine. 2011 Sep 12;171(16):1471-9.

44. Forster A, Young J. Specialist nurse support for patients with stroke in the community: a randomised controlled trial. BMJ. 1996 Jun 29;312(7047):1642-6.

45. Forster A, Young J, Chapman K, Nixon J, Patel A, Holloway I, Mellish K, Anwar S, Breen R, Knapp M, Murray J. Cluster randomized controlled trial: clinical and cost-effectiveness of a system of longer-term stroke care. Stroke. 2015 Aug;46(8):2212-9.

46. Fortinsky RH, Kulldorff M, Kleppinger A, Kenyon-Pesce L. Dementia care consultation for family caregivers: collaborative model linking an Alzheimer's association chapter with primary care physicians. Aging and Mental Health. 2009 Mar 1;13(2):162-70.

47. Freund T, Peters-Klimm F, Boyd CM, Mahler C, Gensichen J, Erler A, Beyer M, Gondan M, Rochon J, Gerlach FM, Szecsenyi J. Medical assistant–based care management for high-risk patients in small primary care practices: a cluster randomized clinical trial. Annals of internal medicine. 2016 Mar 1;164(5):323-30.

48. Galbreath AD, Krasuski RA, Smith B, Stajduhar KC, Kwan MD, Ellis R, Freeman GL. Long-term healthcare and cost outcomes of disease management in a large, randomized, community-based population with heart failure. Circulation. 2004 Dec 7;110(23):3518-26.

49. Gallagher RA, Miller C, Cronan TA, Groessl E. Gender differences in participation and responsiveness to a health intervention for older Americans. Women & Health. 1997 Jul 24;25(3):63-81.

50. Gaugler JE, Roth DL, Haley WE, Mittelman MS. Can counseling and support reduce Alzheimer's caregivers' burden and depressive symptoms during the transition to institutionalization? Results from the NYU caregiver intervention study. Journal of the American Geriatrics Society. 2008 Mar;56(3):421.

51. Gellis ZD, Kenaley B, McGinty J, Bardelli E, Davitt J, Ten Have T. Outcomes of a telehealth intervention for homebound older adults with heart or chronic respiratory failure: a randomized controlled trial. The Gerontologist. 2012 Aug 1;52(4):541-52.

52. Goderis G, Borgermans L, Grol R, Van Den Broeke C, Boland B, Verbeke G, Carbonez A, Mathieu C, Heyrman J. Start improving the quality of care for people with type 2 diabetes through a general practice support program: a cluster randomized trial. Diabetes research and clinical practice. 2010 Apr 1;88(1):56-64.

53. Graven C, Brock K, Hill KD, Cotton S, Joubert L. First year after stroke: an integrated approach focusing on participation goals aiming to reduce depressive symptoms. Stroke. 2016 Nov;47(11):2820-7.

54. Haerter M, Dirmaier J, Dwinger S, Kriston L, Herbarth L, Siegmund-Schultze E, Bermejo I, Matschinger H, Heider D, König HH. Effectiveness of telephone-based health coaching for patients with chronic conditions: a randomised controlled trial. PLoS ONE 2016 Sep 15;11(9):e0161269.

55. Heisler M, Hofer TP, Schmittdiel JA, Selby JV, Klamerus ML, Bosworth HB, Bermann M, Kerr EA. Improving blood pressure control through a clinical pharmacist outreach program in patients with diabetes mellitus in 2 high-performing health systems: the adherence and intensification of medications cluster randomized, controlled pragmatic trial. Circulation. 2012 Jun 12;125(23):2863-72.

56. Hendriks JM, De Wit R, Crijns HJ, Vrijhoef HJ, Prins MH, Pisters R, Pison LA, Blaauw Y, Tieleman RG. Nurse-led care vs. usual care for patients with atrial fibrillation: results of a randomized trial of integrated chronic care vs. routine clinical care in ambulatory patients with atrial fibrillation. European heart journal. 2012 Nov 1;33(21):2692-9.

57. Hernández C, Alonso A, Garcia-Aymerich J, Serra I, Marti D, Rodriguez-Roisin R, Narsavage G, Carmen Gomez M, Roca J. Effectiveness of community-based integrated care in frail COPD patients: a randomised controlled trial. NPJ primary care respiratory medicine. 2015 Apr 9;25(1):1-6.

58. Hetlevik I, Holmen J, Krüger Ø, Kristensen P, Iversen H, Furuseth K. Implementing clinical guidelines in the treatment of diabetes mellitus in general practice: Evaluation of effort, process, and patient outcome related to implementation of a computer-based decision support system. International journal of technology assessment in health care. 2000 Jan;16(1):210-27.

59. Hogg W, Lemelin J, Dahrouge S, Liddy C, Armstrong CD, Legault F, Dalziel B, Zhang W. Randomized controlled trial of anticipatory and preventive multidisciplinary team care: for complex patients in a community-based primary care setting. Canadian family physician. 2009 Dec 1;55(12):e76-85.

60. Holbrook A, Pullenayegum E, Thabane L, Troyan S, Foster G, Keshavjee K, Chan D, Dolovich L, Gerstein H, Demers C, Curnew G. Shared electronic vascular risk decision support in primary care: Computerization of Medical Practices for the Enhancement of Therapeutic Effectiveness (COMPETE III) randomized trial. Archives of Internal Medicine. 2011 Oct 24;171(19):1736-44.

61. Holm T, Lassen JF, Husted SE, Christensen P, Heickendorff L. A randomized controlled trial of shared care versus routine care for patients receiving oral anticoagulant therapy. Journal of internal medicine. 2002 Oct;252(4):322-31.

62. Holt TA, Thorogood M, Griffiths F, Munday S, Friede T, Stables D. Automated electronic reminders to facilitate primary cardiovascular disease prevention: randomised controlled trial. British Journal of General Practice. 2010 Apr 1;60(573):e137-43.

63. Hughes SL, Weaver FM, Giobbie-Hurder A, Manheim L, Henderson W, Kubal JD, Ulasevich A, Cummings J, Department of Veterans Affairs Cooperative Study Group on Home-Based Primary Care, Department of Veterans Affairs Cooperative Study Group on Home-Based Primary Care. Effectiveness of team-managed home-based primary care: a randomized multicenter trial. JAMA. 2000 Dec 13;284(22):2877-85.

64. Hunger M, Kirchberger I, Holle R, Seidl H, Kuch B, Wende R, Meisinger C. Does nurse-based case management for aged myocardial infarction patients improve risk factors, physical functioning and mental health? The KORINNA trial. European journal of preventive cardiology. 2015 Apr 1;22(4):442-50.

65. Irewall AL, Ögren J, Bergström L, Laurell K, Söderström L, Mooe T. Nurse-led, telephone-based, secondary preventive follow-up after stroke or transient ischemic attack improves blood pressure and LDL cholesterol: results from the first 12 months of the randomized, controlled NAILED stroke risk factor trial. PLoS ONE. 2015 Oct 16;10(10):e0139997.

66. Jaarsma T, van der Wal MH, Lesman-Leegte I, Luttik ML, Hogenhuis J, Veeger NJ, Sanderman R, Hoes AW, van Gilst WH, Lok DJ, Dunselman PH. Effect of moderate or intensive disease management program on outcome in patients with heart failure: Coordinating Study Evaluating Outcomes of Advising and Counseling in Heart Failure (COACH). Archives of internal medicine. 2008 Feb 11;168(3):316-24.

67. Joling KJ, van Marwijk HW, Smit F, van der Horst HE, Scheltens P, van de Ven PM, Mittelman MS, van Hout HP. Does a family meetings intervention prevent depression and anxiety in family caregivers of dementia patients? A randomized trial. PloS one. 2012 Jan 27;7(1):e30936.

68. Kalter-Leibovici O, Freimark D, Freedman LS, Kaufman G, Ziv A, Murad H, Benderly M, Silverman BG, Friedman N, Cukierman-Yaffe T, Asher E. Disease management in the treatment of patients with chronic heart failure who have universal access to health care: a randomized controlled trial. BMC medicine. 2017 Dec;15(1):1-3.

69. Karhula T, Vuorinen AL, Rääpysjärvi K, Pakanen M, Itkonen P, Tepponen M, Junno UM, Jokinen T, Van Gils M, Lähteenmäki J, Kohtamäki K. Telemonitoring and mobile phone-based health coaching among Finnish diabetic and heart disease patients: randomized controlled trial. Journal of medical Internet research. 2015 Jun 17;17(6):e4059.

70. Kennedy A, Bower P, Reeves D, Blakeman T, Bowen R, Chew-Graham C, Eden M, Fullwood C, Gaffney H, Gardner C, Lee V. Implementation of self management support for long term conditions in routine primary care settings: cluster randomised controlled trial. BMJ. 2013 May 13;346.

71. Kennedy CC, Ioannidis G, Thabane L, Adachi JD, Marr S, Giangregorio LM, Morin SN, Crilly RG, Josse RG, Lohfeld L, Pickard LE. Successful knowledge translation intervention in long-term care: final results from the vitamin D and osteoporosis study (ViDOS) pilot cluster randomized controlled trial. Trials. 2015 Dec;16(1):1-1.

72. Khdour MR, Kidney JC, Smyth BM, McElnay JC. Clinical pharmacy-led disease and medicine management programme for patients with COPD. British Journal of Clinical Pharmacology. 2009 Oct;68(4):588-98.

73. Kiefe CI, Allison JJ, Williams OD, Person SD, Weaver MT, Weissman NW. Improving quality improvement using achievable benchmarks for physician feedback: a randomized controlled trial. JAMA. 2001 Jun 13;285(22):2871-9.

74. Kim KB, Han HR, Huh B, Nguyen T, Lee H, Kim MT. The effect of a community-based self-help multimodal behavioral intervention in Korean American seniors with high blood pressure. American journal of hypertension. 2014 Sep 1;27(9):1199-208.

75. Ko FW, Cheung NK, Rainer TH, Lum C, Wong I, Hui DS. Comprehensive care programme for patients with chronic obstructive pulmonary disease: a randomised controlled trial. Thorax. 2017 Feb 1;72(2):122-8.

76. Kruis AL, Boland MR, Assendelft WJ, Gussekloo J, Tsiachristas A, Stijnen T, Blom C, Sont JK, Rutten-van Mölken MP, Chavannes NH. Effectiveness of integrated disease management for primary care chronic obstructive pulmonary disease patients: results of cluster randomised trial. BMJ. 2014 Sep 10;349.

77. Krum H, Forbes A, Yallop J, Driscoll A, Croucher J, Chan B, Clark R, Davidson P, Huynh L, Kasper EK, Hunt D. Telephone support to rural and remote patients with heart failure: the Chronic Heart Failure Assessment by Telephone (CHAT) study. Cardiovascular Therapeutics. 2013 Aug;31(4):230-7.

78. Kurz A, Wagenpfeil S, Hallauer J, Schneider-Schelte H, Jansen S, AENEAS Study Group. Evaluation of a brief educational program for dementia carers: The AENEAS Study. International journal of geriatric psychiatry. 2010 Aug;25(8):861-9.

79. Leveille SG, Wagner EH, Davis C, Grothaus L, Wallace J, LoGerfo M, Kent D. Preventing disability and managing chronic illness in frail older adults: a randomized trial of a community-based partnership with primary care. Journal of the American Geriatrics Society. 1998 Oct;46(10):1191-8.

80. Leventhal ME, Denhaerynck K, Brunner-La Rocca HP, Burnand B, Conca A, Bernasconi AT, Mahrer-Imhof R, Sivarajan Froelicher E, De Geest S. Swiss Interdisciplinary Management Programme for Heart Failure (SWIM-HF): a randomised controlled trial study of an outpatient inter-professional management programme for heart failure patients in Switzerland. Swiss Medical Weekly. 2011 Mar 8;141(0910).

81. Levine DA, Funkhouser EM, Houston TK, Gerald JK, Johnson-Roe N, Allison JJ, Richman J, Kiefe CI. Improving Care After Myocardial Infarction Using a 2-Year Internet-Delivered Intervention: The Department of Veterans Affairs Myocardial Infarction–Plus Cluster-Randomized Trial. Archives of internal medicine. 2011 Nov 28;171(21):1910-7.

82. Li X, Zhou Q, Zou F, Wu L, Chen H, Liu Z. Effectiveness of systematic self-management education on blood sugar level of patients in the community with type 2 diabetes. Zhong nan da xue xue bao. Yi xue ban [Journal of Central South University. Medical Sciences]. 2012 Apr 1;37(4):355-8.

83. Licskai C, Ferrone M, Malus N, Stitt L, O'Callahan T, Roberts Z, Johnson L, Samson J, Durocher L. COPD collaborative self-management in primary care: A randomized controlled trial. European Respiratory Journal. Conference: European Respiratory Society Annual Congress. 2016; 48(no pagination).

84. Lin HW, Lin CH, Chang CK, Chou CY, Yu IW, Lin CC, Li TC, Li CI, Hsieh YW. Economic outcomes of pharmacist-physician medication therapy management for polypharmacy elderly: a prospective, randomized, controlled trial. Journal of the Formosan Medical Association. 2018 Mar 1;117(3):235-43.

85. Liu WT, Wang CH, Lin HC, Lin SM, Lee KY, Lo YL, Hung SH, Chang YM, Chung KF, Kuo HP. Efficacy of a cell phone-based exercise programme for COPD. European Respiratory Journal. 2008 Sep 1;32(3):651-9.

86. López Cabezas C, Falces Salvador C, Cubí Quadrada D, Arnau Bartés A, Ylla Bore M, Muro Perea N, Homs Peipoch E. Randomized clinical trial of a postdischarge pharmaceutical care program vs regular follow-up in patients with heart failure. Farmacia Hospitalaria. 2006;30(6):328-42.

87. Lowrie R, Lloyd SM, McConnachie A, Morrison J. A cluster randomised controlled trial of a pharmacist-led collaborative intervention to improve statin prescribing and attainment of cholesterol targets in primary care. PLoS ONE. 2014 Nov 18;9(11):e113370.

88. Machline-Carrion MJ, Soares RM, Damiani LP, Campos VB, Sampaio B, Fonseca FH, Izar MC, Amodeo C, Pontes-Neto OM, Santos JY, do Carmo Gomes SP. Effect of a multifaceted quality improvement intervention on the prescription of evidence-based treatment in patients at high cardiovascular risk in

Brazil: the bridge cardiovascular prevention cluster randomized clinical trial. JAMA cardiology. 2019 May 1;4(5):408-17.

89. Mahoney DF, Tarlow BJ, Jones RN. Effects of an automated telephone support system on caregiver burden and anxiety: findings from the REACH for TLC intervention study. The Gerontologist. 2003 Aug 1;43(4):556-67.

90. Maltais F, Bourbeau J, Shapiro S, Lacasse Y, Perrault H, Baltzan M, Hernandez P, Rouleau M, Julien M, Parenteau S, Paradis B. Effects of home-based pulmonary rehabilitation in patients with chronic obstructive pulmonary disease: a randomized trial. Annals of internal medicine. 2008 Dec 16;149(12):869-78.

91. Markle-Reid M, Orridge C, Weir R, Browne G, Gafni A, Lewis M, Walsh M, Levy C, Daub S, Brien H, Roberts J. Interprofessional stroke rehabilitation for stroke survivors using home care. Canadian Journal of Neurological Sciences. 2011 Mar;38(2):317-34.

92. Markun S, Dishy A, Neuner-Jehle S, Rosemann T, Frei A. The chronic care for wet age related macular degeneration (CHARMED) study: a randomized controlled trial. PLoS ONE. 2015 Nov 16;10(11):e0143085.

93. McCluskey A, Ada L, Kelly PJ, Middleton S, Goodall S, Grimshaw JM, Logan P, Longworth M, Karageorge A. A behavior change program to increase outings delivered during therapy to stroke survivors by community rehabilitation teams: The Out-and-About trial. International Journal of Stroke. 2016 Jun;11(4):425-37.

94. McElrath M, Myers J, Chan K, Fonda H. Exercise adherence in the elderly: Experience with abdominal aortic aneurysm simple treatment and prevention. Journal Of Vascular Nursing. 2017 Mar 1;35(1):12-20.

95. McManus RJ, Mant J, Haque MS, Bray EP, Bryan S, Greenfield SM, Jones MI, Jowett S, Little P, Penaloza C, Schwartz C. Effect of self-monitoring and medication self-titration on systolic blood pressure in hypertensive patients at high risk of cardiovascular disease: the TASMIN-SR randomized clinical trial. JAMA. 2014 Aug 27;312(8):799-808.

96. Meeuwsen EJ, Melis RJ, Van Der Aa GC, Golüke-Willemse GA, De Leest BJ, Van Raak FH, Schölzel-Dorenbos CJ, Verheijen DC, Verhey FR, Visser MC, Wolfs CA. Effectiveness of dementia follow-up care by memory clinics or general practitioners: randomised controlled trial. BMJ. 2012 May 15;344.

97. Meisinger C, Stollenwerk B, Kirchberger I, Seidl H, Wende R, Kuch B, Holle R. Effects of a nurse-based case management compared to usual care among aged patients with myocardial infarction: results from the randomized controlled KORINNA study. BMC geriatrics. 2013 Dec;13(1):1-0.

98. Mejhert M, Kahan T, Persson H, Edner M. Limited long term effects of a management programme for heart failure. Heart. 2004 Sep 1;90(9):1010-5.

99. Mitchell E, Sullivan F, Grimshaw JM, Donnan PT, Watt G. Improving management of hypertension in general practice: a randomised controlled trial of feedback derived from electronic patient data. British Journal of General Practice. 2005 Feb 1;55(511):94-101.

100. Moher M, Yudkin P, Wright L, Turner R, Fuller A, Schofield T, Mant D. Cluster randomised controlled trial to compare three methods of promoting secondary prevention of coronary heart disease in primary care. BMJ. 2001 Jun 2;322(7298):1338.

101. Morganroth M, Pape G, Rozenfeld Y, Heffner JE. Multidisciplinary COPD disease management program: impact on clinical outcomes. Postgraduate Medicine. 2016 Feb 17;128(2):239-49.

102. Moriyama M, Nakano M, Kuroe Y, Nin K, Niitani M, Nakaya T. Efficacy of a self-management education program for people with type 2 diabetes: results of a 12 month trial. Japan Journal of Nursing Science. 2009 Jun;6(1):51-63.

103. Moy ML, Martinez CH, Kadri R, Roman P, Holleman RG, Kim HM, Nguyen HQ, Cohen MD, Goodrich DE, Giardino ND, Richardson CR. Long-term effects of an internet-mediated pedometer-based walking program for chronic obstructive pulmonary disease: randomized controlled trial. Journal of Medical Internet Research. 2016 Aug 8;18(8):e5622.

104. Murphy AW, Cupples ME, Smith SM, Byrne M, Byrne MC, Newell J. Effect of tailored practice and patient care plans on secondary prevention of heart disease in general practice: cluster randomised controlled trial. BMJ. 2009 Oct 29;339.

105. Nguyen HQ, Donesky D, Reinke LF, Wolpin S, Chyall L, Benditt JO, Paul SM, Carrieri-Kohlman V. Internet-based dyspnea self-management support for patients with chronic obstructive pulmonary disease. Journal of Pain and Symptom Management. 2013 Jul 1;46(1):43-55.

106. Ojeda S, Anguita M, Delgado M, Atienza F, Rus C, Granados AL, Ridocci F, Vallés F, Velasco JA. Short-and long-term results of a programme for the prevention of readmissions and mortality in patients with heart failure: are effects maintained after stopping the programme?. European journal of heart failure. 2005 Aug;7(5):921-6.

107. Olaiya MT, Kim J, Nelson MR, Srikanth VK, Bladin CF, Gerraty RP, Fitzgerald SM, Phan T, Frayne J, Cadilhac DA, Thrift AG. Effectiveness of a shared team approach between nurses and doctors for improved risk factor management in survivors of stroke: a cluster randomized controlled trial. European Journal of Neurology. 2017 Jul;24(7):920-8.

108. Olson KL, Delate T, Rasmussen J, Humphries TL, Merenich JA. Outcomes of patients discharged from pharmacy-managed cardiovascular disease management. The American Journal of Managed Care. 2009 Aug 1;15(8):497-503.

109. Ortiz-Bautista C, Diaz M, Delgado-Nicolas MA, Moran-Fernandez L, De Juan-Baguda J, Ponz I, Rodriguez-Chaverri A, Lora-Pablos D, Garcia-Cosio MD, Garcia-Cosio MD, Delgado-Jimenez J. Evaluation of a nurse-led cross intervention program in heart failure. European Journal of Heart Failure. 2017; 19 (Supplement 1): 306-7.

110. Ostwald SK, Godwin KM, Cron SG, Kelley CP, Hersch G, Davis S. Home-based psychoeducational and mailed information programs for stroke-caregiving dyads post-discharge: a randomized trial. Disability and Rehabilitation. 2014 Jan 1;36(1):55-62.

111. Palacio AM, Uribe C, Hazel-Fernandez L, Li H, Tamariz LJ, Garay SD, Carrasquillo O. Can phone-based motivational interviewing improve medication adherence to antiplatelet medications after a coronary stent among racial minorities? A randomized trial. Journal of General Internal Medicine. 2015 Apr;30(4):469-75.

112. Peters-Klimm F, Campbell S, Hermann K, Kunz CU, Müller-Tasch T, Szecsenyi J. Case management for patients with chronic systolic heart failure in primary care: the HICMan exploratory randomised controlled trial. Trials. 2010 Dec;11(1):1-4.

113. Piette JD, Striplin D, Marinec N, Chen J, Trivedi RB, Aron DC, Fisher L, Aikens JE. A mobile health intervention supporting heart failure patients and their informal caregivers: a randomized comparative effectiveness trial. Journal of Medical Internet Research. 2015 Jun 10;17(6):e4550.

114. Pinnock H, Hanley J, McCloughan L, Todd A, Krishan A, Lewis S, Stoddart A, van der Pol M, MacNee W, Sheikh A, Pagliari C. Effectiveness of telemonitoring integrated into existing clinical services on hospital admission for exacerbation of chronic obstructive pulmonary disease: researcher blind, multicentre, randomised controlled trial. BMJ. 2013 Oct 17;347.

115. Pols AD, Van Dijk SE, Bosmans JE, Hoekstra T, Van Marwijk HW, Van Tulder MW, Adriaanse MC. Effectiveness of a stepped-care intervention to prevent major depression in patients with type 2 diabetes mellitus and/or coronary heart disease and subthreshold depression: a pragmatic cluster randomized controlled trial. PLoS ONE. 2017 Aug 1;12(8):e0181023.

116. Rea H, McAuley S, Stewart A, Lamont C, Roseman P, Didsbury P. A chronic disease management programme can reduce days in hospital for patients with chronic obstructive pulmonary disease. Internal medicine journal. 2004 Nov;34(11):608-14.

117. Reiber GE, Au D, McDonell M, Fihn SD. Diabetes quality improvement in Department of Veterans Affairs Ambulatory Care Clinics: a group-randomized clinical trial. Diabetes Care. 2004 May 1;27(suppl_2):b61-8.

118. Rovner BW, Casten RJ, Piersol CV, White N, Kelley M, Leiby BE. Improving glycemic control in African Americans with diabetes and mild cognitive impairment. Journal of the American Geriatrics Society. 2020 May;68(5):1015-22.

119. Rubenstein LZ, Alessi CA, Josephson KR, Trinidad Hoyl M, Harker JO, Pietruszka FM. A randomized trial of a screening, case finding, and referral system for older veterans in primary care. Journal of the American Geriatrics Society. 2007 Feb;55(2):166-74.

120. Saal S, Becker C, Lorenz S, Schubert M, Kuss O, Stang A, Müller T, Kraft A, Behrens J. Effect of a stroke support service in Germany: a randomized trial. Topics in Stroke Rehabilitation. 2015 Aug 18;22(6):429-36.

121. Sackley CM, Walker MF, Burton CR, Watkins CL, Mant J, Roalfe AK, Wheatley K, Sheehan B, Sharp L, Stant KE, Fletcher-Smith J. An occupational therapy intervention for residents with stroke related disabilities in UK care homes (OTCH): cluster randomised controlled trial. BMJ. 2015 Feb 5;350.

122. Salinero-Fort MA, Pau EC, Arrieta-Blanco FJ, Abanades-Herranz JC, Martín-Madrazo C, Rodés-Soldevila B, de Burgos-Lunar C. Effectiveness of PRECEDE model for health education on changes and level of control of HbA1c, blood pressure, lipids, and body mass index in patients with type 2 diabetes mellitus. BMC public health. 2011 Dec;11(1):1-9.

123. Salisbury C, Man MS, Bower P, Guthrie B, Chaplin K, Gaunt DM, Brookes S, Fitzpatrick B, Gardner C, Hollinghurst S, Lee V. Management of multimorbidity using a patient-centred care model: a pragmatic cluster-randomised trial of the 3D approach. The Lancet. 2018 Jul 7;392(10141):41-50.

124. Samus QM, Johnston D, Black BS, Hess E, Lyman C, Vavilikolanu A, Pollutra J, Leoutsakos JM, Gitlin LN, Rabins PV, Lyketsos CG. A multidimensional home-based care coordination intervention for elders with memory disorders: the maximizing independence at home (MIND) pilot randomized trial. The American Journal of Geriatric Psychiatry. 2014 Apr 1;22(4):398-414.

125. Sánchez-Nieto JM, Andújar-Espinosa R, Bernabeu-Mora R, Hu C, Gálvez-Martínez B, Carrillo-Alcaraz A, Álvarez-Miranda CF, Meca-Birlanga O, Abad-Corpa E. Efficacy of a self-management plan in exacerbations for patients with advanced COPD. International journal of chronic obstructive pulmonary disease. 2016;11:1939.

126. Sarkadi A, Rosenqvist U. Experience-based group education in Type 2 diabetes: a randomised controlled trial. Patient education and counseling. 2004 Jun 1;53(3):291-8.

127. Schäfer I, Kaduszkiewicz H, Mellert C, Löffler C, Mortsiefer A, Ernst A, Stolzenbach CO, Wiese B, Abholz HH, Scherer M, van den Bussche H. Narrative medicine-based intervention in primary care to reduce polypharmacy: results from the cluster-randomised controlled trial MultiCare AGENDA. BMJ open. 2018 Jan 1;8(1):e017653.

128. Schrader G, Cheok F, Hordacre AL, Marker J, Wade V. Effect of psychiatry liaison with general practitioners on depression severity in recently hospitalised cardiac patients: a randomised controlled trial. Medical journal of Australia. 2005 Mar;182(6):272-6.

129. Schraeder C, Fraser C, Clark I, Newcomer R, Stoll J, Krock C, Shelton P. The effect of primary care management on lipids testing and LDL-C control of elderly patients with comorbidities. Professional Case Management. 2009 Mar 1;14(2):84-95.

130. Shea S, Weinstock RS, Starren J, Teresi J, Palmas W, Field L, Morin P, Goland R, Izquierdo RE, Wolff LT, Ashraf M. A randomized trial comparing telemedicine case management with usual care in older, ethnically diverse, medically underserved patients with diabetes mellitus. Journal of the American Medical Informatics Association. 2006 Jan 1;13(1):40-51.

131. Smith BJ, Appleton SL, Bennett PW, Roberts GC, Fante PD, Adam R, Trott CM, Allan DP, Southcott AM, Ruffin RE. The effect of a respiratory home nurse intervention in patients with chronic obstructive pulmonary disease (COPD). Australian and New Zealand journal of medicine. 1999 Oct;29(5):718-25.

132. Solomon DH, Iversen MD, Avorn J, Gleeson T, Brookhart MA, Patrick AR, Rekedal L, Shrank WH, Lii J, Losina E, Katz JN. Osteoporosis telephonic intervention to improve medication regimen adherence: a large, pragmatic, randomized controlled trial. Archives of Internal Medicine. 2012 Mar 26;172(6):477-83.

133. Sönnichsen AC, Winkler H, Flamm M, Panisch S, Kowatsch P, Klima G, Fürthauer B, Weitgasser R. The effectiveness of the Austrian disease management programme for type 2 diabetes: a cluster-randomised controlled trial. BMC family practice. 2010 Dec;11(1):1-0.

134. Stewart S, Carrington MJ, Marwick TH, Davidson PM, Macdonald P, Horowitz JD, Krum H, Newton PJ, Reid C, Chan YK, Scuffham PA. Impact of home versus clinic-based management of chronic heart failure: the WHICH?(Which Heart Failure Intervention Is Most Cost-Effective & Consumer Friendly in Reducing Hospital Care) multicenter, randomized trial. Journal of the American College of Cardiology. 2012 Oct 2;60(14):1239-48.

135. Stewart S, Ball J, Horowitz JD, Marwick TH, Mahadevan G, Wong C, Abhayaratna WP, Chan YK, Esterman A, Thompson DR, Scuffham PA. Standard versus atrial fibrillation-specific management strategy (SAFETY) to reduce recurrent admission and prolong survival: pragmatic, multicentre, randomised controlled trial. The Lancet. 2015 Feb 28;385(9970):775-84.

136. Subramanian U, Fihn SD, Weinberger M, Plue L, Smith FE, Udris EM, McDonell MB, Eckert GJ, Temkit MH, Zhou XH, Chen L. A controlled trial of including symptom data in computer-based care suggestions for managing patients with chronic heart failure. The American Journal of Medicine. 2004 Mar 15;116(6):375-84.

137. Suominen MH, Puranen TM, Jyväkorpi SK, Eloniemi-Sulkava U, Kautiainen H, Siljamäki-Ojansuu U, Pitkalä KH. Nutritional guidance improves nutrient intake and quality of life, and may prevent falls in aged persons with Alzheimer disease living with a spouse (NuAD trial). The journal of nutrition, health & aging. 2015 Nov;19(9):901-7.

138. Tjia J, Field T, Mazor K, Lemay CA, Kanaan AO, Donovan JL, Briesacher BA, Peterson D, Pandolfi M, Spenard A, Gurwitz JH. Dissemination of evidence-based antipsychotic prescribing guidelines to nursing homes: a cluster randomized trial. Journal of the American Geriatrics Society. 2015 Jul;63(7):1289-98.

139. Tomita MR, Tsai BM, Fisher NM, Kumar NA, Wilding G, Stanton K, Naughton BJ. Effects of multidisciplinary Internet-based program on management of heart failure. Journal of Multidisciplinary Healthcare. 2009;2:13.

140. Tremont G, Duncan Davis J, Bishop DS, Fortinsky RH. Telephone-delivered psychosocial intervention reduces burden in dementia caregivers. Dementia. 2008 Nov;7(4):503-20.

141. Trento M, Gamba S, Gentile L, Grassi G, Miselli V, Morone G, Passera P, Tonutti L, Tomalino M, Bondonio P, Cavallo F. Rethink Organization to iMprove Education and Outcomes (ROMEO) A multicenter randomized trial of lifestyle intervention by group care to manage type 2 diabetes. Diabetes care. 2010 Apr 1;33(4):745-7.

142. Trofimov EE, Poskrebysheva AS, Gurskaya A, Novikova A. Therapeutic education for patients with heart failure: effect on medication adherence and clinical outcomes.European Journal of Heart Failure. 2015;17: 294.

143. Vaillant-Roussel H, Laporte C, Pereira B, De Rosa M, Eschalier B, Vorilhon C, Eschalier R, Clément G, Pouchain D, Chenot JF, Dubray C. Impact of patient education on chronic heart failure in primary care (ETIC): a cluster randomised trial. BMC Family Practice. 2016 Dec;17(1):1-3.

144. van de Ven G, Draskovic I, Adang EM, Donders R, Zuidema SU, Koopmans RT, Vernooij-Dassen MJ. Effects of dementia-care mapping on residents and staff of care homes: a pragmatic cluster-randomised controlled trial. PLoS ONE. 2013 Jul 2;8(7):e67325.

145. Van der Aa HP, van Rens GH, Comijs HC, Margrain TH, Gallindo-Garre F, Twisk JW, van Nispen RM. Stepped care for depression and anxiety in visually impaired older adults: multicentre randomised controlled trial. BMJ. 2015; 351.

146. Van Wetering CR, Hoogendoorn M, Mol SJ, Rutten-van Mölken MP, Schols AM. Short-and long-term efficacy of a community-based COPD management programme in less advanced COPD: a randomised controlled trial. Thorax. 2010 Jan 1;65(1):7-13.

147. Vickrey BG, Mittman BS, Connor KI, Pearson ML, Della Penna RD, Ganiats TG, DeMonte Jr RW, Chodosh J, Cui X, Vassar S, Duan N. The effect of a disease management intervention on quality and outcomes of dementia care: a randomized, controlled trial. Annals of Internal Medicine. 2006 Nov 21;145(10):713-26.

148. Vinereanu D, Lopes RD, Bahit MC, Xavier D, Jiang J, Al-Khalidi HR, He W, Xian Y, Ciobanu AO, Kamath DY, Fox KA. A multifaceted intervention to improve treatment with oral anticoagulants in atrial fibrillation (IMPACT-AF): an international, cluster-randomised trial. The Lancet. 2017 Oct 14;390(10104):1737-46.

149. Wagenaar KP, Broekhuizen BD, Jaarsma T, Kok I, Mosterd A, Willems FF, Linssen GC, Agema WR, Anneveldt S, Lucas CM, Mannaerts HF. Effectiveness of the European Society of Cardiology/Heart Failure Association website ‘heartfailurematters. org’and an e-health adjusted care pathway in patients with stable heart failure: results of the ‘e-Vita HF’randomized controlled trial. European Journal of Heart Failure. 2019 Feb;21(2):238-46.

150. Waldorff FB, Buss DV, Eckermann A, Rasmussen ML, Keiding N, Rishøj S, Siersma V, Sørensen J, Sørensen LV, Vogel A, Waldemar G. Efficacy of psychosocial intervention in patients with mild Alzheimer’s disease: the multicentre, rater blinded, randomised Danish Alzheimer Intervention Study (DAISY). BMJ. 2012 Jul 17;345.

151. Walters J, Cameron-Tucker H, Wills K, Schüz N, Scott J, Robinson A, Nelson M, Turner P, Wood-Baker R, Walters EH. Effects of telephone health mentoring in community-recruited chronic obstructive pulmonary disease on self-management capacity, quality of life and psychological morbidity: a randomised controlled trial. BMJ Open. 2013 Sep 1;3(9):e003097.

152. Whittle J, Schapira MM, Fletcher KE, Hayes A, Morzinski J, Laud P, Eastwood D, Ertl K, Patterson L, Mosack KE. A randomized trial of peer-delivered self-management support for hypertension. American Journal of Hypertension. 2014 Nov 1;27(11):1416-23.

153. Wilcock J, Iliffe S, Griffin M, Jain P, Thuné-Boyle I, Lefford F, Rapp D. Tailored educational intervention for primary care to improve the management of dementia: the EVIDEM-ED cluster randomized controlled trial. Trials. 2013 Dec;14(1):1-10.

154. Wright LK, Litaker M, Laraia MT, DeAndrade S. Continuum of care for Alzheimer's disease: a nurse education and counseling program. Issues in mental health nursing. 2001 Jan 1;22(3):231-52.

155. Xi F, Wang Z, Qi Y, Brightwell R, Roberts P, Stewart A, Sim M, Wang W. Long-term effect of respiratory training for chronic obstructive pulmonary disease patients at an outpatient clinic: a randomised controlled trial. Clinical and Translational Medicine. 2015 Dec;4(1):1-7.

156. Xiao LD, De Bellis A, Kyriazopoulos H, Draper B, Ullah S. The effect of a personalized dementia care intervention for caregivers from Australian minority groups. American Journal of Alzheimer's Disease & Other Dementias. 2016 Feb;31(1):57-67.

157. Zwar NA, Bunker JM, Reddel HK, Dennis SM, Middleton S, van Schayck OC, Crockett AJ, Hasan I, Hermiz O, Vagholkar S, Xuan W. Early intervention for chronic obstructive pulmonary disease by practice nurse and GP teams: a cluster randomized trial. Family Practice. 2016 Aug 17;33(6):663-7

List of 39 Companion reports:

CR1. Alexopoulos GS, Reynolds III, MD CF, Bruce ML, Katz IR, Raue PJ, Mulsant BH, Oslin DW, Ten Have T, PROSPECT Group. Reducing suicidal ideation and depression in older primary care patients: 24-month outcomes of the PROSPECT study. American Journal of Psychiatry. 2009 Aug;166(8):882-90.

CR2. Bogner HR, Joo JH, Hwang S, Morales KH, Bruce ML, Reynolds III CF, Gallo JJ. Does a depression management program decrease mortality in older adults with specific medical conditions in primary care? An exploratory analysis. Journal of the American Geriatrics Society. 2016 Jan;64(1):126-31.

CR3. Boland MR, Kruis AL, Huygens SA, Tsiachristas A, Assendelft WJ, Gussekloo J, Blom CM, Chavannes NH, Rutten-van Mölken MP. Exploring the variation in implementation of a COPD disease management programme and its impact on health outcomes: a post hoc analysis of the RECODE cluster randomised trial. NPJ primary care respiratory medicine. 2015 Dec 17;25(1):1-9.

CR4. Bourbeau J, Nault D, Dang-Tan T. Self-management and behaviour modification in COPD. Patient education and counseling. 2004 Mar 1;52(3):271-7.

CR5. Boyne J, Vrijhoef HJ, Spreeuwenberg M, Kragten J, Deweerd GJ, Gorgels AP. The effects of telemonitoring on heart failure patients' knowledge, self-care, self-efficacy and adherence: a randomized

controlled trial. In European Journal of Cardiovascular Nursing 2013 Apr 1 (Vol. 12, pp. S49-S49). 1 OLIVERS YARD, 55 CITY ROAD, LONDON EC1Y 1SP, ENGLAND: SAGE PUBLICATIONS LTD.

CR6. Burns R, Nichols LO, Martindale-Adams J, Graney MJ. Interdisciplinary geriatric primary care evaluation and management: Two-year outcomes. Journal of the American Geriatrics Society. 2000 Jan;48(1):8-13.

CR7. Cleveringa FG, Gorter KJ, Van den Donk M, Pijman PL, Rutten GE. Task delegation and computerized decision support reduce coronary heart disease risk factors in type 2 diabetes patients in primary care. Diabetes technology & therapeutics. 2007 Oct 1;9(5):473-81.

CR8. Cleveringa FG, Minkman MH, Gorter KJ, Van Den Donk M, Rutten GE. Diabetes Care Protocol: effects on patient-important outcomes. A cluster randomized, non-inferiority trial in primary care. Diabetic Medicine. 2010 Apr;27(4):442-50.

CR9. Dorsey ER, Achey MA, Beck CA, Beran DB, Biglan KM, Boyd CM, Schmidt PN, Simone R, Willis AW, Galifianakis NB, Katz M. National randomized controlled trial of virtual house calls for people with Parkinson's disease: interest and barriers. Telemedicine and e-Health. 2016 Jul 1;22(7):590-8.

CR10. Drentea P, Clay OJ, Roth DL, Mittelman MS. Predictors of improvement in social support: Five-year effects of a structured intervention for caregivers of spouses with Alzheimer's disease. Social science & medicine. 2006 Aug 1;63(4):957-67.

CR11. Flamm M, Panisch S, Winkler H, Johansson T, Weitgasser R, Sönnichsen AC. Effectiveness of the Austrian disease management programme “Therapie Aktiv” for type 2 diabetes regarding the improvement of metabolic control, risk profile and guideline adherence: 2 years of follow up. Wiener klinische Wochenschrift. 2012 Sep;124(17):639-46.

CR12. Flamm M, Panisch S, Winkler H, Sönnichsen AC. Impact of a randomized control group on perceived effectiveness of a Disease Management Programme for diabetes type 2. The European Journal of Public Health. 2012 Oct 1;22(5):625-9.

CR13. Gadoury, M. A.; Schwartzman, K.; Rouleau, M.; Maltais, F.; Julien, M.; Beaupre, A.; Renzi, P.; Begin, R.; Nault, D.; Bourbeau, J. Self-management reduces both short- and long-term hospitalisation in COPD. European Respiratory Journal. 2005; 26(5):853-857.

CR14. Gallo JJ, Morales KH, Bogner HR, Raue PJ, Zee J, Bruce ML, Reynolds CF. Long term effect of depression care management on mortality in older adults: follow-up of cluster randomized clinical trial in primary care. BMJ. 2013 Jun 5;346.

CR15. Hunger M, Kirchberger I, Holle R, Seidl H, Kuch B, Wende R, Meisinger C. Does nurse-based case management for aged myocardial infarction patients improve risk factors, physical functioning and mental health? The KORINNA trial. European journal of preventive cardiology. 2015 Apr 1;22(4):442-50.

CR16. Iliffe S, Koch T, Jain P, Lefford F, Wong G, Warner A, Wilcock J. Developing an educational intervention on dementia diagnosis and management in primary care for the EVIDEM-ED trial. Trials. 2012 Dec;13(1):1-2.

CR17. Izquierdo R, Meyer S, Starren J, Goland R, Teresi J, Shea S, Weinstock RS. Detection and remediation of medically urgent situations using telemedicine case management for older patients with diabetes mellitus. Therapeutics and clinical risk management. 2007 Jun;3(3):485.

CR18. Jingbo L, Guangwei Z. GW25-e4202 Analysis for readmission of the heart failure patients. Journal of the American College of Cardiology. 2014 Oct 21;64(16S):C105-.

CR19. Joling KJ, Van Marwijk HW, Van der Horst HE, Scheltens P, Van de Ven PM, Appels BA, Van Hout HP. Effectiveness of family meetings for family caregivers on delaying time to nursing home placement of dementia patients: a randomized trial.

CR20. Kennedy CC, Thabane L, Ioannidis G, Adachi JD, Papaioannou A, ViDOS Investigators. Implementing a knowledge translation intervention in long-term care: feasibility results from the Vitamin D and Osteoporosis Study (ViDOS). Journal of the American Medical Directors Association. 2014 Dec 1;15(12):943-5.

CR21. Kirchberger I, Hunger M, Stollenwerk B, Seidl H, Burkhardt K, Kuch B, Meisinger C, Holle R. Effects of a 3-year nurse-based case management in aged patients with acute myocardial infarction on rehospitalisation, mortality, risk factors, physical functioning and mental health. A secondary analysis of the randomized controlled KORINNA study. PloS one. 2015 Mar 26;10(3):e0116693.

CR22. Lowrie R, Morrison J, McConnachie A. A cluster randomised controlled trial of pharmacist led Statin Outreach Support (SOS) in primary care: Design and baseline characteristics. Contemporary Clinical Trials. 2010 Jul 1;31(4):303-11.

CR23. Mittelman MS, Haley WE, Clay OJ, Roth DL. Improving caregiver well-being delays nursing home placement of patients with Alzheimer disease. Neurology. 2006 Nov 14;67(9):1592-9.

CR24. Mittelman MS, Roth DL, Coon DW, Haley WE. Sustained benefit of supportive intervention for depressive symptoms in caregivers of patients with Alzheimer’s disease. American Journal of Psychiatry. 2004 May 1;161(5):850-6.

CR25. Moy ML, Collins RJ, Martinez CH, Kadri R, Roman P, Holleman RG, Kim HM, Nguyen HQ, Cohen MD, Goodrich DE, Giardino ND. An internet-mediated pedometer-based program improves health-related quality-of-life domains and daily step counts in COPD. Chest. 2015 Jul 1;148(1):128-37.

CR26. Moy ML, Martinez CH, Kadri R, Roman P, Holleman RG, Kim HM, Nguyen HQ, Cohen MD, Goodrich DE, Giardino ND, Richardson CR. Long-term effects of an internet-mediated pedometer-based walking program in COPD: a randomized controlled trial. InB16. NEW RANDOMISED CONTROLLED TRIALS IN PULMONARY REHABILITATION 2015 May (pp. A2457-A2457). American Thoracic Society.

CR27. Murphy AW, Cupples ME, Murphy E, Newell J, Scarrott CJ, Vellinga A, Gillespie P, Byrne M, Kearney C, Smith SM. Six-year follow-up of the SPHERE RCT: secondary prevention of heart disease in general practice. BMJ open. 2015 Nov 1;5(11):e007807.

CR28. Nesbitt T, Doctorvaladan S, Southard JA, Singh S, Fekete A, Marie K, Moser DK, Pelter MM, Robinson S, Wilson MD, Cooper L. Correlates of quality of life in rural patients with heart failure. Circulation: Heart Failure. 2014 Nov;7(6):882-7.

CR29. Nielsen AB, de Fine Olivarius N, Gannik D, Hindsberger C, Hollnagel H. Structured personal diabetes care in primary health care affects only women’s HbA1c. Diabetes care. 2006 May 1;29(5):963-9.

CR30. Olaiya MT, Cadilhac DA, Kim J, Nelson MR, Srikanth VK, Gerraty RP, Bladin CF, Fitzgerald SM, Phan T, Frayne J, Thrift AG. Community-based intervention to improve cardiometabolic targets in patients with stroke: a randomized controlled trial. Stroke. 2017 Sep;48(9):2504-10.

CR31. Peters A, Döring A, Ladwig KH, Meisinger C, Linkohr B, Autenrieth C, Baumeister SE, Behr J, Bergner A, Bickel H, Bidlingmaier M. Multimorbidity and successful aging: the population-based KORA-Age study. Zeitschrift fur Gerontologie und Geriatrie. 2011 Dec 1;44:41-54.

CR32. Phung KT, Waldorff FB, Buss DV, Eckermann A, Keiding N, Rishøj S, Siersma V, Sørensen J, Søgaard R, Sørensen LV, Vogel A. A three-year follow-up on the efficacy of psychosocial interventions for patients with mild dementia and their caregivers: the multicentre, rater-blinded, randomised Danish Alzheimer Intervention Study (DAISY). BMJ open. 2013 Nov 1;3(11):e003584.

CR33. Piette JD, Striplin D, Marinec N, Chen J, Aikens JE. A randomized trial of mobile health support for heart failure patients and their informal caregivers: impacts on caregiver-reported outcomes. Medical care. 2015 Aug;53(8):692.

CR34. Shea S, Weinstock RS, Teresi JA, Palmas W, Starren J, Cimino JJ, Lai AM, Field L, Morin PC, Goland R, Izquierdo RE. A randomized trial comparing telemedicine case management with usual care in older, ethnically diverse, medically underserved patients with diabetes mellitus: 5 year results of the IDEATel study. Journal of the American Medical Informatics Association. 2009 Jul 1;16(4):446-56.

CR35. Trief PM, Izquierdo R, Eimicke JP, Teresi JA, Goland R, Palmas W, Shea S, Weinstock RS. Adherence to diabetes self care for white, African-American and Hispanic American telemedicine participants: 5 year results from the IDEATel project. Ethnicity & Health. 2013 Feb 1;18(1):83-96.

CR36. Vallespin B, Fonollosa D, Alonso A, Roca, J. The deployment of integrated care services for chronic patients: The NEXES project. Journal on Information Technology in Healthcare. 2008; 6(5):334-343.

CR37. van de Ven G, Draskovic I, Adang EM, Donders RA, Post A, Zuidema SU, Koopmans RT, Vernooij-Dassen MJ. Improving person-centred care in nursing homes through dementia-care mapping: design of a cluster-randomised controlled trial. BMC geriatrics. 2012 Dec;12(1):1-9.

CR38. Whitty JA, Stewart S, Carrington MJ, Calderone A, Marwick T, Horowitz JD, Krum H, Davidson PM, Macdonald PS, Reid C, Scuffham PA. Patient preferences and willingness-to-pay for a home or clinic based program of chronic heart failure management: findings from the Which? trial. PLoS One. 2013 Mar 7;8(3):e58347.

CR39. Whitty P, Eccles MP, Hawthorne G, Steen N, Vanoli A, Grimshaw JM, Wood L, Speed C, McDowell D. Improving services for people with diabetes: lessons from setting up the DREAM trial. Practical Diabetes International. 2004 Nov;21(9):323-8.

Appendix 9. Individual Study Characteristics

**Author, year (*companion report ref. number*)**	**Study duration**	**KT Intervention name**	**Duration of KT Intervention**	**Intervention (EPOC) components**	**Intervention (BCT) components**	**Outcomes included in analysis***	**Setting**	**Funding**
Ansari, 2003[43]	Oct 1998 to Apr 2001 (30 months)	Clinician education+patient registry; Clinician education+nurse facilitator; Clinician education		staff+clin_reminder+px_reminder; team+staff+case-M; staff	NT_HP_1.1+NT_HP_2.3 +NT_HP_4.1+NT_HP_7.1 +NT_HP_9.1+NT_HP_12.1+NT_HP_12.2+NT_HP_12.5+NT_P_7.1; NT_HP_3.2+NT_HP_4.1+NT_HP_9.1+NT_HP_12.2; NT_HP_4.1+NT_HP_9.1+NT_HP_13.2	Quality of care; Assessed adherence	NR	Governmental/Public funding
Baker, 2003[44]	NR	Review + feedback; Review; Guideline alone	12 months	AF+staff; staff; staff	NT_HP_1.3+T_HP_2.2+NT_HP_6.2; NT_HP_1.3+NT_HP_9.1; NT_HP_1.3+NT_HP_9.1	Quality of care; Assessed adherence	NA	Governmental/Public funding
Batchelor, 2012[45]	NR	Exercise program; Control		Self-M+px_educ; px_educ; px_educ	NT_P_1.1+T_P_1.4+NT_P_2.3 +NT_P_2.7+NT_P_12.1+T_P_12.6; NT_P_2.3	Assessed adherence; Assessed sustainability	home	Governmental/Public funding
Beck, 1997[46]	NR	Group visits; Control	12 months	team+case-M+px_educ;	NT_P_4.1+NT_P_12.2	Quality of care	clinic	Mixed
Beck, 2017[47] (*CR9*)	NR	Virtual home visit; Usual care	12 months	team+case-M+px_finan	NT_P_4.1+NT_P_12.2	EQ5D	NA	Research funding body
Bekelman, 2015[48]	NR	Patient-Centered Disease Management (PCDM) intervention; Usual care		team+relay+staff+case-M+Self-M+px_reminder+px_educ; px_educ	NT_HP_1.2+NT_HP_4.1+NT_P_7.1+T_P_11.1; NT_P_4.1+NT_P_7.1+NT_P_12.1+NT_P_13.1		home, clinic	Governmental/Public funding
Benzo, 2016[49]	Sep 2010-Aug 2014 (47 months)	Health coaching; Usual care	12 months	relay+staff+case-M+Self-M+mot_interview; staff	NT_P_1.1+NT_P_1.4+NT_P_4.1+NT_P_6.1 +NT_P_12.2+NT_P_12.5+NT_P_12.6	Assessed adherence; Assessed fidelity	home, clinic	Governmental/Public funding
Blue, 2001[50]	NR	Home visits; Control	12 months	case-M+Self-M+px_educ	NT_P_2.4+T_P_3.2+NT_P_4.1 +T_P_5.1	Assessed sustainability	home	Governmental/Public funding
Bohingamu, 2019[50, 51]	NR	Patient education; Usual care	12 months	relay+case-M+Self-M+px_educ	T_P_1.3 +NT_P_2.7+NT_P_4.1+NT_P_12.1+NT_P_12.2		home	Mixed
Bohm, 2016[50, 52]	NR	Telemedicine alerts; Control		team+relay+case-M	NT_HP_2.5+NT_HP_7.1	Assessed sustainability	home	Commercial
Community Pharmacy Medicines	Nov 2002 to May 2004 (18 months)	Pharmacist management; Usual care		team+px_educ	T_HP_12.2	EQ5D	community	Governmental/Public funding

Author, year (*companion report ref. number*)	Study duration	KT Intervention name	Duration of KT Intervention	Intervention (EPOC) components	Intervention (BCT) components	Outcomes included in analysis*	Setting	Funding
Management Project Evaluation Team, 2007 [53]								
Bosanquet, 2017[54]	NR	Collaborative care; Usual care	18 months	team+EPR+relay+case-M+Self-M	NT_HP_2.4 +NT_HP_2.7+NT_HP_4.1+NT_HP_12.5+NT_P_1.2+NT_P_1.3+NT_P_4.1+NT_P_12.2+NT_P_12.5	SF-12 mental; SF-12 physical; Assessed adherence; Assessed fidelity	NR	Governmental/Public funding
Bourbeau, 2003[55] (*CR4, 13*)	Feb 1998 to Jul 1999 (17 months)	Provider education; Usual care	7-8 week long programme + telephone calls, monthly calls for rest of 12 months	case-M+Self-M+px_educ	NT_P_1.4+T_P_2.3	SGRQ; Assessed sustainability	clinic, home	Mixed
Boyne, 2012[56] (*CR5*)	NR	Telemonitoring; Control	12 months	relay+px_educ; staff+px_educ	NT_P_2.7+NT_P_4.1+NT_P_12.5; NT_P_4.1	Assessed adherence	home, clinic	Mixed
Bruce, 2004[57] (*CR1, 2, 14*)	NR	Prospect; Control	12-24 months	hs finan+team+relay+staff+case-M; relay+staff	NT_HP_3.2+NT_HP_4.1+NT_HP_7.1 +NT_HP_9.1+NT_P_12.2; NT_HP_4.1+NT_HP_7.1		NR	Mixed
Bruce, 2015[58]	NR	Protocol development; Control	12 months	team+staff+case-M+Self-M+px_educ	NT_HP_3.2+NT_HP_4.1+NT_HP_12.1+NT_P_1.3+NT_P_4.1 + NT_P_12.2; NT_HP_4.1+NT_HP_12.1	Assessed adherence	home	Governmental/Public funding
Bucknall, 2012[59]	Jun 2007 to May 2010 (35 months)	Patient education; Usual care	training sessions for 2 months; home visits for a total of 12 months	case-M+Self-M	NT_P_1.1+NT_P_2.4+NT_P_2.7+NT_P_3.1+T_P_4.1	EQ5D (a); SGRQ; Assessed sustainability	home	Mixed
Burns, 1995[60] (*CR6*)	NR	Comprehensive assessment; Control	36 months	team+case-M	NT_HP_1.1+NT_HP_1.3+NT_P_1.1+NT_P_1.3+NT_P_12.2		clinic	Voluntary body
Burns, 2003[61]	NR	Enhanced care; Behavior care	24 months	Self-M+px_educ+mot_interview; Self-M+px_educ+mot_interview	NT_C_1.2+NT_C_1.3 +NT_C_1.4+NT_C_4.1; NT_C_1.2+NT_C_1.3 +NT_C_4.1+NT_C_13.1		NA	Governmental/Public funding
Callahan, 2006[62]	NR	Collaborative care; Control	12 months	team+EPR+case-M+Self-M+px_educ	NT_HP_2.1+NT_HP_12.2+T_C_2.2+NT_C_3.1+NT_C_13.3; NT_C_3.1		NR	Governmental/Public funding
Campbell, 1998[63]	NR	Nurse-led clinic; Control	12 months	staff+case-M+Self-M+px_educ	NT_HP_4.1+NT_HP_12.2+NT_P_1.1+NT_P_1.4+NT_P_4.1+ NT_P_12.2+NT_P_12.6		clinic	Governmental/Public funding

**Author, year (*companion report ref. number*)**	**Study duration**	**KT Intervention name**	**Duration of KT Intervention**	**Intervention (EPOC) components**	**Intervention (BCT) components**	**Outcomes included in analysis***	**Setting**	**Funding**
Chen, 2015[36]	Jan 2011 to Nov 2013 (34 months)	Collaborative-care depression care management (DCM) intervention; Usual care	12 months	team+staff+case-M+px_reminder+px_educ; staff	NT_HP_1.2+NT_HP_4.1+NT_HP_9.1+NT_HP_12.2+NT_HP_13.2+NT_P_2.5+NT_P_4.1+NT_P_7.1+NT_P_12.2; NT_HP_9.1	Assessed adherence	clinic	Governmental/Public funding
Chi, 2012[64]	NR	Self-care education; Usual care	NR	Self-M	NT_P_2.3	MLHFQ	home	NR
Ciaschini, 2010[65]	NR	Guideline-based care; Usual care	NR	staff+Self-M+px_educ	NT_HP_4.1+NT_P_1.3+NT_P_4.1		home, clinic	Mixed
Cleveringa, 2008[66] (*CR7,8*)	NR	Physician support; Control	12 months	AF+staff	NT_HP_4.1+NT_HP_6.2+NT_HP_12.2		NR	Commercial
Coleman, 1999[67]	NR	Care team; Usual care	24 months	team+staff+case-M+Self-M	NT_HP_1.1+NT_HP_1.2+NT_HP_4.1+NT_HP_12.2+NT_P_1.2+NT_P_1.3+NT_P_4.1+NT_P_12.2		NA	Research funding body
Coull, 2004[68]	NR	Health mentors; Usual care	12 months	Self-M+px_educ+px_finan	NT_P_2.5+T_P_5.1+NT_P_9.1+NT_P_13.1		NR	Mixed
de la Porte, 2007[69]	NR	HF clinic follow-up; Usual care	12 months	team+case-M+Self-M+px_educ	NT_P_1.1+NT_P_2.5+NT_P_4.1+NT_P_12.2	MLHFQ; Defined Sustainability; Assessed adherence, Assessed sustainability	clinic	Commercial
de Lusignana, 2013[70]	NR	Audit-based education; Guidelines; Control	24 months	EPR+relay+AF+staff; staff+clin_reminder	T_HP_2.7+NT_HP_3.2+NT_HP_6.2; NT_HP_4.1+NT_HP_7.1	Assessed adherence; Assessed fidelity	NA	Mixed
DeBusk, 2004[71]	NR	Nurse care management; Control	12 months	relay+case-M+Self-M+px_educ; px_educ	NT_P_1.3+NT_P_2.4+NT_P_2.7+NT_P_4.1+NT_P_12.2		home, clinic	Governmental/Public funding
Del Sindaco, 2007[72]	NR	Team management; Usual care	24 months	team+case-M+px_reminder+px_educ	NT_HP_3.2+NT_P_2.5+NT_P_3.2 +NT_P_13.1; NT_P_13.1		hospital, clinic	NR
DeVore, 2015[73]	Oct 2009 to Mar 2011 (17 months)	Personalized reporting; Control	NR	EPR+relay+cQI+AF+staff; EPR+relay+cQI+AF	NT_HS_1.1+NT_HS_1.3+NT_HS_2.1+NT_HS_2.2+NT_HS_2.7+NT_HS_4.1+NT_HS_12.5; NT_HS_1.1+NT_HS_2.1	Assessed adherence	hospital	Governmental/Public funding
Dopp, 2015[74]	Jan 2009 to Dec 2011 (35 months)	Complex training; Usual care	12 months	EPR+staff+mot_interview; staff	NT_HP_1.2+NT_HP_4.1+NT_HP_6.1+NT_HP_8.1+NT_HP_12.5; NT_HP_4.1+NT_HP_6.1	Assessed adherence	NA	Mixed
Dracup, 2014[42] (*CR28*)	Sep 2006 to Jan 2012 (64 months)	Fluid Watchers PLUS; Fluid	24 months	relay+case-M+Self-M+px_reminder+px_educ+px_finan; relay+Self-	NT_P_1.3+NT_P_2.4+NT_P_4.1+NT_P_10.1 +NT_P_12.2+NT_P_12.5;	Assessed sustainability	home, clinic	Governmental/Public funding

**Author, year (*companion report ref. number*)**	**Study duration**	**KT Intervention name**	**Duration of KT Intervention**	**Intervention (EPOC) components**	**Intervention (BCT) components**	**Outcomes included in analysis***	**Setting**	**Funding**
		Watchers LITE; Control		M+px_reminder+px_educ+ px_finan; px_educ	NT_P_1.3+NT_P_2.4+NT_P_4 .1+NT_P_9.1+NT_P_10.1 +NT_P_12.2+NT_P_12.5; NT_P_4.1+NT_P_9.1			
Dunagan, 2005[75]	NR	Telephone support; Educational materials	12 months	relay+case-M+Self-M+px_educ; relay+px_educ	NT_P_4.1+NT_P_5.1+NT_P_1 2.2; NT_P_4.1+NT_P_5.1+NT_P_1 3.1	SF-12 mental; SF-12 physical MLHFQ; (b)	NR	Mixed
Eccles, 2007[34] (*CR38*)	Apr 2002 to June 2003 (14 months)	Patient register; Usual care	15 months	EPR+staff+clin_reminder+ px_reminder	NT_HP_4.1+NT_HP_7.1 +NT_HP_12.1	SF-36 mental; SF-36 physical; Quality of care; Assessed adherence	clinic	Government al/Public funding
Eckert, 2010[76]	NR	Comprehensive care; Control	NR	staff	NT_HP_4.1+T_P_3.2		NR	NR
Ell, 2007[77]	NR	Medication-enhanced usual care; Enhanced usual care	12 months	relay+cQI+staff; staff	NT_HP_12.2+NT_HP_13.2; NT_HP_13.2	Assessed adherence	home	Government al/Public funding
Fan, 2012[78]	Jan 2007 to 27 Feb 2009 (26 months)	Patient education; Usual care	1 month education sessions; 12 months phone calls	team+staff+case-M+Self-M+px_educ; staff+px_educ	T_P_3.1+T_P_3.2+T_P_4.1 +NT_P_12.2; NT_P_13.1	SGRQ; Assessed adherence; Assessed fidelity	home, clinic	Government al/Public funding
Federman, 2019[79]	NR	Patient education; Usual care	12 months	Self-M+px_reminder+px_educ	NT_P_1.2+NT_P_1.3+NT_P_1 .7+NT_P_4.1+NT_P_12.2		home, clinic	Voluntary body
Fihn, 2004[80]	NR	Clinician reporting; Usual care	18-24 months	AF+staff	NT_HP_4.1+NT_HP_6.2+NT_ HP_7.1	SF-36 mental; SF-36 physical	NA	Government al/Public funding
Fihn, 2011[81]	Mar 1997 to Dec 1999 (33 months)	Team care; Usual care	12 months	team+relay+case-M+px_educ	T_HP_1.1+NT_HP_12.2+NT_ P_13.1	Assessed adherence	clinic	Government al/Public funding
Forster, 1996[82]	NR	Specialist nurse support; Control	12 months	staff+case-M+Self-M+px_educ	NT_HP_8.1+NT_P_1.2+NT_P _1.3+NT_P_1.4+NT_P_4.1+N T_P_12.2+T_P_12.5	Defined Sustainability; Assessed adherence	home	Charitable trust
Forster, 2015[83]	Jul 2009 to May 2012 (34 months)	Care plan; Usual care	12 months	team+case-M+Self-M+px_educ	NT_P_1.2+NT_P_1.3+NT_P_1 .4+NT_P_4.1+NT_P_7.1+NT_ P_12.2	EQ5D	NA	Government al/Public funding
Fortinsky, 2009[84]	NR	Dementia care consultation; Control	12 months	relay+case-M+Self-M+px_educ; staff	NT_C_1.2+NT_C_1.3 +NT_C_1.4+NT_C_4.1+NT_C _12.2	Assessed sustainability	NA	Voluntary body
Freund, 2016[85]	Jul 2010 to Jun 2013 (35 months)	Health coach; Usual care	mean duration 21 months	team+staff+finan+case-M+Self-M+px_educ	T_HP_1.1+NT_HP_2.4 +NT_HP_13.2+NT_P_1.1+NT _P_12.2; NT_P_13.1	SF-12 mental; SF-12 physical; EQ5D	clinic	Commercial
Galbreath, 2004 (c)[86]	1999 to 2003 (48 months)	Disease management; Augmented	18 months	team+staff+case-M+Self-M+px_educ;	NT_P_4.1+NT_P_12.2+NT_P_ 12.5;	SF-36 mental; SF-36 physical; Quality of care; Assessed adherence	NR	Government al/Public funding

Author, year (*companion report ref. number*)	Study duration	KT Intervention name	Duration of KT Intervention	Intervention (EPOC) components	Intervention (BCT) components	Outcomes included in analysis*	Setting	Funding
		disease management; Usual care		team+relay+staff+case-M+Self-M+px_educ	NT_P_2.4+NT_P_2.7+NT_P_4.1+NT_P_12.2+NT_P_12.5			
Gallagher, 1997[87]	NR	Social support-only group; Social support + education group; Education-only group; Control	36 months	px_educ; px_educ; px_educ	NT_P_6.1 +NT_P_12.2+NT_P_13.1; T_P_4.1 +NT_P_6.1 +NT_P_12.2; T_P_4.1		NA	Governmental/Public funding
Gaugler, 2008[88] (*CR10, 23, 24*)	NR	Counseling; Control	6 session over 4 months (counselling), weekly support group (8 months)	Self-M+px_educ	NT_C_1.2+NT_C_4.1+NT_C_12.2	Defined Sustainability	NA	Governmental/Public funding
Gellis, 2012[89]	NR	Telehealth; Usual care	12 months	team+relay+Self-M+px_educ; case-M+px_educ	NT_P_1.2+NT_P_1.3+NT_P_2.4+NT_P_2.7+NT_P_4.1+NT_P_12.2+NT_P_12.5; NT_P_4.1+NT_P_12.2+NT_P_13.1		home	Governmental/Public funding
Goderis, 2010[27]	NR	Usual Quality Improvement Program (UQIP); Advanced Quality Improvement Program (AQIP)	18 months	AF+staff+finan+px_educ; hs finan+team+AF+staff+clin_reminder+px_educ+mot_interview	NT_HP_1.4+NT_HP_3.2+NT_HP_4.1+NT_HP_6.2+NT_P_4.1; NT_HP_4.1+NT_HP_6.2+NT_HP_7.1 +NT_HP_12.2+NT_P_4.1+NT_P_12.5		home, clinic	Governmental/Public funding
Graven, 2016[90]	NR	Telephone support; Control	12 months	case-M+Self-M+px_educ	NT_P_1.2+NT_P_1.3+NT_P_1.4+NT_P_3.2 +NT_P_4.1+NT_P_12.2		NA	Mixed
Haerter, 2016[91]	NR	Telephone-based coaching; Control	12 to up to 24 months	staff+case-M+Self-M+px_educ+mot_interview	NT_HP_4.1+T_P_1.1+NT_P_1.3+NT_P_4.1+NT_P_12.2	Defined Sustainability; Assessed adherence; Assessed fidelity	home	Commercial
Heisler, 2012[28]	Aug 2008 to March 2010 (20 months)	Provider training; Control	14 months	team+relay+staff+case-M+Self-M+px_educ+mot_interview; relay+case-M	NT_HP_3.2+NT_HP_4.1+NT_P_1.3+T_P_1.7+NT_P_2.4+NT_P_2.7+NT_P_4.2+NT_P_12.1 +NT_P_12.5	Assessed adherence	clinic	Governmental/Public funding
Hendriks, 2012[92]	NR	Nurse-led care; Control	NR	case-M+Self-M+px_educ	NT_HP_2.5+NT_HP_12.5+NT_P_4.1+NT_P_12.2+NT_P_12.5	Assessed adherence	clinic	Mixed
Hernandez, 2015[93] (*CR36*)	NR	Patient education; Usual care	12 months	team+relay+staff+case-M+Self-M+px_educ	NT_HP_12.2+T_P_1.1+NT_P_3.2 +NT_P_4.1	SGRQ	home, clinic	Unclear
Hetlevik, 2000[94]	NR	Decision support; Control	18 months	EPR+staff+clin_reminder+px_educ	NT_HP_4.1+NT_HP_7.1 +NT_HP_12.5	Quality of care; Assessed adherence	NR	Mixed

**Author, year (*companion report ref. number*)**	**Study duration**	**KT Intervention name**	**Duration of KT Intervention**	**Intervention (EPOC) components**	**Intervention (BCT) components**	**Outcomes included in analysis***	**Setting**	**Funding**
Hogg, 2009[95]	Oct 2004 to Mar 2006 (17 months)	Team care; Usual care	12 to 18 months (mean of 14.9 months)	team+case-M	T_P_1.1+NT_P_3.2 +NT_P_12.2	SF-36 mental; SF-36 physical; Assessed adherence	home, clinic	Government al/Public funding
Holbrook, 2011[96]	NR	Compete III; Control	12 months	relay+px_reminder+px_edu c	NT_HP_7.1 +NT_HP_12.1+NT_HP_12.5+ NT_P_2.4+NT_P_4.1+NT_P_7 .1+NT_P_12.5		home, clinic	Government al/Public funding
Holm, 2002[97]	Apr 1997 to Mar 2000 (35 months)	Shared care program; Control	24 months	AF+staff+Self-M+px_educ	NT_HP_4.1+NT_HP_6.2+NT_ HP_12.2+NT_P_4.1	Assessed adherence	clinic	Government al/Public funding
Holt, 2010[98]	Jun 2006 to Sep 2008 (27 months)	Patient registry; Control	24 months	EPR+clin_reminder	T_HP_7.1	Assessed fidelity	NA	Government al/Public funding
Hughes, 2000[99]	Oct 1994 to Sep 1998 (47 months)	Home based care; Control	12 months	team+case-M	T_HP_12.2	Assessed fidelity	home	Government al/Public funding
Hunger, 2015[100]	NR	Nurse-led care; Control	12 months	case-M+px_educ	NT_P_1.2+NT_P_4.1+NT_P_1 2.2		home	Government al/Public funding
Irewall, 2015[101]	NR	Counselling and assessment; Control	NR	relay+case-M+px_educ	NT_P_4.1+NT_P_12.2	Defined Sustainability	home	Government al/Public funding
Jaarsma, 2008[102] (*CR18*)	NR	Intensive support; Basic support; Control	18 months	team+case-M+Self-M+px_educ; team+case-M+Self-M+px_educ	NT_HP_4.1+NT_P_2.3+T_P_1 2.2+NT_P_13.1; NT_HP_4.1+T_P_12.2+NT_P_ 13.1		home, clinic	Mixed
Joling, 2012[103] (*CR19*)	NR	Counseling; Usual care	12 months	Self-M+px_educ	NT_C_1.2+NT_C_4.1+NT_C_ 11.2+NT_C_12.2	Assessed adherence; Assessed fidelity	NA	Government al/Public funding
Karhula, 2015a (d)[104]	Feb 2011 to Dec 2012 (22 months)	Health coach (heart disease); Usual care	12 months	team+EPR+relay+case-M+Self-M+px_educ+mot_interview ; px_educ	T_P_1.1+NT_P_2.4+T_P_3.2+ NT_P_12.2+NT_P_12.5+NT_P _13.1; NT_P_13.1	SF-36 mental; SF-36 physical; Defined Sustainability; Assessed adherence; Assessed fidelity	home	Government al/Public funding
Karhula, 2015b (d)[104]	Feb 2011 to Dec 2012 (22 months	Health coach (diabetes); Usual care	12 months	team+EPR+relay+case-M+Self-KTM+px_educ+mot_interv iew; px_educ	T_P_1.1+NT_P_2.4+T_P_3.2+ NT_P_12.2+NT_P_12.5+NT_P _13.1; NT_P_13.1	SF-36 mental; SF-36 physical; Defined Sustainability; Assessed adherence; Assessed fidelity	home	Government al/Public funding
Kennedy, 2013[37]	2009 to 2012 (36 months)	Staff training; Usual care	12 months	staff+Self-M	T_HP_3.1+NT_HP_4.1	EQ5D; Assessed adherence; Assessed fidelity	NA	Research funding body
Kennedy, 2015[105] (*CR20*)	2009 to 2012 (36 months)	Quality improvement; Control	12 months	cQI+AF+staff; staff	NT_HP_1.2+NT_HP_1.3+NT_ HP_1.4+NT_HP_4.1+NT_HP_ 6.2+NT_HP_9.1	Assessed fidelity	NA	Government al/Public funding

**Author, year (*companion report ref. number*)**	**Study duration**	**KT Intervention name**	**Duration of KT Intervention**	**Intervention (EPOC) components**	**Intervention (BCT) components**	**Outcomes included in analysis***	**Setting**	**Funding**
Khdour, 2009[106]	Oct 2006 to May 2008 (19 months)	Patient education; Usual care	12 months	team+case-M+Self-M+px_educ+mot_interview	NT_P_1.3+NT_P_1.4+NT_P_4.1+NT_P_6.1 +NT_P_7.1+NT_P_12.2	SGRQ; Assessed adherence	clinic	Voluntary body
Kiefe, 2001[31]	Jan 1994 to Jun 1998 (53 months)	Physician benchmarks; Control	12 months	cQI+AF; cQI+AF	NT_HP_1.2+NT_HP_1.3+T_HP_2.2+NT_HP_6.2; NT_HP_1.2+NT_HP_1.3+T_HP_2.2+NT_HP_6.2	Quality of care	NA	Governmental/Public funding
Kim, 2014[107]	2007 to 2011 (48 months)	Patient education; Control	education for 6 weeks; counseling for 12 months	relay+case-M+Self-M+px_educ; px_educ	NT_P_1.2+NT_P_1.3+NT_P_1.4+NT_P_2.4+NT_P_2.7+NT_P_4.1+NT_P_12.2+NT_P_12.5; NT_P_4.1	Defined Sustainability; Assessed adherence	home, clinic	Governmental/Public funding
Ko, 2017[108]	NR	Patient education; Usual care	12 months	case-M+Self-M+px_educ	NT_P_1.3+NT_P_4.1+NT_P_11.2+NT_P_12.2+NT_P_12.6	SGRQ; Defined Sustainability	home, clinic	NR
Kruis, 2014[109] (*CR3*)	NR	Clinician training; Usual care	12 months	AF+staff+Self-M+mot_interview; staff	NT_HP_1.3+NT_HP_4.1+NT_HP_6.2; NT_HP_4.1	SF-36 mental; SF-36 physical; EQ5D; SGRQ	NA	Mixed
Krum, 2013[110]	NR	Telemedicine support; Control	12 months	team+relay+staff+clin_reminder+case-M+Self-M+px_educ; staff	NT_HP_4.1+NT_HP_7.1 +NT_HP_9.1+NT_P_1.3+NT_P_1.4+NT_P_2.4+NT_P_4.1+NT_P_12.2+NT_P_12.5	Assessed adherence	NR	Governmental/Public funding
Kurz, 2010[111]	NR	Group education; Control	15 months	staff+Self-M+px_educ; px_educ	NT_C_1.2+NT_C_4.1+NT_C_12.2		NA	Governmental/Public funding
Kalter-Leibovici , 2017[112]	Aug 2007 to Jul 2012 (58 months)	Nurse management; Usual care	NR	team+relay+case-M+Self-M+px_educ	T_HP_7.1+NT_P_2.4+T_P_3.2 +NT_P_4.1	SF-36 mental; SF-36 physical; Assessed adherence; Assessed sustainability	home, clinic	Mixed
Leveille, 1998[113]	NR	Health management plan; Usual care	visits and phone calls for 12 months; education course for 7 weeks	team+relay+case-M+Self-M+px_educ	NT_P_1.3+NT_P_1.4+NT_P_4.1+NT_P_12.2+NT_P_12.6		home, community	Mixed
Leventhal, 2011[114]	NR	Patient education; Control	12 months	case-M+Self-M+px_educ; px_educ	NT_P_1.2+NT_P_1.3+NT_P_1.4+NT_P_2.4+NT_P_4.1+NT_P_12.2	Assessed adherence	home, clinic	Governmental/Public funding
Levine, 2011[115]	Jan 2002 to Dec 2008 (83 months)	Clinician education; Control	27 months	staff+clin_reminder; staff	NT_HP_4.1+NT_HP_7.1; NT_HP_9.1	Quality of care; Assessed adherence	NR	Governmental/Public funding
Li, 2012[116]	NR	Self-management education; Control	18 months	Self-M+px_educ; px_educ	NT_P_3.1+NT_P_4.1+NT_P_6.1; NT_P_4.1	Assessed adherence	community	NR

**Author, year (*companion report ref. number*)**	**Study duration**	**KT Intervention name**	**Duration of KT Intervention**	**Intervention (EPOC) components**	**Intervention (BCT) components**	**Outcomes included in analysis***	**Setting**	**Funding**
Licskai, 2016[117]		Case management; Usual care	NR	case-M+px_educ	NT_P_13.1+NT_P_14.1		NR	NR
Lin, 2018 (e)[118]	Nov 2007 to Mar 2011 (40 months)	Pharmacist counselling; Usual care	16 months	team+case-M+px_educ	NT_HP_12.2+NT_P_4.1+NT_P_12.2	EQ5D	NR	Governmental/Public funding
Liu, 2008[119]	NR	Cell phone support; Usual care	NR	relay+Self-M+px_reminder+px_educ; px_educ	NT_P_1.1+NT_P_2.3+NT_P_4.1+NT_P_7.1+NT_P_12.5+NT_P_12.6; NT_P_1.1+NT_P_2.3+NT_P_4.1+NT_P_7.1+NT_P_12.6	SF-12 physical; Defined Sustainability; Assessed adherence	home, clinic	NR
Lopez Cabezas, 2006[120]	NR	Active information program; Usual care	NR	case-M+px_educ	NT_C_1.2+NT_C_4.1+NT_C_12.2+NT_P_1.2+NT_P_4.1+NT_P_12.2	EQ5D; Assessed adherence	home, hospital	Governmental/Public funding
Lowrie, 2014[121] (*CR22*)	Jan 2003 to Jan 2013 (120 months)	Provider training; Control	12 months	team+staff; staff	NT_HP_1.2+NT_HP_1.3+NT_HP_2.7+NT_HP_4.1+NT_HP_9.1+NT_HP_12.2	Quality of care	clinic	Governmental/Public funding
Machline-Carrion, 2019[122]	NR	Clinician Education + Case management; Control	12 months	AF+staff+case-M+px_educ	NT_HP_2.2+NT_HP_4.1+NT_HP_6.1+NT_HP_6.2+NT_HP_7.1 +T_P_3.2+NT_P_4.1+NT_P_12.2	Assessed adherence; Assessed fidelity	NR	Commercial
Mahoney, 2003[123]	NR	Telephone support; Control	12 months	Self-M+px_reminder+px_educ	NT_C_1.2+NT_C_4.1+NT_C_7.1+NT_C_12.2+NT_C_12.4+NT_C_12.5		NA	Governmental/Public funding
Maltais, 2008[124]	NR	Hospital-based intervention; Home-based intervention	12 months	Self-M+px_educ; Self-M+px_educ	NT_P_1.3+NT_P_2.1+NT_P_4.1; NT_P_1.3+NT_P_2.3+NT_P_4.1+NT_P_7.1	SGRQ (f); Assessed adherence	home, clinic	Research funding body
Markle-Reid, 2011[125]	Oct 2005 Sep 2008 (35 months)	Interprofessional rehabilitation; Usual care	12 months	team+staff+case-M+Self-M+px_educ+px_finan; case-M+px_educ+px_finan	NT_P_1.1+NT_P_1.4+NT_P_4.1+NT_P_12.2+T_P_12.5; NT_P_4.1+NT_P_12.2+T_P_12.5	SF-36 mental; SF-36 physical	home	Mixed
Markun, 2015[126]	NR	Care coaching; Usual care	12 months	EPR+staff+clin_reminder+case-M+Self-M+px_reminder+px_educ	NT_HP_4.1+NT_HP_7.1 +NT_HP_12.5+NT_P_1.3+NT_P_1.4+NT_P_4.1+NT_P_7.1+NT_P_12.2	Defined Sustainability	NA	Charitable trust
McCluskey, 2016[127]	Jul 2009 to Dec 2013 (53 months)	Provider training; Control	12 months	AF+staff; staff	NT_HP_1.1+NT_HP_1.2+NT_HP_4.1+NT_HP_6.2+T_HP_7.1; NT_HP_9.1		NA	Governmental/Public funding
McElrath [128], 2017	NR	Exercise education; Control	up to 36 months	relay+case-M+px_educ	NT_P_1.3+NT_P_2.4+NT_P_2.7+NT_P_4.1+T_P_8.1+NT_P_12.2+NT_P_12.5+NT_P_12.6	Assessed adherence	home, community, clinic	Governmental/Public funding

**Author, year (*companion report ref. number*)**	**Study duration**	**KT Intervention name**	**Duration of KT Intervention**	**Intervention (EPOC) components**	**Intervention (BCT) components**	**Outcomes included in analysis***	**Setting**	**Funding**
McManus, 2014[129]	Mar 2011 to Jan 2013 (22 months)	Self-management; Usual care	NR	relay+Self-M+px_educ	NT_P_1.1+NT_P_2.4+NT_P_4.1+NT_P_12.5	EQ5D; Defined Sustainability; Assessed sustainability	NR	Governmental/Public funding
Meeuwsen, 2012[130]		Care coordination; Usual care	NR	team+case-M; staff	T_HP_9.1+T_HP_12.2; NT_HP_9.1		clinic	Mixed
Meisinger, 2013[131] (*CR15*, *21*, *31*)	Sep 2008 to Jun 2011 (33 months)	Case management; Control	12 months	case-M+px_educ	T_P_2.1+T_P_3.2+NT_P_13.1	Assessed adherence	home	Governmental/Public funding
Mejhert, 2004[132]	NR	Nurse management; Control	18 months	team+case-M+Self-M+px_educ	NT_P_2.4+NT_P_4.1+NT_P_12.2		clinic	Mixed
Mitchell, 2005[133]	Feb 1999 to Dec 2001 (34 months)	Audit + risk; Audit only; Control	12 months	AF+staff; AF+staff	NT_HP_4.1+NT_HP_6.2; NT_HP_4.1+NT_HP_6.2	Quality of care	NR	Governmental/Public funding
Moher, 2001[134]	Jun 1997 ro Oct 1999 (28 months)	Nurse recall group; GP recall group; Audit group	18 months	team+EPR+relay+AF+staff; EPR+relay+AF; AF	NT_HP_1.3+NT_HP_2.7+NT_HP_3.2+NT_HP_4.1+NT_HP_12.2+NT_HP_12.5; NT_HP_1.3+NT_HP_2.7+NT_HP_3.2+NT_HP_12.5; NT_HP_2.7+NT_HP_6.2	Defined Sustainability; Assessed adherence	clinic	Governmental/Public funding
Morganroth, 2016[135]	Jun 2011 to Jun 2012 (12 months)	Care Manager program; Control	12 months	EPR+AF+staff+clin_reminder	NT_HP_4.1+NT_HP_6.2+NT_HP_7.1 +NT_HP_12.5	Quality of care; Assessed adherence	NA	Mixed
Moriyama, 2009[136]	NR	Self-management education; Usual care	12 months	relay+case-M+Self-M+px_educ+mot_interview; px_educ	NT_P_1.1+NT_P_1.2+NT_P_1.4+NT_P_2.2+NT_P_2.3+NT_P_4.1+NT_P_12.2		NA	Governmental/Public funding
Moy, 2016[38] (*CR25*, *26*)	NR	Web education; Control	12 months	Self-M+px_reminder+px_educ; px_reminder	NT_P_1.1+NT_P_2.2+NT_P_2.3+NT_P_4.1+NT_P_7.1+NT_P_12.2+NT_P_12.5; NT_P_2.3+NT_P_12.5	Assessed adherence	NR	Governmental/Public funding
Murphy, 2009[137] (*CR27*)	NR	Tailored care; Usual care	18 months	staff+Self-M	NT_HP_1.4+NT_HP_4.1+NT_HP_6.1+NT_HP_12.2+NT_P_1.1+NT_P_1.4+NT_P_4.1+NT_P_12.2	SF-12 mental; SF-12 physical	clinic	Mixed
Nguyen, 2013[138]	NR	Internet-based dyspnea self-management program (eDSMP); Face-to-face dyspnea self-management program (fDSMP);	12 months	team+case-M+Self-M+px_reminder+px_educ; team+case-M+Self-M+px_educ+mot_interview; px_educ	T_P_1.4+T_P_2.2+NT_P_2.3+NT_P_3.1+T_P_3.2+NT_P_12.5+NT_P_13.1; T_P_1.4+T_P_2.2+NT_P_2.3+NT_P_3.1+T_P_3.2+NT_P_13.1; NT_P_3.2 +NT_P_13.1	SF-36 mental; SF-36 physical; Assessed adherence; Assessed sustainability	home	Governmental/Public funding

**Author, year (*companion report ref. number*)**	**Study duration**	**KT Intervention name**	**Duration of KT Intervention**	**Intervention (EPOC) components**	**Intervention (BCT) components**	**Outcomes included in analysis***	**Setting**	**Funding**
		General health education control (GHE)						
Ojeda, 2005[139]	NR	Formal education; Usual care	16 months	Self-M+px_educ	NT_P_1.1+NT_P_4.1+NT_P_12.2	MLHFQ; Defined Sustainability; Assessed sustainability	NR	NR
Olaiya, 2017[35] (*CR30*)	NR	Care coordination; Control	12 months	finan+case-M+Self-M+px_educ	NT_P_1.2+NT_P_1.3+NT_P_1.4+NT_P_4.1+NT_P_12.2	Assessed adherence	home, clinic	Governmental/Public funding
De Fine Olivarius, 2001[33] (*CR29*)	1989 to 1998 (108 months)	Structured care; Control	NR	AF+staff+clin_reminder+Self-M; staff	T_HP_1.3+T_HP_1.7+NT_HP_2.7+NT_HP_7.1 +NT_HP_13.2; NT_HP_9.1	Defined Sustainability	clinic	Mixed
Olson, 2009[140]	Sep 2005 to Aug 2007 (23 months)	Pharmacy-managed cardiovascular disease management; Control	24 months	case-M+Self-M+px_reminder+mot_interview; staff+px_reminder	NT_P_1.3+NT_P_2.7+NT_P_3.2 +NT_P_7.1; NT_P_2.7+NT_P_7.1		NR	Research funding body
Ortiz-Bautista, 2017[141]	NR	Nurse-led care; Usual care	NR	team+case-M+px_educ	NT_P_4.1+NT_P_12.2	MLHFQ	NR	Governmental/Public funding
Ostwald, 2014[142]	NR	Home visits; Informational mailing	12 months	case-M+px_educ+px_finan; px_educ+px_finan	NT_C_4.1+NT_C_12.2+NT_P_4.1+NT_P_12.2; NT_C_4.1+NT_P_4.1		NA	Mixed
Palacio, 2015[41]	NR	Motivational interviewing; Educational DVD	12 months	staff+px_educ+mot_interview; px_reminder+px_educ	T_P_1.1+NT_P_1.2+NT_P_1.4 +NT_P_1.5+NT_P_4.1+NT_P_12.2; NT_P_4.1+NT_P_7.1	Defined Sustainability; Assessed adherence; Assessed fidelity	NA	Governmental/Public funding
Peters-Klimm, 2010[143]	NR	Clinician training case management; Usual care	12 months	team+EPR+relay+AF+case-M+Self-M+px_educ	NT_HP_2.7+NT_HP_12.2+NT_HP_12.5+NT_P_1.3+NT_P_2.4+NT_P_4.1+NT_P_12.2	SF-36 mental; SF-36 physical; Assessed adherence	NR	Governmental/Public funding
Piette, 2015[144] (*CR33*)	NR	Mobile Health (mHealth)-CarePartner intervention; Standard Mobile-Health (M-health)	12 months	relay+Self-M+px_reminder+px_educ; relay+Self-M+px_educ	NT_HP_7.1 +NT_C_4.1+NT_C_7.1+NT_P_2.2+NT_P_2.3+NT_P_3.2 +NT_P_7.1+NT_P_12.2+NT_P_12.5; NT_HP_7.1 +NT_C_13.1+NT_P_2.3+NT_P_12.2+NT_P_12.5	MLHFQ; Assessed adherence	home	Governmental/Public funding
Pinnock, 2013[145]	NR	Telemonitoring; Usual care	12 months	relay+Self-M+px_educ; staff+Self-M+px_educ	NT_HP_7.1 +NT_P_2.4+NT_P_3.2 +NT_P_4.1; NT_P_4.1+NT_P_12.5	SGRQ; Assessed adherence	home, clinic	Governmental/Public funding

**Author, year (*companion report ref. number*)**	**Study duration**	**KT Intervention name**	**Duration of KT Intervention**	**Intervention (EPOC) components**	**Intervention (BCT) components**	**Outcomes included in analysis***	**Setting**	**Funding**
Pols, 2017[146]	Jan 2013 to Nov 2015 (34 months)	Provider and patient training; Control	12 months	staff+case-M+px_educ+mot_interview ;	NT_HP_4.1+NT_P_1.2+NT_P _4.1+NT_P_12.2		NR	Government al/Public funding
Rea, 2004[147]	NR	Team care; Usual care	12 months	team+case-M+Self-M+px_educ; staff	NT_HP_3.2+NT_HP_7.1 +NT_HP_9.1+NT_HP_10.1+T _P_1.1+NT_P_1.7+NT_P_5.1; NT_HP_9.1+NT_HP_10.1		clinic	Mixed
Reiber, 2004[148]	NR	Patient feedback; Control	24 months	relay+staff+clin_reminder	NT_HP_4.1+NT_HP_7.1	Assessed adherence	NA	Government al/Public funding
Rovner, 2020[149]	NR	CHW education; OT tailored education	12 months	Self-M+px_educ; Self-M+px_reminder+px_educ	NT_P_4.1+NT_P_12.2; NT_P_1.1+NT_P_1.5+NT_P_4 .1+NT_P_5.1+NT_P_7.1+NT_ P_8.7+NT_P_12.1+T_P_12.2	Assessed fidelity	home	Voluntary body
Rubenstein, 2007[150]	NR	Case management; Usual care	36 months	team+case-M+px_reminder+px_educ	T_P_3.2+NT_P_7.1+NT_P_13. 1	Defined Sustainability; Assessed adherence; Assessed sustainability	clinic	Government al/Public funding
Saal, 2015[151]	Jun 2009 to Oct 2010 (16 months)	Stroke support service; Usual care	12 months	team+case-M+Self-M+px_educ; px_educ	T_P_3.2+NT_P_4.1; NT_P_13.1		NR	Mixed
Sackley, 2015[152]	May 2010 to Feb 2013 (33 months)	Training workshop; Usual care	NR	staff+case-M+Self-M+px_educ; staff	NT_HP_4.1	EQ5D	long term facility	Government al/Public funding
Salinero-Fort,2011[153]	2003 to 2005 (24 months)	Precede Health Promotion Education (PHPE); Conventional Health Promotion Education (CHPE)	24 months	Self-M+px_educ+mot_interview ; Self-M+px_educ	NT_P_1.2+NT_P_2.4+NT_P_4 .1+NT_P_12.6; NT_P_4.1	Assessed adherence	clinic	Government al/Public funding
Salisbury, 2018[154]	NR	3D Review; Usual care	NR	team+EPR+AF+staff+clin_ reminder+finan+case-M+Self-M+px_reminder	NT_HP_4.1+NT_HP_6.2+NT_ HP_7.1 +NT_HP_10.2+NT_HP_12.1+ NT_HP_12.2	Assessed adherence	NR	Government al/Public funding
Samus, 2014[155]	NR	Care coordination; Usual care	18 months	team+EPR+staff+case-M+px_reminder+px_educ	NT_HP_4.1+NT_HP_12.2+NT _HP_12.5+NT_C_1.2+NT_C_4 .1+NT_C_7.1+NT_C_12.2+NT _P_1.2+NT_P_4.1+NT_P_7.1+ NT_P_12.2; T_HP_1.1+NT_HP_13.1	Assessed fidelity	home	Mixed
Sanchez-Nieto, 2016[156]	Feb 2012 to Jan 2014 (23 months)	Self-management; Control	12 months	Self-M+px_educ	NT_P_1.1+NT_P_4.1+NT_P_6 .1 +NT_P_12.6		home, clinic	Commercial

**Author, year (*companion report ref. number*)**	**Study duration**	**KT Intervention name**	**Duration of KT Intervention**	**Intervention (EPOC) components**	**Intervention (BCT) components**	**Outcomes included in analysis***	**Setting**	**Funding**
Sarkadi, 2004[157]	NR	Pharmacist education; Control	12 months	staff+case-M+Self-M	NT_P_1.2+NT_P_1.3+NT_P_2.4+NT_P_4.1+NT_P_11.2+NT_P_12.2	Assessed adherence	NR	Mixed
Schafer, 2018[158]	Oct 2011 to Jul 2013 (21 months)	GP training; Usual care	NR	staff+Self-M+px_educ; staff	NT_HP_4.1	EQ5D	clinic	Governmental/Public funding
Schrader, 2005[159]	NR	Team management; Usual care	12 months	team+staff	NT_HP_4.1+NT_HP_12.2		NA	Governmental/Public funding
Schraeder, 2009[29]	NR	Case management; Control	36 months	team+EPR+staff+clin_reminder+case-M+Self-M+px_educ	NT_P_2.7+NT_P_4.1+NT_P_12.2	SF-36 physical; Defined Sustainability; Assessed adherence	clinic	NR
Shea, 2006[30] (*CR17, 34, 35*)	Dec 2000 to Oct 2003 (34 months)	Telemedicine care; Control	28 months	EPR+relay+staff+case-M+px_educ+px_finan; staff	NT_HP_4.1+NT_P_2.4+NT_P_4.1+NT_P_12.2+NT_P_12.5; NT_HP_4.1+NT_HP_9.1		NA	Governmental/Public funding
Smith, 1999[160]	NR	Nurse management; Usual care	12 months	case-M+Self-M+px_educ; px_educ	NT_P_4.1+NT_P_6.1 +T_P_11.1 +NT_P_11.2+NT_P_12.2+NT_P_12.6; NT_P_4.1		home, hospital, clinic	NR
Solomon, 2012[161]	NR	Motivational interviewing; Informational mailings	NR	staff+px_educ+mot_interview; px_educ	NT_P_3.2 +NT_P_5.1+NT_P_7.1; NT_P_5.1		NR	Governmental/Public funding
Sonnichsen, 2010[162] (*CR11, 12*)	NR	Provider and patient training; Control	12 months	staff+case-M+Self-M+px_educ	NT_P_1.1+T_P_4.1; NT_HP_4.1	Quality of care; Assessed adherence	NR	Mixed
Stewart, 2012[163] (*CR39*)	Jun 2008 to Mar 2011 (33 months)	Clinic-based intervention; Home-based intervention	NR	team+case-M	NT_P_12.2	EQ5D; MLHFQ	home, clinic	Governmental/Public funding
Stewart, 2015[164] (*CR39*)	NR	Safety intervention; Usual care	24 months	case-M+Self-M+px_educ	NT_P_1.2+NT_P_1.3+NT_P_1.4+NT_P_4.1+NT_P_12.2	SF-12 mental; SF-12 physical; EQ5D	home, clinic	Mixed
Subramanian, 2004[165]	NR	Guideline-based care; Usual care	12 months	staff+clin_reminder; staff+clin_reminder	NT_HP_4.1+NT_HP_7.1 +T_HP_9.1; NT_HP_4.1+NT_HP_7.1 +NT_HP_9.1	SF-36 mental; SF-36 physical; Assessed adherence	NA	Governmental/Public funding
Suominen, 2015[166]	NR	Nutrition advice; Usual care	NR	case-M+px_educ; px_educ	NT_P_1.1+NT_P_2.2+NT_P_2.3+NT_P_4.1+NT_P_12.2		home, clinic	Governmental/Public funding
Tjia, 2015[32]	NR	Toolkit + audit + academic detailing; Toolkit + audit &	12 months	AF+staff; AF+staff; staff	NT_HP_2.7+NT_HP_4.1+NT_HP_6.2+NT_HP_9.1; NT_HP_2.7+NT_HP_6.2+NT_HP_9.1; NT_HP_9.1		NA	Governmental/Public funding

**Author, year (*companion report ref. number*)**	**Study duration**	**KT Intervention name**	**Duration of KT Intervention**	**Intervention (EPOC) components**	**Intervention (BCT) components**	**Outcomes included in analysis***	**Setting**	**Funding**
		feedback; Toolkit						
Tomita, 2009[167]	NR	Computer support; Usual care	12 months	team+Self-M+px_reminder+px_educ	NT_P_1.1+NT_P_2.3+NT_P_2.7+NT_P_4.1+NT_P_12.5	Defined Sustainability; Assessed adherence	NA	Governmental/Public funding
Tremont, 2008[84]	NR	Telephone Tracking-Dementia (FITT-D) intervention; Control	12 months	Self-M+px_educ+mot_interview; px_educ	NT_C_1.2+NT_C_4.1+NT_C_11.2+NT_C_12.2	Assessed adherence	NA	Governmental/Public funding
Trento, 2010[168]	4 years (48 months)	Group care; Usual care	24 months	staff+Self-M+px_educ	NT_P_4.1	Defined Sustainability	clinic	NR
Trofimov, 2015[39]	NR	Patient education; Control	12 months	px_educ	NT_P_4.1		home, hospital	NR
Vaillant-Roussel, 2016[169]	NR	Patient education; Usual care	19 months	staff+case-M+Self-M+px_educ+mot_interview	NT_HP_4.1+NT_HP_6.1+NT_P_1.3+NT_P_4.1	SF-36 mental; SF-36 physical; MLHFQ; Assessed adherence	clinic	Mixed
van de Ven, 2013[170] (*CR37*)	Oct 2010 to Apr 2012 (18 months)	Provider education; Usual care	NR	staff	NT_HP_1.4+NT_HP_4.1+NT_HP_8.1	EQ5D	NA	Research funding body
van Der Aa, 2015[171]	Jul 2012 to Jul 2015 (36 months)	Outpatient rehabilitation; Usual care	12 months	case-M+Self-M+px_educ	NT_P_1.2+NT_P_1.3+NT_P_1.4+NT_P_4.1+NT_P_11.2+NT_P_12.2	EQ5D; Assessed adherence; Assessed fidelity	home, clinic	Research funding body
Vickrey, 2006 [26]	NR	Care management; Usual care	12 months	hs EPR+staff+case-M+Self-M	NT_HP_4.1+NT_HP_12.5+T_C_1.1+NT_C_1.2+NT_C_1.4+T_C_3.2	Assessed adherence	home, community	Mixed
Vinereanu, 2017[172]	NR	Education and feedback; Control	12 months	EPR+AF+staff+px_educ	NT_HP_2.2+NT_HP_9.1+T_HP_13.2+NT_C_13.1+NT_P_13.1	Quality of care; Assessed adherence	NR	Commercial
Wagenaar, 2019[173]	NR	E-Vita platform; Usual care website; Usual care	12 months	EPR+relay+case-M+px_reminder+px_educ; Self-M+px_reminder	NT_HP_7.1 +NT_HP_12.5+NT_P_1.2+NT_P_1.3+NT_P_2.4+NT_P_2.7+NT_P_4.1+NT_P_7.1+NT_P_12.2+NT_P_12.5; NT_P_1.2+NT_P_4.1+NT_P_7.1+NT_P_12.5	MLHFQ	clinic	Charitable trust
Waldorff, 2012[174] (*CR32*)	NR	Daisy intervention; Usual care	12 months	Self-M+px_educ+mot_interview	T_C_3.3+NT_C_13.1+T_P_3.3+NT_P_13.1	Assessed adherence	home, clinic	Governmental/Public funding
Walters, 2013[175]	May 2008 to Dec 2010 (31 months)	Health mentoring; Usual care	12 months	staff+case-M+Self-M+px_educ	NT_HP_4.1+NT_HP_12.2+NT_P_1.1+NT_P_1.2+NT_P_1.4+T_P_4.1 +NT_P_11.2+NT_P_12.2	SF-36 mental; SF-36 physical; SGRQ; Assessed adherence; Assessed fidelity	home, clinic	Mixed

Author, year (*companion report ref. number*)	Study duration	KT Intervention name	Duration of KT Intervention	Intervention (EPOC) components	Intervention (BCT) components	Outcomes included in analysis*	Setting	Funding
Wetering, 2010[176]	NR	Rehabilitation; Usual care	4 months with 20 months maintenance	team+Self-M+px_educ+mot_interview ; px_educ	NT_P_3.1+NT_P_4.1+NT_P_5 .1+NT_P_7.1+NT_P_12.2; NT_P_5.1+NT_P_11.1	SGRQ; Defined Sustainability; Assessed adherence	home, clinic	Mixed
Whittle, 2014[177]	NR	Self-monitoring (education seminar); Peer-led intervention	12 months	Self-M+px_educ; Self-M+px_educ	NT_P_4.1+NT_P_12.5; NT_P_13.1	Assessed fidelity	NA	Governmental/Public funding
Wilcock, 2013[178] (*CR16*)	36 months	Educational workshops; Control	24 months	staff; staff	NT_HP_4.1+NT_HP_12.1; NT_HP_4.1		NA	Governmental/Public funding
Wright, 2001[179]	NR	Care education; Control	12 months	case-M+Self-M+px_educ	NT_C_1.2+NT_C_1.3 +NT_C_4.1+NT_C_11.2+NT_C_12.2		NA	Charitable trust
Xi, 2015[180]	Feb 2010 to Apr 2012 (26 months)	Respiratory training; Educational program	12 months	Self-M+px_educ; px_educ	NT_P_2.5+NT_P_4.1; NT_P_13.1	SGRQ; Defined Sustainability; Assessed sustainability	home, clinic	Governmental/Public funding
Xiao, 2016[181]	NR	Care coordination; Usual care	12 months	staff+case-M+Self-M; px_educ	NT_C_1.2+NT_C_1.3 +NT_C_2.4+NT_C_2.7+NT_C_4.1+NT_C_12.2; NT_C_4.1+NT_C_12.2	SF-36 mental (g); SF-36 physical; Defined Sustainability, Assessed fidelity	NA	Research funding body
Zwar, 2016[182]	Feb 2011 to Aug 2013 (30 months)	Team-based management; Usual care	18 months	EPR+staff; staff	NT_HP_1.3+NT_HP_2.2+NT_HP_4.1+T_HP_12.1; NT_HP_2.2+NT_HP_4.1	SGRQ; Quality of care (h); Assessed adherence	clinic	Governmental/Public funding

Abbreviations: EQ5D, EuroQoL Quality of Life Scale (EQ-5D); EPOC, Effective Practice and Organisation of Care (EPOC) Taxonomy; MLHFQ , Minnesota Living with Heart Failure Questionnaire; NA, Not Applicable; NR, Not Reported**;** SF-12 mental, 12 item Short-Form Health Survey (SF-12) – mental component; SF-12 physical, 12 item Short-Form Health Survey (SF-12) – physical component; SF-36 mental, 36 item Short-Form Health Survey (SF-36) – mental component; SF-36 physical, 36 item Short-Form Health Survey (SF-36) – physical component; SGRQ, St George's Respiratory Questionnaire; NR, not reported; CR, companion report;

***: Sustainability** is defined as the extent to which a KT intervention continues after adoption was initiated; **fidelity** refers to the consistency and quality of targeted organizational members' use of the specific innovation; **adherence** refers to the extent to which individuals implementing the intervention confirm to the intervention protocol for at least one year.

Notes:

(a) Study Bucknall, 2012 was excluded from the analysis because data provided are unclear - the study, does not report the final values on the regular EQ-5D score (similar with the provided MD value).
(b) Study Dunagan, 2005 was excluded from the analysis because the data are reported on two MLHFQ sub-scales (physical and emotional) without the total score. All remaining studies report a total score.
(c) Study Galbreath, 2004, the outcomes were not reported for one arm, so the study was treated in MA as two-arm study; however, the study has 3 arms.
(d) Study Karhula, 2015 a & b is one study with two different populations (two chronic diseases), so it is presented separately.

(e) Study Lin, 2018 is an outlier in the EQ-5D outcome so it is excluded from sensitivity analysis.
(f) Study Maltais, 2008 was excluded from the analysis as it compared the same exact components in both arms [the study includes the interventions hospital-based and home-based, but these include the same exact components (patient education and promotion of self-management) in both arms.
(g) Study Xiao, 2016 is an outlier in the SF-36 item Short-Form Mental Health Survey so it is excluded from sensitivity analysis.
(h) Study Zwar, 2016 is an outlier in the Quality-of-Care outcome so it is excluded from sensitivity analysis.

Appendix 10: Individual Patient Characteristics

Author, Year	Country	Study Design	Sample Size	Overall Age (Years), Age Type (Variance)	Female (%)	Duration of follow up (Months)	Sites
Ansari, 2003[43]	USA	RCT	169	NR	NR	14	single site
Baker, 2003[44]	UK	Cluster RCT	3151	NR	NA	12	multi site
Batchelor, 2012[45]	Australia	RCT	156	NR	36.54	12	multi site
Beck, 1997[46]	USA	Cluster RCT	321	NR	66.49	12	multi site
Beck, 2017[47]	USA	RCT	195	66.4, mean (8.1 SD)	46.7	12	multi site
Bekelman, 2015[48]	USA	RCT	392	68, mean (NR)	NR	12	multi site
Benzo, 2016[49]	USA	RCT	215	NR	NR	12	multi site
Blue, 2001[50]	UK	RCT	165	NR	42.42	12	single site
Bohingamu, 2019[51]	Australia	RCT	171	NR	48	12	single site
Bohm, 2016[52]	Germany	RCT	1002	66.3, mean (10.4 SD)	20.30	24	multi site
Team CPMMPE, 2007[53]	UK	RCT	1614	NR	31.51	12	multi site
Bosanquet, 2017[54]	UK	RCT	485	72, mean (NR)	62.05	18	multi site
Bourbeau, 2003[55]	Canada	RCT	191	NR	NR	12	multi site
Boyne, 2012[56]	Netherlands	RCT	382	71, mean (11 SD)	41	12	multi site
Bruce, 2004[57]	USA	Cluster RCT	598	NR (60 to 94 range)	71.61	12	multi site
Bruce, 2015[58]	USA	Cluster RCT	327	76.5, mean (8 SD)	69.60	12	multi site
Bucknall, 2012[59]	UK	RCT	464	69.1, mean (9.3 SD)	NR	12	multi site
Burns, 1995[60]	USA	RCT	130	NR	NR	12	single site
Burns, 2003[61]	USA	RCT	334	NR	55.29	24	multi site
Callahan, 2006[62]	USA	RCT	380	NR	43.14	18	multi site
Campbell, 1998[63]	UK	RCT	1173	66.1, mean (NR)	NR	12	multi site
Chen, 2015[36]	China	Cluster RCT	360	NR	63.50	12	multi site
Chi, 2012[64]	China	RCT	171	NR	NR	12	NR
Ciaschini, 2010[65]	Canada	RCT	201	71.9, mean (7.2 SD)	93.54	12	multi site
Cleveringa, 2008[66]	Netherlands	Cluster RCT	3391	NR	NR	12	multi site
Coleman, 1999[67]	USA	Cluster RCT	264	NR	48.5	24	multi site
Coull, 2004[68]	UK (Scotland)	RCT	320	NR	39.48	12	single site
de la Porte, 2007[69]	Netherlands	RCT	240	71, mean (NR)	NR	12	multi site
de Lusignana, 2013[70]	UK	Cluster RCT	691626	75.04, mean (11.82 SD)	0.50	24	multi site
DeBusk, 2004[71]	USA	RCT	462	72, mean (11 SD)	48.92	12	multi site
Del Sindaco, 2007[72]	Italy	RCT	173	77, mean (6 SD)	48.00	24	multi site
DeVore, 2015[73]	USA	Cluster RCT	71829	NR	48.6	12	multi site

Author, Year	Country	Study Design	Sample Size	Overall Age (Years), Age Type (Variance)	Female (%)	Duration of follow up (Months)	Sites
Dopp, 2015[74]	Netherlands	Cluster RCT	285	NR	45.06	12	multi site
Dracup, 2014[42]	USA	RCT	614	66.1, mean (12.9 SD)	40.5	24	multi site
Dunagan, 2005[75]	USA	RCT	151	NR	56.29	12	multi site
Eccles, 2007[34]	UK (England)	Cluster RCT	3608	NR	NR	15	multi site
Eckert, 2010[76]	Australia	Cluster RCT	321	70, mean (10 SD)	NR	12	multi site
Ell, 2007[77]	USA	RCT	486	NR	72.3	12	multi site
Fan, 2012[78]	USA	RCT	426	NR	3.05	12	multi site
Federman, 2019[79]	USA	RCT	391	67.8, mean (7.4 SD)	85	12	multi site
Fihn, 2004[80]	USA	Cluster RCT	23308	65, mean (10 SD)	NR	24	multi site
Fihn, 2011[81]	USA	Cluster RCT	986	68, mean (NR)	NR	20	multi site
Forster, 1996[82]	UK	RCT	240	NR	47	12	single site
Forster, 2015[83]	UK	Cluster RCT	1008	NR	NR	12	multi site
Fortinsky, 2009[183]	USA	RCT	226	NR	64.21	12	multi site
Freund, 2016[85]	Germany	Cluster RCT	2208	NR	52.00	24	multi site
Galbreath, 2004[86]	USA	RCT	1069	70.9, mean (10.3 SD)	29	18	single site
Gallagher, 1997[87]	USA	RCT	363	69, mean (NR)	62.87	36	multi site
Gaugler, 2008[88]	USA	RCT	812	NR	NR	190.8	single site
Gellis, 2012[89]	USA	RCT	115	79, mean (NR)	65.68	12	single site
Goderis, 2010[27]	Belgium	Cluster RCT	2637	NR	51.74	22	multi site
Graven, 2016[90]	Australia	RCT	110	69.9, mean (14.2 SD)	NR	12	multi site
Haerter, 2016[91]	Germany	RCT	10815	NR	60.39	24	multi site
Heisler, 2012[28]	USA	Cluster RCT	4622	NR	NR	14	multi site
Hendriks, 2012[92]	Netherlands	RCT	712	NR	41.29	12	single site
Hernandez, 2015[93]	Spain	RCT	155	NR	15.48	12	single site
Hetlevik, 2000[94]	Norway	Cluster RCT	1090	NR	54.03	18	multi site
Hogg, 2009[95]	Canada	RCT	241	NR	NR	14.9	multi site
Holbrook, 2011[96]	Canada	RCT	1102	69.1, mean (8.7 SD)	53.4	12	multi site
Holm, 2002[97]	Denmark	RCT	1567	70, median (17.0 to 91.0 range)	NR	24	single site
Holt, 2010[98]	UK	RCT	38147	NR	NR	24	multi site
Hughes, 2000[99]	USA	RCT	3849	70.4, mean (10.3 SD)	NR	12	multi site
Hunger, 2015[100]	Germany	RCT	340	NR, (65 to 92 range)	NR	12	single site
Irewall, 2015[101]	Sweden	RCT	537	70.8, mean (10.7 SD)	43	12	single site

Author, Year	Country	Study Design	Sample Size	Overall Age (Years), Age Type (Variance)	Female (%)	Duration of follow up (Months)	Sites
Jaarsma, 2008[102]	Netherlands	RCT	1023	71, mean (11 SD)	38.00	18	multi site
Joling, 2012[103]	Netherlands	RCT	384	NR	32.3	12	multi site
Karhula, 2015[104]	Finland	RCT	595	67.7, mean (8.97 SD)	40.80	12	single site
Kennedy, 2013[37]	UK	Cluster RCT	5863	NR	0.54	12	multi site
Kennedy, 2015[105]	Canada	Cluster RCT	5478	84.4, mean (10.9 SD)	70.82	12	multi site
Khdour, 2009[106]	UK	RCT	173	67, mean (NR)	56.05	12	single site
Kiefe, 2001[31]	USA	Cluster RCT	2028	NR	NR	17	multi site
Kim, 2014[107]	USA	RCT	440	70.9, mean (5.3 SD)	69.9	18	multi site
Ko, 2017[108]	Hong Kong	RCT	180	74.7, mean (8.2 SD)	NR	12	single site
Kruis, 2014[109]	Netherlands	Cluster RCT	2886	NR	NR	24	multi site
Krum, 2013[110]	Australia	Cluster RCT	548	NR	36.93	12	multi site
Kurz, 2010[111]	Austria; Switzerland; Germany	RCT	584	76, mean (NR)	63.7	15	multi site
Kalter-Leibovici, 2017[112]	Israel	RCT	1360	70.7, mean (11.3 SD)	NR	32.4	multi site
Leveille, 1998[113]	USA	RCT	201	NR	55.74	12	multi site
Leventhal, 2011[114]	Switzerland	RCT	42	77, mean (6.5 SD)	38.1	12	single site
Levine, 2011[115]	USA	Cluster RCT	16694	66, mean (10 SD)	NR	22	multi site
Li, 2012[116]	China	Cluster RCT	248	65.34, mean (12.25 SD)	60.89	18	multi site
Licskai, 2016[117]	Canada	RCT	181	67.7, mean (10.2 SD)	54.50	12	multi site
Lin, 2018[118]	Taiwan	RCT	178	NR	NR	12	multi site
Liu, 2008[119]	Taiwan	RCT	48	NR	NR	12	NR
Lopez Cabezas, 2006[120]	Spain	RCT	134	75, mean (NR)	55.97	12	multi site
Lowrie, 2014[121]	UK (Scotland)	Cluster RCT	4051	NR	NR	20.4	multi site
Machline-Carrion, 2019[122]	Brazil	Cluster RCT	1619	65.6, mean (10.5 SD)	36	12	multi site
Mahoney, 2003[123]	USA	RCT	200	NR	48.02	18	multi site
Maltais, 2008[124]	Canada	RCT	252	NR	44.50	12	multi site
Markle-Reid, 2011[125]	Canada	RCT	101	NR	NR	12	multi site
Markun, 2015[126]	Switzerland	RCT	169	76.7, mean (8 SD)	63.3	12	multi site
McCluskey, 2016[127]	Australia	Cluster RCT	22	NR	NR	12	multi site
McElrath, 2017[128]	USA	RCT	140	NR (50 to 85 range)	NR	36	multi site

Author, Year	Country	Study Design	Sample Size	Overall Age (Years), Age Type (Variance)	Female (%)	Duration of follow up (Months)	Sites
McManus, 2014[129]	UK	RCT	555	NR	NR	12	multi site
Meeuwsen, 2012[130]	Netherlands	RCT	350	78.1, mean (5.7 SD)	61.00	12	multi site
Meisinger, 2013[131]	Germany	RCT	340	75.4, mean (6 SD)	NR	21	single site
Mejhert, 2004[132]	Sweden	RCT	208	75.8, mean (7.1 SD)	NR	18	single site
Mitchell, 2005[133]	UK (Scotland)	RCT	1755	NR (65 to 79 range)	56.17	27	multi site
Moher, 2001[134]	UK	Cluster RCT	1906	55-75, range (NR)	13.8	18	multi site
Morganroth, 2016[135]	USA	Cluster RCT	242	69, mean (66, 72 95% (CI))	63	12	multi site
Moriyama, 2009[136]	Japan	RCT	65	NR	53.85	12	multi site
Moy, 2016[38]	USA	RCT	238	66.8, mean (8.8 SD)	6.3	12	multi site
Murphy, 2009[137]	Northern Ireland, Ireland	Cluster RCT	903	NR	NR	18	multi site
Nguyen, 2013[138]	USA	RCT	125	68.7, mean (9.7 SD)	46.00	12	multi site
Ojeda, 2005[139]	Spain	RCT	153	NR	NR	12	multi site
Olaiya, 2017[35]	Australia	Cluster RCT	563	70.1, median (60.9, 78.6 IQR (1,3))	35.49	12	multi site
De Fine Olivarius, 2001[33]	Denmark	Cluster RCT	1954	NR	NR	28	multi site
Olson, 2009[140]	USA	RCT	421	72, mean (NR)	26	24	multi site
Ortiz-Bautista, 2017[141]	Spain	RCT	127	75, mean (12 SD)	31	24	NR
Ostwald, 2014[142]	USA	RCT	318	NR	25.16	12	multi site
Palacio, 2015[41]	USA	RCT	452	69.5, mean (8.8 SD)	42	12	multi site
Peters-Klimm, 2010[143]	Germany	RCT	228	NR	NR	12	multi site
Piette, 2015[144]	USA	RCT	662	67.8, mean (10.2 SD)	NR	12	multi site
Pinnock, 2013[145]	UK	RCT	256	69.4, mean (8.6 SD)	55.00	12	multi site
Pols, 2017[146]	Netherlands	Cluster RCT	315	67.5, mean (10 SD)	45	12	multi site
Rea, 2004[147]	New Zealand	Cluster RCT	251	68, mean (44-84 range)	58.52	12	multi site
Reiber, 2004[148]	USA	Cluster RCT	5721	65.7, mean (NR)	NR	24	multi site
Rovner, 2020[149]	USA	RCT	101	68.4, mean (6.4 SD)	62	12	single site
Rubenstein, 2007[150]	USA	RCT	792	74, mean (NR)	3.00	36	single site
Saal, 2015[151]	Germany	RCT	265	NR	48.30	12	multi site
Sackley, 2015[152]	UK	Cluster RCT	1042	82.9, mean (NR)	NR	12	multi site
Salinero-Fort, 2011[153]	Spain	RCT	638	66.7, mean (14.5 SD)	51.6	24	multi site

Author, Year	Country	Study Design	Sample Size	Overall Age (Years), Age Type (Variance)	Female (%)	Duration of follow up (Months)	Sites
Salisbury, 2018[154]	UK	Cluster RCT	1546	71, mean (NR)	50.52	12	multi site
Samus, 2014[155]	USA	RCT	592	83.9, mean (5.9 SD)	63.7	18	multi site
Sanchez-nieto, 2016[156]	Spain	RCT	85	67.6, mean (6.9 SD)	9.35	12	multi site
Sarkadi, 2004[157]	Sweden	RCT	77	NR	NR	24	multi site
Schafer, 2018[158]	Germany	Cluster RCT	650	NR	54.64	12	multi site
Schrader, 2005[159]	Australia	RCT	1149	18 to 84, range (NR)	NR	12	multi site
Schraeder, 2009[29]	USA	RCT	1977	76, mean (NR)	53.07	36	multi site
Shea, 2006[30]	USA	Cluster RCT	1665	71, mean (NR)	62.8	12	multi site
Smith, 1999[160]	Australia	RCT	96	NR	39.58	12	multi site
Solomon, 2012[161]	USA	RCT	2097	78, mean (NR)	93.8	12	multi site
Sonnichsen, 2010[162]	Austria	Cluster RCT	1581	NR	47.82	12	multi site
Stewart, 2012[163]	Australia	RCT	280	71, mean (14 SD)	NR	18	multi site
Stewart, 2015[164]	Australia	RCT	335	72, mean (11 SD)	NR	24	multi site
Subramanian, 2004[165]	USA	RCT	811	68, mean (9 SD)	NR	12	multi site
Suominen, 2015[166]	Finland	RCT	156	77.4, mean (NR)	NR	12	multi site
Tjia, 2015[32]	USA	Cluster RCT	42	NR	NR	12	multi site
Tomita, 2009[167]	USA	RCT	40	76.2, mean (NR)	67.5	12	multi site
Tremont, 2008[84]	USA	RCT	120	NR	NR	12	multi site
Trento, 2010[168]	Italy	RCT	815	NR	49.33	48	multi site
Trofimov, 2015[39]	Russia	RCT	120	71, mean (NR)	NR	12	NR
Vaillant-Roussel, 2016[169]	France	Cluster RCT	325	74, mean (10.5 SD)	NR	19	multi site
van de Ven, 2013[170]	Netherlands	Cluster RCT	810	84.7, mean (6.3 SD)	75.10	12	multi site
Van Der Aa, 2015[171]	Netherlands, Belgium	RCT	265	50 to 98, range (NR)	70	24	multi site
Vickrey, 2006 [26]	USA	Cluster RCT	816	80.1, mean (NR)	54.91	22.5	multi site
Vinereanu, 2017[172]	Argentina; Brazil; China; India; Romania	Cluster RCT	2281	NR	47.04	12	multi site
Wagenaar, 2019[173]	The Netherlands ;	RCT	450	66.8, mean (11 SD)	NR	12	multi site
Waldorff, 2012[174]	Denmark	RCT	660	76.2, mean (NR)	54.01	12	multi site

Author, Year	Country	Study Design	Sample Size	Overall Age (Years), Age Type (Variance)	Female (%)	Duration of follow up (Months)	Sites
Walters, 2013[175]	Australia	Cluster RCT	182	68, mean (NR)	NR	12	multi site
Van Wetering, 2010[176]	Netherlands	RCT	199	NR	NR	24	multi site
Whittle, 2014[177]	USA	Cluster RCT	404	NR	NR	12	multi site
Wilcock, 2013[178]	UK	Cluster RCT	1072	NR	65.95	12	multi site
Wright, 2001[179]	USA	RCT	186	NR	NR	12	single site
Xi, 2015[180]	China	RCT	70	NR	25.00	12	single site
Xiao, 2016[181]	Australia	RCT	122	83, median (76.0 to 86.0 IQR)	60.7	12	multi site
Zwar, 2016[182]	Australia	Cluster RCT	364	66, mean (NR)	NR	12	multi site

Abbreviations: NR, Not Reported; RCT, Randomized Controlled Trial

Appendix 11: Sustainability of KT Interventions Summarized Results

Summarized by combination of components			**Assessed sustainability**		**Assessed adherence**		**Assessed fidelity**	
**EPOC coding**	**N**	**Percent (%)**	**No**	**Yes**	**No**	**Yes**	**No**	**Ye s**
AF	1	0.3	1	0	0	1	1	0
AF+staff	7	2.1	7	0	6	1	7	0
AF+staff+	1	0.3	1	0	1	0	1	0
AF+staff+Self-M+mot_interview	1	0.3	1	0	1	0	1	0
AF+staff+Self-M+px_educ	1	0.3	1	0	0	1	1	0
AF+staff+case-M+px_educ	1	0.3	1	0	0	1	0	1
AF+staff+clin_reminder+Self-M	1	0.3	1	0	1	0	1	0
AF+staff+finan+px_educ	1	0.3	1	0	1	0	1	0
EPR+AF+staff+clin_reminder	1	0.3	1	0	0	1	1	0
EPR+AF+staff+px_educ	1	0.3	1	0	0	1	1	0
EPR+clin_reminder	1	0.3	1	0	1	0	0	1
EPR+relay+AF	1	0.3	1	0	0	1	1	0
EPR+relay+AF+staff	1	0.3	1	0	0	1	0	1
EPR+relay+cQI+AF	1	0.3	1	0	0	1	1	0
EPR+relay+cQI+AF+staff	1	0.3	1	0	0	1	1	0
EPR+relay+case-M+px_reminder+px_educ	1	0.3	1	0	1	0	1	0
EPR+relay+staff+case-M+px_educ+px_finan	1	0.3	1	0	1	0	1	0
EPR+staff	1	0.3	1	0	0	1	1	0
EPR+staff+clin_reminder+case-M+Self-M+px_reminder+px_educ	1	0.3	1	0	1	0	1	0
EPR+staff+clin_reminder+px_educ	1	0.3	1	0	0	1	1	0
EPR+staff+clin_reminder+px_reminder	1	0.3	1	0	0	1	1	0
EPR+staff+mot_interview	1	0.3	1	0	0	1	1	0
Self-M	1	0.3	1	0	1	0	1	0
Self-M+px_educ	13	3.95	9	3	6	6	9	3
Self-M+px_educ+mot_interview	5	1.52	5	0	2	3	5	0
Self-M+px_educ+px_finan	1	0.3	1	0	1	0	1	0
Self-M+px_reminder	1	0.3	1	0	1	0	1	0
Self-M+px_reminder+px_educ	4	1.2	3	0	2	1	2	1
UC	92	28.1	84	5	66	23	86	3
cQI+AF	1	0.3	1	0	1	0	1	0

cQI+AF+	1	0.3	1	0	1	0	1	0
cQI+AF+staff	1	0.3	1	0	1	0	0	1
case-M+Self-M	1	0.3	0	1	1	0	1	0
case-M+Self-M+px_educ	10	3.04	8	2	7	3	9	1
case-M+Self-M+px_reminder+mot_interview	1	0.3	1	0	1	0	1	0
case-M+px_educ	6	1.82	6	0	4	2	6	0
case-M+px_educ+px_finan	2	0.61	2	0	2	0	2	0
finan+case-M+Self-M+px_educ	1	0.3	1	0	0	1	1	0
hs EPR+staff+case-M+Self-M	1	0.3	1	0	0	1	1	0
hs finan+team+AF+staff+clin_reminder+px_educ+mot_interview	1	0.3	1	0	1	0	1	0
hs finan+team+relay+staff+case-M	1	0.3	1	0	1	0	1	0
px_educ	24	7.29	21	3	20	4	23	1
px_educ+px_finan	1	0.3	1	0	1	0	1	0
px_reminder	1	0.3	1	0	0	1	1	0
px_reminder+px_educ	1	0.3	1	0	1	0	1	0
relay+Self-M+px_educ	3	0.91	2	1	1	2	3	0
relay+Self-M+px_reminder+px_educ	2	0.61	2	0	0	2	2	0
relay+Self-M+px_reminder+px_educ+px_finan	1	0.3	0	1	1	0	1	0
relay+cQI+staff	1	0.3	1	0	0	1	1	0
relay+case-M	1	0.3	1	0	0	1	1	0
relay+case-M+Self-M+px_educ	5	1.52	3	1	3	1	4	0
relay+case-M+Self-M+px_educ+mot_interview	1	0.3	1	0	1	0	1	0
relay+case-M+Self-M+px_reminder+px_educ+px_finan	1	0.3	0	1	1	0	1	0
relay+case-M+px_educ	2	0.61	2	0	1	1	2	0
relay+px_educ	2	0.61	2	0	1	1	2	0
relay+px_reminder+px_educ	1	0.3	1	0	1	0	1	0
relay+staff	1	0.3	1	0	1	0	1	0
relay+staff+case-M+Self-M+mot_interview	1	0.3	1	0	0	1	0	1
relay+staff+clin_reminder	1	0.3	1	0	0	1	1	0
staff	26	7.9	26	0	19	7	25	1
staff+Self-M	2	0.6	2	0	1	1	1	1
staff+Self-M+px_educ	5	1.5	5	0	4	1	5	0
staff+case-M+Self-M	2	0.6	2	0	1	1	1	1
staff+case-M+Self-M+px_educ	5	1.5	5	0	2	3	4	1
staff+case-M+Self-M+px_educ+mot_interview	2	0.6	2	0	0	2	1	1
staff+case-M+px_educ+mot_interview	1	0.3	1	0	1	0	1	0
staff+clin_reminder	4	1.2	4	0	0	4	3	1
staff+clin_reminder+px_reminder	1	0.3	1	0	0	1	1	0

staff+px_educ	2	0.6	2	0	2	0	2	0
staff+px_educ+mot_interview	2	0.6	2	0	1	1	1	1
staff+px_reminder	1	0.3	1	0	1	0	1	0
team+EPR+AF+staff+clin_reminder+finan+case-M+Self-M+px_reminder	1	0.3	1	0	0	1	1	0
team+EPR+case-M+Self-M+px_educ	1	0.3	1	0	1	0	1	0
team+EPR+relay+AF+case-M+Self-M+px_educ	1	0.3	1	0	0	1	1	0
team+EPR+relay+AF+staff	1	0.3	1	0	0	1	1	0
team+EPR+relay+case-M+Self-M	1	0.3	1	0	0	1	0	1
team+EPR+relay+case-M+Self-M+px_educ+mot_interview	1	0.3	1	0	0	1	0	1
team+EPR+staff+case-M+px_reminder+px_educ	1	0.3	1	0	1	0	0	1
team+EPR+staff+clin_reminder+case-M+Self-M+px_educ	1	0.3	1	0	0	1	1	0
team+Self-M+px_educ+mot_interview	1	0.3	1	0	0	1	1	0
team+Self-M+px_reminder+px_educ	1	0.3	1	0	0	1	1	0
team+case-M	5	1.5	5	0	4	1	4	1
team+case-M+Self-M+px_educ	7	2.1	6	1	6	1	7	0
team+case-M+Self-M+px_educ+mot_interview	2	0.6	1	1	0	2	2	0
team+case-M+Self-M+px_reminder+px_educ	1	0.3	0	1	0	1	1	0
team+case-M+px_educ	3	0.9	3	0	3	0	3	0
team+case-M+px_finan	1	0.3	1	0	1	0	1	0
team+case-M+px_reminder+px_educ	2	0.6	1	1	1	1	2	0
team+px_educ	1	0.3	1	0	1	0	1	0
team+relay+Self-M+px_educ	1	0.3	1	0	1	0	1	0
team+relay+case-M	1	0.3	0	1	1	0	1	0
team+relay+case-M+Self-M+px_educ	2	0.6	1	1	1	1	2	0
team+relay+case-M+px_educ	1	0.3	1	0	0	1	1	0
team+relay+staff+case-M+Self-M+px_educ	2	0.6	2	0	1	1	2	0
team+relay+staff+case-M+Self-M+px_educ+mot_interview	1	0.3	1	0	0	1	1	0
team+relay+staff+case-M+Self-M+px_reminder+px_educ	1	0.3	1	0	1	0	1	0
team+relay+staff+clin_reminder+case-M+Self-M+px_educ	1	0.3	1	0	0	1	1	0
team+staff	2	0.6	2	0	2	0	2	0
team+staff+case-M	1	0.3	1	0	0	1	1	0
team+staff+case-M+Self-M	1	0.3	1	0	1	0	1	0
team+staff+case-M+Self-M+px_educ	3	0.9	3	0	0	3	2	1
team+staff+case-M+Self-M+px_educ+px_finan	1	0.3	1	0	1	0	1	0
team+staff+case-M+px_reminder+px_educ	1	0.3	1	0	0	1	1	0
team+staff+finan+case-M+Self-M+px_educ	1	0.3	1	0	1	0	1	0
**Total**	**327**	**100**	**297**	**25**	**207**	**115**	**295**	**27**

Summarized by combination of components			Assessed sustainability		Assessed adherence		Assessed fidelity	
**BCT coding**	**N**	**Percent (%)**	**No**	**Yes**	**No**	**Yes**	**No**	**Yes**
NT_C_1.2+NT_C_1.3 +NT_C_1.4+NT_C_4.1	1	0.3	1	0	1	0	1	0
NT_C_1.2+NT_C_1.3 +NT_C_1.4+NT_C_4.1+NT_C_12.2	1	0.3	0	1	1	0	1	0
NT_C_1.2+NT_C_1.3 +NT_C_2.4+NT_C_2.7+NT_C_4.1+NT_C_12.2	1	0.3	1	0	1	0	0	1
NT_C_1.2+NT_C_1.3 +NT_C_4.1+NT_C_11.2+NT_C_12.2	1	0.3	1	0	1	0	1	0
NT_C_1.2+NT_C_1.3 +NT_C_4.1+NT_C_13.1	1	0.3	1	0	1	0	1	0
NT_C_1.2+NT_C_4.1+NT_C_11.2+NT_C_12.2	2	0.61	2	0	0	2	1	1
NT_C_1.2+NT_C_4.1+NT_C_12.2	2	0.61	2	0	2	0	2	0
NT_C_1.2+NT_C_4.1+NT_C_12.2+NT_P_1.2+NT_P _4.1+NT_P_12.2	1	0.3	1	0	0	1	1	0
NT_C_1.2+NT_C_4.1+NT_C_7.1+NT_C_12.2+NT_C _12.4+NT_C_12.5	1	0.3	1	0	1	0	1	0
NT_C_3.1	1	0.3	1	0	1	0	1	0
NT_C_4.1+NT_C_12.2	1	0.3	1	0	1	0	1	0
NT_C_4.1+NT_C_12.2+NT_P_4.1+NT_P_12.2	1	0.3	1	0	1	0	1	0
NT_C_4.1+NT_P_4.1	1	0.3	1	0	1	0	1	0
NT_HP_1.1+NT_HP_1.2+NT_HP_4.1+NT_HP_12.2+ NT_P_1.2+NT_P_1.3+NT_P_4.1+NT_P_12.2	1	0.3	1	0	1	0	1	0
NT_HP_1.1+NT_HP_1.2+NT_HP_4.1+NT_HP_6.2+T _HP_7.1	1	0.3	1	0	1	0	1	0
NT_HP_1.1+NT_HP_1.3+NT_P_1.1+NT_P_1.3+NT_ P_12.2	1	0.3	1	0	1	0	1	0
NT_HP_1.1+NT_HP_2.3 +NT_HP_4.1+NT_HP_7.1 +NT_HP_9.1+NT_HP_12.1+NT_HP_12.2+NT_HP_1 2.5+NT_P_7.1	1	0.3	1	0	0	1	1	0
NT_HP_1.2+NT_HP_1.3+NT_HP_1.4+NT_HP_4.1+ NT_HP_6.2+NT_HP_9.1	1	0.3	1	0	1	0	0	1
NT_HP_1.2+NT_HP_1.3+NT_HP_2.7+NT_HP_4.1+ NT_HP_9.1+NT_HP_12.2	1	0.3	1	0	1	0	1	0
NT_HP_1.2+NT_HP_1.3+T_HP_2.2+NT_HP_6.2	2	0.61	2	0	2	0	2	0
NT_HP_1.2+NT_HP_4.1+NT_HP_6.1+NT_HP_8.1+ NT_HP_12.5	1	0.3	1	0	0	1	1	0
NT_HP_1.2+NT_HP_4.1+NT_HP_9.1+NT_HP_12.2+ NT_HP_13.2+NT_P_2.5+NT_P_4.1+NT_P_7.1+NT_ P_12.2	1	0.3	1	0	0	1	1	0
NT_HP_1.2+NT_HP_4.1+T_P_4.1 +NT_P_7.1+T_P_11.1	1	0.3	1	0	1	0	1	0
NT_HP_1.3+NT_HP_2.2+NT_HP_4.1+T_HP_12.1	1	0.3	1	0	0	1	1	0
NT_HP_1.3+NT_HP_2.7+NT_HP_3.2+NT_HP_12.5	1	0.3	1	0	0	1	1	0
NT_HP_1.3+NT_HP_2.7+NT_HP_3.2+NT_HP_4.1+ NT_HP_12.2+NT_HP_12.5	1	0.3	1	0	0	1	1	0

NT_HP_1.3+NT_HP_4.1+NT_HP_6.2	1	0.3	1	0	1	0	1	0
NT_HP_1.3+NT_HP_9.1	2	0.61	2	0	0	2	2	0
NT_HP_1.3+T_HP_2.2+NT_HP_6.2	1	0.3	1	0	0	1	1	0
NT_HP_1.4+NT_HP_3.2+NT_HP_4.1+NT_HP_6.2+ NT_P_4.1	1	0.3	1	0	1	0	1	0
NT_HP_1.4+NT_HP_4.1+NT_HP_6.1+NT_HP_12.2+ NT_P_1.1+NT_P_1.4+NT_P_4.1+NT_P_12.2	1	0.3	1	0	1	0	1	0
NT_HP_1.4+NT_HP_4.1+NT_HP_8.1	1	0.3	1	0	1	0	1	0
NT_HP_12.2+NT_HP_13.2	1	0.3	1	0	0	1	1	0
NT_HP_12.2+NT_P_4.1+NT_P_12.2	1	0.3	1	0	1	0	1	0
NT_HP_12.2+T_P_1.1+NT_P_3.2 +NT_P_4.1	1	0.3	1	0	1	0	1	0
NT_HP_13.2	1	0.3	1	0	1	0	1	0
NT_HP_2.1+NT_HP_12.2+T_C_2.2+NT_C_3.1+NT_ C_13.3	1	0.3	1	0	1	0	1	0
NT_HP_2.2+NT_HP_4.1	1	0.3	1	0	0	1	1	0
NT_HP_2.2+NT_HP_4.1+NT_HP_6.1+NT_HP_6.2+ NT_HP_7.1 +T_P_3.2+NT_P_4.1+NT_P_12.2	1	0.3	1	0	0	1	0	1
NT_HP_2.2+NT_HP_9.1+T_HP_13.2+NT_C_13.1+N T_P_13.1	1	0.3	1	0	0	1	1	0
NT_HP_2.4 +NT_HP_2.7+NT_HP_4.1+NT_HP_12.5+NT_P_1.2+ NT_P_1.3+NT_P_4.1+NT_P_12.2+NT_P_12.5	1	0.3	1	0	0	1	0	1
NT_HP_2.5+NT_HP_12.5+NT_P_4.1+NT_P_12.2+N T_P_12.5	1	0.3	1	0	0	1	1	0
NT_HP_2.5+NT_HP_7.1	1	0.3	0	1	1	0	1	0
NT_HP_2.7+NT_HP_12.2+NT_HP_12.5+NT_P_1.3+ NT_P_2.4+NT_P_4.1+NT_P_12.2	1	0.3	1	0	0	1	1	0
NT_HP_2.7+NT_HP_4.1+NT_HP_6.2+NT_HP_9.1	1	0.3	1	0	1	0	1	0
NT_HP_2.7+NT_HP_6.2	1	0.3	1	0	0	1	1	0
NT_HP_2.7+NT_HP_6.2+NT_HP_9.1	1	0.3	1	0	1	0	1	0
NT_HP_3.2+NT_HP_4.1+NT_HP_12.1+NT_P_1.3+N T_P_4.1+NT_P_12.2	1	0.3	1	0	0	1	1	0
NT_HP_3.2+NT_HP_4.1+NT_HP_7.1 +NT_HP_9.1+NT_P_12.2	1	0.3	1	0	1	0	1	0
NT_HP_3.2+NT_HP_4.1+NT_HP_9.1+NT_HP_12.2	1	0.3	1	0	0	1	1	0
NT_HP_3.2+NT_HP_4.1+NT_P_1.3+T_P_1.7+NT_P _2.4+NT_P_2.7+NT_P_4.2+NT_P_12.1+NT_P_12.5	1	0.3	1	0	0	1	1	0
NT_HP_3.2+NT_HP_7.1 +NT_HP_9.1+NT_HP_10.1+T_P_1.1+NT_P_1.7+NT _P_5.1	1	0.3	1	0	1	0	1	0
NT_HP_3.2+NT_P_2.5+NT_P_3.2 +NT_P_13.1	1	0.3	1	0	1	0	1	0
NT_HP_4.1	5	1.52	5	0	4	1	5	0
NT_HP_4.1+NT_HP_12.1	2	0.61	2	0	1	1	2	0
NT_HP_4.1+NT_HP_12.2	1	0.3	1	0	1	0	1	0
NT_HP_4.1+NT_HP_12.2+NT_HP_12.5+NT_C_1.2+ NT_C_4.1+NT_C_7.1+NT_C_12.2+NT_P_1.2+NT_P _4.1+NT_P_7.1+NT_P_12.2	1	0.3	1	0	1	0	0	1
NT_HP_4.1+NT_HP_12.2+NT_P_1.1+NT_P_1.2+NT _P_1.4+T_P_4.1 +NT_P_11.2+NT_P_12.2	1	0.3	1	0	0	1	0	1
NT_HP_4.1+NT_HP_12.2+NT_P_1.1+NT_P_1.4+NT _P_4.1+NT_P_12.2+NT_P_12.6	1	0.3	1	0	1	0	1	0

NT_HP_4.1+NT_HP_12.5+T_C_1.1+NT_C_1.2+NT_C_1.4+T_C_3.2	1	0.3	1	0	0	1	1	0
NT_HP_4.1+NT_HP_6.1	1	0.3	1	0	0	1	1	0
NT_HP_4.1+NT_HP_6.1+NT_P_1.3+NT_P_4.1	1	0.3	1	0	0	1	1	0
NT_HP_4.1+NT_HP_6.2	2	0.61	2	0	2	0	2	0
NT_HP_4.1+NT_HP_6.2+NT_HP_12.2	1	0.3	1	0	1	0	1	0
NT_HP_4.1+NT_HP_6.2+NT_HP_12.2+NT_P_4.1	1	0.3	1	0	0	1	1	0
NT_HP_4.1+NT_HP_6.2+NT_HP_7.1	1	0.3	1	0	1	0	1	0
NT_HP_4.1+NT_HP_6.2+NT_HP_7.1 +NT_HP_10.2+NT_HP_12.1+NT_HP_12.2	1	0.3	1	0	0	1	1	0
NT_HP_4.1+NT_HP_6.2+NT_HP_7.1 +NT_HP_12.2+NT_P_4.1+NT_P_12.5	1	0.3	1	0	1	0	1	0
NT_HP_4.1+NT_HP_6.2+NT_HP_7.1 +NT_HP_12.5	1	0.3	1	0	0	1	1	0
NT_HP_4.1+NT_HP_7.1	4	1.22	4	0	1	3	3	1
NT_HP_4.1+NT_HP_7.1 +NT_HP_12.1	1	0.3	1	0	0	1	1	0
NT_HP_4.1+NT_HP_7.1 +NT_HP_12.5	1	0.3	1	0	0	1	1	0
NT_HP_4.1+NT_HP_7.1 +NT_HP_12.5+NT_P_1.3+NT_P_1.4+NT_P_4.1+NT_P_7.1+NT_P_12.2	1	0.3	1	0	1	0	1	0
NT_HP_4.1+NT_HP_7.1 +NT_HP_9.1	1	0.3	1	0	0	1	1	0
NT_HP_4.1+NT_HP_7.1 +NT_HP_9.1+NT_P_1.3+NT_P_1.4+NT_P_2.4+NT_P_4.1+NT_P_12.2+NT_P_12.5	1	0.3	1	0	0	1	1	0
NT_HP_4.1+NT_HP_7.1 +T_HP_9.1	1	0.3	1	0	0	1	1	0
NT_HP_4.1+NT_HP_9.1	1	0.3	1	0	1	0	1	0
NT_HP_4.1+NT_HP_9.1+NT_HP_13.2	1	0.3	1	0	1	0	1	0
NT_HP_4.1+NT_P_1.2+NT_P_4.1+NT_P_12.2	1	0.3	1	0	1	0	1	0
NT_HP_4.1+NT_P_1.3+NT_P_4.1	1	0.3	1	0	1	0	1	0
NT_HP_4.1+NT_P_2.3+T_P_12.2+NT_P_13.1	1	0.3	1	0	1	0	1	0
NT_HP_4.1+NT_P_2.4+NT_P_4.1+NT_P_12.2+NT_P_12.5	1	0.3	1	0	1	0	1	0
NT_HP_4.1+T_P_1.1+NT_P_1.3+NT_P_4.1+NT_P_12.2	1	0.3	1	0	0	1	0	1
NT_HP_4.1+T_P_12.2+NT_P_13.1	1	0.3	1	0	1	0	1	0
NT_HP_4.1+T_P_3.2	1	0.3	1	0	1	0	1	0
NT_HP_7.1 +NT_C_13.1+NT_P_2.3+NT_P_12.2+NT_P_12.5	1	0.3	1	0	0	1	1	0
NT_HP_7.1 +NT_C_4.1+NT_C_7.1+NT_P_2.2+NT_P_2.3+NT_P_3.2 +NT_P_7.1+NT_P_12.2+NT_P_12.5	1	0.3	1	0	0	1	1	0
NT_HP_7.1 +NT_HP_12.1+NT_HP_12.5+NT_P_2.4+NT_P_4.1+NT_P_7.1+NT_P_12.5	1	0.3	1	0	1	0	1	0
NT_HP_7.1 +NT_HP_12.5+NT_P_1.2+NT_P_1.3+NT_P_2.4+NT_P_2.7+NT_P_4.1+NT_P_7.1+NT_P_12.2+NT_P_12.5	1	0.3	1	0	1	0	1	0
NT_HP_7.1 +NT_P_2.4+NT_P_3.2 +NT_P_4.1	1	0.3	1	0	0	1	1	0
NT_HP_8.1+NT_P_1.2+NT_P_1.3+NT_P_1.4+NT_P_4.1+NT_P_12.2+T_P_12.5	1	0.3	1	0	0	1	1	0

NT_HP_9.1	6	1.82	6	0	4	2	6	0
NT_HP_9.1+NT_HP_10.1	1	0.3	1	0	1	0	1	0
NT_HS_1.1+NT_HS_1.3+NT_HS_2.1+NT_HS_2.2+ NT_HS_2.7+NT_HS_4.1+NT_HS_12.5	1	0.3	1	0	0	1	1	0
NT_HS_1.1+NT_HS_2.1	1	0.3	1	0	0	1	1	0
NT_P_1.1+NT_P_1.2+NT_P_1.4+NT_P_2.2+NT_P_2 .3+NT_P_4.1+NT_P_12.2	1	0.3	1	0	1	0	1	0
NT_P_1.1+NT_P_1.4+NT_P_4.1+NT_P_12.2+T_P_1 2.5	1	0.3	1	0	1	0	1	0
NT_P_1.1+NT_P_1.4+NT_P_4.1+NT_P_6.1 +NT_P_12.2+NT_P_12.5+NT_P_12.6	1	0.3	1	0	0	1	0	1
NT_P_1.1+NT_P_1.5+NT_P_4.1+NT_P_5.1+NT_P_7 .1+NT_P_8.7+NT_P_12.1+T_P_12.2	1	0.3	1	0	1	0	0	1
NT_P_1.1+NT_P_2.2+NT_P_2.3+NT_P_4.1+NT_P_7 .1+NT_P_12.2+NT_P_12.5	1	0.3	1	0	1	0	1	0
NT_P_1.1+NT_P_2.2+NT_P_2.3+NT_P_4.1+NT_P_1 2.2	1	0.3	1	0	0	1	1	0
NT_P_1.1+NT_P_2.3+NT_P_2.7+NT_P_4.1+NT_P_1 2.5	1	0.3	1	0	0	1	1	0
NT_P_1.1+NT_P_2.3+NT_P_4.1+NT_P_7.1+NT_P_1 2.5+NT_P_12.6	1	0.3	1	0	0	1	1	0
NT_P_1.1+NT_P_2.3+NT_P_4.1+NT_P_7.1+NT_P_1 2.6	1	0.3	1	0	1	0	1	0
NT_P_1.1+NT_P_2.4+NT_P_2.7+NT_P_3.1+T_P_4.1	1	0.3	0	1	1	0	1	0
NT_P_1.1+NT_P_2.4+NT_P_4.1+NT_P_12.5	1	0.3	0	1	1	0	1	0
NT_P_1.1+NT_P_2.5+NT_P_4.1+NT_P_12.2	1	0.3	0	1	0	1	1	0
NT_P_1.1+NT_P_4.1+NT_P_12.2	1	0.3	0	1	1	0	1	0
NT_P_1.1+NT_P_4.1+NT_P_6.1 +NT_P_12.6	1	0.3	1	0	1	0	1	0
NT_P_1.1+T_P_1.4+NT_P_2.3+NT_P_2.7+NT_P_12. 1+T_P_12.6	1	0.3	0	1	0	1	1	0
NT_P_1.1+T_P_4.1	1	0.3	1	0	0	1	1	0
NT_P_1.2+NT_P_1.3+NT_P_1.4+NT_P_2.4+NT_P_2 .7+NT_P_4.1+NT_P_12.2+NT_P_12.5	1	0.3	1	0	0	1	1	0
NT_P_1.2+NT_P_1.3+NT_P_1.4+NT_P_2.4+NT_P_4 .1+NT_P_12.2	1	0.3	1	0	0	1	1	0
NT_P_1.2+NT_P_1.3+NT_P_1.4+NT_P_3.2 +NT_P_4.1+NT_P_12.2	1	0.3	1	0	1	0	1	0
NT_P_1.2+NT_P_1.3+NT_P_1.4+NT_P_4.1+NT_P_1 1.2+NT_P_12.2	1	0.3	1	0	0	1	0	1
NT_P_1.2+NT_P_1.3+NT_P_1.4+NT_P_4.1+NT_P_1 2.2	2	0.61	2	0	1	1	2	0
NT_P_1.2+NT_P_1.3+NT_P_1.4+NT_P_4.1+NT_P_7 .1+NT_P_12.2	1	0.3	1	0	1	0	1	0
NT_P_1.2+NT_P_1.3+NT_P_1.7+NT_P_4.1+NT_P_1 2.2	1	0.3	1	0	1	0	1	0
NT_P_1.2+NT_P_1.3+NT_P_2.4+NT_P_2.7+NT_P_4 .1+NT_P_12.2+NT_P_12.5	1	0.3	1	0	1	0	1	0
NT_P_1.2+NT_P_1.3+NT_P_2.4+NT_P_4.1+NT_P_1 1.2+NT_P_12.2	1	0.3	1	0	0	1	1	0
NT_P_1.2+NT_P_2.4+NT_P_4.1+NT_P_12.6	1	0.3	1	0	0	1	1	0
NT_P_1.2+NT_P_4.1+NT_P_12.2	1	0.3	1	0	1	0	1	0
NT_P_1.2+NT_P_4.1+NT_P_7.1+NT_P_12.5	1	0.3	1	0	1	0	1	0
NT_P_1.3+NT_P_1.4+NT_P_4.1+NT_P_12.2+NT_P_ 12.6	1	0.3	1	0	1	0	1	0

NT_P_1.3+NT_P_1.4+NT_P_4.1+NT_P_6.1 +NT_P_7.1+NT_P_12.2	1	0.3	1	0	0	1	1	0
NT_P_1.3+NT_P_2.1+NT_P_4.1	1	0.3	1	0	0	1	1	0
NT_P_1.3+NT_P_2.3+NT_P_4.1+NT_P_7.1	1	0.3	1	0	0	1	1	0
NT_P_1.3+NT_P_2.4+NT_P_2.7+NT_P_4.1+NT_P_12.2	1	0.3	1	0	1	0	1	0
NT_P_1.3+NT_P_2.4+NT_P_2.7+NT_P_4.1+T_P_8.1 +NT_P_12.2+NT_P_12.5+NT_P_12.6	1	0.3	1	0	0	1	1	0
NT_P_1.3+NT_P_2.4+NT_P_4.1+NT_P_10.1 +NT_P_12.2+NT_P_12.5	1	0.3	0	1	1	0	1	0
NT_P_1.3+NT_P_2.4+NT_P_4.1+NT_P_9.1+NT_P_10.1 +NT_P_12.2+NT_P_12.5	1	0.3	0	1	1	0	1	0
NT_P_1.3+NT_P_2.7+NT_P_3.2 +NT_P_7.1	1	0.3	1	0	1	0	1	0
NT_P_1.3+NT_P_4.1+NT_P_11.2+NT_P_12.2+NT_P_12.6	1	0.3	1	0	1	0	1	0
NT_P_1.4+T_P_2.3	1	0.3	0	1	1	0	1	0
NT_P_12.2	1	0.3	1	0	1	0	1	0
NT_P_13.1	7	2.13	7	0	6	1	5	2
NT_P_13.1+NT_P_14.1	1	0.3	1	0	1	0	1	0
NT_P_2.3	2	0.61	1	1	1	1	2	0
NT_P_2.3+NT_P_12.5	1	0.3	1	0	0	1	1	0
NT_P_2.4+NT_P_2.7+NT_P_4.1+NT_P_12.2+NT_P_12.5	1	0.3	1	0	0	1	1	0
NT_P_2.4+NT_P_4.1+NT_P_12.2	1	0.3	1	0	1	0	1	0
NT_P_2.4+T_P_3.2+NT_P_4.1+T_P_5.1	1	0.3	0	1	1	0	1	0
NT_P_2.5+NT_P_4.1	1	0.3	0	1	1	0	1	0
NT_P_2.5+T_P_5.1+NT_P_9.1+NT_P_13.1	1	0.3	1	0	1	0	1	0
NT_P_2.7+NT_P_4.1+NT_P_12.2	1	0.3	1	0	0	1	1	0
NT_P_2.7+NT_P_4.1+NT_P_12.5	1	0.3	1	0	0	1	1	0
NT_P_2.7+NT_P_7.1	1	0.3	1	0	1	0	1	0
NT_P_3.1+NT_P_4.1+NT_P_5.1+NT_P_7.1+NT_P_12.2	1	0.3	1	0	0	1	1	0
NT_P_3.1+NT_P_4.1+NT_P_6.1	1	0.3	1	0	0	1	1	0
NT_P_3.2 +NT_P_13.1	1	0.3	0	1	0	1	1	0
NT_P_3.2 +NT_P_5.1+NT_P_7.1	1	0.3	1	0	1	0	1	0
NT_P_4.1	7	2.13	7	0	5	2	7	0
NT_P_4.1+NT_P_12.2	5	1.52	4	0	4	0	4	0
NT_P_4.1+NT_P_12.2+NT_P_12.5	1	0.3	1	0	0	1	1	0
NT_P_4.1+NT_P_12.2+NT_P_13.1	1	0.3	1	0	1	0	1	0
NT_P_4.1+NT_P_12.2+T_P_12.5	1	0.3	1	0	1	0	1	0
NT_P_4.1+NT_P_12.5	2	0.61	2	0	1	1	1	1
NT_P_4.1+NT_P_5.1+NT_P_12.2	1	0.3	1	0	1	0	1	0
NT_P_4.1+NT_P_5.1+NT_P_13.1	1	0.3	1	0	1	0	1	0
NT_P_4.1+NT_P_6.1 +T_P_11.1 +NT_P_11.2+NT_P_12.2+NT_P_12.6	1	0.3	1	0	1	0	1	0

NT_P_4.1+NT_P_7.1	1	0.3	1	0	1	0	1	0
NT_P_4.1+NT_P_7.1+NT_P_12.1+NT_P_13.1	1	0.3	1	0	1	0	1	0
NT_P_4.1+NT_P_9.1	1	0.3	0	1	1	0	1	0
NT_P_5.1	1	0.3	1	0	1	0	1	0
NT_P_5.1+NT_P_11.1	1	0.3	1	0	1	0	1	0
NT_P_6.1 +NT_P_12.2+NT_P_13.1	1	0.3	1	0	1	0	1	0
T_C_3.3+NT_C_13.1+T_P_3.3+NT_P_13.1	1	0.3	1	0	0	1	1	0
T_HP_1.1+NT_HP_12.2+NT_P_13.1	1	0.3	1	0	0	1	1	0
T_HP_1.1+NT_HP_13.1	1	0.3	1	0	1	0	1	0
T_HP_1.1+NT_HP_2.4 +NT_HP_13.2+NT_P_1.1+NT_P_12.2	1	0.3	1	0	1	0	1	0
T_HP_1.3+T_HP_1.7+NT_HP_2.7+NT_HP_7.1 +NT_HP_13.2	1	0.3	1	0	1	0	1	0
T_HP_12.2	2	0.61	2	0	2	0	1	1
T_HP_2.7+NT_HP_3.2+NT_HP_6.2	1	0.3	1	0	0	1	0	1
T_HP_3.1+NT_HP_4.1	1	0.3	1	0	0	1	0	1
T_HP_7.1	1	0.3	1	0	1	0	0	1
T_HP_7.1+NT_P_2.4+T_P_3.2+NT_P_4.1	1	0.3	0	1	0	1	1	0
T_P_1.1+NT_P_1.2+NT_P_1.4+NT_P_1.5+NT_P_4.1 +NT_P_12.2	1	0.3	1	0	0	1	0	1
T_P_1.1+NT_P_2.4+T_P_3.2+NT_P_12.2+NT_P_12.5+NT_P_13.1	1	0.3	1	0	0	1	0	1
T_P_1.1+NT_P_3.2 +NT_P_12.2	1	0.3	1	0	0	1	1	0
T_P_1.3 +NT_P_2.7+NT_P_4.1+NT_P_12.1+NT_P_12.2	1	0.3	1	0	1	0	1	0
T_P_1.4+T_P_2.2+NT_P_2.3+NT_P_3.1+T_P_3.2+NT_P_12.5+NT_P_13.1	1	0.3	0	1	0	1	1	0
T_P_1.4+T_P_2.2+NT_P_2.3+NT_P_3.1+T_P_3.2+NT_P_13.1	1	0.3	0	1	0	1	1	0
T_P_2.1+T_P_3.2+NT_P_13.1	1	0.3	1	0	0	1	1	0
T_P_3.1+T_P_3.2+T_P_4.1 +NT_P_12.2	1	0.3	1	0	0	1	0	1
T_P_3.2+NT_P_4.1	1	0.3	1	0	1	0	1	0
T_P_3.2+NT_P_7.1+NT_P_13.1	1	0.3	0	1	0	1	1	0
T_P_4.1	1	0.3	1	0	1	0	1	0
T_P_4.1 +NT_P_6.1 +NT_P_12.2	1	0.3	1	0	1	0	1	0
UC	101	31	93	5	75	23	94	4
**Total**	**327**	**100**	**299**	**25**	**209**	**115**	**297**	**27**

Note:
KT Interventions in 15 studies assessing sustainability were:
case management, face-to-face/Internet-based self management, exercise program, Fluid Watchers LITE, Fluid Watchers PLUS, formal education, general health education, HF clinic follow-up, home visits, nurse management, patient education, peers for progress-diabetes, provider education, respiratory training, self-management telemedicine alerts

Appendix 12: Cochrane Effective Practice and Organisation of Care (EPOC) Risk of Bias Results

Study (Author,year)	1. Random sequence generation	2. Allocation concealment	3. Blinding of participants and personnel	4. Blinding of outcome assessment	5. Incomplete outcome data	6. Selective reporting	7. Other bias*
**Cluster RCTs**							
Baker, 2003[44]	Low risk	Low risk	Unclear risk	Low risk	Unclear risk	Unclear risk	Low risk
Beck, 1997[46]	Unclear risk	High risk	High risk	High risk	Low risk	Unclear risk	Unclear risk
Bruce, 2004[57]	Low risk	Unclear risk	Unclear risk	High risk	Low risk	High risk	Low risk
Bruce, 2015[58]	Unclear risk	Unclear risk	Unclear risk	Low risk	Low risk	Low risk	Low risk
Chen, 2015[36]	Low risk	Low risk	High risk	High risk	Low risk	High risk	Low risk
Cleveringa, 2008[66]	Unclear risk	Unclear risk	Low risk	Low risk	Low risk	High risk	High risk
Coleman, 1999[67]	Unclear risk	Unclear risk	Unclear risk	Low risk	Low risk	Unclear risk	Unclear risk
de Lusignana, 2013[70]	Low risk	Low risk	Low risk	Low risk	Low risk	High risk	Low risk
DeVore, 2015[73]	Unclear risk	Unclear risk	High risk	High risk	Low risk	High risk	Low risk
Dopp, 2015[74]	Low risk	Low risk	High risk	Low risk	Low risk	High risk	Low risk
Eccles, 2007[34]	Low risk	Low risk	High risk	High risk	Low risk	Low risk	Low risk
Eckert, 2010[76]	Unclear risk	Low risk	Unclear risk	Unclear risk	Unclear risk	Unclear risk	Unclear risk
Fihn, 2004[80]	Unclear risk	Low risk	Unclear risk	Low risk	High risk	Unclear risk	Unclear risk
Fihn, 2011[81]	Unclear risk	Low risk	Low risk	High risk	Low risk	Unclear risk	Low risk
Forster, 2015[83]	Low risk	Low risk	Low risk	High risk	Low risk	Low risk	Low risk
Freund, 2016[85]	Low risk	Low risk	High risk	Low risk	Low risk	High risk	Low risk
Goderis, 2010[27]	Low risk	Unclear risk	High risk	High risk	Low risk	Low risk	Low risk
Heisler, 2012[28]	Low risk	Unclear risk	High risk	Low risk	Low risk	Low risk	Low risk
Hetlevik, 2000[94]	Unclear risk	Unclear risk	Unclear risk	Unclear risk	High risk	Unclear risk	Unclear risk
Kennedy, 2013[37]	Low risk	Low risk	High risk	High risk	Low risk	Low risk	Low risk
Kennedy, 2015[105]	Low risk	Unclear risk	High risk	Low risk	Low risk	Unclear risk	Low risk
Kiefe, 2001[31]	Unclear risk	Unclear risk	High risk	High risk	Low risk	Unclear risk	Low risk

Study (Author,year)	1. Random sequence generation	2. Allocation concealment	3. Blinding of participants and personnel	4. Blinding of outcome assessment	5. Incomplete outcome data	6. Selective reporting	7. Other bias*
Kruis, 2014[109]	Low risk	Unclear risk	High risk	Unclear risk	Low risk	Low risk	Low risk
Krum, 2013[110]	Unclear risk	Unclear risk	High risk	Low risk	Low risk	Unclear risk	Low risk
Levine, 2011[115]	Unclear risk	Unclear risk	High risk	High risk	Low risk	Low risk	Low risk
Li, 2012[116]	Unclear risk	Unclear risk	Unclear risk	Low risk	Unclear risk	Unclear risk	Unclear risk
Lowrie, 2014[121]	Low risk	Low risk	Low risk	Low risk	Low risk	Low risk	Low risk
Machline-Carrion, 2019[122]	Low risk	Low risk	Unclear risk	Unclear risk	Low risk	Unclear risk	High risk
McCluskey, 2016[127]	Unclear risk	Low risk	High risk	Low risk	Low risk	Low risk	Low risk
Mitchell, 2005[133]	Low risk	Unclear risk	High risk	Unclear risk	High risk	Unclear risk	Low risk
Moher, 2001[134]	Low risk	Low risk	High risk	High risk	Low risk	Unclear risk	Low risk
Morganroth, 2016[135]	Unclear risk	Unclear risk	High risk	High risk	Low risk	Unclear risk	High risk
Murphy, 2009[137]	Low risk	Low risk	High risk	High risk	Low risk	Low risk	Low risk
Olaiya, 2017[35]	Low risk	Low risk	Low risk	Low risk	Low risk	High risk	Low risk
De Fine Olivarius, 2001[33]	Low risk	Unclear risk	High risk	High risk	Low risk	Unclear risk	Low risk
Pols, 2017[146]	Low risk	High risk	High risk	Low risk	Low risk	Low risk	Low risk
Rea, 2004[147]	Low risk	Unclear risk	High risk	Unclear risk	Unclear risk	Unclear risk	Low risk
Reiber, 2004[148]	Unclear risk	Unclear risk	High risk	Unclear risk	High risk	Unclear risk	Low risk
Sackley, 2015[152]	Low risk	Low risk	High risk	Low risk	Low risk	Low risk	Low risk
Salisbury, 2018[154]	Low risk	Low risk	High risk	Low risk	Low risk	Low risk	Low risk
Schafer, 2018[158]	Low risk	Low risk	High risk	High risk	Low risk	Low risk	Low risk
Shea, 2006[30]	Unclear risk	Unclear risk	Low risk	Low risk	Low risk	Low risk	Low risk
Sonnichsen, 2010[162]	Low risk	Unclear risk	High risk	Low risk	Low risk	High risk	Low risk
Tjia, 2015[32]	Unclear risk	Unclear risk	High risk	High risk	Unclear risk	Unclear risk	Low risk
Vaillant-Roussel, 2016[169]	Low risk	Unclear risk	High risk	High risk	Low risk	Low risk	Low risk

Study (Author,year)	1. Random sequence generation	2. Allocation concealment	3. Blinding of participants and personnel	4. Blinding of outcome assessment	5. Incomplete outcome data	6. Selective reporting	7. Other bias*
van de Ven, 2013[170]	Low risk	Low risk	High risk	High risk	Low risk	High risk	Low risk
Vickrey, 2006[26]	Low risk	Low risk	High risk	High risk	Low risk	Low risk	Low risk
Vinereanu, 2017[172]	Low risk	Low risk	High risk	High risk	Low risk	Unclear risk	Low risk
Walters, 2013[175]	Low risk	Low risk	High risk	Unclear risk	Low risk	High risk	Low risk
Whittle, 2014[177]	Low risk	Unclear risk	High risk	Unclear risk	Low risk	High risk	Low risk
Wilcock, 2013[178]	Low risk	Unclear risk	High risk	High risk	Low risk	High risk	Low risk
Zwar, 2016[182]	Low risk	Low risk	High risk	Low risk	Low risk	High risk	Low risk
**RCTs**							
Ansari, 2003[43]	Low risk	Unclear risk	High risk	Unclear risk	Low risk	Unclear risk	Unclear risk
Batchelor, 2012[45]	Low risk	Low risk	High risk	High risk	Low risk	Low risk	Low risk
Beck, 2017[46]	Low risk	Low risk	High risk	High risk	Low risk	Low risk	Low risk
Bekelman, 2015[48]	Low risk	Unclear risk	High risk	Unclear risk	Low risk	Low risk	Low risk
Benzo, 2016[49]	Low risk	Unclear risk	High risk	Low risk	Low risk	Low risk	Low risk
Blue, 2001[50]	Low risk	Unclear risk	High risk	Low risk	Low risk	Unclear risk	Low risk
Bohingamu, 2019[51]	Unclear risk	Unclear risk	Unclear risk	High risk	Low risk	Unclear risk	Low risk
Bohm, 2016[52]	Low risk	Low risk	High risk	Low risk	Low risk	Low risk	High risk
Team CPMMPE, 2007[53]	Low risk	Low risk	High risk	Low risk	Low risk	Unclear risk	Low risk
Bosanquet, 2017[54]	Low risk	High risk	High risk	High risk	Low risk	Low risk	Low risk
Bourbeau, 2003[55]	Low risk	Low risk	High risk	Low risk	Low risk	Unclear risk	Low risk
Boyne, 2012[56]	Low risk	Unclear risk	High risk	Low risk	Low risk	Low risk	Low risk
Bucknall, 2012[59]	Low risk	Low risk	Low risk	Low risk	Low risk	High risk	Low risk
Burns, 1995[60]	Low risk	Unclear risk	High risk	High risk	High risk	Unclear risk	Low risk
Burns, 2003[61]	Unclear risk	Unclear risk	Unclear risk	Low risk	High risk	High risk	Unclear risk
Callahan, 2006[62]	Low risk	Unclear risk	Unclear risk	Low risk	Low risk	Low risk	Low risk
Campbell, 1998[63]	Low risk	Unclear risk	High risk	Low risk	Low risk	Unclear risk	Unclear risk

Study (Author,year)	1. Random sequence generation	2. Allocation concealment	3. Blinding of participants and personnel	4. Blinding of outcome assessment	5. Incomplete outcome data	6. Selective reporting	7. Other bias*
Chi, 2012[64]	Unclear risk	Unclear risk	Unclear risk	Unclear risk	Unclear risk	Unclear risk	Unclear risk
Ciaschini, 2010[65]	Low risk	Unclear risk	High risk	High risk	Low risk	Low risk	High risk
Coull, 2004[68]	Low risk	Low risk	High risk	Low risk	Low risk	Unclear risk	Low risk
de la Porte, 2007[69]	Low risk	Unclear risk	High risk	High risk	Low risk	Unclear risk	Low risk
DeBusk, 2004[71]	Unclear risk	Low risk	High risk	Low risk	Low risk	Unclear risk	Low risk
Del Sindaco, 2007[72]	Unclear risk	Unclear risk	High risk	Low risk	Low risk	Unclear risk	Low risk
Dracup, 2014[42]	Low risk	Low risk	Low risk	Low risk	Low risk	Low risk	Low risk
Dunagan, 2005[75]	Unclear risk	Unclear risk	Unclear risk	Low risk	Low risk	Unclear risk	Low risk
Ell, 2007[77]	Unclear risk	Unclear risk	High risk	High risk	Low risk	Unclear risk	Low risk
Fan, 2012[78]	Low risk	Low risk	High risk	Low risk	Low risk	Low risk	High risk
Federman, 2019[79]	Unclear risk	Unclear risk	Low risk	Unclear risk	Low risk	Unclear risk	Low risk
Forster, 1996[82]	Low risk	Unclear risk	Unclear risk	Unclear risk	Low risk	Unclear risk	Low risk
Fortinsky, 2009[183]	Unclear risk	Unclear risk	High risk	High risk	High risk	Unclear risk	Unclear risk
Galbreath, 2004[86]	Low risk	Unclear risk	High risk	High risk	Low risk	Unclear risk	Low risk
Gallagher, 1997[87]	Unclear risk	Unclear risk	High risk	High risk	Unclear risk	Unclear risk	Low risk
Gaugler, 2008[88]	Low risk	Low risk	High risk	High risk	Low risk	Unclear risk	Low risk
Gellis, 2012[89]	Low risk	Low risk	High risk	Low risk	High risk	Unclear risk	Low risk
Graven, 2016[90]	Low risk	Low risk	High risk	Low risk	Low risk	Low risk	Low risk
Haerter, 2016[90]	Unclear risk	Unclear risk	High risk	Unclear risk	Low risk	Unclear risk	Unclear risk
Hendriks, 2012[92]	Low risk	Unclear risk	High risk	Low risk	Low risk	High risk	Low risk
Hernandez, 2015[93]	Low risk	Low risk	High risk	Low risk	Unclear risk	Unclear risk	Low risk
Hogg, 2009[95]	Low risk	Low risk	Unclear risk	High risk	Low risk	Unclear risk	Low risk
Holbrook, 2011[96]	Unclear risk	Low risk	High risk	Low risk	Low risk	High risk	Low risk
Holm, 2002[97]	Unclear risk	Unclear risk	High risk	Low risk	Low risk	Unclear risk	Low risk
Holt, 2010[98]	Low risk	Low risk	Low risk	Low risk	Low risk	Low risk	Unclear risk

Study (Author,year)	1. Random sequence generation	2. Allocation concealment	3. Blinding of participants and personnel	4. Blinding of outcome assessment	5. Incomplete outcome data	6. Selective reporting	7. Other bias*
Hughes, 2000[99]	Unclear risk	Unclear risk	Unclear risk	Unclear risk	High risk	Unclear risk	Unclear risk
Hunger, 2015[100]	Low risk	Low risk	Unclear risk	Low risk	Low risk	High risk	Low risk
Irewall, 2015[101]	Low risk	Unclear risk	High risk	High risk	Low risk	High risk	Low risk
Jaarsma, 2008[102]	Low risk	Unclear risk	Unclear risk	Low risk	Low risk	High risk	Low risk
Joling, 2012[103]	Unclear risk	Unclear risk	High risk	Low risk	Low risk	High risk	Low risk
Karhula, 2015[104]	Low risk	Low risk	High risk	High risk	Low risk	High risk	Unclear risk
Khdour, 2009[106]	Low risk	Unclear risk	High risk	High risk	High risk	Unclear risk	Low risk
Kim, 2014[107]	Unclear risk	Unclear risk	High risk	High risk	High risk	High risk	Low risk
Ko, 2017[108]	Low risk	Unclear risk	High risk	Low risk	Low risk	Low risk	Low risk
Kurz, 2010[111]	Unclear risk	Low risk	High risk	High risk	High risk	Unclear risk	Low risk
Kalter-Leibovici, 2017[112]	Low risk	Unclear risk	High risk	Low risk	Low risk	Low risk	Low risk
Leveille, 1998[113]	Low risk	Unclear risk	High risk	Unclear risk	Low risk	Unclear risk	Unclear risk
Leventhal, 2011[114]	Low risk	Low risk	High risk	Low risk	Low risk	Unclear risk	Low risk
Licskai, 2016[117]	Unclear risk	Unclear risk	Unclear risk	Unclear risk	High risk	Unclear risk	Unclear risk
Lin, 2018[118]	Low risk	Low risk	High risk	High risk	High risk	Unclear risk	Low risk
Liu, 2008[119]	Low risk	Unclear risk	High risk	High risk	Low risk	High risk	Low risk
Lopez Cabezas, 2006[120]	Low risk	Unclear risk	High risk	High risk	High risk	Unclear risk	Low risk
Mahoney , 2003[123]	Low risk	Unclear risk	High risk	Low risk	Low risk	High risk	Unclear risk
Maltais, 2008[124]	Low risk	Low risk	High risk	High risk	Low risk	Unclear risk	Low risk
Markle-Reid, 2011[125]	Low risk	Low risk	High risk	Low risk	Unclear risk	Low risk	Unclear risk
Markun, 2015[126]	Low risk	Low risk	High risk	High risk	Low risk	Low risk	Low risk
McElrath, 2017[128]	Low risk	Unclear risk	High risk	High risk	Low risk	Low risk	Low risk
McManus, 2014[129]	Unclear risk	Low risk	High risk	Low risk	Low risk	High risk	Low risk
Meeuwsen, 2012[130]	Unclear risk	Unclear risk	High risk	Low risk	Low risk	High risk	Low risk

Study (Author,year)	1. Random sequence generation	2. Allocation concealment	3. Blinding of participants and personnel	4. Blinding of outcome assessment	5. Incomplete outcome data	6. Selective reporting	7. Other bias*
Meisinger, 2013[131]	Low risk	Low risk	High risk	Low risk	Low risk	High risk	Low risk
Mejhert, 2004[132]	Unclear risk	Unclear risk	High risk	High risk	Low risk	Unclear risk	Low risk
Moriyama, 2009[136]	Unclear risk	Unclear risk	High risk	High risk	High risk	Unclear risk	Unclear risk
Moy, 2016[38]	Low risk	Unclear risk	High risk	High risk	Low risk	Low risk	Low risk
Nguyen, 2013[138]	Unclear risk	Unclear risk	Unclear risk	Unclear risk	High risk	High risk	Unclear risk
Ojeda, 2005[139]	Low risk	Unclear risk	High risk	High risk	Low risk	Unclear risk	Unclear risk
Olson, 2009[140]	Low risk	Unclear risk	High risk	High risk	Low risk	Unclear risk	Low risk
Ortiz-Bautista, 2017[141]	Unclear risk	Unclear risk	High risk	High risk	Low risk	Unclear risk	Unclear risk
Ostwald, 2014[142]	Low risk	Unclear risk	High risk	High risk	Low risk	Unclear risk	Low risk
Palacio, 2015[41]	Unclear risk	Unclear risk	High risk	High risk	Low risk	Low risk	Low risk
Peters-Klimm, 2010[143]	Low risk	Low risk	High risk	High risk	Low risk	High risk	Low risk
Piette, 2015[144]	Low risk	Low risk	High risk	High risk	Low risk	Low risk	Low risk
Pinnock, 2013[145]	Low risk	Low risk	High risk	Low risk	Low risk	High risk	Low risk
Rovner, 2020[149]	Low risk	Low risk	Low risk	High risk	Low risk	Unclear risk	Low risk
Rubenstein, 2007[150]	High risk	High risk	High risk	Low risk	Low risk	Unclear risk	Low risk
Saal, 2015[151]	Low risk	Low risk	High risk	High risk	Low risk	Low risk	High risk
Salinero-Fort, 2011[153]	Unclear risk	Unclear risk	High risk	High risk	Low risk	Low risk	Low risk
Samus, 2014[155]	Low risk	Unclear risk	High risk	High risk	Low risk	High risk	Low risk
Sanchez-nieto, 2016[156]	Low risk	Unclear risk	Low risk	Low risk	Low risk	Unclear risk	High risk
Sarkadi, 2004[157]	Low risk	Low risk	Low risk	Low risk	High risk	Unclear risk	Low risk
Schrader, 2005[159]	Unclear risk	Unclear risk	High risk	Unclear risk	High risk	Unclear risk	Unclear risk
Schraeder, 2009[29]	Unclear risk	Unclear risk	High risk	Unclear risk	Low risk	Unclear risk	Unclear risk
Smith, 1999[160]	Low risk	Unclear risk	High risk	High risk	High risk	Unclear risk	Unclear risk
Solomon, 2012[161]	Low risk	Low risk	High risk	Low risk	Low risk	Low risk	Low risk
Stewart, 2012[163]	Low risk	Unclear risk	High risk	Low risk	High risk	Low risk	Low risk

Study (Author,year)	1. Random sequence generation	2. Allocation concealment	3. Blinding of participants and personnel	4. Blinding of outcome assessment	5. Incomplete outcome data	6. Selective reporting	7. Other bias*
Stewart, 2015[164]	Low risk	Low risk	High risk	Low risk	Low risk	High risk	Low risk
Subramanian, 2004[165]	Low risk	Unclear risk	Unclear risk	Unclear risk	Unclear risk	Unclear risk	Low risk
Suominen, 2015[166]	Low risk	Low risk	High risk	High risk	Unclear risk	Low risk	Low risk
Tomita, 2009[167]	Unclear risk	Unclear risk	High risk	High risk	Unclear risk	Unclear risk	Unclear risk
Tremont, 2008[84]	Unclear risk	Unclear risk	High risk	Low risk	High risk	Unclear risk	Low risk
Trento, 2010[168]	Low risk	Unclear risk	High risk	High risk	Low risk	High risk	High risk
Trofimov, 2015[39]	Unclear risk	Unclear risk	High risk	Unclear risk	Low risk	Unclear risk	Unclear risk
Van Der Aa, 2015[171]	Low risk	Unclear risk	High risk	Low risk	Low risk	Low risk	Low risk
Wagenaar, 2019[173]	Low risk	Unclear risk	High risk	High risk	Low risk	Low risk	Low risk
Waldorff, 2012[174]	Low risk	Low risk	High risk	Low risk	Low risk	High risk	Low risk
Van Wetering, 2010[176]	Low risk	Low risk	High risk	High risk	Low risk	Low risk	Low risk
Wright, 2001[179]	Unclear risk	Unclear risk	High risk	High risk	High risk	Unclear risk	High risk
Xi, 2015[180]	Low risk	Unclear risk	High risk	Low risk	Low risk	Unclear risk	Low risk
Xiao, 2016[181]	Unclear risk	Unclear risk	High risk	High risk	High risk	Unclear risk	Low risk

* Other bias refers to other sources of bias.

High risk of bias: There is at least one important risk of bias, for example a study had a potential source of bias related to the specific study design used or has been claimed to have been fraudulent or had some other problem such as a drug manufacturer funded study or a funding agency/pharma company involved with data collection, analysis, or report writing.

Unclear risk of bias: There may be a risk of bias, but there is either insufficient information to assess whether an important risk of bias exists or insufficient rationale or evidence that an identified problem will introduce bias, such as the role of pharmaceutical company funding is not clarified, or conflicts of interest are not declared.

Low risk of bias - The study appears to be free of other sources of bias.

Appendix 13: Contour-Enhanced Funnel Plots

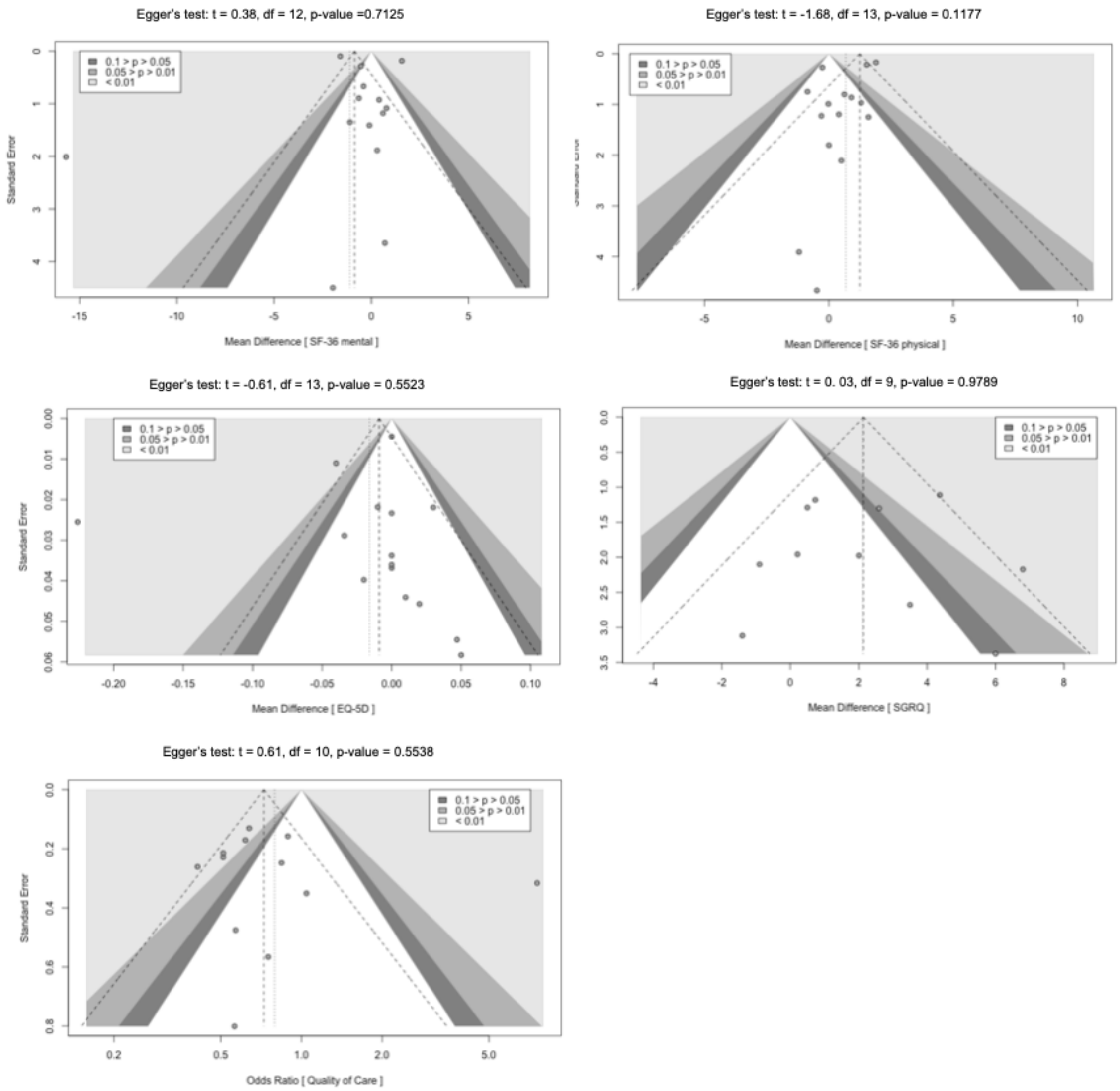

Appendix 14. Additional Analysis Results

Quality of Life

Short-Form (SF) Mental Health Survey

The 12 item Short-Form mental (SF-12 mental) was reported in five studies assessing six interventions plus usual care (Appendix 12). We performed a meta-analysis of four studies (2,978 participants) comparing any intervention vs usual care, which showed that KT interventions improved QOL compared to usual care, but with imprecise effect estimate (MD 1.27, 95% CI [-0.24, 2.78]; I^2=16%, τ=0.53; range of longer follow-up across studies 18-24 months) (Figure 4, Appendix 13). The combination of team changes, staff education, financial incentives, case management, promotion of self-management, and patient education interventions was associated with the largest effect compared with usual care, but evidence was from a single study (MD 2.00, 95% CI [0.95, 3.05]) [184].

For the outcome of SF-12 mental health survey, subgroup analysis showed there were no important differences between study settings or comorbidities, but a KT intervention effect was greater when more than one chronic disease was present (four chronic diseases: MD 2.00, 95% CI [0.95, 3.05], one RCT; one chronic disease: MD 0.63, 95% CI [−0.64, 1.90], 3 RCTs, I^2=0%, τ=0.00; Appendix 14). Additional analyses restricting to studies with history of prescription use, concomitant CDM therapy, and low ROB for incomplete outcome data and for selective outcome reporting also provided imprecise KT intervention effects for SF-12 mental (Appendix 15).

Short-Form (SF) Physical Health Survey

For SF-12 physical scale outcome, six studies were included that assessed seven interventions plus usual care. Our meta-analysis of five studies (2,581 participants, five interventions plus usual care) comparing any intervention vs usual care, showed that KT interventions improved QOL, with imprecise effect estimate (MD 3.72, 95% CI [-5.08, 12.52]; I^2=90%, τ=6.67; range of longer follow-up across

studies was 12-24 months; all studies were at low ROB for incomplete outcome data). Excluding an outlier [185], results suggested that KT interventions marginally improved QOL (MD 0.84, 95% CI [-0.02, 1.69]; I^2=16%, τ= 0.00).

For the outcome of the SF-12 physical scale, subgroup analysis showed no differences between study settings, number of chronic diseases or comorbidities (Appendix 13). Results of additional analyses restricting to studies with up to 80% male participants, history of prescription use, concomitant CDM therapy (allowed concomitant non-pharmacological therapies or procedures for all participants/arms during the study), and low ROB for selective outcome reporting, agreed with the SF-12 physical primary meta-analysis (Appendix 14). However, the KT intervention effect was greater when only men were included in a 12-month follow-up, yet this subgroup included only a single RCT [119] informed by 48 participants (MD 17.00, 95% CI [11.78, 22.22]).

Minnesota Living with Heart Failure Questionnaire (MLHFQ)

The MLHFQ scale was reported in eight studies of 10 interventions plus usual care. Meta-analysis of seven studies (1,580 participants) comparing any intervention vs usual care, showed that KT interventions improved QOL, but the effect was uncertain (MD -1.37, 95% CI [-7.39, 4.64]; I^2=82%, τ=5.82; range of longer follow-up across studies 12-25 months, single chronic disease across all studies; the proportion of male participants was less than 80% in all studies but one, which did not report this information).

Results were in agreement with primary meta-analysis when restricting to studies with reported study SD, low ROB due to attrition and selective reporting, history of prescription use, and concomitant CDM therapy. No major differences observed across subgroups of time in KT sustainability or a different number of comorbidities and settings (Appendices 14-15).

Appendix 15: Individual Study Results

Study	Treatment Comparison (EPOC) T1 VS T2	Treatment Comparison (BCT) T1 VS T2	Group 1: Mean/ SD/ total; Group 2: Mean/ SD/ total [continuous data]	Group 1: Events per total (n/N); Group 2: Events per total (n/N) [binary data]	MD (95% CI)	OR (95% CI)
**Quality of life: 12 item Short-Form Mental Health Survey**						
Dunagan 2005 [75]	relay+case-M+Self-M+px_educ VS relay+px_educ	NT_P_4.1+NT_P_5.1+NT_P_12.2 VS NT_P_4.1+NT_P_5.1+NT_P_13.1	Group 1: 6.3/10/76 Group 2: 7.6/12.3/75	N/A	-1.30 (-4.88 to 2.28)	N/A
Freund-2016 [85]	team+staff+finan+case-M+Self-M+px_educ VS UC	T_HP_1.1+NT_HP_2.4 +NT_HP_13.2+NT_P_1.1 +NT_P_12.2 VS UC	Group 1: 48.9/10.8/934 Group 2: 46.9/11.6/840	N/A	2.00 (0.95 to 3.05)	N/A
Bosanquet-2017[54]	team+EPR+relay+case-M+Self-M VS UC	NT_HP_2.4 +NT_HP_2.7 +NT_HP_4.1 +NT_HP_12.5+NT_P_1.2 +NT_P_1.3+NT_P_4.1+NT_P_12.2+NT_P_12.5 VS UC	Group 1: 40.1/11.34/249 Group 2: 38.9/10.84/236	N/A	1.20 (-0.77 to 3.17)	N/A
Murphy-2009[137]	staff+Self-M VS UC	NT_HP_1.4+NT_HP_4.1+NT_HP_6.1+NT_HP_12.2 +NT_P_1.1+NT_P_1.4+NT_P_4.1+NT_P_12.2 VS UC	Group 1: 49.6/10.9/189 Group 2: 48.9/11.7/195	N/A	0.70 (-1.56 to 2.96)	N/A
Stewart-2015[164]	case-M+Self-M+px_educ VS UC	NT_P_1.2+NT_P_1.3+NT_P_1.4+NT_P_4.1+NT_P_12.2 VS UC	Group 1: 48.4/11.3/168 Group 2: 48.7/11.3/167	N/A	-0.30 (-2.72 to 2.12)	N/A

**Quality of life: 36 item Short-Form Mental Health Survey**						
Hogg-2009[95]	team+case-M VS UC	T_P_1.1+NT_P_3.2+NT_P_12.2 VS UC	N/A	N/A	1.10 (-1.55 to 3.75)	N/A
Kalter-Leibovici-2017[112]	team+relay+case-M+Self-M+px_educ VS UC	T_HP_7.1+NT_P_2.4+T_P_3.2+NT_P_4.1 VS UC	N/A	N/A	-1.57 (-1.93 to -1.21)	N/A
Karhula(a)-2015[104]	team+EPR+relay+case-M+Self-M+px_educ+mot_intervie VS UC	T_P_1.1+NT_P_2.4+T_P_3.2+NT_P_12.2+NT_P_12.5+NT_P_13.1 VS UC	Group 1: -0.05/9.22/190 Group 2: 0.55/8.65/79	N/A	-0.60 (-2.91 to 1.71)	N/A
Karhula(b)-2015[104]	team+EPR+relay+case-M+Self-M+px_educ+mot_interview VS UC	T_P_1.1+NT_P_2.4+T_P_3.2+NT_P_12.2+NT_P_12.5+NT_P_13.1 VS UC	Group 1: 1.06/9.16/180 Group 2: 1.84/7.04/70	N/A	-0.78 (-2.90 to 1.34)	N/A
Nguyen-2013[138]	team+case-M+Self-M+px_reminder+px_educ VS team+case-M+Self-M+px_educ+mot_interview VS px_educ	T_P_1.4+T_P_2.2+NT_P_2.3+NT_P_3.1+T_P_3.2+NT_P_12.5+NT_P_13.1 VS T_P_1.4+T_P_2.2+NT_P_2.3+NT_P_3.1+T_P_3.2+NT_P_13.1 VS NT_P_3.2+NT_P_13.1	Group 1: 52.2/11.71/43 Group 2: 50.6/12.25/41 Group 3: 52.1/11.76/41	N/A	0.10 (-4.92 to 5.12) (group 1 vs group 3) -1.50 (-6.70 to 3.70) (group 2 vs group 3) -1.60 (-6.73 to 3.53) (group 1 vs group 2)	N/A
Fihn-2004[80]	AF+staff VS UC	NT_HP_4.1+NT_HP_6.2+NT_HP_7.1 VS UC	N/A	N/A	0.53 (-0.03 to 1.09)	N/A
Markle-Reid-2011[125]	team+staff+case-M+Self-M+px_educ+px_finan VS UC	NT_P_1.1+NT_P_1.4+NT_P_4.1+NT_P_12.2+T_P_12.5 VS	Group 1: 69.55/22.12/43 Group 2: 67.57/18.58/39	N/A	1.98 (-6.84 to 10.80)	N/A

		UC				
Peters-Klimm-2010[143]	team+EPR+relay+AF+case-M+Self-M+px_educ VS UC	NT_HP_2.7+NT_HP_12.2+NT_HP_12.5+NT_P_1.3+NT_P_2.4+NT_P_4.1+NT_P_12.2 VS UC	Group 1: 46.6/9.9/97 Group 2: 46.5/9.9/100	N/A	0.10 (-2.67 to 2.87)	N/A
Subramanian-2004[165]	staff+clin_reminder VS UC	NT_HP_4.1+NT_HP_7.1+T_HP_9.1 VS UC	Group 1: 3.7/1.3/355 Group 2: 2.1/1.3/365	N/A	1.60 (1.41 to 1.79)	N/A
Xiao-2016[181]	staff+case-M+Self-M VS UC	NT_C_1.2+NT_C_1.3+NT_C_2.4+NT_C_2.7+NT_C_4.1+NT_C_12.2 VS UC	Group 1: 38.7/7/31 Group 2: 23/8.6/30	N/A	15.70 (11.76 to 19.64)	N/A
Kruis-2014[109]	AF+staff+Self-M+mot_interview VS UC	NT_HP_1.3+NT_HP_4.1+NT_HP_6.2 VS UC	Group 1: 0.73/9.67/240 Group 2: 0.09/9.77/231	N/A	0.64 (-1.12 to 2.40)	N/A
Vaillant-Roussel-2016[169]	staff+case-M+Self-M+px_educ+mot_interview VS UC	NT_HP_4.1+NT_HP_6.1+NT_P_1.3+NT_P_4.1 VS UC	Group 1: 58/22.1/76 Group 2: 58.7/23.9/83	N/A	-0.70 (-7.85 to 6.45)	N/A
Walters-2013[175]	staff+case-M+Self-M+px_educ VS UC	NT_HP_4.1+NT_HP_12.2+NT_P_1.1+NT_P_1.2+NT_P_1.4+T_P_4.1+NT_P_11.2+NT_P_12.2 VS UC	Group 1: 50.2/11.4/67 Group 2: 50.5/10.5/68	N/A	-0.30 (-4.00 to 3.40)	N/A
Galbreath-2004[86]	team+relay+staff+case-M+Self-M+px_educ VS team+staff+case-M+Self-M+px_educ	NT_P_2.4+NT_P_2.7+NT_P_4.1+NT_P_12.2+NT_P_12.5 VS	Group 1: 53.2/10.5/354 Group 2: 53.8/11/356 Group 3: 53.1/10.1/359	N/A	0.10 (-1.41 to 1.61) (group 1 vs group 3) 0.70 (-0.85 to 2.25) (group 2 vs group 3)	N/A

	VS<br>UC	NT_P_4.1+NT_P_12.2+NT_P_12.5<br>VS<br>UC			0.60 (-0.98 to 2.18)<br>(group 1 vs group 2)	
Eccles-2007[34]	EPR+staff+clin_reminder+px_reminder<br>VS<br>UC	NT_HP_4.1+NT_HP_7.1+NT_HP_12.1<br>VS<br>UC	Group 1:<br>45.8/12.1/311<br>Group 2:<br>46.2/11.8/360	N/A	-0.40 (-2.22 to 1.42)	N/A
**Quality of life: 12 item Short-Form Physical Health Survey**						
Dunagan-2005[75]	relay+case-M+Self-M+px_educ<br>VS<br>relay+px_educ	NT_P_4.1+NT_P_5.1+NT_P_12.2<br>VS<br>NT_P_4.1+NT_P_5.1+NT_P_13.1	Group 1:<br>-1.2/13/60<br>Group 2:<br>-2.5/12/61	N/A	1.30 (-3.16 to 5.76)	N/A
Freund-2016[85]	team+staff+finan+case-M+Self-M+px_educ<br>VS<br>UC	T_HP_1.1+NT_HP_2.4+NT_HP_13.2+NT_P_1.1+NT_P_12.2<br>VS<br>UC	Group 1:<br>36.5/10.6/777<br>Group 2:<br>35.5/10.2/711	N/A	1.00 (-0.06 to 2.06)	N/A
Bosanquet-2017[54]	team+EPR+relay+case-M+Self-M<br>VS<br>UC	NT_HP_2.4+NT_HP_2.7+NT_HP_4.1+NT_HP_12.5+NT_P_1.2+NT_P_1.3+NT_P_4.1+NT_P_12.2+NT_P_12.5<br>VS<br>UC	Group 1:<br>34/13.51/249<br>Group 2:<br>35.1/12.11/236	N/A	-1.10 (-3.38 to 1.18)	N/A
Murphy-2009[137]	staff+Self-M<br>VS<br>UC	NT_HP_1.4+NT_HP_4.1+NT_HP_6.1+NT_HP_12.2+NT_P_1.1+NT_P_1.4+NT_P_4.1+NT_P_12.2<br>VS<br>UC	Group 1:<br>40.5/11.1/159<br>Group 2:<br>38.8/11.1/173	N/A	1.70 (-0.69 to 4.09)	N/A
Stewart-2015[164]	case-M+Self-M+px_educ<br>VS<br>UC	NT_P_1.2+NT_P_1.3+NT_P_1.4+NT_P_4.1+NT_P_12.2<br>VS<br>UC	Group 1:<br>38.1/10.8/116<br>Group 2:<br>36.6/12.1/112	N/A	1.50 (-1.48 to 4.48)	N/A

Liu-2008[119]	relay+Self-M+px_reminder+px_educ VS UC	NT_P_1.1+NT_P_2.3+NT_P_4.1+NT_P_7.1+NT_P_12.5+NT_P_12.6 VS UC	Group 1: 47.9/7.35/24 Group 2: 30.9/10.78/24	N/A	17 (11.78 to 22.22)	N/A
**Quality of life: 36 item Short-Form Physical Health Survey**						
Hogg-2009[95]	team+case-M VS UC	T_P_1.1+NT_P_3.2+NT_P_12.2 VS UC	N/A	N/A	-1.60 (-4.05 to 0.85)	N/A
Kalter-Leibovici-2017[112]	team+relay+case-M+Self-M+px_educ VS UC	T_HP_7.1+NT_P_2.4+T_P_3.2+NT_P_4.1 VS UC	N/A	N/A	-1.53 (-1.95 to -1.11)	N/A
Karhula-2015a[104]	team+EPR+relay+case-M+Self-M+px_educ+mot_intervie VS UC	T_P_1.1+NT_P_2.4+T_P_3.2+NT_P_12.2+NT_P_12.5+NT_P_13.1 VS UC	Group 1: 1.25/6.27/162 Group 2: 0.39/4.65/68	N/A	0.86 (-0.61 to 2.33)	N/A
Karhula-2015b[104]	team+EPR+relay+case-M+Self-M+px_educ+mot_interview VS UC	T_P_1.1+NT_P_2.4+T_P_3.2+NT_P_12.2+NT_P_12.5+NT_P_13.1 VS UC	Group 1: 0.53/5.76/146 Group 2: 0.51/6.43/55	N/A	0.02 (-1.92 to 1.96)	N/A
Nguyen-2013[138]	team+case-M+Self-M+px_reminder+px_educ VS team+case-M+Self-M+px_educ+mot_interview VS px_educ	T_P_1.4+T_P_2.2+NT_P_2.3+NT_P_3.1+T_P_3.2+NT_P_12.5+NT_P_13.1 VS T_P_1.4+T_P_2.2+NT_P_2.3+NT_P_3.1+T_P_3.2+NT_P_13.1 VS NT_P_3.2+NT_P_13.1	Group 1: 38.9/11.21/43 Group 2: 35.8/11.6/41 Group 3: 35.2/11.27/41	N/A	3.70 (-1.11 to 8.51) (group 1 vs group 3) 0.60 (-4.35 to 5.55) (group 2 vs group 3) -3.10 (-7.98 to 1.78) (group 1 vs group 2)	N/A
Fihn-2004[80]	AF+staff VS UC	NT_HP_4.1+NT_HP_6.2+NT_HP_7.1	N/A	N/A	0.26 (-0.27 to 0.79)	N/A

		VS UC				
Markle-Reid-2011[125]v	team+staff+case-M+Self-M+px_educ+px_finan VS UC	NT_P_1.1+NT_P_1.4+NT_P_4.1+NT_P_12.2+T_P_12.5 VS UC	Group 1: 48.29/23.23/43 Group 2: 47.81/18.96/39	N/A	0.48 (-8.66 to 9.62)	N/A
Peters-Klimm-2010[143]	team+EPR+relay+AF+case-M+Self-M+px_educ VS UC	NT_HP_2.7+NT_HP_12.2+NT_HP_12.5+NT_P_1.3+NT_P_2.4+NT_P_4.1+NT_P_12.2 VS UC	Group 1: 38.3/8.6/97 Group 2: 38/8.6/100	N/A	0.30 (-2.10 to 2.70)	N/A
Schrader-2005[159]	team+staff+case-M+Self-M+px_educ VS UC	NT_HP_4.1+NT_HP_12.2 VS UC	Group 1: 36.7/7.35/213 Group 2: 37.6/10.78/237	N/A	-0.90 (-2.59 to 0.79)	N/A
Galbreath-2004[86]	team+relay+staff+case-M+Self-M+px_educ VS UC	NT_P_2.4+NT_P_2.7+NT_P_4.1+NT_P_12.2+NT_P_12.5 VS UC	Group 1: 32.1/12.4/354 Group 2: 33.4/13.4/359	N/A	-1.30 (-3.19 to 0.59)	N/A
Subramanian-2004[165]	staff+clin_reminder VS UC	NT_HP_4.1+NT_HP_7.1+T_HP_9.1 VS UC	Group 1: -0.6/2/269 Group 2: 1.3/2/280	N/A	-1.90 (-2.23 to -1.57)	N/A
Xiao-2016[181]	staff+case-M+Self-M VS UC	NT_C_1.2+NT_C_1.3+NT_C_2.4+NT_C_2.7+NT_C_4.1+NT_C_12.2 VS UC	Group 1: 41.1/7.7/31 Group 2: 41.6/8.7/30	N/A	-0.50 (-4.63 to 3.63)	N/A
Kruis-2014[109]	AF+staff+Self-M+mot_interview VS UC	NT_HP_1.3+NT_HP_4.1+NT_HP_6.2 VS UC	Group 1: -1.1/8.65/240 Group 2: -0.48/8.77/231	N/A	-0.62 (-2.19 to 0.95)	N/A
Vaillant-Roussel-2016[169]	staff+case-M+Self-M+px_educ+mot_interview VS	NT_HP_4.1+NT_HP_6.1+NT_P_1.3+NT_P_4.1 VS UC	Group 1: 52.8/23.8/76 Group 2: 51.6/25.5/83	N/A	1.20 (-6.46 to 8.86)	N/A

	UC					
Walters-2013[175]	staff+case-M+Self-M+px_educ VS UC	NT_HP_4.1+NT_HP_12.2+NT_P_1.1+NT_P_1.2+NT_P_1.4+T_P_4.1+NT_P_11.2+NT_P_12.2 VS UC	Group 1: 38.5/10.3/58 Group 2: 38.5/9.4/62	N/A	0.00 (-3.54 to 3.54)	N/A
Eccles-2007[34]	EPR+staff+clin_reminder+px_reminder VS UC	NT_HP_4.1+NT_HP_7.1+NT_HP_12.1 VS UC	Group 1: 29.7/15.6/311 Group 2: 30.1/15.3/360	N/A	-0.40 (-2.75 to 1.95)	N/A
**Quality of life: European Quality of Life-5 Dimensions (EQ-5D)**						
Team CPMMPE-2007[53]	team+px_educ VS UC	T_HP_12.2 VS UC	Group 1: 0.58/0.07/810 Group 2: 0.58/0.07/422	N/A	0.00 (-0.01 to 0.01)	N/A
Freund-2016[85]	team+staff+finan+case-M+Self-M+px_educ VS UC	T_HP_1.1+NT_HP_2.4+NT_HP_13.2+NT_P_1.1+NT_P_12.2 VS UC	Group 1: 0.65/0.22/864 Group 2: 0.61/0.23/806	N/A	0.04 (0.02 to 0.06)	N/A
Kennedy-2013[37]	staff+Self-M VS UC	T_HP_3.1+NT_HP_4.1 VS UC	Group 1: 0.6/0.3/277 Group 2: 0.6/0.3/409	N/A	0.00 (-0.05 to 0.05)	N/A
van de Ven-2013[170]	Staff VS UC	NT_HP_1.4+NT_HP_4.1+NT_HP_8.1 VS UC	Group 1: 0.35/0.26/49 Group 2: 0.36/0.22/79	N/A	-0.01 (-0.10 to 0.08)	N/A
Forster-2015[83]	team+case-M+Self-M+px_educ VS UC	NT_P_1.2+NT_P_1.3+NT_P_1.4+NT_P_4.1+NT_P_7.1+NT_P_12.2 VS UC	Group 1: 0.51/0.46/134 Group 2: 0.56/0.48/128	N/A	-0.05 (-0.16 to 0.06)	N/A
Sackley-2015[152]	staff+case-M+Self-M+px_educ VS UC	NT_HP_4.1 VS UC	Group 1: 0.2/0.36/160 Group 2: 0.18/0.31/123	N/A	0.02 (-0.06 to 0.10)	N/A
Schafer-2018[158]	staff+Self-M+px_educ VS	NT_HP_4.1 VS UC	Group 1: 0.68/0.32/86 Group 2:	N/A	-0.02 (-0.11 to 0.07)	N/A

	UC		0.7/0.28/87			
Van Der Aa-2015[171]	case-M+Self-M+px_educ VS UC	NT_P_1.2+NT_P_1.3+NT_P_1.4+NT_P_4.1+NT_P_11.2+NT_P_12.2 VS UC	Group 1: 0.7/0.3/131 Group 2: 0.7/0.3/134	N/A	0.00 (-0.07 to 0.07)	N/A
Stewart-2015[164]	case-M+Self-M+px_educ VS UC	NT_P_1.2+NT_P_1.3+NT_P_1.4+NT_P_4.1+NT_P_12.2 VS UC	Group 1: 0.68/0.25/116 Group 2: 0.68/0.26/112	N/A	0.00 (-0.07 to 0.07)	N/A
Beck-2017[47]	team+case-M+px_finan VS UC	NT_P_4.1+NT_P_12.2 VS UC	Group 1: 0/0.25/97 Group 2: 0/0.25/98	N/A	0.00 (-0.07 to 0.07)	N/A
Kruis-2014[109]	AF+staff+Self-M+mot_interview VS UC	NT_HP_1.3+NT_HP_4.1+NT_HP_6.2 VS UC	Group 1: -0.04/0.24/240 Group 2: -0.01/0.24/231	N/A	-0.03 (-0.07 to 0.01)	N/A
Stewart-2012[163]	team+case-M VS UC	NT_P_12.2 VS UC	Group 1: -0.136/0.363/87 Group 2: -0.183/0.35/84	N/A	-0.05 (-0.15 to 0.06)	N/A
McManus-2014[129]	relay+Self-M+px_educ VS UC	NT_P_1.1+NT_P_2.4+NT_P_4.1+NT_P_12.5 VS UC	Group 1: 0.816/0.254/276 Group 2: 0.806/0.258/276	N/A	0.01 (-0.03 to 0.05)	N/A
Lin-2018[118]	team+case-M+px_educ VS UC	NT_HP_12.2+NT_P_4.1+NT_P_12.2 VS UC	Group 1: 0.216/0.16/87 Group 2: -0.01/0.18/91	N/A	0.23 (0.18 to 0.28)	N/A
Lopez Cabezas-2006[120]	case-M+px_educ VS UC	NT_C_1.2+NT_C_4.1+NT_C_12.2+NT_P_1.2+NT_P_4.1+NT_P_12.2 VS UC	Group 1: 0.64/0.154/70 Group 2: 0.606/0.178/64	N/A	0.03 (-0.02 to 0.09)	N/A
**Quality of life: St George's Respiratory Questionnaire**						

Ko-2017[108]	case-M+Self-M+px_educ VS UC	NT_P_1.3+NT_P_4.1+NT_P_11.2+NT_P_12.2+NT_P_12.6 VS UC	Group 1: -6.9/15.3/90 Group 2: -0.1/13.8/90	N/A	-6.80 (-11.06 to -2.54)	N/A
Bourbeau-2003[55]	case-M+Self-M+px_educ VS UC	NT_P_1.4+T_P_2.3 VS UC	Group 1: -3.5/14.99/96 Group 2: -1.5/12.18/95	N/A	-2.00 (-5.87 to 1.87)	N/A
Bucknall-2012[59]	case-M+Self-M VS UC	NT_P_1.1+NT_P_2.4+NT_P_2.7+NT_P_3.1+T_P_4.1 VS UC	Group 1: -2.99/12.56/232 Group 2: 1.38/11.33/232	N/A	-4.37 (-6.55 to -2.19)	N/A
Fan-2012[78]	case-M+Self-M VS UC	NT_P_1.1+NT_P_2.4+NT_P_2.7+NT_P_3.1+T_P_4.1 VS UC	Group 1: 51.3/13.3/209 Group 2: 51.8/13.3/217	N/A	-0.50 (-3.03 to 2.03)	N/A
Hernandez 2015[93]	case-M+Self-M VS UC	NT_P_1.1+NT_P_2.4+NT_P_2.7+NT_P_3.1+T_P_4.1 VS UC	Group 1: 43/20/71 Group 2: 49/22/84	N/A	-6.00 (-12.62 to 0.62)	N/A
Khdour-2009[106]	case-M+Self-M VS UC	NT_P_1.3+NT_P_1.4+NT_P_4.1+NT_P_6.1+NT_P_7.1+NT_P_12.2 VS UC	Group 1: 61.8/16.55/86 Group 2: 65.3/18.61/87	N/A	-3.50 (-8.75 to 1.75)	N/A
Pinnock-2013[145]	relay+Self-M+px_educ VS UC	NT_HP_7.1+NT_P_2.4+NT_P_3.2+NT_P_4.1 VS UC	Group 1: 68.2/16.3/128 Group 2: 67.3/17.3/128	N/A	0.90 (-3.22 to 5.02)	N/A
Xi-2015[180]	Self-M+px_educ VS px_educ	NT_P_2.5+NT_P_4.1 VS NT_P_13.1	Group 1: 59.6/5.4/30 Group 2: 63.66/3.6/30	N/A	-4.06 (-6.38 to -1.74)	N/A
Van Wetering-2010[176]	team+Self-M+px_educ+mot_interview VS	NT_P_3.1+NT_P_4.1+NT_P_5.1+NT_P_7.1+NT_P_12.2	N/A	N/A	-2.60 (-5.15 to -0.05)	N/A

	UC	VS UC				
Zwar-2016[182]	EPR+staff VS UC	NT_HP_1.3+ NT_HP_2.2+ NT_HP_4.1+ T_HP_12.1 VS UC	Group 1: 16.85/15.2/136 Group 2: 17.06/14.9/104	N/A	-0.21 (-4.05 to 3.63)	N/A
Kruis-2014[109]	AF+staff+Self-M+mot_interview VS UC	NT_HP_1.3+ NT_HP_4.1+ NT_HP_6.2 VS UC	Group 1: -0.4/12.67/241 Group 2: 0.33/13.00/232	N/A	-0.73 (-3.04 to 1.58)	N/A
Walters-2013[175]	staff+case-M+Self-M+px_educ VS UC	NT_HP_4.1+ NT_HP_12.2 +NT_P_1.1+ NT_P_1.2+NT_P_1.4+T_P_4.1+NT_P_11.2+ NT_P_12.2 VS UC	Group 1: 41.9/18.9/67 Group 2: 40.5/17.4/69	N/A	1.40 (-4.71 to 7.51)	N/A
**Quality of life: Minnesota Living with Heart Failure Questionnaire**						
Chi-2012[64]	Self-M VS UC	NT_P_2.3 VS UC	Group 1: 21.11/18.3/84 Group 2: 34.53/14.85/87	N/A	-13.42 (-18.43 to -8.41)	N/A
Ortiz-Bautista-2017[141]	team+case-M+px_educ VS UC	NT_P_4.1+NT_P_12.2 VS UC	Group 1: 23.46/22.1/84 Group 2: 19.8/23.3/43	N/A	3.66 (-4.76 to 12.08)	N/A
Piette-2015[144]	relay+Self-M+px_reminder+px_educ VS relay+Self-M+px_educ	NT_HP_7.1+ NT_C_4.1+NT_C_7.1+NT_P_2.2+NT_P_2.3+NT_P_3.2+NT_P_7.1+NT_P_12.2+NT_P_12.5 VS NT_HP_7.1+ NT_C_13.1+ NT_P_2.3+NT_P_12.2+NT_P_12.5	N/A	N/A	-0.74 (-5.43 to 3.95)	N/A
Vaillant-Roussel-2016[169]	case-M+Self-M VS UC	NT_HP_4.1+ NT_HP_6.1+ NT_P_1.3+NT_P_4.1 VS UC	Group 1: 33.4/22.1/76 Group 2: 27.2/23.3/83	N/A	6.20 (-0.86 to 13.26)	N/A

Stewart-2012[163]	team+case-M VS UC	NT_P_12.2 VS UC	Group 1: -9.75/11.68/143 Group 2: -8/15.84/137	N/A	1.75 -1.52 to 5.02)	N/A
de la Porte-2007[69]	team+case-M+Self-M+px_educ VS UC	NT_P_1.1+NT_P_2.5+NT_P_4.1+NT_P_12.2 VS UC	Group 1: 30.2/22.1/118 Group 2: 34.5/23.3/122	N/A	-4.30 (-10.04 to 1.44)	N/A
Ojeda-2005[139]	Self-M+px_educ VS UC	NT_P_1.1+NT_P_4.1+NT_P_12.2 VS UC	Group 1: 35.7/18.9/76 Group 2: 37.8/21/77	N/A	-2.10 (-8.43 to 4.23)	N/A
Wagenaar-2019[173]	EPR+relay+case-M+px_reminder+px_educ VS Self-M+px_reminder VS UC	NT_HP_7.1+NT_HP_12.5+NT_P_1.2+NT_P_1.3+NT_P_2.4+NT_P_2.7+NT_P_4.1+NT_P_7.1+NT_P_12.2+NT_P_12.5 VS NT_P_1.2+NT_P_4.1+NT_P_7.1+NT_P_12.5 VS UC	Group 1: 25.5/27.8/150 Group 2: 28.3/31/150 Group 3: 26.5/32.5/150	N/A	-1.00 (-7.84 to 5.84) (group 1 vs group 3) 1.80 (-5.39 to 8.99) (group 2 vs group 3) 2.80 (-3.86 to 9.46) (group 1 vs group 2)	N/A
**Quality of Care**						
Baker-2003[44]	AF+staff VS staff	NT_HP_1.3+T_HP_2.2+NT_HP_6.2 VS NT_HP_1.3+NT_HP_9.1	N/A	Group 1: 151/176 Group 2: 298/361	N/A	0.78 (0.47 to 1.30)
Kiefe-2001[31]	cQI+AF VS UC	NT_HP_1.2+NT_HP_1.3+T_HP_2.2+NT_HP_6.2 VS UC	N/A	Group 1: 162/279 Group 2: 129/280	N/A	0.62 (0.44 to 0.86)
Levine-2011[115]	staff+clin_reminder VS UC	NT_HP_4.1+NT_HP_7.1 VS UC	N/A	Group 1: 547/640 Group 2: 508/605	N/A	0.89 (0.65 to 1.21)
Vinereanu-2017[172]	EPR+AF+staff+px_educ VS UC	NT_HP_2.2+NT_HP_9.1+T_HP_13.2+NT_C_13.1+NT_P_13.1	N/A	Group 1: 171/214 Group 2: 132/197	N/A	0.51 (0.33 to 0.80)

		VS<br>UC				
Ansari-2003[43]	staff+clin_reminder+px_reminder<br>VS<br>team+staff+case-M<br>VS<br>staff	NT_HP_1.1+<br>NT_HP_2.3+<br>NT_HP_4.1+<br>NT_HP_7.1+<br>NT_HP_9.1+<br>NT_HP_12.1<br>+NT_HP_12.2+NT_HP_12.5+NT_P_7.1<br>VS<br>NT_HP_3.2+<br>NT_HP_4.1+<br>NT_HP_9.1+<br>NT_HP_4.1+<br>NT_HP_9.1+<br>NT_HP_12.2<br>VS<br>NT_HP_4.1+<br>NT_HP_7.1	N/A	Group 1:<br>1/64<br>Group 2:<br>23/54<br>Group 3:<br>5/51	N/A	6.85 (0.77 to 60.61)<br>(group 1 vs group 3)<br>0.15 (0.05 to 0.43)<br>(group 2 vs group 3)<br>46.74 (6.03 to 362.29))<br>(group 1 vs group 2)
Galbreath-2004[86]	team+staff+case-M+Self-M+px_educ<br>VS<br>UC	NT_P_2.5+NT_P_4.1+NT_P_9.1+NT_P_12.2+NT_P_12.5<br>VS<br>UC	N/A	Group 1:<br>386/710<br>Group 2:<br>155/359	N/A	0.64 (0.49 to 0.82)
Lowrie-2014[121]	team+staff<br>VS<br>UC	NT_HP_1.2+<br>NT_HP_1.3+<br>NT_HP_2.7+<br>NT_HP_4.1+<br>NT_HP_9.1+<br>NT_HP_12.2<br>VS<br>UC	N/A	Group 1:<br>40/43<br>Group 2:<br>30/34	N/A	0.56 (0.12 to 2.70)
Morganroth-2016[135]	EPR+AF+staff+clin_reminder<br>VS<br>UC	NT_HP_4.1+<br>NT_HP_6.2+<br>NT_HP_7.1+<br>NT_HP_12.5<br>VS<br>UC	N/A	Group 1:<br>33/43<br>Group 2:<br>30/46	N/A	0.57 (0.22 to 1.44)
Zwar-2016[182]	EPR+staff<br>VS<br>UC	NT_HP_1.3+<br>NT_HP_2.2+<br>NT_HP_4.1+<br>T_HP_12.1<br>VS<br>UC	N/A	Group 1:<br>50/132<br>Group 2:<br>83/101	N/A	7.56 (4.07 to 14.05)
Mitchell-2005[133]	AF+staff<br>VS<br>UC	NT_HP_4.1+<br>NT_HP_6.2<br>VS<br>UC	N/A	Group 1:<br>77/380<br>Group 2:<br>27/153	N/A	0.84 (0.52 to 1.37)

Sonnichsen-2010[162]	staff+case-M+Self-M+px_educ VS UC	NT_HP_4.1 VS UC	N/A	Group 1: 10/23 Group 2: 11/30	N/A	0.75 (0.25 to 2.28)
Beck-1997[46]	team+case-M+px_educ VS UC	NT_P_4.1+NT_P_12.2 VS UC	N/A	Group 1: 130/160 Group 2: 103/161	N/A	0.41 (0.25 to 0.68)
Eccles-2007[34]	EPR+staff+clin_reminder+px_reminder VS UC	NT_HP_4.1+NT_HP_7.1+NT_HP_12.1 VS UC	N/A	Group 1: 171/216 Group 2: 165/250	N/A	0.51 (0.34 to 0.78)
Hetlevik-2000[94]	EPR+staff+clin_reminder+px_educ VS UC	NT_HP_4.1+NT_HP_7.1+NT_HP_12.5 VS UC	N/A	Group 1: 19/104 Group 2: 21/111	N/A	1.04 (0.52 to 2.08)

Appendix 16: Meta-analysis Results of All Interventions vs Usual Care

Outcome	No of studies (No of patients)	MD/OR (95% CI) [fixed-effect model]	MD/OR (95% CI) [random-effects model]	95% PI	τ (95% CI)	I^2 (95% CI)	Q (d.f.); p-value
Quality of life: 12 item Short-Form Mental Health Survey	4 (2,978)	MD: 1.45 (0.64 to 2.25)	MD: 1.27 (-0.24 to 2.78)	-1.94 to 4.48	0.53 (0.00 to 3.45)	16% (0% to 87%)	3.55 (3); 0.31
Quality of life: 36 item Short-Form Mental Health Survey	14 (5,876)	MD: 0.86 (0.70 to 1.02)	MD: 1.11 (-1.25 to 3.47)	-7.08 to 9.30	3.61 (2.42 to 6.59)	96% (94% to 97%)	297.38 (13); <0.0001
Quality of life: 12 item Short-Form Physical Health Survey	5 (2,581)	MD: 1.26 (0.42 to 2.10)	MD: 3.72 (-5.08 to 12.52)	-19.63 to 27.07	6.67 3.68 to 20.96)	90% (79% to 95%)	39.44 (4); <0.0001
Quality of life: 36 item Short-Form Physical Health Survey	15 (5,678)	MD: -1.24 (-1.46 to -1.02)	MD: -0.68 (-1.20 to -0.15)	-2.55 to 1.98	0.81 (0.31 to 1.05)	77% (63% to 86%)	61.05 (14); <0.0001
Quality of life: European Quality of Life-5 Dimensions (EQ-5D)	15 (6,628)	MD: 0.01 (0.00 to 0.02)	MD: 0.02 (-0.02 to 0.05)	-0.12 to 0.15	0.06 (0.04 to 0.10)	85% (76% to 90%)	91.38 (14); <0.0001
Quality of life: St George's Respiratory Questionnaire	11 (2,893)	MD: -2.14 (-3.12 to -1.16)	MD: -2.12 (-3.72 to -0.51)	-5.76 to 1.52	1.45 (0.00 to 4.10)	44% (0% to 72%)	17.73 (10.00); 0.06
Quality of life: Minnesota Living with Heart Failure Questionnaire	7 (1,580)	MD: -1.48 (-3.48 to -0.52)	MD: -1.37 (-7.39 to 4.64)	-17.65 to 14.91	5.82 (2.98 to 13.88)	82% (63% to 91%)	32.90 (6); <0.0001
Quality of care	12 (5,271)	OR: 1.38 (1.21 to 1.57)	OR: 1.26 (0.77 to 2.05)	0.24 to 6.49	0.70 (0.44 to 1.25)	84% (74% to 91%)	70.28 (11); <0.0001

Appendix 17: Subgroup Analyses of All KT Interventions vs Usual Care

KT setting

Study	Total.KT	Mean.KT	SD.KT	Total.UC	Mean.UC	SD.UC	MD	95%−CI	Weight (common)	Weight (random)
KT_setting = Not reported										
Freund−2016	934	48.90	10.80	840	46.90	11.60	2.00	[0.95; 3.05]	59.4%	47.5%
KT_setting = Home										
Bosanquet−2017	249	40.10	11.34	236	38.90	10.84	1.20	[−0.77; 3.17]	16.7%	20.8%
KT_setting = GP clinic										
Murphy−2009	189	49.60	10.90	195	48.90	11.70	0.70	[−1.56; 2.96]	12.7%	16.7%
KT_setting = Home + outpatient clinic										
Stewart−2015	168	48.40	11.30	167	48.70	11.30	−0.30	[−2.72; 2.12]	11.1%	14.9%
**Common effect model**							**1.45**	**[0.64; 2.25]**	**100.0%**	**−−**
**Random effects model**							**1.27**	**[−0.24; 2.78]**	**−−**	**100.0%**
**Prediction interval**								**[−1.93; 4.48]**		

Heterogeneity: $I^2 = 16\%$, $\tau^2 = 0.2846$, $p = 0.31$
Test for subgroup differences (fixed effect): $c^2_3 = 3.55$, df = 3 ($p = 0.31$)
Test for subgroup differences (random effects): $c^2_3 = 3.55$, df = 3 ($p = 0.31$)

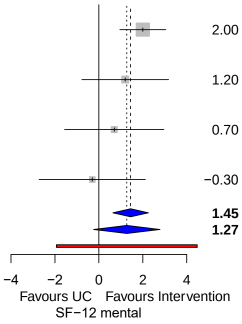

Study	Total.KT	Mean.KT	SD.KT	Total.UC	Mean.UC	SD.UC	MD	95%−CI	Weight (common)	Weight (random)
KT_setting = Home										
Galbreath−2004	710	53.50	10.75	359	53.10	10.10	0.40	[−0.91; 1.71]	1.4%	8.2%
Hogg−2009	120			121			1.10	[−1.55; 3.75]	0.3%	7.4%
Markle−Reid−2011	43	69.55	22.12	39	67.57	18.58	1.98	[−6.84; 10.80]	0.0%	3.3%
Xiao−2016	31	38.70	7.00	30	23.00	8.60	15.70	[11.76; 19.64]	0.2%	6.4%
Leibovici−2017	682			678			−1.57	[−1.93; −1.21]	19.1%	8.4%
Common effect model							−1.26	[−1.60; −0.92]	21.0%	−−
Random effects model							3.44	[−5.26; 12.13]	−−	33.8%
Heterogeneity: $I^2 = 95\%$, $\tau^2 = 45.9591$, $p < 0.01$										
KT_setting = Not reported										
Peters−Klimm−2010	97	46.60	9.90	100	46.50	9.90	0.10	[−2.67; 2.87]	0.3%	7.3%
Karhula(a)−2015	190	−0.05	9.22	79	0.55	8.65	−0.60	[−2.91; 1.71]	0.5%	7.6%
Karhula(b)−2015	180	1.06	9.16	70	1.84	7.04	−0.78	[−2.90; 1.34]	0.5%	7.7%
Common effect model							−0.50	[−1.87; 0.86]	1.3%	−−
Random effects model							−0.50	[−1.57; 0.56]	−−	22.7%
Heterogeneity: $I^2 = 0\%$, $\tau^2 = 0$, $p = 0.88$										
KT_setting = GP clinic										
Fihn−2004	123			68			0.53	[−0.03; 1.09]	7.8%	8.4%
Vaillant−Roussel−2016	76	58.00	22.10	83	58.70	23.90	−0.70	[−7.85; 6.45]	0.0%	4.2%
Common effect model							0.52	[−0.04; 1.08]	7.9%	−−
Random effects model							0.52	[−0.69; 1.74]	−−	12.6%
Heterogeneity: $I^2 = 0\%$, $\tau^2 = 0$, $p = 0.74$										
KT_setting = Primary care clinic										
Subramanian−2004	355	3.70	1.30	365	2.10	1.30	1.60	[1.41; 1.79]	68.1%	8.4%
Eccles−2007	311	45.80	12.10	360	46.20	11.80	−0.40	[−2.22; 1.42]	0.7%	7.9%
Kruis−2016	240	0.73	9.67	231	0.09	9.77	0.64	[−1.12; 2.40]	0.8%	8.0%
Common effect model							1.57	[1.38; 1.76]	69.6%	−−
Random effects model							0.89	[−1.61; 3.38]	−−	24.3%
Heterogeneity: $I^2 = 65\%$, $\tau^2 = 0.7231$, $p = 0.06$										
KT_setting = Community										
Walters−2013	67	50.20	11.40	68	50.50	10.50	−0.30	[−4.00; 3.40]	0.2%	6.6%
**Common effect model**							**0.86**	**[0.70; 1.02]**	**100.0%**	**−−**
**Random effects model**							**1.11**	**[−1.25; 3.47]**	**−−**	**100.0%**
**Prediction interval**								**[−7.08; 9.30]**		

Heterogeneity: $I^2 = 96\%$, $\tau^2 = 13.0188$, $p < 0.01$
Test for subgroup differences (fixed effect): $c^2_4 = 207.61$, df = 4 ($p < 0.01$)
Test for subgroup differences (random effects): $c^2_4 = 16.71$, df = 4 ($p < 0.01$)

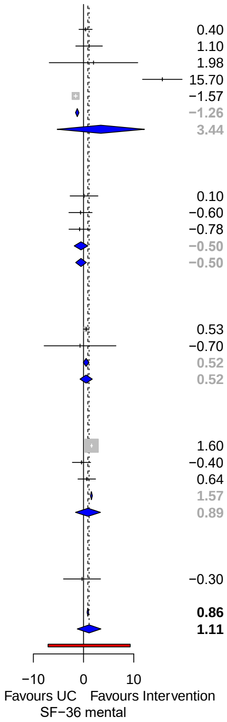

Study	Total.KT	Mean.KT	SD.KT	Total.UC	Mean.UC	SD.UC	MD	95%−CI	Weight (common)	Weight (random)
KT_setting = Not reported										
Freund−2016	777	36.50	10.60	711	35.50	10.20	1.00	[−0.06; 2.06]	63.4%	20.9%
KT_setting = Home										
Bosanquet−2017	249	34.00	13.51	236	35.10	12.11	−1.10	[−3.38; 1.18]	13.6%	20.5%
KT_setting = GP clinic										
Murphy−2009	159	40.50	11.10	173	38.80	11.10	1.70	[−0.69; 4.09]	12.4%	20.4%
KT_setting = Home + outpatient clinic										
Liu−2008	24	47.90	7.35	24	30.90	10.78	17.00	[11.78; 22.22]	2.6%	18.2%
Stewart−2015	116	38.10	10.80	112	36.60	12.10	1.50	[−1.48; 4.48]	8.0%	20.0%
Common effect model							5.31	[2.72; 7.90]	10.6%	−−
Random effects model							9.10	[−89.36; 107.55]	−−	38.2%
Heterogeneity: $I^2 = 96\%$, $\tau^2 = 115.4236$, $p < 0.01$										
**Common effect model**							**1.26**	**[0.42; 2.10]**	**100.0%**	**−−**
**Random effects model**							**3.72**	**[−5.08; 12.52]**	**−−**	**100.0%**
**Prediction interval**								**[−19.63; 27.07]**		

Heterogeneity: $I^2 = 90\%$, $\tau^2 = 44.4548$, $p < 0.01$
Test for subgroup differences (fixed effect): $\chi^2_3 = 13.89$, df = 3 ($p < 0.01$)
Test for subgroup differences (random effects): $\chi^2_3 = 4.49$, df = 3 ($p = 0.21$)

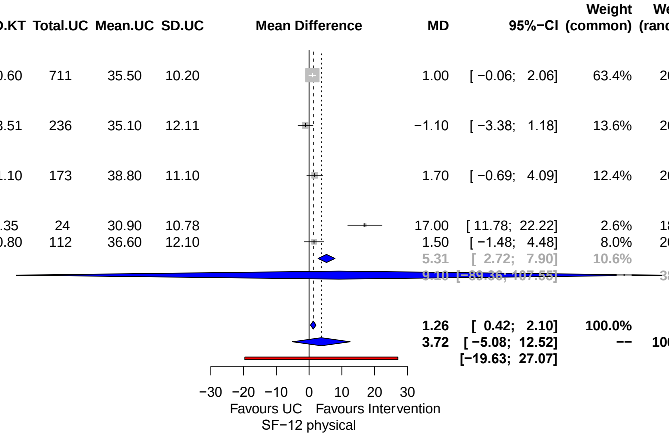

Study	Total.KT	Mean.KT	SD.KT	Total.UC	Mean.UC	SD.UC	MD	95%−CI	Weight (common)	Weight (random)
KT_setting = Home										
Galbreath−2004	354	32.10	12.40	359	33.40	13.40	−1.30	[−3.19; 0.59]	1.4%	6.4%
Hogg−2009	109			114			−1.60	[−4.05; 0.85]	0.8%	4.6%
Markle−Reid−2011	43	48.29	23.23	39	47.81	18.96	0.48	[−8.66; 9.62]	0.1%	0.5%
Xiao−2016	31	41.10	7.70	30	41.60	8.70	−0.50	[−4.63; 3.63]	0.3%	2.0%
Leibovici−2017	682			678			−1.53	[−1.95; −1.11]	27.2%	14.6%
Common effect model							−1.51	[−1.91; −1.10]	29.7%	−−
Random effects model							−1.51	[−1.71; −1.31]	−−	28.1%
Heterogeneity: $I^2 = 0\%$, $\tau^2 = 0$, $p = 0.98$										
KT_setting = Not reported										
Peters−Klimm−2010	97	38.30	8.60	100	38.00	8.60	0.30	[−2.10; 2.70]	0.8%	4.7%
Karhula−2015a	162	1.25	6.27	68	0.39	4.65	0.86	[−0.61; 2.33]	2.3%	8.4%
Karhula−2015b	146	0.53	5.76	55	0.51	6.43	0.02	[−1.92; 1.96]	1.3%	6.3%
Common effect model							0.51	[−0.55; 1.56]	4.4%	−−
Random effects model							0.51	[−0.64; 1.65]	−−	19.4%
Heterogeneity: $I^2 = 0\%$, $\tau^2 = 0$, $p = 0.78$										
KT_setting = Primary care clinic										
Fihn−2004	123			68			0.26	[−0.27; 0.79]	17.3%	14.1%
Subramanian−2004	269	−0.60	2.00	280	1.30	2.00	−1.90	[−2.23; −1.57]	43.5%	15.0%
Eccles−2007	311	29.70	15.60	360	30.10	15.30	−0.40	[−2.75; 1.95]	0.9%	4.9%
Kruis−2016	240	−1.10	8.65	231	−0.48	8.77	−0.62	[−2.19; 0.95]	2.0%	7.9%
Common effect model							−1.25	[−1.53; −0.97]	63.7%	−−
Random effects model							−0.73	[−2.36; 0.89]	−−	41.9%
Heterogeneity: $I^2 = 94\%$, $\tau^2 = 1.0196$, $p < 0.01$										
KT_setting = GP clinic										
Schrader−2005	213	36.70	7.35	237	37.60	10.78	−0.90	[−2.59; 0.79]	1.7%	7.3%
Vaillant−Roussel−2016	76	52.80	23.80	83	51.60	25.50	1.20	[−6.46; 8.86]	0.1%	0.6%
Common effect model							−0.80	[−2.45; 0.85]	1.8%	−−
Random effects model							−0.80	[−6.41; 4.81]	−−	8.0%
Heterogeneity: $I^2 = 0\%$, $\tau^2 = 0$, $p = 0.60$										
KT_setting = Community										
Walters−2013	58	38.50	10.30	62	38.50	9.40	−0.00	[−3.54; 3.54]	0.4%	2.6%
**Common effect model**							**−1.24**	**[−1.46; −1.02]**	**100.0%**	**−−**
**Random effects model**							**−0.68**	**[−1.20; −0.15]**	**−−**	**100.0%**
**Prediction interval**								**[−2.55; 1.20]**		

Heterogeneity: $I^2 = 77\%$, $\tau^2 = 0.6505$, $p < 0.01$
Test for subgroup differences (fixed effect): $\chi^2_4 = 13.02$, df = 4 ($p = 0.01$)
Test for subgroup differences (random effects): $\chi^2_4 = 57.02$, df = 4 ($p < 0.01$)

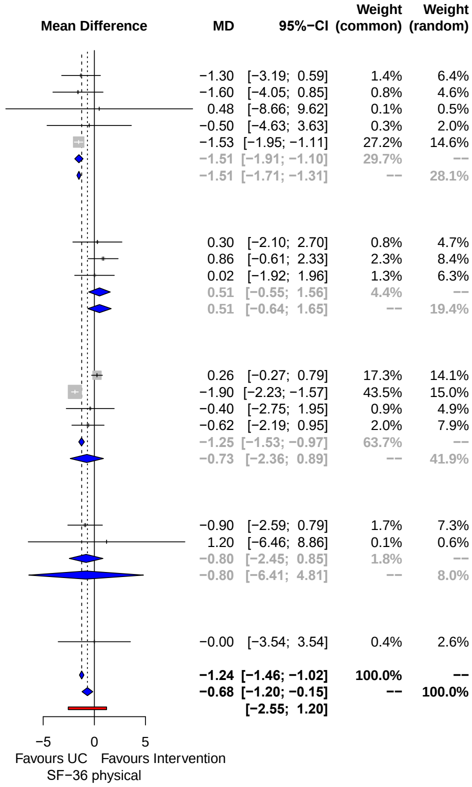

Study	Total.KT	Mean.KT	SD.KT	Total.UC	Mean.UC	SD.UC	MD	95%−CI	Weight (common)	Weight (random)
KT_setting = Community										
Bond−2007	810	0.58	0.07	422	0.58	0.07	−0.00	[−0.01; 0.01]	69.9%	8.7%
Forster−2015	134	0.51	0.46	128	0.56	0.48	−0.05	[−0.16; 0.06]	0.4%	4.4%
Common effect model							−0.00	[−0.01; 0.01]	70.3%	−−
Random effects model							−0.00	[−0.05; 0.05]	−−	13.1%
Heterogeneity: $I^2 = 0\%$, $\tau^2 = 0$, $p = 0.39$										
KT_setting = Not reported										
van de Ven−2013	49	0.35	0.26	79	0.36	0.22	−0.01	[−0.10; 0.08]	0.7%	5.6%
Freund−2016	864	0.65	0.22	806	0.61	0.23	0.04	[0.02; 0.06]	11.5%	8.4%
Lin−2018	87	0.22	0.16	91	−0.01	0.18	0.23	[0.18; 0.28]	2.1%	7.4%
Common effect model							0.07	[0.05; 0.08]	14.3%	−−
Random effects model							0.09	[−0.22; 0.40]	−−	21.4%
Heterogeneity: $I^2 = 96\%$, $\tau^2 = 0.0144$, $p < 0.01$										
KT_setting = Primary care clinic										
Kennedy−2013	277	0.60	0.30	409	0.60	0.30	−0.00	[−0.05; 0.05]	2.6%	7.6%
Kruis−2016	240	−0.04	0.24	231	−0.01	0.24	−0.03	[−0.07; 0.01]	2.9%	7.7%
Schafer−2017	86	0.68	0.32	87	0.70	0.28	−0.02	[−0.11; 0.07]	0.7%	5.5%
Common effect model							−0.02	[−0.05; 0.01]	6.1%	−−
Random effects model							−0.02	[−0.06; 0.03]	−−	20.7%
Heterogeneity: $I^2 = 0\%$, $\tau^2 = 0$, $p = 0.64$										
KT_setting = Care homes										
Sackley−2015	160	0.20	0.36	123	0.18	0.31	0.02	[−0.06; 0.10]	0.9%	6.0%
KT_setting = Rehab or home										
Van Der Aa−2015	131	0.70	0.30	134	0.70	0.30	−0.00	[−0.07; 0.07]	1.0%	6.3%
KT_setting = Home										
McManus−2014	276	0.82	0.25	276	0.81	0.26	0.01	[−0.03; 0.05]	2.9%	7.7%
Beck−2017	97	0.00	0.25	98	0.00	0.25	−0.00	[−0.07; 0.07]	1.1%	6.4%
Common effect model							0.01	[−0.03; 0.04]	4.0%	−−
Random effects model							0.01	[−0.05; 0.06]	−−	14.1%
Heterogeneity: $I^2 = 0\%$, $\tau^2 = 0$, $p = 0.81$										
KT_setting = Home + outpatient clinic										
Lopez Cabezas−2006	70	0.64	0.15	64	0.61	0.18	0.03	[−0.02; 0.09]	1.7%	7.1%
Stewart−2015	116	0.68	0.25	112	0.68	0.26	−0.00	[−0.07; 0.07]	1.2%	6.6%
Common effect model							0.02	[−0.02; 0.06]	2.9%	−−
Random effects model							0.02	[−0.19; 0.23]	−−	13.6%
Heterogeneity: $I^2 = 0\%$, $\tau^2 = 0$, $p = 0.44$										
KT_setting = Disease/HF/COPD/HF clinic										
Stewart−2012	84	−0.18	0.35	87	−0.14	0.36	−0.05	[−0.15; 0.06]	0.5%	4.7%
**Common effect model**							**0.01**	**[0.00; 0.02]**	**100.0%**	**−−**
**Random effects model**							**0.02**	**[−0.02; 0.05]**	**−−**	**100.0%**
**Prediction interval**								**[−0.12; 0.15]**		

Heterogeneity: $I^2 = 85\%$, $\tau^2 = 0.0035$, $p < 0.01$
Test for subgroup differences (fixed effect): $\chi^2_7 = 41.23$, df = 7 ($p < 0.01$)
Test for subgroup differences (random effects): $\chi^2_7 = 8.71$, df = 7 ($p = 0.27$)

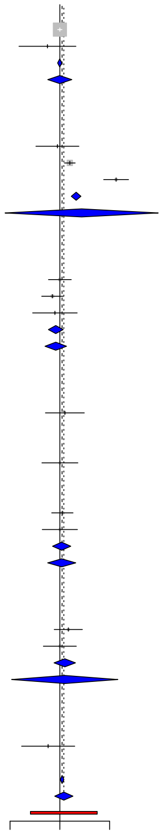

Study	Total.KT	Mean.KT	SD.KT	Total.UC	Mean.UC	SD.UC	MD	95%−CI	Weight (common)	Weight (random)
KT_setting = Home										
Bourbeau−2003	96	−3.50	15.00	95	−1.50	12.18	−2.00	[−5.87; 1.87]	6.4%	8.3%
Bucknall−2012	232	−2.99	12.56	232	1.38	11.33	−4.37	[−6.55; −2.19]	20.2%	14.9%
Common effect model							−3.80	[−5.70; −1.90]	26.6%	−−
Random effects model							−3.75	[−16.99; 9.50]	−−	23.1%
Heterogeneity: $I^2 = 9\%$, $\tau^2 = 0.2392$, $p = 0.30$										
KT_setting = Not reported										
Fan−2012	209	51.30	13.30	217	51.80	13.30	−0.50	[−3.03; 2.03]	15.0%	13.2%
Ko−2017	90	−6.90	15.30	90	−0.10	13.80	−6.80	[−11.06; −2.54]	5.3%	7.3%
Common effect model							−2.14	[−4.31; 0.03]	20.3%	−−
Random effects model							−3.41	[−43.31; 36.50]	−−	20.4%
Heterogeneity: $I^2 = 84\%$, $\tau^2 = 16.6557$, $p = 0.01$										
KT_setting = Home + outpatient clinic										
Herna..ndez−2015	71	43.00	20.00	84	49.00	22.00	−6.00	[−12.62; 0.62]	2.2%	3.7%
KT_setting = Home + GP clinic										
Pinnock−2013	128	68.20	16.30	128	67.30	17.30	0.90	[−3.22; 5.02]	5.7%	7.6%
KT_setting = Disease/HF/COPD/HF clinic										
Khdour−2009	86	61.80	16.55	87	65.30	18.62	−3.50	[−8.75; 1.75]	3.5%	5.3%
KT_setting = Primary care clinic										
Wetering−2010	102			97			−2.60	[−5.15; −0.05]	14.8%	13.1%
Kruis−2016	241	−0.40	12.67	232	0.33	13.00	−0.73	[−3.04; 1.58]	17.9%	14.2%
Common effect model							−1.58	[−3.29; 0.14]	32.7%	−−
Random effects model							−1.59	[−13.42; 10.25]	−−	27.3%
Heterogeneity: $I^2 = 12\%$, $\tau^2 = 0.2060$, $p = 0.29$										
KT_setting = GP clinic										
Zwar−2016	136	16.85	15.20	104	17.06	14.90	−0.21	[−4.05; 3.63]	6.5%	8.4%
KT_setting = Community										
Walters−2013	67	41.90	18.90	69	40.50	17.40	1.40	[−4.71; 7.51]	2.6%	4.2%
**Common effect model**							**−2.14**	**[−3.12; −1.16]**	**100.0%**	**−−**
**Random effects model**							**−2.12**	**[−3.72; −0.51]**	**−−**	**100.0%**
**Prediction interval**								**[−5.76; 1.52]**		

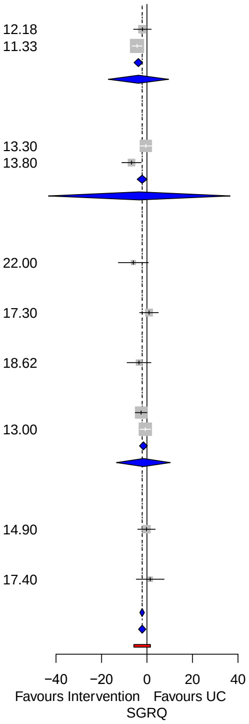

Heterogeneity: $I^2 = 44\%$, $\tau^2 = 2.0953$, $p = 0.06$
Test for subgroup differences (fixed effect): $c_7^2 = 9.28$, df = 7 ($p = 0.23$)
Test for subgroup differences (random effects): $c_7^2 = 8.82$, df = 7 ($p = 0.27$)

Study	Total.KT	Mean.KT	SD.KT	Total.UC	Mean.UC	SD.UC	MD	95%−CI	Weight (common)	Weight (random)
KT_setting = Home										
Chi−2012	84	21.11	18.30	87	34.53	14.85	−13.42	[−18.43; −8.41]	16.0%	15.3%
Wagenaar−2019	300	26.90	29.43	150	26.50	32.50	0.40	[−5.78; 6.58]	10.5%	14.1%
Common effect model							−7.94	[−11.83; −4.05]	26.6%	−−
Random effects model							−6.63	[−94.42; 81.15]	−−	29.5%
Heterogeneity: $I^2 = 91\%$, $\tau^2 = 87.2711$, $p < 0.01$										
KT_setting = Not reported										
Ortiz−Bautista−2017	84	23.46	22.10	43	19.80	23.30	3.66	[−4.76; 12.08]	5.7%	11.8%
KT_setting = GP clinic										
Vaillant−Roussel−2016	76	33.40	22.10	83	27.20	23.30	6.20	[−0.86; 13.26]	8.1%	13.2%
KT_setting = Disease/HF/COPD/HF clinic										
Ojeda−2005	76	35.70	18.90	77	37.80	21.00	−2.10	[−8.43; 4.23]	10.0%	14.0%
de la Porte−2007	118	30.20	22.10	122	34.50	23.30	−4.30	[−10.04; 1.44]	12.2%	14.6%
Stewart−2012	137	−8.00	15.84	143	−9.75	11.68	1.75	[−1.52; 5.02]	37.5%	16.9%
Common effect model							−0.13	[−2.72; 2.46]	59.7%	−−
Random effects model							−0.90	[−8.90; 7.09]	−−	45.5%
Heterogeneity: $I^2 = 45\%$, $\tau^2 = 5.7257$, $p = 0.16$										
**Common effect model**							**−1.48**	**[−3.48; 0.52]**	**100.0%**	**−−**
**Random effects model**							**−1.37**	**[−7.39; 4.64]**	**−−**	**100.0%**
**Prediction interval**								**[−17.65; 14.91]**		

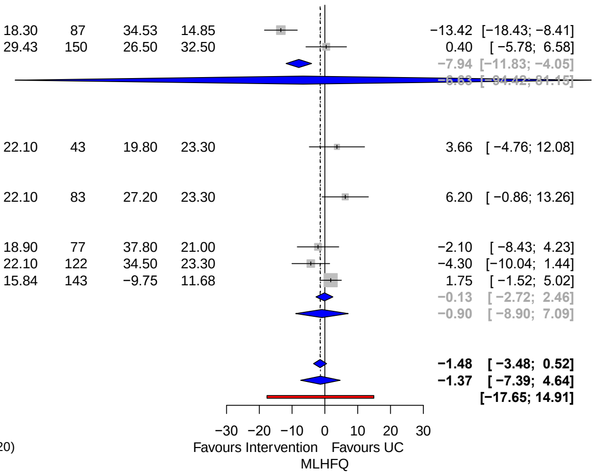

Heterogeneity: $I^2 = 82\%$, $\tau^2 = 33.9067$, $p < 0.01$
Test for subgroup differences (fixed effect): $c_3^2 = 17.63$, df = 3 ($p < 0.01$)
Test for subgroup differences (random effects): $c_3^2 = 4.68$, df = 3 ($p = 0.20$)

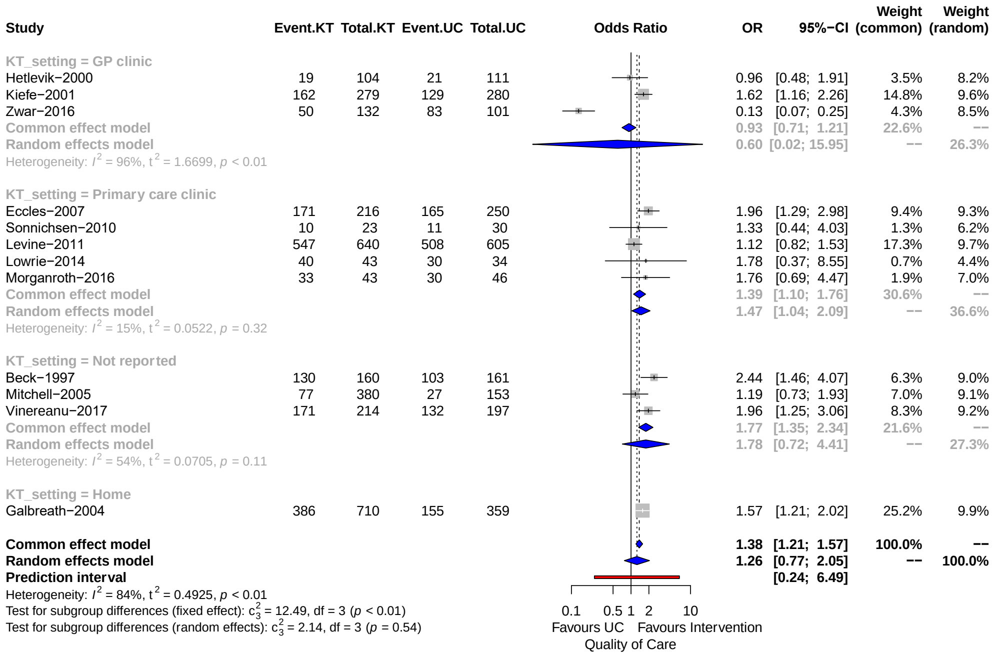

Number of chronic diseases

* Defined as the primary condition that is being treated/managed in the trial (e.g., hypertension).

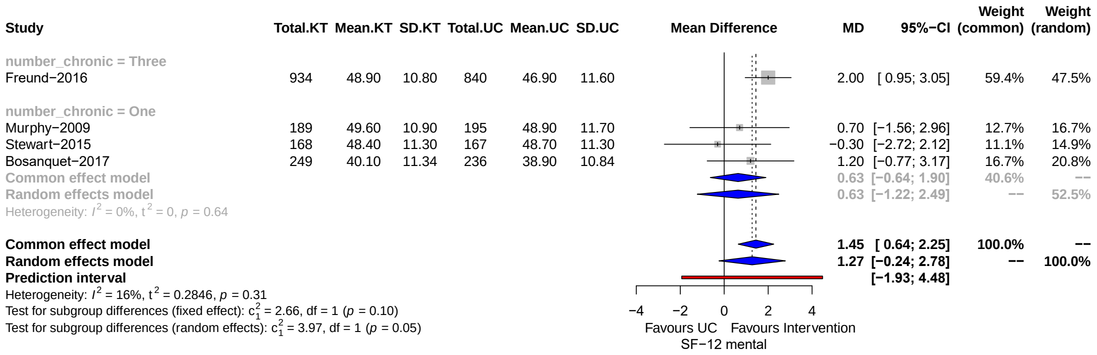

Study	Total.KT	Mean.KT	SD.KT	Total.UC	Mean.UC	SD.UC	MD	95%−CI	Weight (common)	Weight (random)
number_chronic = Four										
Hogg−2009	120			121			1.10	[−1.55; 3.75]	0.3%	7.4%
number_chronic = One										
Subramanian−2004	355	3.70	1.30	365	2.10	1.30	1.60	[1.41; 1.79]	68.1%	8.4%
Galbreath−2004	710	53.50	10.75	359	53.10	10.10	0.40	[−0.91; 1.71]	1.4%	8.2%
Eccles−2007	311	45.80	12.10	360	46.20	11.80	−0.40	[−2.22; 1.42]	0.7%	7.9%
Peters−Klimm−2010	97	46.60	9.90	100	46.50	9.90	0.10	[−2.67; 2.87]	0.3%	7.3%
Markle−Reid−2011	43	69.55	22.12	39	67.57	18.58	1.98	[−6.84; 10.80]	0.0%	3.3%
Walters−2013	67	50.20	11.40	68	50.50	10.50	−0.30	[−4.00; 3.40]	0.2%	6.6%
Karhula(a)−2015	190	−0.05	9.22	79	0.55	8.65	−0.60	[−2.91; 1.71]	0.5%	7.6%
Karhula(b)−2015	180	1.06	9.16	70	1.84	7.04	−0.78	[−2.90; 1.34]	0.5%	7.7%
Xiao−2016	31	38.70	7.00	30	23.00	8.60	15.70	[11.76; 19.64]	0.2%	6.4%
Kruis−2016	240	0.73	9.67	231	0.09	9.77	0.64	[−1.12; 2.40]	0.8%	8.0%
Vaillant−Roussel−2016	76	58.00	22.10	83	58.70	23.90	−0.70	[−7.85; 6.45]	0.0%	4.2%
Leibovici−2017	682			678			−1.57	[−1.93; −1.21]	19.1%	8.4%
Common effect model							0.89	[0.72; 1.05]	91.8%	−−
Random effects model							1.20	[−1.67; 4.08]	−−	84.2%
Heterogeneity: I^2 = 96%, t^2 = 16.7837, p < 0.01										
number_chronic = Five or more										
Fihn−2004	123			68			0.53	[−0.03; 1.09]	7.8%	8.4%
**Common effect model**							**0.86**	**[0.70; 1.02]**	**100.0%**	**−−**
**Random effects model**							**1.11**	**[−1.25; 3.47]**	**−−**	**100.0%**
**Prediction interval**								**[−7.08; 9.30]**		

Heterogeneity: I^2 = 96%, t^2 = 13.0188, p < 0.01
Test for subgroup differences (fixed effect): c^2_2 = 1.48, df = 2 (p = 0.48)
Test for subgroup differences (random effects): c^2_2 = 0.41, df = 2 (p = 0.82)

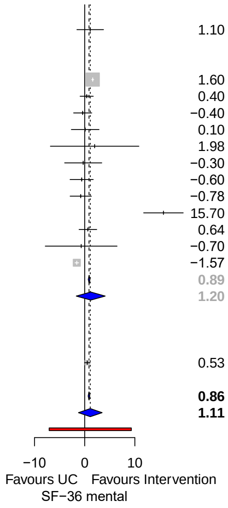

Study	Total.KT	Mean.KT	SD.KT	Total.UC	Mean.UC	SD.UC	MD	95%−CI	Weight (common)	Weight (random)
number_chronic = Three										
Freund−2016	777	36.50	10.60	711	35.50	10.20	1.00	[−0.06; 2.06]	63.4%	20.9%
number_chronic = One										
Liu−2008	24	47.90	7.35	24	30.90	10.78	17.00	[11.78; 22.22]	2.6%	18.2%
Murphy−2009	159	40.50	11.10	173	38.80	11.10	1.70	[−0.69; 4.09]	12.4%	20.4%
Stewart−2015	116	38.10	10.80	112	36.60	12.10	1.50	[−1.48; 4.48]	8.0%	20.0%
Bosanquet−2017	249	34.00	13.51	236	35.10	12.11	−1.10	[−3.38; 1.18]	13.6%	20.5%
Common effect model							1.70	[0.31; 3.09]	36.6%	−−
Random effects model							4.52	[−8.34; 17.38]	−−	79.1%
Heterogeneity: I^2 = 92%, t^2 = 59.7557, p < 0.01										
**Common effect model**							**1.26**	**[0.42; 2.10]**	**100.0%**	**−−**
**Random effects model**							**3.72**	**[−5.08; 12.52]**	**−−**	**100.0%**
**Prediction interval**								**[−19.63; 27.07]**		

Heterogeneity: I^2 = 90%, t^2 = 44.4548, p < 0.01
Test for subgroup differences (fixed effect): c^2_1 = 0.62, df = 1 (p = 0.43)
Test for subgroup differences (random effects): c^2_1 = 0.75, df = 1 (p = 0.39)

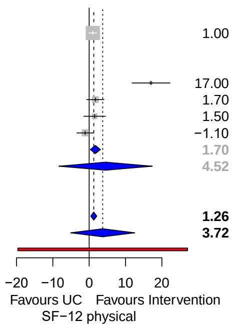

Study	Total.KT	Mean.KT	SD.KT	Total.UC	Mean.UC	SD.UC	MD	95%−CI	Weight (common)	Weight (random)
**number_chronic = Four**										
Hogg−2009	109			114			−1.60	[−4.05; 0.85]	0.8%	4.6%
**number_chronic = One**										
Subramanian−2004	269	−0.60	2.00	280	1.30	2.00	−1.90	[−2.23; −1.57]	43.5%	15.0%
Galbreath−2004	354	32.10	12.40	359	33.40	13.40	−1.30	[−3.19; 0.59]	1.4%	6.4%
Schrader−2005	213	36.70	7.35	237	37.60	10.78	−0.90	[−2.59; 0.79]	1.7%	7.3%
Eccles−2007	311	29.70	15.60	360	30.10	15.30	−0.40	[−2.75; 1.95]	0.9%	4.9%
Peters−Klimm−2010	97	38.30	8.60	100	38.00	8.60	0.30	[−2.10; 2.70]	0.8%	4.7%
Markle−Reid−2011	43	48.29	23.23	39	47.81	18.96	0.48	[−8.66; 9.62]	0.1%	0.5%
Walters−2013	58	38.50	10.30	62	38.50	9.40	−0.00	[−3.54; 3.54]	0.4%	2.6%
Karhula−2015a	162	1.25	6.27	68	0.39	4.65	0.86	[−0.61; 2.33]	2.3%	8.4%
Karhula−2015b	146	0.53	5.76	55	0.51	6.43	0.02	[−1.92; 1.96]	1.3%	6.3%
Xiao−2016	31	41.10	7.70	30	41.60	8.70	−0.50	[−4.63; 3.63]	0.3%	2.0%
Kruis−2016	240	−1.10	8.65	231	−0.48	8.77	−0.62	[−2.19; 0.95]	2.0%	7.9%
Vaillant−Roussel−2016	76	52.80	23.80	83	51.60	25.50	1.20	[−6.46; 8.86]	0.1%	0.6%
Leibovici−2017	682			678			−1.53	[−1.95; −1.11]	27.2%	14.6%
**Common effect model**							**−1.55**	**[−1.80; −1.31]**	**81.8%**	**−−**
**Random effects model**							**−0.81**	**[−1.38; −0.24]**	**−−**	**81.3%**
Heterogeneity: $I^2 = 50\%$, $\tau^2 = 0.5776$, $p = 0.02$										
**number_chronic = Five or more**										
Fihn−2004	123			68			0.26	[−0.27; 0.79]	17.3%	14.1%
**Common effect model**							**−1.24**	**[−1.46; −1.02]**	**100.0%**	**−−**
**Random effects model**							**−0.68**	**[−1.20; −0.15]**	**−−**	**100.0%**
**Prediction interval**								**[−2.55; 1.20]**		

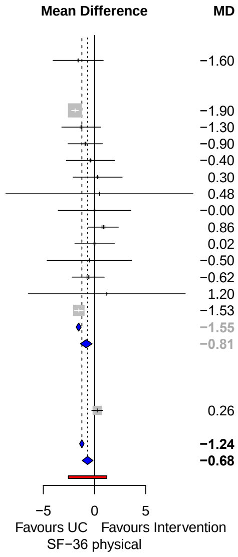

Heterogeneity: $I^2 = 77\%$, $\tau^2 = 0.6505$, $p < 0.01$
Test for subgroup differences (fixed effect): $c_2^2 = 37.11$, df = 2 ($p < 0.01$)
Test for subgroup differences (random effects): $c_2^2 = 9.12$, df = 2 ($p = 0.01$)

Study	Total.KT	Mean.KT	SD.KT	Total.UC	Mean.UC	SD.UC	MD	95%−CI	Weight (common)	Weight (random)
**number_chronic = One**										
Lopez Cabezas−2006	70	0.64	0.15	64	0.61	0.18	0.03	[−0.02; 0.09]	1.7%	7.1%
Bond−2007	810	0.58	0.07	422	0.58	0.07	−0.00	[−0.01; 0.01]	69.9%	8.7%
Stewart−2012	84	−0.18	0.35	87	−0.14	0.36	−0.05	[−0.15; 0.06]	0.5%	4.7%
van de Ven−2013	49	0.35	0.26	79	0.36	0.22	−0.01	[−0.10; 0.08]	0.7%	5.6%
Forster−2015	134	0.51	0.46	128	0.56	0.48	−0.05	[−0.16; 0.06]	0.4%	4.4%
Sackley−2015	160	0.20	0.36	123	0.18	0.31	0.02	[−0.06; 0.10]	0.9%	6.0%
Stewart−2015	116	0.68	0.25	112	0.68	0.26	−0.00	[−0.07; 0.07]	1.2%	6.6%
Kruis−2016	240	−0.04	0.24	231	−0.01	0.24	−0.03	[−0.07; 0.01]	2.9%	7.7%
Beck−2017	97	0.00	0.25	98	0.00	0.25	−0.00	[−0.07; 0.07]	1.1%	6.4%
**Common effect model**							**−0.00**	**[−0.01; 0.01]**	**79.2%**	**−−**
**Random effects model**							**−0.00**	**[−0.01; 0.01]**	**−−**	**57.2%**
Heterogeneity: $I^2 = 0\%$, $\tau^2 = 0$, $p = 0.76$										
**number_chronic = Three**										
Kennedy−2013	277	0.60	0.30	409	0.60	0.30	−0.00	[−0.05; 0.05]	2.6%	7.6%
Freund−2016	864	0.65	0.22	806	0.61	0.23	0.04	[0.02; 0.06]	11.5%	8.4%
Schafer−2017	86	0.68	0.32	87	0.70	0.28	−0.02	[−0.11; 0.07]	0.7%	5.5%
Lin−2018	87	0.22	0.16	91	−0.01	0.18	0.23	[0.18; 0.28]	2.1%	7.4%
**Common effect model**							**0.06**	**[0.04; 0.07]**	**16.8%**	**−−**
**Random effects model**							**0.06**	**[−0.12; 0.24]**	**−−**	**28.8%**
Heterogeneity: $I^2 = 95\%$, $\tau^2 = 0.0118$, $p < 0.01$										
**number_chronic = Two**										
Van Der Aa−2015	131	0.70	0.30	134	0.70	0.30	−0.00	[−0.07; 0.07]	1.0%	6.3%
**number_chronic = Five or more**										
McManus−2014	276	0.82	0.25	276	0.81	0.26	0.01	[−0.03; 0.05]	2.9%	7.7%
**Common effect model**							**0.01**	**[0.00; 0.02]**	**100.0%**	**−−**
**Random effects model**							**0.02**	**[−0.02; 0.05]**	**−−**	**100.0%**
**Prediction interval**								**[−0.12; 0.15]**		

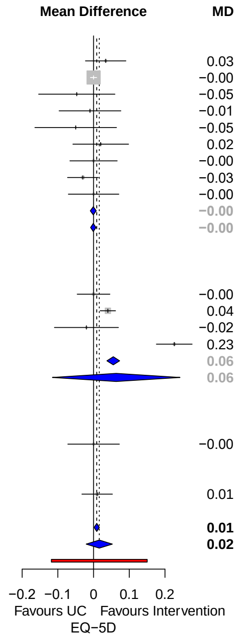

Heterogeneity: $I^2 = 85\%$, $\tau^2 = 0.0035$, $p < 0.01$
Test for subgroup differences (fixed effect): $c_3^2 = 31.31$, df = 3 ($p < 0.01$)
Test for subgroup differences (random effects): $c_3^2 = 1.53$, df = 3 ($p = 0.68$)

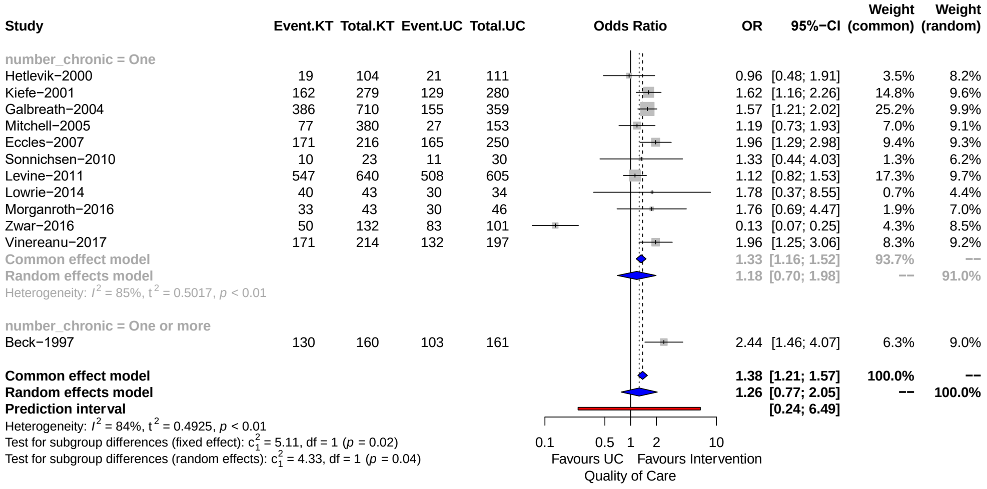

Comorbidities

Comorbidities are additional diseases existed or occurred during the study that the individuals had besides the primary chronic disease.[186, 187]

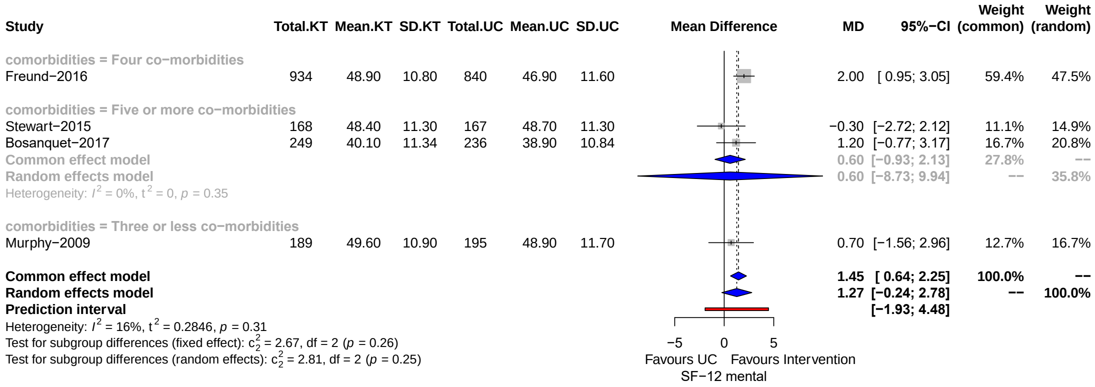

Study	Total.KT	Mean.KT	SD.KT	Total.UC	Mean.UC	SD.UC	MD	95%−CI	Weight (common)	Weight (random)
**comorbidities = Three or less co−morbidities**										
Hogg−2009	120			121			1.10	[−1.55; 3.75]	0.3%	7.4%
Walters−2013	67	50.20	11.40	68	50.50	10.50	−0.30	[−4.00; 3.40]	0.2%	6.6%
Xiao−2016	31	38.70	7.00	30	23.00	8.60	15.70	[11.76; 19.64]	0.2%	6.4%
**Common effect model**							**4.09**	**[2.20; 5.98]**	**0.7%**	**−−**
**Random effects model**							**5.45**	**[−16.49; 27.38]**	**−−**	**20.5%**
Heterogeneity: $I^2 = 95\%$, $\tau^2 = 74.3253$, $p < 0.01$										
**comorbidities = Five or more co−morbidities**										
Subramanian−2004	355	3.70	1.30	365	2.10	1.30	1.60	[1.41; 1.79]	68.1%	8.4%
Galbreath−2004	710	53.50	10.75	359	53.10	10.10	0.40	[−0.91; 1.71]	1.4%	8.2%
Peters−Klimm−2010	97	46.60	9.90	100	46.50	9.90	0.10	[−2.67; 2.87]	0.3%	7.3%
Karhula(a)−2015	190	−0.05	9.22	79	0.55	8.65	−0.60	[−2.91; 1.71]	0.5%	7.6%
Karhula(b)−2015	180	1.06	9.16	70	1.84	7.04	−0.78	[−2.90; 1.34]	0.5%	7.7%
Leibovici−2017	682			678			−1.57	[−1.93; −1.21]	19.1%	8.4%
**Common effect model**							**0.88**	**[0.71; 1.04]**	**89.9%**	**−−**
**Random effects model**							**−0.08**	**[−1.40; 1.23]**	**−−**	**47.7%**
Heterogeneity: $I^2 = 98\%$, $\tau^2 = 1.5181$, $p < 0.01$										
**comorbidities = Four co−morbidities**										
Fihn−2004	123			68			0.53	[−0.03; 1.09]	7.8%	8.4%
Markle−Reid−2011	43	69.55	22.12	39	67.57	18.58	1.98	[−6.84; 10.80]	0.0%	3.3%
Kruis−2016	240	0.73	9.67	231	0.09	9.77	0.64	[−1.12; 2.40]	0.8%	8.0%
Vaillant−Roussel−2016	76	58.00	22.10	83	58.70	23.90	−0.70	[−7.85; 6.45]	0.0%	4.2%
**Common effect model**							**0.54**	**[0.01; 1.07]**	**8.7%**	**−−**
**Random effects model**							**0.54**	**[0.30; 0.78]**	**−−**	**23.8%**
Heterogeneity: $I^2 = 0\%$, $\tau^2 = 0$, $p = 0.97$										
**comorbidities = Not reported**										
Eccles−2007	311	45.80	12.10	360	46.20	11.80	−0.40	[−2.22; 1.42]	0.7%	7.9%
**Common effect model**							**0.86**	**[0.70; 1.02]**	**100.0%**	**−−**
**Random effects model**							**1.11**	**[−1.25; 3.47]**	**−−**	**100.0%**
**Prediction interval**								**[−7.08; 9.30]**		

Heterogeneity: $I^2 = 96\%$, $\tau^2 = 13.0188$, $p < 0.01$
Test for subgroup differences (fixed effect): $\chi^2_3 = 14.53$, df = 3 ($p < 0.01$)
Test for subgroup differences (random effects): $\chi^2_3 = 3.36$, df = 3 ($p = 0.34$)

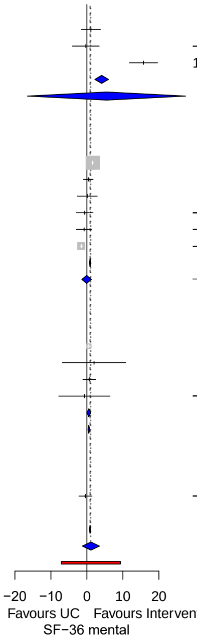

Study	Total.KT	Mean.KT	SD.KT	Total.UC	Mean.UC	SD.UC	MD	95%−CI	Weight (common)	Weight (random)
**comorbidities = Four co−morbidities**										
Freund−2016	777	36.50	10.60	711	35.50	10.20	1.00	[−0.06; 2.06]	63.4%	20.9%
**comorbidities = Five or more co−morbidities**										
Stewart−2015	116	38.10	10.80	112	36.60	12.10	1.50	[−1.48; 4.48]	8.0%	20.0%
Bosanquet−2017	249	34.00	13.51	236	35.10	12.11	−1.10	[−3.38; 1.18]	13.6%	20.5%
**Common effect model**							**−0.14**	**[−1.95; 1.67]**	**21.6%**	**−−**
**Random effects model**							**0.02**	**[−16.34; 16.37]**	**−−**	**40.5%**
Heterogeneity: $I^2 = 46\%$, $\tau^2 = 1.5464$, $p = 0.17$										
**comorbidities = Three or less co−morbidities**										
Murphy−2009	159	40.50	11.10	173	38.80	11.10	1.70	[−0.69; 4.09]	12.4%	20.4%
**comorbidities = Not reported**										
Liu−2008	24	47.90	7.35	24	30.90	10.78	17.00	[11.78; 22.22]	2.6%	18.2%
**Common effect model**							**1.26**	**[0.42; 2.10]**	**100.0%**	**−−**
**Random effects model**							**3.72**	**[−5.08; 12.52]**	**−−**	**100.0%**
**Prediction interval**								**[−19.63; 27.07]**		

Heterogeneity: $I^2 = 90\%$, $\tau^2 = 44.4548$, $p < 0.01$
Test for subgroup differences (fixed effect): $\chi^2_3 = 37.60$, df = 3 ($p < 0.01$)
Test for subgroup differences (random effects): $\chi^2_3 = 36.08$, df = 3 ($p < 0.01$)

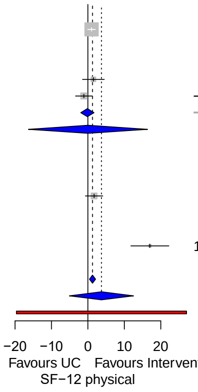

Study	Total.KT	Mean.KT	SD.KT	Total.UC	Mean.UC	SD.UC	MD	95%−CI	Weight (common)	Weight (random)
**comorbidities = Three or less co−morbidities**										
Hogg−2009	109			114			−1.60	[−4.05; 0.85]	0.8%	4.6%
Walters−2013	58	38.50	10.30	62	38.50	9.40	−0.00	[−3.54; 3.54]	0.4%	2.6%
Xiao−2016	31	41.10	7.70	30	41.60	8.70	−0.50	[−4.63; 3.63]	0.3%	2.0%
Common effect model							−0.97	[−2.78; 0.84]	1.5%	−−
Random effects model							−0.97	[−3.13; 1.19]	−−	9.2%
Heterogeneity: $I^2 = 0\%$, $\tau^2 = 0$, $p = 0.74$										
**comorbidities = Five or more co−morbidities**										
Subramanian−2004	269	−0.60	2.00	280	1.30	2.00	−1.90	[−2.23; −1.57]	43.5%	15.0%
Galbreath−2004	354	32.10	12.40	359	33.40	13.40	−1.30	[−3.19; 0.59]	1.4%	6.4%
Schrader−2005	213	36.70	7.35	237	37.60	10.78	−0.90	[−2.59; 0.79]	1.7%	7.3%
Peters−Klimm−2010	97	38.30	8.60	100	38.00	8.60	0.30	[−2.10; 2.70]	0.8%	4.7%
Karhula−2015a	162	1.25	6.27	68	0.39	4.65	0.86	[−0.61; 2.33]	2.3%	8.4%
Karhula−2015b	146	0.53	5.76	55	0.51	6.43	0.02	[−1.92; 1.96]	1.3%	6.3%
Leibovici−2017	682			678			−1.53	[−1.95; −1.11]	27.2%	14.6%
Common effect model							−1.60	[−1.85; −1.35]	78.2%	−−
Random effects model							−0.88	[−1.86; 0.10]	−−	62.8%
Heterogeneity: $I^2 = 70\%$, $\tau^2 = 0.7670$, $p < 0.01$										
**comorbidities = Four co−morbidities**										
Fihn−2004	123			68			0.26	[−0.27; 0.79]	17.3%	14.1%
Markle−Reid−2011	43	48.29	23.23	39	47.81	18.96	0.48	[−8.66; 9.62]	0.1%	0.5%
Kruis−2016	240	−1.10	8.65	231	−0.48	8.77	−0.62	[−2.19; 0.95]	2.0%	7.9%
Vaillant−Roussel−2016	76	52.80	23.80	83	51.60	25.50	1.20	[−6.46; 8.86]	0.1%	0.6%
Common effect model							0.18	[−0.32; 0.68]	19.5%	−−
Random effects model							0.18	[−0.33; 0.68]	−−	23.1%
Heterogeneity: $I^2 = 0\%$, $\tau^2 = 0$, $p = 0.76$										
**comorbidities = Not reported**										
Eccles−2007	311	29.70	15.60	360	30.10	15.30	−0.40	[−2.75; 1.95]	0.9%	4.9%
**Common effect model**							**−1.24**	**[−1.46; −1.02]**	**100.0%**	**−−**
**Random effects model**							**−0.68**	**[−1.20; −0.15]**	**−−**	**100.0%**
**Prediction interval**								**[−2.55; 1.20]**		

Heterogeneity: $I^2 = 77\%$, $\tau^2 = 0.6505$, $p < 0.01$
Test for subgroup differences (fixed effect): $\chi^2_3 = 39.48$, df = 3 ($p < 0.01$)
Test for subgroup differences (random effects): $\chi^2_3 = 9.75$, df = 3 ($p = 0.02$)

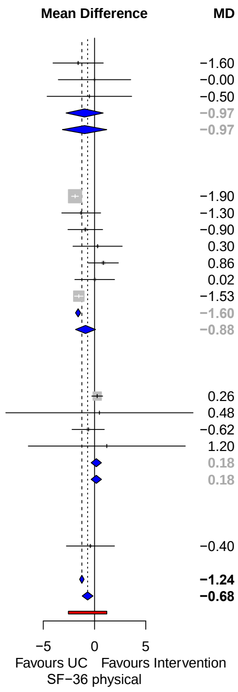

Study	Total.KT	Mean.KT	SD.KT	Total.UC	Mean.UC	SD.UC	MD	95%−CI	Weight (common)	Weight (random)
**comorbidities = Four co−morbidities**										
Bond−2007	810	0.58	0.07	422	0.58	0.07	−0.00	[−0.01; 0.01]	69.9%	8.7%
McManus−2014	276	0.82	0.25	276	0.81	0.26	0.01	[−0.03; 0.05]	2.9%	7.7%
Freund−2016	864	0.65	0.22	806	0.61	0.23	0.04	[0.02; 0.06]	11.5%	8.4%
Kruis−2016	240	−0.04	0.24	231	−0.01	0.24	−0.03	[−0.07; 0.01]	2.9%	7.7%
Common effect model							0.00	[0.00; 0.01]	87.2%	−−
Random effects model							0.01	[−0.04; 0.05]	−−	32.4%
Heterogeneity: $I^2 = 78\%$, $\tau^2 = 0.0006$, $p < 0.01$										
**comorbidities = Not reported**										
Kennedy−2013	277	0.60	0.30	409	0.60	0.30	−0.00	[−0.05; 0.05]	2.6%	7.6%
van de Ven−2013	49	0.35	0.26	79	0.36	0.22	−0.01	[−0.10; 0.08]	0.7%	5.6%
Beck−2017	97	0.00	0.25	98	0.00	0.25	−0.00	[−0.07; 0.07]	1.1%	6.4%
Lin−2018	87	0.22	0.16	91	−0.01	0.18	0.23	[0.18; 0.28]	2.1%	7.4%
Common effect model							0.07	[0.04; 0.10]	6.5%	−−
Random effects model							0.06	[−0.13; 0.24]	−−	26.9%
Heterogeneity: $I^2 = 94\%$, $\tau^2 = 0.0126$, $p < 0.01$										
**comorbidities = Three or less co−morbidities**										
Forster−2015	134	0.51	0.46	128	0.56	0.48	−0.05	[−0.16; 0.06]	0.4%	4.4%
Van Der Aa−2015	131	0.70	0.30	134	0.70	0.30	−0.00	[−0.07; 0.07]	1.0%	6.3%
Common effect model							−0.01	[−0.08; 0.05]	1.4%	−−
Random effects model							−0.01	[−0.30; 0.27]	−−	10.7%
Heterogeneity: $I^2 = 0\%$, $\tau^2 = 0$, $p = 0.47$										
**comorbidities = Five or more co−morbidities**										
Lopez Cabezas−2006	70	0.64	0.15	64	0.61	0.18	0.03	[−0.02; 0.09]	1.7%	7.1%
Stewart−2012	84	−0.18	0.35	87	−0.14	0.36	−0.05	[−0.15; 0.06]	0.5%	4.7%
Sackley−2015	160	0.20	0.36	123	0.18	0.31	0.02	[−0.06; 0.10]	0.9%	6.0%
Stewart−2015	116	0.68	0.25	112	0.68	0.26	−0.00	[−0.07; 0.07]	1.2%	6.6%
Schafer−2017	86	0.68	0.32	87	0.70	0.28	−0.02	[−0.11; 0.07]	0.7%	5.5%
Common effect model							0.01	[−0.03; 0.04]	4.9%	−−
Random effects model							0.01	[−0.03; 0.04]	−−	29.9%
Heterogeneity: $I^2 = 0\%$, $\tau^2 = 0$, $p = 0.67$										
**Common effect model**							**0.01**	**[0.00; 0.02]**	**100.0%**	**−−**
**Random effects model**							**0.02**	**[−0.02; 0.05]**	**−−**	**100.0%**
**Prediction interval**								**[−0.12; 0.15]**		

Heterogeneity: $I^2 = 85\%$, $\tau^2 = 0.0035$, $p < 0.01$
Test for subgroup differences (fixed effect): $\chi^2_3 = 21.17$, df = 3 ($p < 0.01$)
Test for subgroup differences (random effects): $\chi^2_3 = 1.59$, df = 3 ($p = 0.66$)

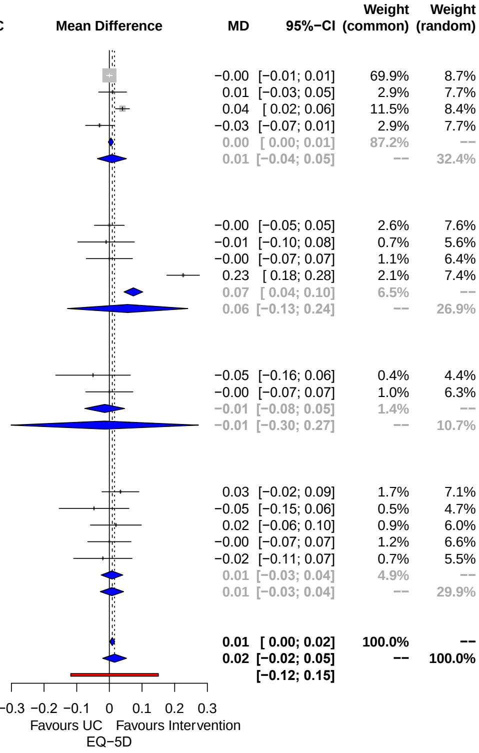

Study	Total.KT	Mean.KT	SD.KT	Total.UC	Mean.UC	SD.UC	MD	95%−CI	Weight (common)	Weight (random)
**comorbidities = Four co−morbidities**										
Bourbeau−2003	96	−3.50	15.00	95	−1.50	12.18	−2.00	[−5.87; 1.87]	6.4%	8.3%
Kruis−2016	241	−0.40	12.67	232	0.33	13.00	−0.73	[−3.04; 1.58]	17.9%	14.2%
Common effect model							−1.06	[−3.05; 0.92]	24.3%	−−
Random effects model							−1.06	[−8.17; 6.04]	−−	22.4%
Heterogeneity: $I^2 = 0\%$, $t^2 = 0$, $p = 0.58$										
**comorbidities = Three or less co−morbidities**										
Wetering−2010	102			97			−2.60	[−5.15; −0.05]	14.8%	13.1%
Bucknall−2012	232	−2.99	12.56	232	1.38	11.33	−4.37	[−6.55; −2.19]	20.2%	14.9%
Pinnock−2013	128	68.20	16.30	128	67.30	17.30	0.90	[−3.22; 5.02]	5.7%	7.6%
Walters−2013	67	41.90	18.90	69	40.50	17.40	1.40	[−4.71; 7.51]	2.6%	4.2%
Zwar−2016	136	16.85	15.20	104	17.06	14.90	−0.21	[−4.05; 3.63]	6.5%	8.4%
Common effect model							−2.40	[−3.79; −1.01]	49.7%	−−
Random effects model							−1.68	[−4.71; 1.35]	−−	48.1%
Heterogeneity: $I^2 = 52\%$, $t^2 = 3.2956$, $p = 0.08$										
**comorbidities = Five or more co−morbidities**										
Fan−2012	209	51.30	13.30	217	51.80	13.30	−0.50	[−3.03; 2.03]	15.0%	13.2%
Herna..ndez−2015	71	43.00	20.00	84	49.00	22.00	−6.00	[−12.62; 0.62]	2.2%	3.7%
Ko−2017	90	−6.90	15.30	90	−0.10	13.80	−6.80	[−11.06; −2.54]	5.3%	7.3%
Common effect model							−2.52	[−4.58; −0.45]	22.5%	−−
Random effects model							−3.93	[−12.96; 5.10]	−−	24.1%
Heterogeneity: $I^2 = 73\%$, $t^2 = 10.3549$, $p = 0.02$										
**comorbidities = Not reported**										
Khdour−2009	86	61.80	16.55	87	65.30	18.62	−3.50	[−8.75; 1.75]	3.5%	5.3%
**Common effect model**							**−2.14**	**[−3.12; −1.16]**	**100.0%**	**−−**
**Random effects model**							**−2.12**	**[−3.72; −0.51]**	**−−**	**100.0%**
**Prediction interval**								**[−5.76; 1.52]**		

Heterogeneity: $I^2 = 44\%$, $t^2 = 2.0953$, $p = 0.06$
Test for subgroup differences (fixed effect): $c_3^2 = 1.65$, df = 3 ($p = 0.65$)
Test for subgroup differences (random effects): $c_3^2 = 2.50$, df = 3 ($p = 0.48$)

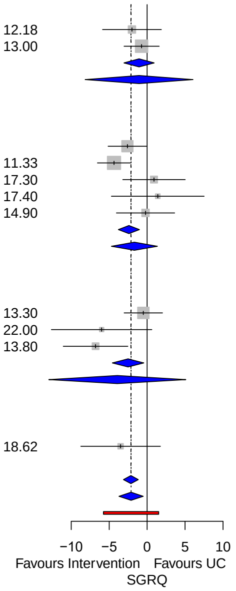

Study	Total.KT	Mean.KT	SD.KT	Total.UC	Mean.UC	SD.UC	MD	95%−CI	Weight (common)	Weight (random)
**comorbidities = Not reported**										
Chi−2012	84	21.11	18.30	87	34.53	14.85	−13.42	[−18.43; −8.41]	16.0%	15.3%
Ortiz−Bautista−2017	84	23.46	22.10	43	19.80	23.30	3.66	[−4.76; 12.08]	5.7%	11.8%
Common effect model							−8.96	[−13.26; −4.66]	21.7%	−−
Random effects model							−5.23	[−113.65; 103.19]	−−	27.2%
Heterogeneity: $I^2 = 91\%$, $t^2 = 133.3826$, $p < 0.01$										
**comorbidities = Four co−morbidities**										
Vaillant−Roussel−2016	76	33.40	22.10	83	27.20	23.30	6.20	[−0.86; 13.26]	8.1%	13.2%
**comorbidities = Five or more co−morbidities**										
de la Porte−2007	118	30.20	22.10	122	34.50	23.30	−4.30	[−10.04; 1.44]	12.2%	14.6%
Stewart−2012	137	−8.00	15.84	143	−9.75	11.68	1.75	[−1.52; 5.02]	37.5%	16.9%
Wagenaar−2019	300	26.90	29.43	150	26.50	32.50	0.40	[−5.78; 6.58]	10.5%	14.1%
Common effect model							0.29	[−2.29; 2.87]	60.2%	−−
Random effects model							−0.19	[−7.92; 7.53]	−−	45.6%
Heterogeneity: $I^2 = 38\%$, $t^2 = 4.1206$, $p = 0.20$										
**comorbidities = Three or less co−morbidities**										
Ojeda−2005	76	35.70	18.90	77	37.80	21.00	−2.10	[−8.43; 4.23]	10.0%	14.0%
**Common effect model**							**−1.48**	**[−3.48; 0.52]**	**100.0%**	**−−**
**Random effects model**							**−1.37**	**[−7.39; 4.64]**	**−−**	**100.0%**
**Prediction interval**								**[−17.65; 14.91]**		

Heterogeneity: $I^2 = 82\%$, $t^2 = 33.9067$, $p < 0.01$
Test for subgroup differences (fixed effect): $c_3^2 = 18.00$, df = 3 ($p < 0.01$)
Test for subgroup differences (random effects): $c_3^2 = 3.73$, df = 3 ($p = 0.29$)

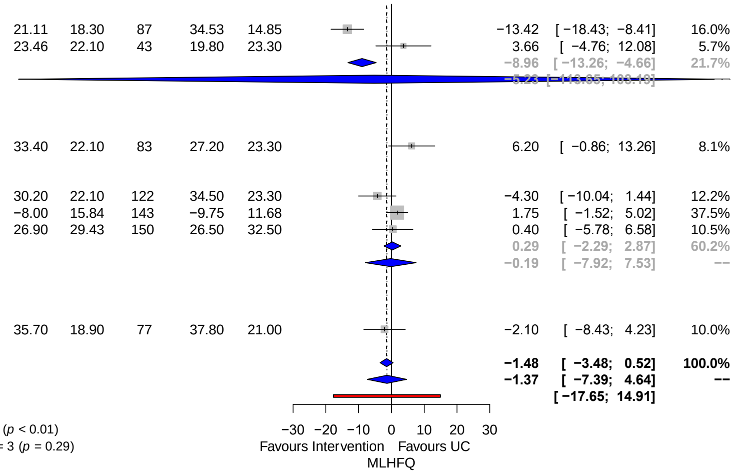

Study	Event.KT	Total.KT	Event.UC	Total.UC	OR	95%−CI	Weight (common)	Weight (random)
**comorbidities = Four co−morbidities**								
Kiefe−2001	162	279	129	280	1.62	[1.16; 2.26]	14.8%	9.6%
**comorbidities = Five or more co−morbidities**								
Beck−1997	130	160	103	161	2.44	[1.46; 4.07]	6.3%	9.0%
Galbreath−2004	386	710	155	359	1.57	[1.21; 2.02]	25.2%	9.9%
Levine−2011	547	640	508	605	1.12	[0.82; 1.53]	17.3%	9.7%
Morganroth−2016	33	43	30	46	1.76	[0.69; 4.47]	1.9%	7.0%
Vinereanu−2017	171	214	132	197	1.96	[1.25; 3.06]	8.3%	9.2%
**Common effect model**					**1.54**	**[1.31; 1.82]**	**59.0%**	**−−**
**Random effects model**					**1.63**	**[1.12; 2.37]**	**−−**	**44.7%**
Heterogeneity: $I^2 = 52\%$, $\tau^2 = 0.0533$, $p = 0.08$								
**comorbidities = Three or less co−morbidities**								
Lowrie−2014	40	43	30	34	1.78	[0.37; 8.55]	0.7%	4.4%
Zwar−2016	50	132	83	101	0.13	[0.07; 0.25]	4.3%	8.5%
**Common effect model**					**0.19**	**[0.11; 0.33]**	**5.0%**	**−−**
**Random effects model**					**0.44**	**[0.00; 6121481.40]**	**−−**	**12.9%**
Heterogeneity: $I^2 = 89\%$, $\tau^2 = 3.0054$, $p < 0.01$								
**comorbidities = Not reported**								
Hetlevik−2000	19	104	21	111	0.96	[0.48; 1.91]	3.5%	8.2%
Mitchell−2005	77	380	27	153	1.19	[0.73; 1.93]	7.0%	9.1%
Eccles−2007	171	216	165	250	1.96	[1.29; 2.98]	9.4%	9.3%
Sonnichsen−2010	10	23	11	30	1.33	[0.44; 4.03]	1.3%	6.2%
**Common effect model**					**1.44**	**[1.09; 1.90]**	**21.2%**	**−−**
**Random effects model**					**1.38**	**[0.82; 2.33]**	**−−**	**32.7%**
Heterogeneity: $I^2 = 26\%$, $\tau^2 = 0.0495$, $p = 0.26$								
**Common effect model**					**1.38**	**[1.21; 1.57]**	**100.0%**	**−−**
**Random effects model**					**1.26**	**[0.77; 2.05]**	**−−**	**100.0%**
**Prediction interval**						**[0.24; 6.49]**		

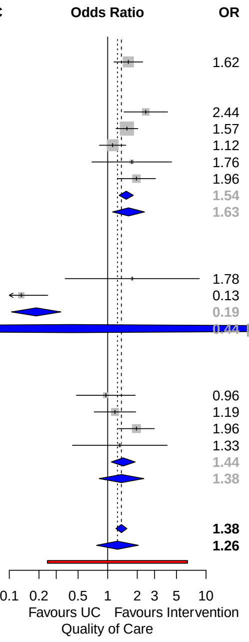

Heterogeneity: $I^2 = 84\%$, $\tau^2 = 0.4925$, $p < 0.01$
Test for subgroup differences (fixed effect): $\chi^2_3 = 48.81$, df = 3 ($p < 0.01$)
Test for subgroup differences (random effects): $\chi^2_3 = 1.64$, df = 3 ($p = 0.65$)

Appendix 18: Sensitivity Analyses of All KT Interventions vs Usual Care

Outcome	No of studies	MD/OR (95% CI) [fixed-effect model]	MD/OR (95% CI) [random-effects model]	95% PI	τ (95% CI)	I^2 (95% CI)	Q (d.f.); p-value
**Excluding Outliers**							
Quality of life: 36 item Short-Form Mental Health Survey (outlier Xiao, 2016[181])	13	MD: 0.84 (0.68 to 0.99)	MD: 0.12 (-0.52 to 0.75)	-2.19 to 2.43	0.98 (0.47 to 1.27)	95% (93% to 97%)	242.87 (12); <0.0001
Quality of life: 12 item Short-Form Physical Health Survey (outlier Liu 2008[119])	4	MD: 0.84 (-0.02 to 1.69)	MD: 0.84 (-0.67 to 2.34)	-1.04 to 2.71	0.00 (0.00 to 4.6)	16% (0% to 87%)	3.55 (3); 0.31
Quality of life: European Quality of Life-5 Dimensions (EQ-5D) (outlier Lin 2018[118])	14	MD: 0.00 (-0.00 to 0.01)	MD: 0.01 (-0.01 to 0.02)	-0.03 to 0.04	0.02 (0.00 to 0.03)	25% (0% to 60%)	17.35 (13); 0.18
Quality of care (outlier Zwar 2016[182])	11	OR: 1.53 (1.34 to 1.75)	OR: 1.55 (1.29 to 1.85)	1.05 to 2.29	0.15 (0.00 to 0.40)	21% (0% to 61%)	12.72 (10); 0.24
**No SD imputations**							
Quality of life: 36 item Short-Form Physical Health Survey	14	MD: -1.24 (-1.47 to 1.02)	MD: -0.65 (-1.22 to -0.08)	-2.63 to 1.34	0.84 (0.33 to 1.13)	79% (65% to 87%)	60.90 (13); <0.0001
Quality of life: Minnesota Living with	6	MD: -1.79 (-3.85 to 0.28)	MD: -2.04 (-9.06 to 4.98)	-20.53 to 16.45	6.07 (3.09 to 16.11)	84% (67% to 92%)	31.39 (5); <0.0001

Heart Failure Questionnaire							
**Low Risk of Bias for attrition bias**							
Quality of life: 12 item Short-Form Mental Health Survey	4	MD: 1.45 (0.64 to 2.25)	MD: 1.27 (-0.24 to 2.78)	-1.93 to 4.48	0.53 (0.00 to 3.45)	16% (0% to 87%)	3.55 (3); 0.31
Quality of life: 36 item Short-Form Mental Health Survey	8	MD: -1.26 (-1.58 to 0.93)	MD: -0.20 (-1.02 to 0.61)	-3.05 to 2.65	1.04 (0.00 to 2.18)	63% (19% to 83%)	18.67 (7); <0.01
Quality of life: 12 item Short-Form Physical Health Survey	5	MD: 1.26 (0.42 to 2.10)	MD: 3.72 (-5.08 to 12.52)	-19.63 to 27.07	6.67 (3.68 to 20.96)	90% (79% to 95%]	39.44 (4); < 0.0001
Quality of life: 36 item Short-Form Physical Health Survey	8	MD: -1.37 (-1.75 to -0.99)	MD: -1.16 (-1.70 to -0.62)	-2.30 to -0.03	0.35 (0.00 to 1.09)	0% (0% to 68%)	4.99 (7); 0.66
Quality of life: European Quality of Life-5 Dimensions (EQ-5D)	12	MD: 0.00 (-0.00 to 0.01)	MD: 0.00 (-0.01 to 0.02)	-0.04 to 0.04	0.02 (0.00 to 0.03)	29% (0% to 64%)	15.40 (11); 0.16
Quality of life: St George's Respiratory Questionnaire	8	MD: -2.13 (-3.18 to -1.09)	MD: -2.04 (-4.08 to 0.01)	-6.51 to 2.44	1.63 (0.00 to 5.13)	54% (0% to 79%)	15.19 (7); 0.03
Quality of life: Minnesota Living with Heart Failure Questionnaire	5	MD: 0.03 (-2.91 to 2.97)	MD: 0.29 (-4.96 to 5.55)	-9.93 to 10.52	2.59 (0.00 to >10.00)	36% (0% to 76%)	6.28 (4); 0.18
Quality of care	2	OR: 1.67 (1.34 to 2.07)	OR: 1.67 (0.48 to 5.83)	N/A	0	0%	0.78 (1); 0.38
**Low Risk of Bias for selective reporting**							
Quality of life: 12 item Short-Form	2	MD: 0.98 (-0.50 to 2.47)	MD: 0.98 (-2.16 to 4.13)	N/A	0	0%	0.11 (1); 0.74

Mental Health Survey							
Quality of life: 36 item Short-Form Mental Health Survey	5	MD: -1.44 (-1.78 to -1.09)	MD: -0.63 (-1.99 to 0.73)	-4.33 to 3.07	0.96 (0.00 to 2.87)	49% (0% to 81%)	7.78 (4); 0.10
Quality of life: 12 item Short-Form Physical Health Survey	2	MD: 0.23 (-1.42 to 1.88)	MD: 0.28 (-17.51 to 18.06)	N/A	1.58	64% (0% to 92%)	2.76 (1); 0.10
Quality of life: 36 item Short-Form Physical Health Survey	5	MD: -1.43 (-1.83 to -1.03)	MD: -1.22 (-1.94 to -0.50)	-2.98 to 0.54	0.40 (0.00 to 1.79)	0% (0% to 79%)	2.60 (4); 0.63
Quality of life: European Quality of Life-5 Dimensions (EQ-5D)	8	MD: -0.01 (-0.04 to 0.01)	MD: -0.01 (-0.03 to 0.00)	-0.04 to 0.02	0 (0.00 to 0.03)	0% (0% to 68%)	2.67 (7); 0.91
Quality of life: St George's Respiratory Questionnaire	4	MD: -1.79 (-3.14 to -0.45)	MD: -2.20 (-6.32 to 1.92)	-11.53 to 7.13	1.83 (0.00 to >10.00)	60% (0% to 87%)	7.52 (3); 0.06
Quality of life: Minnesota Living with Heart Failure Questionnaire	3	MD: 2.14 (-0.54 to 4.81)	MD: 2.14 (-3.17 to 7.44)	-15.21 to 19.48	0.00 (0.00 to >10.00)	0% (0% to 90%)	1.63 (2); 0.44
Quality of care (Eccles, 2007[34])	1	OR: 1.96 (1.29 to 2.98)	N/A	N/A	N/A	N/A	N/A
**Follow-up of 12 months**							
Quality of life: 36 item Short-Form Mental Health Survey	9	MD: 1.56 (1.37 to 1.74)	MD: 1.85 (-2.11 to 5.81)	-10.16 to13.86	4.79 (2.97 to 9.83)	88% (79% to 93%)	65.18 (8); < 0.0001
Quality of life: 12 item Short-Form Physical	1	MD: 17.00 (11.78 to 22.22)	N/A	N/A	N/A	N/A	N/A

Health Survey (Liu, 2008[119])							
Quality of life: 36 item Short-Form Physical Health Survey	10	MD: -1.57 (-1.88 to -1.27)	MD: -0.49 (-1.17 to 0.18)	-3.10 to 2.11	1.03 (0.00 to 1.80)	62% (23% to 81%)	23.35 (9); <0.01
Quality of life: European Quality of Life-5 Dimensions (EQ-5D)	12	MD: 0.01 (-0.00 to 0.01)	MD: 0.02 (-0.03 to 0.06)	-0.14 to 0.17	0.07 (0.04 to 0.12)	87% (79% to 92%)	82.39 (11); <0.0001
Quality of life: St George's Respiratory Questionnaire	10	MD: -2.06 (-3.12 to -1.00)	MD: -2.05 (-3.91 to -0.19)	-6.37 to 2.27	1.69 (0.00 to 4.61)	49% (0% to 75%)	17.58 (9); 0.04
Quality of life: Minnesota Living with Heart Failure Questionnaire	4	MD: -2.59 (-4.89 to -0.30)	MD: -3.84 (-14.84 to 7.16)	-35.49 to 27.81	6.48 (3.04 to >20.48)	88% (73% to 95%)	25.99 (3); <0.0001
Quality of care	8	OR: 1.35 (1.10 to 1.66)	OR: 1.24 (0.55 to 2.77)	0.11 to 13.56	0.91 (0.53 to 1.88)	89% (81% to 94%)	65.61 (7); <0.0001
**Male participants up to 80%**							
Quality of life: 36 item Short-Form Mental Health Survey	12	MD: -1.12 (-1.44 to -0.80)	MD: 1.16 (-1.73 to 4.04)	-8.46 to 10.77	4.12 (2.65 to 7.62)	88% (81% to 92%)	90.04 (11); <0.0001
Quality of life: 12 item Short-Form Physical Health Survey	4	MD: 0.84 (-0.02 to 1.69)	MD: 0.84 (-0.67 to 2.34)	-1.04 to 2.71	0.00 (0.00 to 4.63)	16% (0% to 87%)	3.55 (3); 0.31
Quality of life: 36 item Short-Form Physical Health Survey	13	MD: -1.17 (-1.52 to -0.81)	MD: -0.75 (-1.28 to -0.23)	-1.94 to 0.43	0.45 (0.00 to 0.90)	21% (0% to 59%)	15.17 (12); 0.23
Quality of life: St George's	9	MD: -2.34 (-3.41 to -1.26)	MD: -2.19 (-4.05 to -0.34)	-6.24 to 1.85	1.52 (0.00 to 4.55)	46% (0% to 75%)	14.67 (8); 0.07

Respiratory Questionnaire							
Quality of care	10	OR: 1.40 (1.20 to 1.64)	OR: 1.24 (0.67 to 2.28)	0.18 to 8.62	0.79 (0.48 to 1.49)	87% (78% to 92%)	67.64 (9); <0.0001
**History of prescription**							
Quality of life: 12 item Short-Form Mental Health Survey (Stewart, 2015[164])	1	MD: -0.30 (-2.72 to 2.12)	N/A	N/A	N/A	N/A	N/A
Quality of life: 36 item Short-Form Mental Health Survey	8	MD: -1.32 (-1.65 to -0.99)	MD: -0.38 (-1.24 to 0.47)	-2.86 to 2.10	0.87 (0.00 to 1.47)	52% (0% to 78%)	14.56 (7); 0.04
Quality of life: 12 item Short-Form Physical Health Survey (Stewart, 2015[164])	1	MD: 1.50 (-1.48 to 4.48)	N/A	N/A	N/A	N/A	N/A
Quality of life: 36 item Short-Form Physical Health Survey	8	MD: -1.41 (-1.81 to -1.02)	MD: -1.27 (-1.79 to -0.75)	-2.30 to - 0.24	0.29 (0.00 to 1.12)	0% (0% to 68%)	4.23 (7); 0.75
Quality of life: European Quality of Life-5 Dimensions (EQ-5D)	6	MD: 0.00 (-0.01 to 0.01)	MD: 0.00 (-0.01 to 0.01)	-0.01 to 0.01	0 (0.00 to 0.05)	0% (0% to 75%)	2.30 (5); 0.81
Quality of life: St George's Respiratory Questionnaire	8	MD: -2.72 (-3.97 to -1.47)	MD: -2.68 (-4.91 to -0.44)	-7.55 to 2.20	1.75 (0.00 to 5.35)	47% (0% to 77%)	13.25 (7); 0.07
Quality of life: Minnesota Living with Heart Failure Questionnaire	4	MD: 0.99 (-1.44 to 3.41)	MD: 0.86 (-5.37 to 7.09)	-12.26 to 13.98	2.44 (0.00 to >10.00)	46% (0% to 82%)	5.59 (3); 0.13

Quality of care	7	OR: 1.31 (1.12 to 1.53)	OR: 1.14 (0.46 to 2.83)	0.09 to 15.12	0.93 (0.54 to 2.12)	90% (83% to 95%)	62.69 (6); <0.0001
**Concomitant CMD therapy**							
Quality of life: 12 item Short-Form Mental Health Survey (Murphy, 2009[137])	1	MD: 0.70 (-1.56 to 2.96)	N/A	N/A	N/A	N/A	N/A
Quality of life: 36 item Short-Form Mental Health Survey	4	MD: -1.49 (-1.84 to -1.14)	MD: -1.08 (-2.29 to 0.12)	-4.39 to 2.22	0.59 (0.00 to 2.55)	8% (0% to 86%)	3.25 (3); 0.35
Quality of life: 12 item Short-Form Physical Health Survey (Murphy, 2009[137])	1	MD: 1.70 (-0.69 to 4.09)	N/A	N/A	N/A	N/A	N/A
Quality of life: 36 item Short-Form Physical Health Survey	4	MD: -1.42 (-1.83 to -1.02)	MD: -0.94 (-2.32 to 0.45)	-4.70 to 2.83	0.68 (0.00 to 2.88)	16% (0% to 87%)	3.58 (3); 0.31
Quality of life: European Quality of Life-5 Dimensions (EQ-5D)	2	MD: -0.00 (-0.01 to 0.01)	MD: -0.00 (-0.02 to 0.02)	N/A	0	0%	0.19 (1); 0.66
Quality of life: St George's Respiratory Questionnaire	4	MD: -4.42 (-6.20 to -2.64)	MD: -4.40 (-8.66 to -0.13)	-10.71 to 1.92	0.96 (0.00 to >10.00)	39% (0% to 79%)	4.91 (3); 0.18
Quality of life: Minnesota Living with Heart Failure Questionnaire (de la Porte, 2007[69])	1	MD: -4.30 (-10.04 to 1.44)	N/A	N/A	N/A	N/A	N/A
Quality of care	3	OR: 1.94 (1.45 to 2.59)	OR: 1.94	0.29 to 12.77	0	0%	0.05 (2); 0.98

			(1.76 to 2.13)			(0% to 90%)	

Appendix 19: Meta-regression for Each Outcome/Scale Comparing Any KT Intervention vs Usual Care

Publication Year

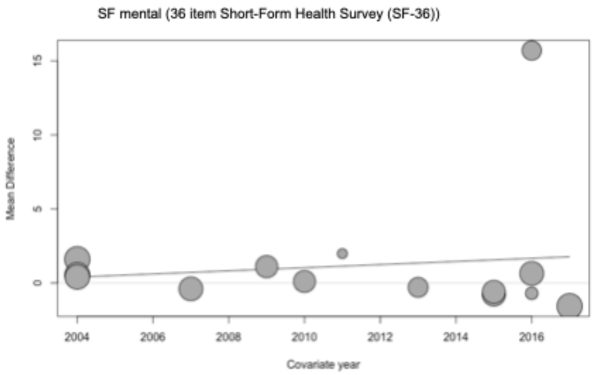

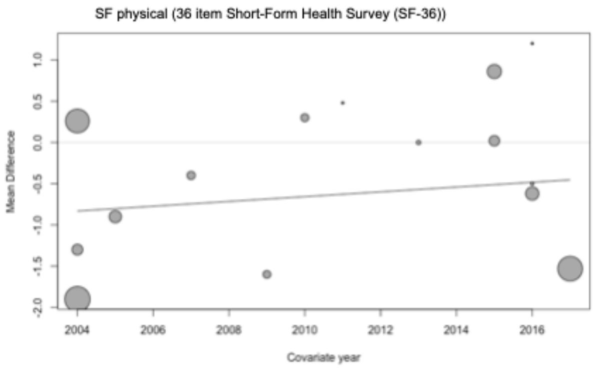

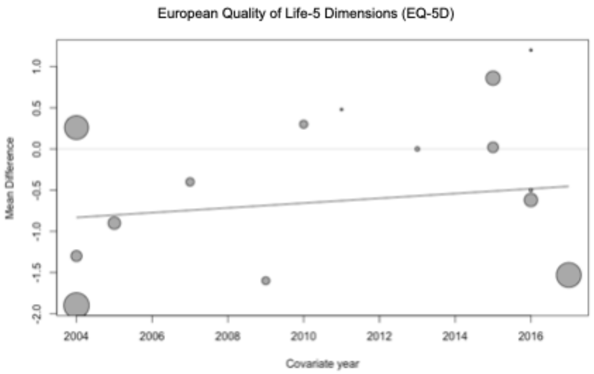

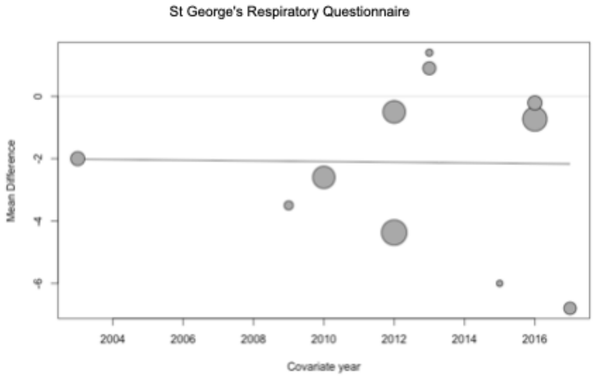

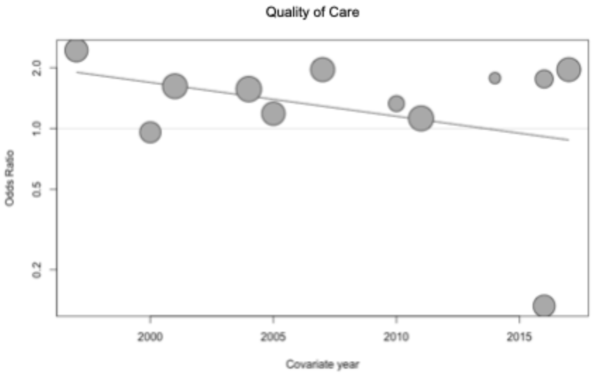

Outcome	No of studies	Regression coefficient (95% CI) [MD/Log OR]	τ	I^2
Publication Year				
Quality of life: 36 item Short-Form Mental Health Survey	14	MD: 0.11<br>(-0.40 to 0.61)	3.82	98%
Quality of life: 36 item Short-Form Physical Health Survey	15	MD: 0.03<br>(-0.07 to 0.13)	0.87	66%
Quality of life: European Quality of Life-5 Dimensions (EQ-5D)	15	MD: 0.00<br>(-0.01 to 0.02)	0.06	84%
Quality of life: St George's Respiratory Questionnaire	11	MD: -0.01<br>(-0.48 to 0.46)	1.64	48%
Quality of care	12	LogOR: -0.04<br>(-0.11 to 0.04)	0.68	89%

Low Quality of Life Baseline

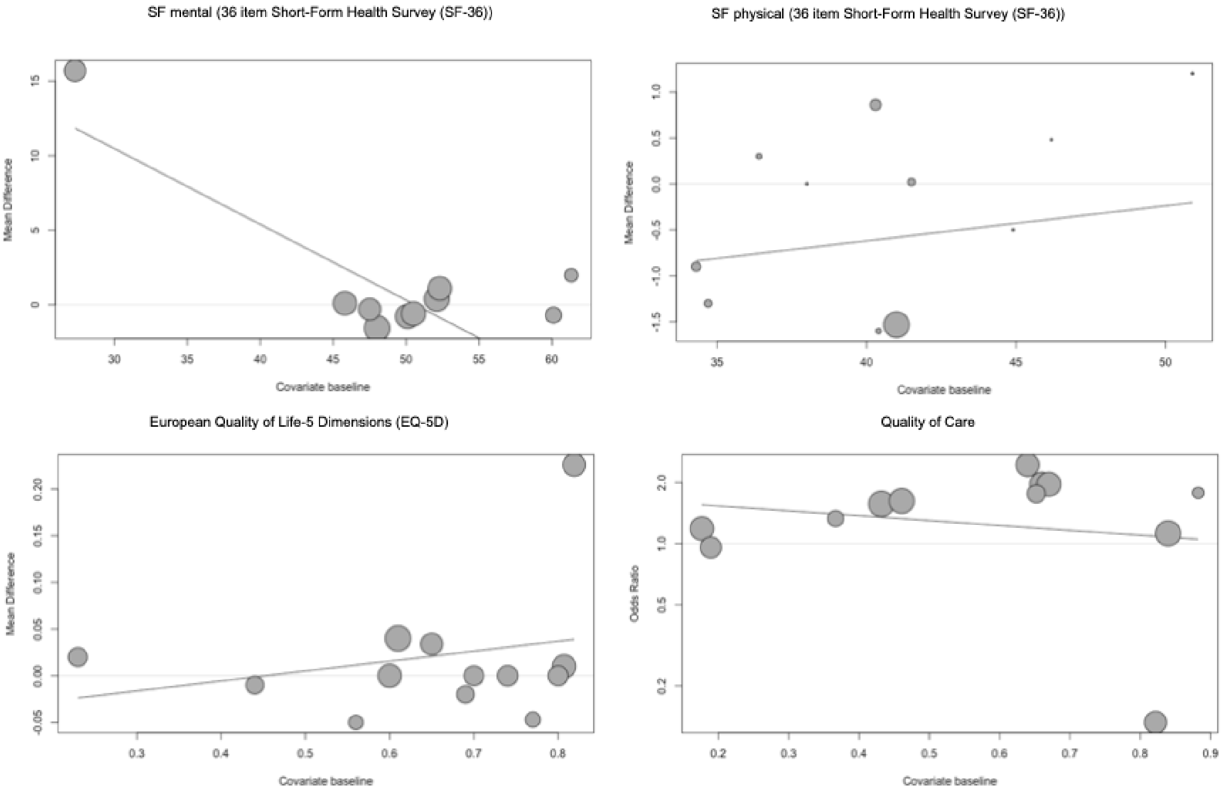

Outcome	No of studies	Regression coefficient (95% CI) [MD/Log OR]	τ	I^2
Low Quality of Life Baseline				
Quality of life: 36 item Short-Form Mental Health Survey	10	MD: -0.51<br>(-0.80 to -0.22)	2.11	83%
Quality of life: 36 item Short-Form Physical Health Survey	11	MD: 0.04<br>(-0.18 to 0.25)	0.71	35%
Quality of life: European Quality of Life-5 Dimensions (EQ-5D)	13	MD: 0.11<br>(-0.17 to 0.38)	0.06	84%
Quality of care	12	Log OR: -0.55<br>(-2.81 to 1.71)	0.73	90%

Mean participant age

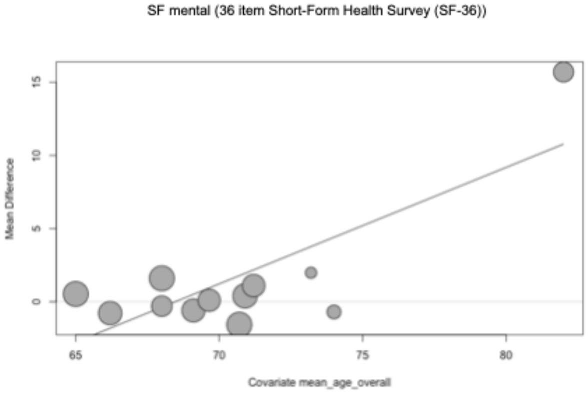

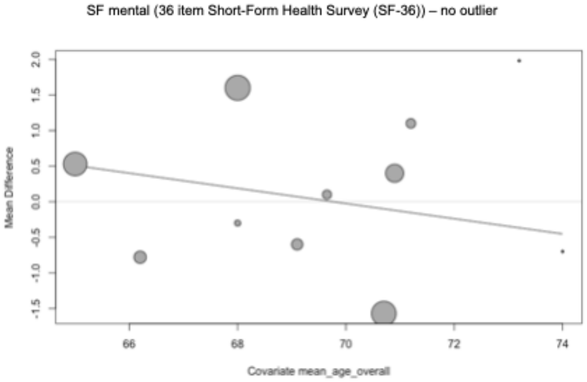

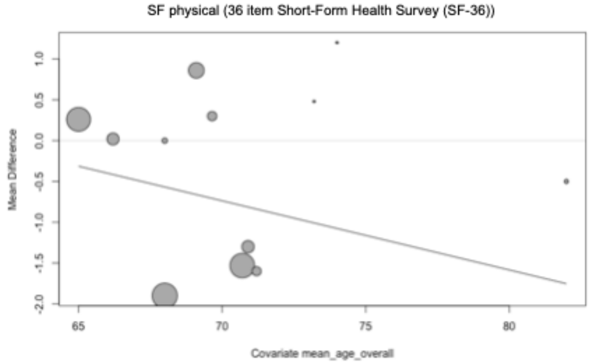

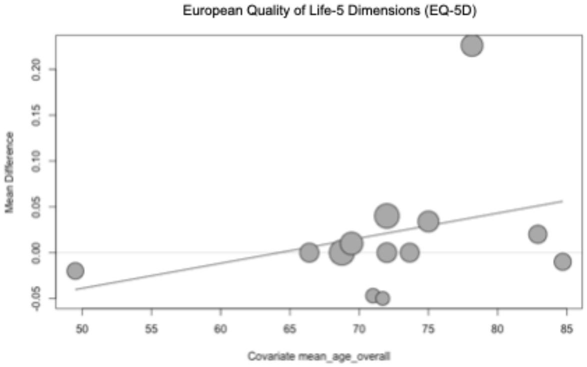

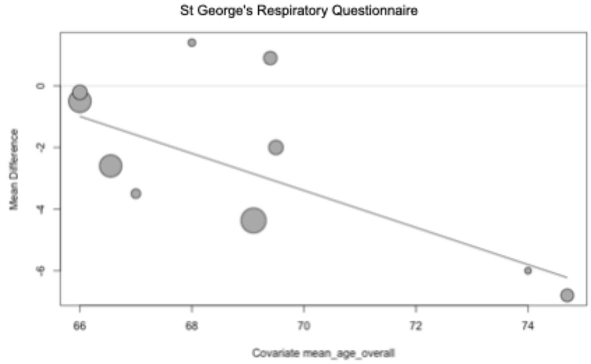

Outcome	No of studies	Regression coefficient (95% CI) [MD/Log OR]	τ	I^2
Mean Participant Age				
Quality of life: 36 item Short-Form Mental Health Survey	12	MD: 0.80 (0.35to 1.24)	2.37	97%
Quality of life: 36 item Short-Form Mental Health Survey (Excluding Outlier)	11	MD: -0.11 (-0.45 to 0.23)	1.07	86%
Quality of life: 36 item Short-Form Physical Health Survey	12	MD: -0.08 (-0.31 to 0.14)	0.90	75%
Quality of life: European Quality of Life-5 Dimensions (EQ-5D)	13	MD: 0.00 (0.00 to 0.01)	0.06	90%
Quality of life: St George's Respiratory Questionnaire	10	MD: -0.60 (-1.15 to -0.06)	0.74	14%

References

1. WHO. Noncommunicable diseases 2022 [Available from: https://www.who.int/news-room/fact-sheets/detail/noncommunicable-diseases.
2. Tricco AC, Moore JE, Beben N, Brownson RC, Chambers DA, Dolovich LR, et al. Sustaining knowledge translation interventions for chronic disease management in older adults: protocol for a systematic review and network meta-analysis. Syst Rev. 2018;7(1):140.
3. CADTH. Grey matters: A tool for searching health-related grey literature. Ottawa2022 [Available from: https://greymatters.cadth.ca.
4. Tricco AC, Ashoor HM, Cardoso R, MacDonald H, Cogo E, Kastner M, et al. Sustainability of knowledge translation interventions in healthcare decision-making: a scoping review. Implement Sci. 2016;11:55.
5. (EPOC) EPaOoC. EPOC Taxonomy [Available from: http://epoc.cochrane.org/epoc-taxonomy.
6. Presseau J, Ivers NM, Newham JJ, Knittle K, Danko KJ, Grimshaw JM. Using a behaviour change techniques taxonomy to identify active ingredients within trials of implementation interventions for diabetes care. Implement Sci. 2015;10:55.
7. Reichard P, Pihl M, Rosenqvist U, Sule J. Complications in IDDM are caused by elevated blood glucose level: the Stockholm Diabetes Intervention Study (SDIS) at 10-year follow up. Diabetologia. 1996;39(12):1483-8.
8. Cochrane EPOC Risk of Bias tool [Available from: http://epoc.cochrane.org/sites/epoc.cochrane.org/files/uploads/Risk%20of%20Bias%2005-01-2009.doc.
9. Sterne JA, Sutton AJ, Ioannidis JP, Terrin N, Jones DR, Lau J, et al. Recommendations for examining and interpreting funnel plot asymmetry in meta-analyses of randomised controlled trials. BMJ. 2011;343.
10. Higgins J, Thomas J, Chandler J, Cumpston M, Li T, Page M, et al. Chapter 6: Choosing effect measures and computing estimates of effect. In: Higgins J, Li T, Deeks J, editors. Cochrane Handbook for Systematic Reviews of Interventions. version 6.3 ed. Cochrane: (updated February 2022); 2022.
11. Higgins J, Thomas J, Chandler J, Cumpston M, Li T, Page M, et al. Cochrane Handbook for Systematic Reviews of Interventions. version 6.1 ed. Higgins J, Li T, Deeks J, editors. Cochrane: (updated September 2020); 2020.
12. Higgins JP, Thomas J, Chandler J, Cumpston M, Li T, Page MJ, et al. Cochrane handbook for systematic reviews of interventions: John Wiley & Sons; 2019.
13. Veroniki AA, Jackson D, Viechtbauer W, Bender R, Bowden J, Knapp G, et al. Methods to estimate the between-study variance and its uncertainty in meta-analysis. Res Synth Methods. 2016;7(1):55-79.
14. Hartung J, Knapp G. A refined method for the meta-analysis of controlled clinical trials with binary outcome. Stat Med. 2001;20(24):3875-89.
15. Sidik K, Jonkman JN. A simple confidence interval for meta-analysis. Stat Med. 2002;21(21):3153-9.
16. Higgins JP, Thompson SG, Deeks JJ, Altman DG. Measuring inconsistency in meta-analyses. BMJ. 2003;327(7414):557-60.
17. Huedo-Medina TB, Sanchez-Meca J, Marin-Martinez F, Botella J. Assessing heterogeneity in meta-analysis: Q statistic or I2 index? Psychol Methods. 2006;11(2):193-206.

18. Veroniki AA, Jackson D, Viechtbauer W, Bender R, Knapp G, Kuss O, et al. Recommendations for quantifying the uncertainty in the summary intervention effect and estimating the between-study heterogeneity variance in random-effects meta-analysis. In: Chandler J, McKenzie J, Boutron I, Welch V, editors.: Cochrane Methods. Cochrane Database of Systematic Reviews; 2015.
19. DerSimonian R, Laird N. Meta-analysis in clinical trials. Control Clin Trials. 1986;7(3):177-88.
20. Viechtbauer W. Confidence intervals for the amount of heterogeneity in meta-analysis. Stat Med. 2007;26(1):37-52.
21. Rover C, Knapp G, Friede T. Hartung-Knapp-Sidik-Jonkman approach and its modification for random-effects meta-analysis with few studies. BMC Med Res Methodol. 2015;15:99.
22. Veroniki AA. Random-effects meta-analysis methods in RevMan2022.
23. Schwarzer G. meta: An R package for meta-analysis. R news. 2007;7(3):40-5.
24. Veroniki AA, Jackson D, Bender R, Kuss O, Langan D, Higgins JPT, et al. Methods to calculate uncertainty in the estimated overall effect size from a random-effects meta-analysis. Res Synth Methods. 2019;10(1):23-43.
25. Sadeghirad B, Foroutan F, Zoratti MJ, Busse JW, Brignardello-Petersen R, Guyatt G, et al. Theory and practice of Bayesian and frequentist frameworks for network meta-analysis. BMJ Evid Based Med. 2022.
26. Vickrey B, Mittman B, Connor K, Pearson M, Della Penna R, Ganiats T, et al. The effect of a disease management intervention on quality and outcomes of dementia care: a randomized, controlled trial. Annals of Internal Medicine. 2006;145(10

):713-26.
27. Goderis G, Borgermans L, Grol R, Van Den Broeke C, Boland B, Verbeke G, et al. Start improving the quality of care for people with type 2 diabetes through a general practice support program: a cluster randomized trial. Diabetes Res Clin Pract. 2010;88(1):56-64.
28. Heisler M, Hofer TP, Schmittdiel JA, Selby JV, Klamerus ML, Bosworth HB, et al. Improving blood pressure control through a clinical pharmacist outreach program in patients with diabetes mellitus in 2 high-performing health systems: the adherence and intensification of medications cluster randomized, controlled pragmatic trial. Circulation. 2012;125(23):2863-72.
29. Schraeder C, Fraser C, Clark I, Newcomer R, Stoll J, Krock C, et al. The effect of primary care management on lipids testing and LDL-C control of elderly patients with comorbidities. Prof Case Manag. 2009;14(2):84-95.
30. Shea S, Weinstock RS, Starren J, Teresi J, Palmas W, Field L, et al. A randomized trial comparing telemedicine case management with usual care in older, ethnically diverse, medically underserved patients with diabetes mellitus. Journal of the American Medical Informatics Association. 2006;13(1):40-51.
31. Kiefe CI, Allison JJ, Williams OD, Person SD, Weaver MT, Weissman NW. Improving quality improvement using achievable benchmarks for physician feedback: a randomized controlled trial. Jama. 2001;285(22):2871-9.
32. Tjia J, Field T, Mazor K, Lemay CA, Kanaan AO, Donovan JL, et al. Dissemination of evidence-based antipsychotic prescribing guidelines to nursing homes: a cluster randomized trial. J Am Geriatr Soc. 2015;63(7):1289-98.

33. de Fine Olivarius N, Beck-Nielsen H, Andreasen AH, Hørder M, Pedersen PA. Randomised controlled trial of structured personal care of type 2 diabetes mellitus. BMJ. 2001;323(7319):970.
34. Eccles MP, Whitty PM, Speed C, Steen IN, Vanoli A, Hawthorne GC, et al. A pragmatic cluster randomised controlled trial of a Diabetes REcall And Management system: the DREAM trial. Implement Sci. 2007;2(1):1-12.
35. Olaiya MT, Kim J, Nelson MR, Srikanth VK, Bladin CF, Gerraty RP, et al. Effectiveness of a shared team approach between nurses and doctors for improved risk factor management in survivors of stroke: a cluster randomized controlled trial. Eur J Neurol. 2017;24(7):920-8.
36. Chen S, Conwell Y, He J, Lu N, Wu J. Depression care management for adults older than 60 years in primary care clinics in urban China: a cluster-randomised trial. Lancet Psychiat. 2015;2(4):332-9.
37. Kennedy A, Bower P, Reeves D, Blakeman T, Bowen R, Chew-Graham C, et al. Implementation of self management support for long term conditions in routine primary care settings: cluster randomised controlled trial. BMJ. 2013;346.
38. Moy ML, Martinez CH, Kadri R, Roman P, Holleman RG, Kim HM, et al. Long-term effects of an internet-mediated pedometer-based walking program for chronic obstructive pulmonary disease: randomized controlled trial. J Med Internet Res. 2016;18(8):e5622.
39. Trofimov EE, Poskrebysheva A, Gurskaya A, Novikova A, editors. Therapeutic education for patients with heart failure: effect on medication adherence and clinical outcomes. Eur J Heart Fail; 2015.
40. Miller W, Rollnick S. Motivational interviewing: preparing people for change, edn. New York Guilford; 2002.
41. Palacio AM, Uribe C, Hazel-Fernandez L, Li H, Tamariz LJ, Garay SD, et al. Can phone-based motivational interviewing improve medication adherence to antiplatelet medications after a coronary stent among racial minorities? A randomized trial. J Gen Intern Med. 2015;30(4):469-75.
42. Dracup K, Moser DK, Pelter MM, Nesbitt TS, Southard J, Paul SM, et al. Randomized, controlled trial to improve self-care in patients with heart failure living in rural areas. Circulation. 2014;130(3):256-64.
43. Ansari M, Shlipak MG, Heidenreich PA, Van Ostaeyen D, Pohl EC, Browner WS, et al. Improving guideline adherence: a randomized trial evaluating strategies to increase β-blocker use in heart failure. Circulation. 2003;107(22):2799-804.
44. Baker R, Fraser RC, Stone M, Lambert P, Stevenson K, Shiels C. Randomised controlled trial of the impact of guidelines, prioritized review criteria and feedback on implementation of recommendations for angina and asthma. Br J Gen Pract. 2003;53(489):284-91.
45. Batchelor FA, Hill KD, Mackintosh SF, Said CM, Whitehead CH. Effects of a multifactorial falls prevention program for people with stroke returning home after rehabilitation: a randomized controlled trial. Arch Phys Med Rehabil. 2012;93(9):1648-55.
46. Beck A, Scott J, Williams P, Robertson B, Jackson D, Gade G, et al. A randomized trial of group outpatient visits for chronically ill older HMO members: the Cooperative Health Care Clinic. J Am Geriatr Soc. 1997;45(5):543-9.
47. Beck CA, Beran DB, Biglan KM, Boyd CM, Dorsey ER, Schmidt PN, et al. National randomized controlled trial of virtual house calls for Parkinson disease. Neurology. 2017;89(11):1152-61.

48. Bekelman DB, Plomondon ME, Carey EP, Sullivan MD, Nelson KM, Hattler B, et al. Primary results of the patient-centered disease management (PCDM) for heart failure study: a randomized clinical trial. JAMA Intern Med. 2015;175(5):725-32.
49. Benzo R, Vickers K, Novotny PJ, Tucker S, Hoult J, Neuenfeldt P, et al. Health coaching and chronic obstructive pulmonary disease rehospitalization. A randomized study. Am J Respir Crit Care Med. 2016;194(6):672-80.
50. Blue L, Lang E, McMurray JJ, Davie AP, McDonagh TA, Murdoch DR, et al. Randomised controlled trial of specialist nurse intervention in heart failure. BMJ. 2001;323(7315):715-8.
51. Bohingamu Mudiyanselage S, Stevens J, Watts JJ, Toscano J, Kotowicz MA, Steinfort CL, et al. Personalised telehealth intervention for chronic disease management: a pilot randomised controlled trial. J Telemed Telecare. 2019;25(6):343-52.
52. Böhm M, Drexler H, Oswald H, Rybak K, Bosch R, Butter C, et al. Fluid status telemedicine alerts for heart failure: a randomized controlled trial. European Heart Journal. 2016;37(41):3154-63.
53. Team CPMMPE. The MEDMAN study: a randomized controlled trial of community pharmacy-led medicines management for patients with coronary heart disease. Family Practice. 2007;24(2):189-200.
54. Bosanquet K, Adamson J, Atherton K, Bailey D, Baxter C, Beresford-Dent J, et al. CollAborative care for Screen-Positive EldeRs with major depression (CASPER plus): a multicentred randomised controlled trial of clinical effectiveness and cost-effectiveness. Health Technol Assess. 2017;21(67):1.
55. Bourbeau J, Julien M, Maltais F, Rouleau M, Beaupré A, Bégin R, et al. Reduction of hospital utilization in patients with chronic obstructive pulmonary disease: a disease-specific self-management intervention. Arch Intern Med. 2003;163(5):585-91.
56. Boyne JJ, Vrijhoef HJ, Crijns HJ, De Weerd G, Kragten J, Gorgels AP, et al. Tailored telemonitoring in patients with heart failure: results of a multicentre randomized controlled trial. Eur J Heart Fail. 2012;14(7):791-801.
57. Bruce ML, Ten Have TR, Reynolds III CF, Katz II, Schulberg HC, Mulsant BH, et al. Reducing suicidal ideation and depressive symptoms in depressed older primary care patients: a randomized controlled trial. Jama. 2004;291(9):1081-91.
58. Bruce ML, Raue PJ, Reilly CF, Greenberg RL, Meyers BS, Banerjee S, et al. Clinical effectiveness of integrating depression care management into medicare home health: the Depression CAREPATH Randomized trial. JAMA Intern Med. 2015;175(1):55-64.
59. Bucknall C, Miller G, Lloyd S, Cleland J, McCluskey S, Cotton M, et al. Glasgow supported self-management trial (GSuST) for patients with moderate to severe COPD: randomised controlled trial. BMJ. 2012;344.
60. Burns R, Nichols LO, Graney MJ, Cloar FT. Impact of continued geriatric outpatient management on health outcomes of older veterans. Arch Intern Med. 1995;155(12):1313-8.
61. Burns R, Nichols LO, Martindale-Adams J, Graney MJ, Lummus A. Primary care interventions for dementia caregivers: 2-year outcomes from the REACH study. Gerontologist. 2003;43(4):547-55.
62. Callahan CM, Boustani MA, Unverzagt FW, Austrom MG, Damush TM, Perkins AJ, et al. Effectiveness of collaborative care for older adults with Alzheimer disease in primary care: a randomized controlled trial. Jama. 2006;295(18):2148-57.

63. Campbell NC, Ritchie L, Thain J, Deans H, Rawles J, Squair J. Secondary prevention in coronary heart disease: a randomised trial of nurse led clinics in primary care. Heart. 1998;80(5):447-52.
64. Chi C, Chen H. DAILY-BASED SELF-MANAGEMENT FOR NON-HOSPITALISED HEART FAILURE PATIENTS IMPROVE PROGNOSIS. Heart. 2012;98(Suppl 2):E231-E2.
65. Ciaschini PM, Straus SE, Dolovich LR, Goeree RA, Leung KM, Woods CR, et al. Community based intervention to optimize osteoporosis management: randomized controlled trial. BMC Geriatr. 2010;10(1):1-7.
66. Cleveringa FG, Gorter KJ, Van Den Donk M, Rutten GE. Combined task delegation, computerized decision support, and feedback improve cardiovascular risk for type 2 diabetic patients: a cluster randomized trial in primary care. Diabetes Care. 2008;31(12):2273-5.
67. Coleman E, Grothaus L, Sandhu N, Wagner E. Chronic care clinics: a randomized controlled trial of a new model of primary care for frail older adults. J Am Geriatr Soc. 1999;47(7):775-83.
68. Coull AJ, Taylor VH, Elton R, Murdoch PS, Hargreaves AD. A randomised controlled trial of senior Lay Health Mentoring in older people with ischaemic heart disease: The Braveheart Project. Age Ageing 2004;33(4):348-54.
69. de la Porte PW, Lok DJ, van Veldhuisen DJ, van Wijngaarden J, Cornel JH, Zuithoff NP, et al. Added value of a physician-and-nurse-directed heart failure clinic: results from the Deventer–Alkmaar heart failure study. Heart. 2007;93(7):819-25.
70. De Lusignana S, Gallagher H, Jones S, Chan T, Van Vlymen J, Tahir A, et al. Audit-based education lowers systolic blood pressure in chronic kidney disease: the Quality Improvement in CKD (QICKD) trial results. Kidney Int. 2013;84(3):609-20.
71. DeBusk RF, Miller NH, Parker KM, Bandura A, Kraemer HC, Cher DJ, et al. Care management for low-risk patients with heart failure: a randomized, controlled trial. Ann Intern Med. 2004;141(8):606-13.
72. Del Sindaco D, Pulignano G, Minardi G, Apostoli A, Guerrieri L, Rotoloni M, et al. Two-year outcome of a prospective, controlled study of a disease management programme for elderly patients with heart failure. J Cardiovasc Med 2007;8(5):324-9.
73. DeVore AD, Cox M, Heidenreich PA, Fonarow GC, Yancy CW, Eapen ZJ, et al. Cluster-randomized trial of personalized site performance feedback in get with the guidelines-heart failure. Circ Cardiovasc Qual Outcomes. 2015;8(4):421-7.
74. Döpp CM, Graff MJ, Teerenstra S, Olde Rikkert MG, Nijhuis–van der Sanden MW, Vernooij-Dassen MJ. Effectiveness of a training package for implementing a community-based occupational therapy program in dementia: a cluster randomized controlled trial. Clin Rehabil. 2015;29(10):974-86.
75. Dunagan WC, Littenberg B, Ewald GA, Jones CA, Emery VB, Waterman BM, et al. Randomized trial of a nurse-administered, telephone-based disease management program for patients with heart failure. J Card Fail. 2005;11(5):358-65.
76. Eckert K, Schrader G, Wilkinson D, Askew D, Dick M, Wade T, et al. Detection and management of depression in patients with chronic heart disease: the TAKE heart in primary care cluster randomised controlled trial. Heart Lung Circ. 2010;19:S240-S1.
77. Ell K, Unützer J, Aranda M, Gibbs NE, Lee P-J, Xie B. Managing depression in home health care: a randomized clinical trial. Home Health Care Serv Q. 2007;26(3):81-104.

78. Fan VS, Gaziano JM, Lew R, Bourbeau J, Adams SG, Leatherman S, et al. A comprehensive care management program to prevent chronic obstructive pulmonary disease hospitalizations: a randomized, controlled trial. Ann Intern Med. 2012;156(10):673-83.
79. Federman AD, O'Conor R, Mindlis I, Hoy-Rosas J, Hauser D, Lurio J, et al. Effect of a self-management support intervention on asthma outcomes in older adults: the SAMBA study randomized clinical trial. JAMA Intern Med. 2019;179(8):1113-21.
80. Fihn SD, McDonell MB, Diehr P, Anderson SM, Bradley KA, Au DH, et al. Effects of sustained audit/feedback on self-reported health status of primary care patients. Am J Med. 2004;116(4):241-8.
81. Fihn SD, Bucher JB, McDonell M, Diehr P, Rumsfeld JS, Doak M, et al. Collaborative care intervention for stable ischemic heart disease. Arch Intern Med. 2011;171(16):1471-9.
82. Forster A, Young J. Specialist nurse support for patients with stroke in the community: a randomised controlled trial. BMJ. 1996;312(7047):1642-6.
83. Forster A, Young J, Chapman K, Nixon J, Patel A, Holloway I, et al. Cluster randomized controlled trial: clinical and cost-effectiveness of a system of longer-term stroke care. Stroke. 2015;46(8):2212-9.
84. Tremont G, Duncan Davis J, Bishop DS, Fortinsky RH. Telephone-delivered psychosocial intervention reduces burden in dementia caregivers. Dementia. 2008;7(4):503-20.
85. Freund T, Peters-Klimm F, Boyd CM, Mahler C, Gensichen J, Erler A, et al. Medical assistant–based care management for high-risk patients in small primary care practices: a cluster randomized clinical trial. Ann Intern Med. 2016;164(5):323-30.
86. Galbreath AD, Krasuski RA, Smith B, Stajduhar KC, Kwan MD, Ellis R, et al. Long-term healthcare and cost outcomes of disease management in a large, randomized, community-based population with heart failure. Circulation. 2004;110(23):3518-26.
87. Gallagher RA, Miller C, Cronan TA, Groessl E. Gender differences in participation and responsiveness to a health intervention for older Americans. J Womens Health. 1997;25(3):63-81.
88. Gaugler JE, Roth DL, Haley WE, Mittelman MS. Can counseling and support reduce Alzheimer's caregivers' burden and depressive symptoms during the transition to institutionalization? Results from the NYU caregiver intervention study. J Am Geriatr Soc. 2008;56(3):421.
89. Gellis ZD, Kenaley B, McGinty J, Bardelli E, Davitt J, Ten Have T. Outcomes of a telehealth intervention for homebound older adults with heart or chronic respiratory failure: a randomized controlled trial. Gerontologist. 2012;52(4):541-52.
90. Graven C, Brock K, Hill KD, Cotton S, Joubert L. First year after stroke: an integrated approach focusing on participation goals aiming to reduce depressive symptoms. Stroke. 2016;47(11):2820-7.
91. Haerter M, Dirmaier J, Dwinger S, Kriston L, Herbarth L, Siegmund-Schultze E, et al. Effectiveness of telephone-based health coaching for patients with chronic conditions: a randomised controlled trial. PloS One. 2016;11(9):e0161269.
92. Hendriks JM, De Wit R, Crijns HJ, Vrijhoef HJ, Prins MH, Pisters R, et al. Nurse-led care vs. usual care for patients with atrial fibrillation: results of a randomized trial of integrated chronic care vs. routine clinical care in ambulatory patients with atrial fibrillation. Eur Heart J. 2012;33(21):2692-9.

93. Hernández C, Alonso A, Garcia-Aymerich J, Serra I, Marti D, Rodriguez-Roisin R, et al. Effectiveness of community-based integrated care in frail COPD patients: a randomised controlled trial. NPJ Prim Care Respir Med. 2015;25(1):1-6.
94. Hetlevik I, Holmen J, Krüger Ø, Kristensen P, Iversen H, Furuseth K. Implementing clinical guidelines in the treatment of diabetes mellitus in general practice: Evaluation of effort, process, and patient outcome related to implementation of a computer-based decision support system. Int J Technol Assess Health Care. 2000;16(1):210-27.
95. Hogg W, Lemelin J, Dahrouge S, Liddy C, Armstrong CD, Legault F, et al. Randomized controlled trial of anticipatory and preventive multidisciplinary team care: for complex patients in a community-based primary care setting. Can Fam Physician. 2009;55(12):e76-e85.
96. Holbrook A, Pullenayegum E, Thabane L, Troyan S, Foster G, Keshavjee K, et al. Shared electronic vascular risk decision support in primary care: Computerization of Medical Practices for the Enhancement of Therapeutic Effectiveness (COMPETE III) randomized trial. Arch Intern Med. 2011;171(19):1736-44.
97. Holm T, Lassen J, Husted S, Christensen P, Heickendorff L. A randomized controlled trial of shared care versus routine care for patients receiving oral anticoagulant therapy. J Intern Med. 2002;252(4):322-31.
98. Holt TA, Thorogood M, Griffiths F, Munday S, Friede T, Stables D. Automated electronic reminders to facilitate primary cardiovascular disease prevention: randomised controlled trial. Br J Gen Pract. 2010;60(573):e137-e43.
99. Hughes S, Weaver F, Giobbie-Hurder A, Manheim L, Henderson W, Kubal J, et al. Department of Veterans Affairs Cooperative Study Group on Home-Based Primary Care Effectiveness of team-managed home-based primary care: A randomized multicenter trial. Jama. 2000;284(22):2877-85.
100. Hunger M, Kirchberger I, Holle R, Seidl H, Kuch B, Wende R, et al. Does nurse-based case management for aged myocardial infarction patients improve risk factors, physical functioning and mental health? The KORINNA trial. Eur J Prev Cardiol. 2015;22(4):442-50.
101. Irewall A-L, Ögren J, Bergström L, Laurell K, Söderström L, Mooe T. Nurse-led, telephone-based, secondary preventive follow-up after stroke or transient ischemic attack improves blood pressure and LDL cholesterol: results from the first 12 months of the randomized, controlled NAILED stroke risk factor trial. PloS one. 2015;10(10):e0139997.
102. Jaarsma T, Van Der Wal M, Lesman-Leegte I, Luttik M, Hogenhuis J, Veeger N, et al. Coordinating Study Evaluating Outcomes of Advising and Counseling in Heart Failure (COACH) Investigators. Effect of moderate or intensive disease management program on outcome in patients with heart failure: Coordinating Study Evaluating Outcomes of Advising and Counseling in Heart Failure (COACH). Arch Intern Med. 2008;168(3):316-24.
103. Joling KJ, van Marwijk HW, Smit F, van der Horst HE, Scheltens P, van de Ven PM, et al. Does a family meetings intervention prevent depression and anxiety in family caregivers of dementia patients? A randomized trial. PloS One. 2012;7(1):e30936.
104. Karhula T, Vuorinen A-L, Rääpysjärvi K, Pakanen M, Itkonen P, Tepponen M, et al. Telemonitoring and mobile phone-based health coaching among Finnish diabetic and heart disease patients: randomized controlled trial. J Med Internet Res. 2015;17(6):e4059.
105. Kennedy CC, Ioannidis G, Thabane L, Adachi JD, Marr S, Giangregorio LM, et al. Successful knowledge translation intervention in long-term care: final results from the vitamin D and osteoporosis study (ViDOS) pilot cluster randomized controlled trial. Trials. 2015;16(1):1-11.

106. Khdour MR, Kidney JC, Smyth BM, McElnay JC. Clinical pharmacy-led disease and medicine management programme for patients with COPD. Br J Clin Pharmacol. 2009;68(4):588-98.
107. Kim KB, Han H-R, Huh B, Nguyen T, Lee H, Kim MT. The effect of a community-based self-help multimodal behavioral intervention in Korean American seniors with high blood pressure. Am J Hypertens. 2014;27(9):1199-208.
108. Ko FW, Cheung N, Rainer TH, Lum C, Wong I, Hui DS. Comprehensive care programme for patients with chronic obstructive pulmonary disease: a randomised controlled trial. Thorax. 2017;72(2):122-8.
109. Kruis AL, Boland MR, Assendelft WJ, Gussekloo J, Tsiachristas A, Stijnen T, et al. Effectiveness of integrated disease management for primary care chronic obstructive pulmonary disease patients: results of cluster randomised trial. BMJ. 2014;349.
110. Krum H, Forbes A, Yallop J, Driscoll A, Croucher J, Chan B, et al. Telephone support to rural and remote patients with heart failure: the Chronic Heart Failure Assessment by Telephone (CHAT) study. Cardiovasc Ther. 2013;31(4):230-7.
111. Kurz A, Wagenpfeil S, Hallauer J, Schneider-Schelte H, Jansen S, Group AS. Evaluation of a brief educational program for dementia carers: The AENEAS Study. Int J Geriatr Psychiatry. 2010;25(8):861-9.
112. Kalter-Leibovici O, Freimark D, Freedman LS, Kaufman G, Ziv A, Murad H, et al. Disease management in the treatment of patients with chronic heart failure who have universal access to health care: a randomized controlled trial. BMC Med. 2017;15(1):1-13.
113. Leveille SG, Wagner EH, Davis C, Grothaus L, Wallace J, LoGerfo M, et al. Preventing disability and managing chronic illness in frail older adults: a randomized trial of a community-based partnership with primary care. J Am Geriatr Soc. 1998;46(10):1191-8.
114. Leventhal ME, Denhaerynck K, Brunner-La Rocca HP, Burnand B, Conca-Zeller A, Bernasconi A, et al. Swiss Interdisciplinary Management Programme for Heart Failure (SWIM-HF): a randomised controlled trial study of an outpatient inter-professional management programme for heart failure patients in Switzerland. Swiss Med Wkly. 2011;141(0910).
115. Levine DA, Funkhouser EM, Houston TK, Gerald JK, Johnson-Roe N, Allison JJ, et al. Improving Care After Myocardial Infarction Using a 2-Year Internet-Delivered Intervention: The Department of Veterans Affairs Myocardial Infarction–Plus Cluster-Randomized Trial. Arch Intern Med. 2011;171(21):1910-7.
116. Li X, Zhou Q, Zou F, Wu L, Chen H, Liu Z. Effectiveness of systematic self-management education on blood sugar level of patients in the community with type 2 diabetes. Zhong Nan Da Xue Xue Bao Yi Xue Ban. 2012;37(4):355-8.
117. Licskai C, Ferrone M, Malus N, Stitt L, O'Callahan T, Roberts Z, et al. COPD collaborative self-management in primary care: A randomized controlled trial. Eur Respiratory Soc; 2016.
118. Lin H-W, Lin C-H, Chang C-K, Chou C-Y, Yu I-W, Lin C-C, et al. Economic outcomes of pharmacist-physician medication therapy management for polypharmacy elderly: a prospective, randomized, controlled trial. J Formos Med Assoc. 2018;117(3):235-43.
119. Liu W, Wang C, Lin H, Lin S, Lee K, Lo Y, et al. Efficacy of a cell phone-based exercise programme for COPD. Eur Respir J. 2008;32(3):651-9.
120. López Cabezas C, Falces Salvador C, Cubí Quadrada D, Arnau Bartés A, Ylla Bore M, Muro Perea N, et al. Randomized clinical trial of a postdischarge pharmaceutical care program vs regular follow-up in patients with heart failure. Farmacia Hospitalaria. 2006;30(6):328-42.

121. Lowrie R, Lloyd SM, McConnachie A, Morrison J. A cluster randomised controlled trial of a pharmacist-led collaborative intervention to improve statin prescribing and attainment of cholesterol targets in primary care. Plos One. 2014;9(11):e113370.
122. Machline-Carrion MJ, Soares RM, Damiani LP, Campos VB, Sampaio B, Fonseca FH, et al. Effect of a multifaceted quality improvement intervention on the prescription of evidence-based treatment in patients at high cardiovascular risk in Brazil: the bridge cardiovascular prevention cluster randomized clinical trial. JAMA Cardiol. 2019;4(5):408-17.
123. Mahoney DF, Tarlow BJ, Jones RN. Effects of an automated telephone support system on caregiver burden and anxiety: findings from the REACH for TLC intervention study. Gerontologist. 2003;43(4):556-67.
124. Maltais F, Bourbeau J, Shapiro S, Lacasse Y, Perrault H, Baltzan M, et al. Effects of home-based pulmonary rehabilitation in patients with chronic obstructive pulmonary disease: a randomized trial. Ann Intern Med. 2008;149(12):869-78.
125. Markle-Reid M, Orridge C, Weir R, Browne G, Gafni A, Lewis M, et al. Interprofessional stroke rehabilitation for stroke survivors using home care. Can J Neurol Sci. 2011;38(2):317-34.
126. Markun S, Dishy A, Neuner-Jehle S, Rosemann T, Frei A. The chronic care for wet age related macular degeneration (CHARMED) study: a randomized controlled trial. PLoS One. 2015;10(11):e0143085.
127. McCluskey A, Ada L, Kelly PJ, Middleton S, Goodall S, Grimshaw JM, et al. A behavior change program to increase outings delivered during therapy to stroke survivors by community rehabilitation teams: The Out-and-About trial. Int J Stroke. 2016;11(4):425-37.
128. McElrath M, Myers J, Chan K, Fonda H. Exercise adherence in the elderly: Experience with abdominal aortic aneurysm simple treatment and prevention. J Vasc Nurs. 2017;35(1):12-20.
129. McManus RJ, Mant J, Haque MS, Bray EP, Bryan S, Greenfield SM, et al. Effect of self-monitoring and medication self-titration on systolic blood pressure in hypertensive patients at high risk of cardiovascular disease: the TASMIN-SR randomized clinical trial. Jama. 2014;312(8):799-808.
130. Meeuwsen EJ, Melis RJ, Van Der Aa GC, Golüke-Willemse GA, De Leest BJ, Van Raak FH, et al. Effectiveness of dementia follow-up care by memory clinics or general practitioners: randomised controlled trial. BMJ. 2012;344.
131. Meisinger C, Stollenwerk B, Kirchberger I, Seidl H, Wende R, Kuch B, et al. Effects of a nurse-based case management compared to usual care among aged patients with myocardial infarction: results from the randomized controlled KORINNA study. BMC Geriatr. 2013;13(1):1-10.
132. Mejhert M, Kahan T, Persson H, Edner M. Limited long term effects of a management programme for heart failure. Heart. 2004;90(9):1010-5.
133. Mitchell E, Sullivan F, Grimshaw JM, Donnan PT, Watt G. Improving management of hypertension in general practice: a randomised controlled trial of feedback derived from electronic patient data. Br J Gen Pract. 2005;55(511):94-101.
134. Moher M, Yudkin P, Wright L, Turner R, Fuller A, Schofield T, et al. Cluster randomised controlled trial to compare three methods of promoting secondary prevention of coronary heart disease in primary care. BMJ. 2001;322(7298):1338.
135. Morganroth M, Pape G, Rozenfeld Y, Heffner JE. Multidisciplinary COPD disease management program: impact on clinical outcomes. Postgrad Med. 2016;128(2):239-49.

136. Moriyama M, Nakano M, Kuroe Y, Nin K, Niitani M, Nakaya T. Efficacy of a self-management education program for people with type 2 diabetes: results of a 12 month trial. Jpn J Nurs Sci. 2009;6(1):51-63.
137. Murphy AW, Cupples M, Smith S, Byrne M, Byrne M, Newell J. Effect of tailored practice and patient care plans on secondary prevention of heart disease in general practice: cluster randomised controlled trial. BMJ. 2009;339.
138. Nguyen HQ, Donesky D, Reinke LF, Wolpin S, Chyall L, Benditt JO, et al. Internet-based dyspnea self-management support for patients with chronic obstructive pulmonary disease. J Pain Symptom Manag. 2013;46(1):43-55.
139. Ojeda S, Anguita M, Delgado M, Atienza F, Rus C, Granados AL, et al. Short-and long-term results of a programme for the prevention of readmissions and mortality in patients with heart failure: are effects maintained after stopping the programme? Eur J Heart Fail. 2005;7(5):921-6.
140. Olson KL, Delate T, Rasmussen J, Humphries TL, Merenich JA. Outcomes of patients discharged from pharmacy-managed cardiovascular disease management. Am J Manag Care. 2009;15(8):497-503.
141. Ortiz-Bautista C, Diaz M, Delgado-Nicolas M, Moran-Fernandez L, De Juan-Baguda J, Ponz I, et al., editors. Evaluation of a nurse-led cross intervention program in heart failure. Eur J Heart Fail; 2017: WILEY 111 RIVER ST, HOBOKEN 07030-5774, NJ USA.
142. Ostwald SK, Godwin KM, Cron SG, Kelley CP, Hersch G, Davis S. Home-based psychoeducational and mailed information programs for stroke-caregiving dyads post-discharge: a randomized trial. Disabil Rehabil. 2014;36(1):55-62.
143. Peters-Klimm F, Campbell S, Hermann K, Kunz CU, Müller-Tasch T, Szecsenyi J. Case management for patients with chronic systolic heart failure in primary care: the HICMan exploratory randomised controlled trial. Trials. 2010;11(1):1-14.
144. Piette JD, Striplin D, Marinec N, Chen J, Trivedi RB, Aron DC, et al. A mobile health intervention supporting heart failure patients and their informal caregivers: a randomized comparative effectiveness trial. J Med Internet Res. 2015;17(6):e4550.
145. Pinnock H, Hanley J, McCloughan L, Todd A, Krishan A, Lewis S, et al. Effectiveness of telemonitoring integrated into existing clinical services on hospital admission for exacerbation of chronic obstructive pulmonary disease: researcher blind, multicentre, randomised controlled trial. BMJ. 2013;347.
146. Pols AD, Van Dijk SE, Bosmans JE, Hoekstra T, Van Marwijk HW, Van Tulder MW, et al. Effectiveness of a stepped-care intervention to prevent major depression in patients with type 2 diabetes mellitus and/or coronary heart disease and subthreshold depression: a pragmatic cluster randomized controlled trial. PLoS One. 2017;12(8):e0181023.
147. Rea H, McAuley S, Stewart A, Lamont C, Roseman P, Didsbury P. A chronic disease management programme can reduce days in hospital for patients with chronic obstructive pulmonary disease. Intern Med J. 2004;34(11):608-14.
148. Reiber GE, Au D, McDonell M, Fihn SD. Diabetes quality improvement in Department of Veterans Affairs Ambulatory Care Clinics: a group-randomized clinical trial. Diabetes Care. 2004;27(suppl_2):b61-b8.
149. Rovner BW, Casten RJ, Piersol CV, White N, Kelley M, Leiby BE. Improving glycemic control in African Americans with diabetes and mild cognitive impairment. J Am Geriatr Soc. 2020;68(5):1015-22.

150. Rubenstein LZ, Alessi CA, Josephson KR, Trinidad Hoyl M, Harker JO, Pietruszka FM. A randomized trial of a screening, case finding, and referral system for older veterans in primary care. J Am Geriatr Soc. 2007;55(2):166-74.
151. Saal S, Becker C, Lorenz S, Schubert M, Kuss O, Stang A, et al. Effect of a stroke support service in Germany: a randomized trial. Top Stroke Rehabil. 2015;22(6):429-36.
152. Sackley CM, Walker MF, Burton CR, Watkins CL, Mant J, Roalfe AK, et al. An occupational therapy intervention for residents with stroke related disabilities in UK care homes (OTCH): cluster randomised controlled trial. BMJ. 2015;350.
153. Salinero-Fort MA, Pau EC-dS, Arrieta-Blanco FJ, Abanades-Herranz JC, Martín-Madrazo C, Rodés-Soldevila B, et al. Effectiveness of PRECEDE model for health education on changes and level of control of HbA1c, blood pressure, lipids, and body mass index in patients with type 2 diabetes mellitus. BMC Public Health. 2011;11(1):1-9.
154. Salisbury C, Man M-S, Bower P, Guthrie B, Chaplin K, Gaunt DM, et al. Management of multimorbidity using a patient-centred care model: a pragmatic cluster-randomised trial of the 3D approach. Lancet. 2018;392(10141):41-50.
155. Samus QM, Johnston D, Black BS, Hess E, Lyman C, Vavilikolanu A, et al. A multidimensional home-based care coordination intervention for elders with memory disorders: the maximizing independence at home (MIND) pilot randomized trial. Am J Geriatr Psychiatry. 2014;22(4):398-414.
156. Sánchez-Nieto JM, Andújar-Espinosa R, Bernabeu-Mora R, Hu C, Gálvez-Martínez B, Carrillo-Alcaraz A, et al. Efficacy of a self-management plan in exacerbations for patients with advanced COPD. International journal of chronic obstructive pulmonary disease. 2016;11:1939.
157. Sarkadi A, Rosenqvist U. Experience-based group education in Type 2 diabetes: a randomised controlled trial. Patient Educ Couns. 2004;53(3):291-8.
158. Schäfer I, Kaduszkiewicz H, Mellert C, Löffler C, Mortsiefer A, Ernst A, et al. Narrative medicine-based intervention in primary care to reduce polypharmacy: results from the cluster-randomised controlled trial MultiCare AGENDA. BMJ Open. 2018;8(1):e017653.
159. Schrader G, Cheok F, Hordacre AL, Marker J, Wade V. Effect of psychiatry liaison with general practitioners on depression severity in recently hospitalised cardiac patients: a randomised controlled trial. Med J Aust. 2005;182(6):272-6.
160. Smith BJ, Appleton S, Bennett P, Roberts G, Fante PD, Adam R, et al. The effect of a respiratory home nurse intervention in patients with chronic obstructive pulmonary disease (COPD). Aust N Z J Med. 1999;29(5):718-25.
161. Solomon DH, Iversen MD, Avorn J, Gleeson T, Brookhart MA, Patrick AR, et al. Osteoporosis telephonic intervention to improve medication regimen adherence: a large, pragmatic, randomized controlled trial. Arch Intern Med. 2012;172(6):477-83.
162. Sönnichsen AC, Winkler H, Flamm M, Panisch S, Kowatsch P, Klima G, et al. The effectiveness of the Austrian disease management programme for type 2 diabetes: a cluster-randomised controlled trial. BMC Fam Pract. 2010;11(1):1-10.
163. Stewart S, Carrington MJ, Marwick TH, Davidson PM, Macdonald P, Horowitz JD, et al. Impact of home versus clinic-based management of chronic heart failure: the WHICH?(Which Heart Failure Intervention Is Most Cost-Effective & Consumer Friendly in Reducing Hospital Care) multicenter, randomized trial. J Am Coll Cardiol. 2012;60(14):1239-48.
164. Stewart S, Ball J, Horowitz JD, Marwick TH, Mahadevan G, Wong C, et al. Standard versus atrial fibrillation-specific management strategy (SAFETY) to reduce recurrent admission

and prolong survival: pragmatic, multicentre, randomised controlled trial. Lancet. 2015;385(9970):775-84.
165. Subramanian U, Fihn SD, Weinberger M, Plue L, Smith FE, Udris EM, et al. A controlled trial of including symptom data in computer-based care suggestions for managing patients with chronic heart failure. Am J Med. 2004;116(6):375-84.
166. Suominen MH, Puranen T, Jyväkorpi S, Eloniemi-Sulkava U, Kautiainen H, Siljamäki-Ojansuu U, et al. Nutritional guidance improves nutrient intake and quality of life, and may prevent falls in aged persons with Alzheimer disease living with a spouse (NuAD trial). J Nutr Health Aging. 2015;19(9):901-7.
167. Tomita MR, Tsai B-M, Fisher NM, Kumar NA, Wilding G, Stanton K, et al. Effects of multidisciplinary Internet-based program on management of heart failure. J Multidiscip Healthc. 2009;2:13.
168. Trento M, Gamba S, Gentile L, Grassi G, Miselli V, Morone G, et al. Rethink Organization to iMprove Education and Outcomes (ROMEO) A multicenter randomized trial of lifestyle intervention by group care to manage type 2 diabetes. Diabetes Care. 2010;33(4):745-7.
169. Vaillant-Roussel H, Laporte C, Pereira B, De Rosa M, Eschalier B, Vorilhon C, et al. Impact of patient education on chronic heart failure in primary care (ETIC): a cluster randomised trial. BMC Fam Pract. 2016;17(1):1-13.
170. van de Ven G, Draskovic I, Adang EM, Donders R, Zuidema SU, Koopmans RT, et al. Effects of dementia-care mapping on residents and staff of care homes: a pragmatic cluster-randomised controlled trial. PloS One. 2013;8(7):e67325.
171. van der Aa HP, van Rens GH, Comijs HC, Margrain TH, Gallindo-Garre F, Twisk JW, et al. Stepped care for depression and anxiety in visually impaired older adults: multicentre randomised controlled trial. BMJ. 2015;351.
172. Vinereanu D, Lopes RD, Bahit MC, Xavier D, Jiang J, Al-Khalidi HR, et al. A multifaceted intervention to improve treatment with oral anticoagulants in atrial fibrillation (IMPACT-AF): an international, cluster-randomised trial. The Lancet. 2017;390(10104):1737-46.
173. Wagenaar KP, Broekhuizen BD, Jaarsma T, Kok I, Mosterd A, Willems FF, et al. Effectiveness of the European Society of Cardiology/Heart Failure Association website 'heartfailurematters. org'and an e-health adjusted care pathway in patients with stable heart failure: results of the 'e-Vita HF'randomized controlled trial. European Journal of Heart Failure. 2019;21(2):238-46.
174. Waldorff F, Buss D, Eckermann A, Rasmussen M, Keiding N, Rishøj S, et al. Efficacy of psychosocial intervention in patients with mild Alzheimer's disease: the multicentre, rater blinded, randomised Danish Alzheimer Intervention Study (DAISY). Bmj. 2012;345.
175. Walters J, Cameron-Tucker H, Wills K, Schüz N, Scott J, Robinson A, et al. Effects of telephone health mentoring in community-recruited chronic obstructive pulmonary disease on self-management capacity, quality of life and psychological morbidity: a randomised controlled trial. Bmj Open. 2013;3(9):e003097.
176. van Wetering CR, Hoogendoorn M, Mol S, Rutten-van Mölken M, Schols A. Short-and long-term efficacy of a community-based COPD management programme in less advanced COPD: a randomised controlled trial. Thorax. 2010;65(1):7-13.
177. Whittle J, Schapira MM, Fletcher KE, Hayes A, Morzinski J, Laud P, et al. A randomized trial of peer-delivered self-management support for hypertension. Am J Hypertens. 2014;27(11):1416-23.

178. Wilcock J, Iliffe S, Griffin M, Jain P, Thuné-Boyle I, Lefford F, et al. Tailored educational intervention for primary care to improve the management of dementia: the EVIDEM-ED cluster randomized controlled trial. Trials. 2013;14(1):1-10.
179. Wright LK, Litaker M, Laraia MT, DeAndrade S. Continuum of care for Alzheimer's disease: a nurse education and counseling program. Issues Ment Health Nurs. 2001;22(3):231-52.
180. Xi F, Wang Z, Qi Y, Brightwell R, Roberts P, Stewart A, et al. Long-term effect of respiratory training for chronic obstructive pulmonary disease patients at an outpatient clinic: a randomised controlled trial. Clin Transl Med. 2015;4(1):1-7.
181. Xiao LD, De Bellis A, Kyriazopoulos H, Draper B, Ullah S. The Effect of a Personalized Dementia Care Intervention for Caregivers From Australian Minority Groups. Am J Alzheimers Dis Other Demen. 2016;31(1):57-67.
182. Zwar NA, Bunker JM, Reddel HK, Dennis SM, Middleton S, van Schayck OC, et al. Early intervention for chronic obstructive pulmonary disease by practice nurse and GP teams: a cluster randomized trial. Family Practice. 2016;33(6):663-70.
183. Fortinsky RH, Kulldorff M, Kleppinger A, Kenyon-Pesce L. Dementia care consultation for family caregivers: collaborative model linking an Alzheimer's association chapter with primary care physicians. Aging Ment Health. 2009;13(2):162-70.
184. Freund T, Peters-Klimm F, Boyd CM, Mahler C, Gensichen J, Erler A, et al. Medical Assistant-Based Care Management for High-Risk Patients in Small Primary Care Practices: A Cluster Randomized Clinical Trial. Ann Intern Med. 2016;164(5):323-30.
185. Liu WT, Wang CH, Lin HC, Lin SM, Lee KY, Lo YL, et al. Efficacy of a cell phone-based exercise programme for COPD. Eur Respir J. 2008;32(3):651-9.
186. Feinstein AR. The Pre-Therapeutic Classification of Co-Morbidity in Chronic Disease. J Chronic Dis. 1970;23(7):455-68.
187. Valderas JM, Starfield B, Sibbald B, Salisbury C, Roland M. Defining comorbidity: implications for understanding health and health services. Ann Fam Med. 2009;7(4):357-63.
